# DFT Studies on *cis*-1,4-Polymerization of Dienes Catalyzed by a Cationic Rare-Earth Metal Complex Bearing an Ancillary PNP Ligand

**DOI:** 10.3390/polym9020053

**Published:** 2017-02-07

**Authors:** Xingbao Wang, Xiaohui Kang, Guangli Zhou, Jingping Qu, Zhaomin Hou, Yi Luo

**Affiliations:** 1 State Key Laboratory of Fine Chemicals, School of Chemical Engineering, Dalian University of Technology, Dalian 116024, China; wangxb@mail.dlut.edu.cn (X.W.); huixiaokang328@163.com (X.K.); zhouguangli.com@163.com (G.Z.); qujp@dlut.edu.cn (J.Q.); 2 College of Pharmacy, Dalian Medical University, Dalian 116044, China; 3 Organometallic Chemistry Laboratory and Center for Sustainable Resource Science, RIKEN, 2-1 Hirosawa, Wako, Saitama 351-0198, Japan

**Keywords:** density functional theory (DFT) calculations, *cis*-1,4-polymerization of dienes, nonmetallocene complexes, rare-earth metal

## Abstract

Dnsity functional theory (DFT) calculations have been carried out for the highly selective *cis*-1,4-polymerization of butadiene catalyzed by a cationic rare-earth metal complex bearing an ancillary PNP ligand. It has been found that the chain initiation and propagation of butadiene polymerization occurs via the favorable *cis*-1,4-insertion route. The *trans*-1,4 and 1,2-insertion are unfavorable both kinetically and thermodynamically. The chain growth follows the π-allyl-insertion mechanism. The analyses of energy decomposition of transition states indicate that the likelihood of rival insertion pathways is predominantly controlled by the interaction energy of butadiene with a metal center and the deformation energy of butadiene moiety. The electronic factor of the central metal has a decisive influence on the *cis-* vs. *trans*-insertion and the regioselectivity (*cis*-1,4- vs. *cis*-1,2-insertion) is mainly determined by steric hindrance. Tetrahydrofuran (THF) coordination made monomer insertion less favorable compared with THF-free case and had more noticeable impact on the *trans*-monomer insertion compared with the *cis* case. During the chain propagation, *cis*-insertion of monomer facilitates THF de-coordination and the THF molecule could therefore dissociate from the central metal.

## 1. Introduction

The microstructures of polyisoprene (PIP) and polybutadiene (PBD) have significant influences on its physical and chemical properties, and hence for certain usage [1,2,3,4]. *cis*-1,4 selective polymerization of isoprene and butadiene is a very important process in the chemical industry to provide products that are among the most significant and widely used rubbers [5,6]. Generally, it is believed that a slight increase in the *cis*-1,4 regularity of the product leads to a great improvement in the elastic properties [6,7]. Therefore, the synthesis of PIP and PBD with high *cis*-1,4-selectivity has attracted considerable interest. In this content, transition metal complexes have been widely used as precatalysts, which showed excellent performance in isoprene and butadiene polymerization [8,9,10]. However, catalyst systems showing high both activity and stereoselectivity for isoprene and butadiene polymerization remained less. Therefore, further exploration of new and highly active catalysts for stereoselective synthesis of such polymers with narrow molecular-weight distribution and desired microstructure is obviously attractive.

**Chart 1:**
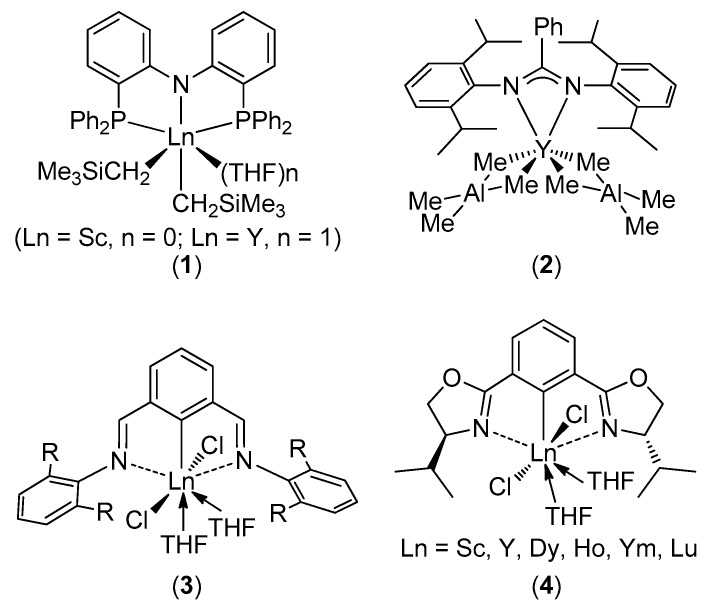
Representative examples of non-metallocene rare-earth metal complexes for *cis*-1,4-polymerization of isoprene. (**1**) ({2-(Ph_2_P)C_6_H_4_}_2_N)Ln(CH_2_SiMe_3_)_2_(thf)_n_; (**2**) (PhC(NC_6_H_4_^*i*^Pr_2_-2,6)_2_)YMe_2_(AlMe_3_)_2_; (**3**) (2,6-(2,6-C_6_H_3_R_2_N=CH)_2_-C_6_H_3_)LnCl_2_(THF)_2_; (**4**) ((*S*,*S*)-Phebox-^*i*^Pr)LnCl_2_(THF)_2_).

Cationic rare-earth metal alkyl species with various ancillary ligands were reported to show high activity and *cis*-1,4-selectivety in the polymerization of isoprene and butadiene. Among which, besides metallocene rare earth metal complex [4,11,12,13,14,15,16,17], nonmetallocene complexes were also reported for the *cis*-1,4-selective polymerization of isoprene and butadiene [4,7,18,19,20,21,22,23,24,25]. Notably, Hou and coworkers had reported a best-defined catalyst system based on a cationic alkyl Y metal species bearing an ancillary PNP (PNP^Ph^ = [2-(Ph_2_P)C_6_H_4_]_2_N) ligand, [(PNP^Ph^)Y(CH_2_SiMe_3_)]^+^ (**1**, Chart 1) [18]. This non-metallocene rare-earth metal catalyst with no need of any aluminum additives exhibited extremely high *cis*-1,4 selectivity (>99%) and excellent livingness for the polymerization of isoprene and butadiene. Subsequently, the same group had demonstrated that an amidinate-ligated aminobenzylyttrium complex, in combination with [Ph_3_C][B(C_6_F_5_)_4_], is an excellent catalyst system for the 3,4-polymerization of isoprene [19]. However, the addition of a small amount of AlMe_3_ (3–5 equivalents) to the system led to the formation of complex **2** (Chart 1). Interestingly, upon activation, **2** shows high *cis*-1,4-selectivity toward isoprene polymerization [19]. The NCN-pincer stabilized rare earth metal chlorides (**3** and **4**), in combination with aluminum trialkyl and arylborate salt, were also reported to be highly active and *cis*-1,4-selective catalyst systems toward isoprene polymerization [7,20]. Besides the *cis*-1,4- and 3,4-selective polymerizations [19,26], non-metallocene rare-earth metal complexes also showed excellent activity toward highly *trans*-1,4-(co)polymerizations of conjugated dienes [27]. However, compared with the experimental synthesis of such polymers, the related mechanism and factors governing the selectivity for these catalytic systems have remained unclear.

Computational analysis on the mechanism of various olefin polymerizations has led to a new level of insight in the electronic structure features governing reactivity and selectivity [28,29,30,31,32,33,34,35,36,37,38,39,40,41,42]. Based on DFT studies, Maron and co-workers clarified and rationalized the observed preference for *cis*-1,4 polymerization of isoprene by a cationic metallocene rare-earth [Cp*ScR]^+^ complex [29]. In their study, the difference in energy barrier between the *cis*-1,4 and *trans*-1,4 butadiene insertions into [Cp*Sc(η^3^-*syn*-C_8_H_13_)]^+^ correlated with the difference in butadiene coordination to the metal center. The mechanistic study of the copolymerization of ethylene with butadiene catalyzed by neodymocene complexes has been presented in the literature [41]. This study also offers a survey of the ligand effects on the activity of catalysts and the microstructure of the resulting copolymers. Our group reported a DFT study on *trans*-1,4-specific polymerization of isoprene catalyzed by the cationic heterobimetallic half-sandwich complex [(C_5_Me_5_)La(AlMe_4_)]^+^ [35]. The results showed that the AlMe_3_ serves as a ligand to regulate polymerization selectivity. In comparison with computational studies on the polymerization of isoprene and butadiene catalyzed by metallocene rare-earth catalysts, the investigation on the mechanistic details of the polymerization of isoprene or butadiene catalyzed by non-metallocene rare-earth species is very limited. 

Herein, we present the results of DFT calculations on the possible mechanism for the isoprene and butadiene polymerization catalyzed by a non-metallocene rare-earth catalyst. The cationic alkyl complex [(PNP^Ph^)Y(CH_2_SiMe_3_)]^+^ (**1^+^**, derived from **1** in Chart 1) [18] was selected as a model catalyst for two reasons: (a) This catalyst system provides extremely high *cis*-1,4 selectivity for the polymerization of isoprene and butadiene; (b) There was no need to introduce aluminum additives to the catalyst system, which is a benefit to the mechanistic study. Considering that the polymerizations of butadiene and isoprene showed the same selectivity [18], we considered the case of butadiene. In some cases, isoprene was considered to further confirm our conclusion. Alongside this investigation, we aim to explore the intrinsic factors controlling its regio- and stereo-chemistry and provide fundamental understandings and guidelines for the development of efficient and structurally tailorable catalysts.

## 2. Computational Details

All the DFT calculations were performed with the Gaussian 09 program [43]. The B3PW91 [44,45] hybrid exchange-correlation functional was used for geometry optimization and subsequent frequency calculation. The 6-31G* basis set was used for H, C, O, and N atoms, and the P, Si, and Y atoms were treated by the Stuttgart/Dresden effective core potential (ECP) and the associated basis sets. The basis sets for the Si and P atoms were augmented by one *d*-polarization function with exponents of 0.284 and 0.389, respectively. Such basis sets are donated as BSI. Single point energy calculations were also performed for the B3PW91/BSI geometries by using the M06 [46] functional and a larger basis set BSII. In the BSII, 6-31+G** was used for H, C, O, and N atoms, and the basis set for Si, P, and Y atoms is same as that in geometry optimizations. In these single-point calculations, solvation effects were considered with the IEFPCM model [47]. Toluene (ε = 2.37) was employed as a solvent in the IEFPCM solvation calculations. The reported Gibbs free energies in solution were calculated at the level of M06(IEFPCM)/BSII, including the corresponding thermal corrections derived from gas-phase frequency calculations.

## 3. Results and Discussion

**Scheme 1:**
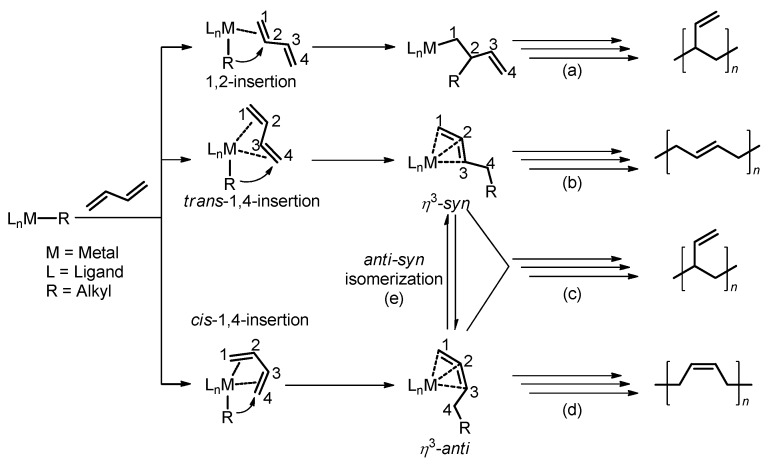
Possible reaction pathways for butadiene polymerization catalyzed by rare-earth metal complexes.

It is well recognized that the chain initiation proceeds by the insertion of butadiene into the metal–alkyl bond [48]. This leads to a species with butenyl moiety, which is ligated to the metal center in an η^3^ mode. In general, there are eight different coordination modes of butadiene to the central metal. However, due to the symmetry of cationic catalyst backbone, the pairs of stereoisomers are identical. Therefore, only four different modes of butadiene coordination (η^2^-*cis*, η^2^-*trans*, η^4^-*cis*, η^4^-*trans*) and two different structures of the butenyl end group (η^3^-*anti*, η^3^-*syn*) were considered to elucidate of the mechanism of stereoregulation. The reported coordination/insertion forms are corresponding favorable ones. Based on the previous computational studies [28,31,35,48], one may propose that the reaction process for polymerization of butadiene catalyzed by rare-earth metal complexes has five possible pathways (Scheme 1): (a) 1,2-insertion of butadiene into the metal–alkyl bond to form 1,2-polybutadiene; (b) *trans*-butadiene 1,4-insertion into the terminal of η^3^-*syn*-metal–allyl linkage (M–C1) to form *trans*-1,4-polybutadiene; (c) butadiene insertion into the internal of η^3^-metal–allyl linkage (M–C3) to form 1,2-polybutadiene; (d) *cis*-butadiene 1,4-insertion into the terminal of η^3^-*anti*- metal–allyl linkage (M–C1) to form *cis*-1,4-polybutadiene; (e) interconversion of the *syn* and *anti* forms of the metal–allyl linkage to form corresponding polybutadiene.

### 3.1. Cationic Species

**Figure 1:**
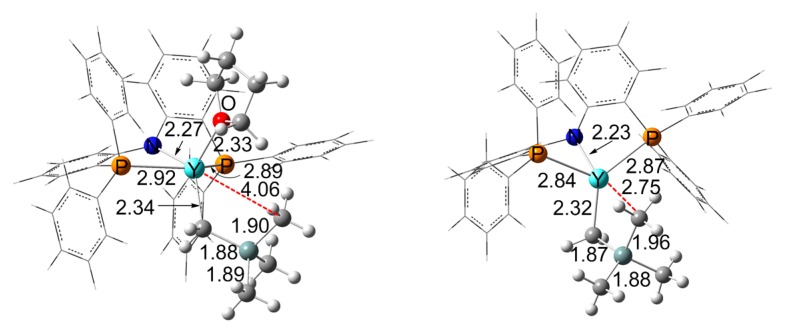
Optimized structures of cationic species **^thf^A** (**left**) and **A** (**right**) (distances in Å).

Experimentally, monitoring the reaction of [(PNP^Ph^)Y(CH_2_SiMe_3_)_2_(thf)] with one equivalent of [PhMe_2_NH][B(C_6_F_5_)_4_] in C_6_D_5_Cl by ^1^H NMR spectroscopy showed the formation of [(PNP^Ph^)Y–(CH_2_SiMe_3_)(thf)]^+^ (**^thf^A**) [18]. This result suggested that **^thf^A** may be the true active species during the polymerization process. A previous theoretical study on a metallocene system suggested that THF-coordinated cationic species worked in chain initiation, but THF-free cationic complex was the true active species during the chain propagation [36]. In order to investigate the effect of THF on the current butadiene polymerization, both **^thf^A** and THF-free cationic species ([(PNP^Ph^)Y–(CH_2_SiMe_3_)]^+^, **A**) were considered in the present study. As shown in Figure 1, the tridentate coordination mode is retained in the cationic species. The optimized structure of **A** shows an significant agostic interaction between metal center and a methyl carbon atom of the SiMe_3_ group, as manifested by the short Y∙∙∙C contact (2.75 Å) and elongated Si–C bond length (1.96 Å) in comparison with the normal Si–C bond length of 1.88 Å. In **^thf^A**, the distance of Y∙∙∙C is 4.06 Å and Si–C bond length (1.90 Å) is similar to the normal Si–C bond length. Due to the coordination of THF, the bond length of Y–P in **^thf^A** is longer than that in **A**. It is noteworthy that the activation by [PhMe_2_NH][B(C_6_F_5_)_4_] resulted in a discrete contacted ion pair [18]. In this ion pair, the fluoroaryl borates anion did not directly coordinate to the central metal, but just had weak electrostatic interaction with the cationic species. This experimental finding suggests that the anion could have a negligible effect on the polymerization mechanism. In previous theoretical studies, the effect of counterions was also neglected when predicting the stereoselectivity and regioselectivity [30,35,49], it is reasonable to assume that the magnitude of the effect of anion should be the same for all different types of insertions. Thus, the minor effect of fluoroaryl borates on the prediction of stereoselectivity and regioselectivity was not considered in this study.

### 3.2. Chain Initiation Stage

**Figure 2:**
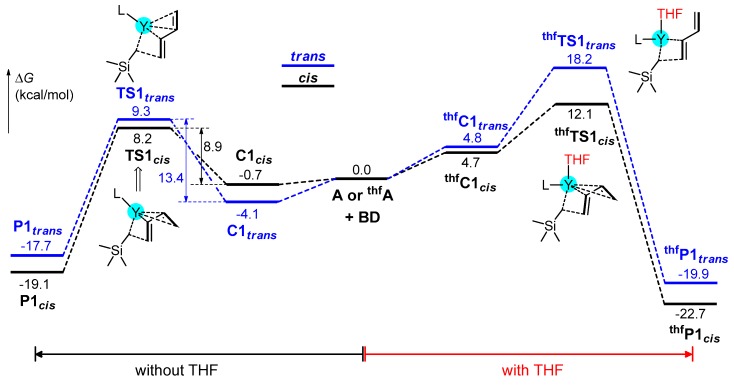
Computed energy profiles (∆*G* in kcal/mol) for insertion of the first butadiene monomer into the Y–alkyl σ-bond of the cationic species **^thf^A** or **A**.

To investigate the chain initiation of butadiene polymerization, the energy profiles for the insertions of *cis*- and *trans*-butadiene into the Y–alkyl bond of cationic species **^thf^A** and **A** were computed, respectively. As shown in Figure 2, in the case of both with and without THF, the *cis*-insertion pattern is more favorable both kinetically and thermodynamically. The presence of THF results in a higher energy barrier (12.1 vs. 8.9 kcal/mol, Figure 2). This is consistent with the previous studies [36]. It is noted that the difference in energy barrier between with and without THF cases (∆∆*G*^‡^ = ∆^thf^*G*^‡^ − ∆*G*^‡^) are 4.8 (18.2 − 13.4 = 4.8) and 3.2 (12.1 − 8.9 = 3.2) kcal/mol for the insertions of *trans*- and *cis*-conformers, respectively (Figure 2). This result suggests that THF coordination made monomer insertion less favorable compared with the THF-free case and had more noticeable impact on the *trans*-monomer insertion compared with the *cis* case. The computed results indicate that the 1,2-insertion has higher energy barrier and is less competitive (∆*G*^‡^_1,2_ = 16.0 kcal/mol for **^thf^A** case and 19.6 kcal/mol for **A** case, see Figures S1 and S2 in the Supporting Information). Therefore, the 1,2-insertion will be not considered in the following calculations.

**Figure 3:**
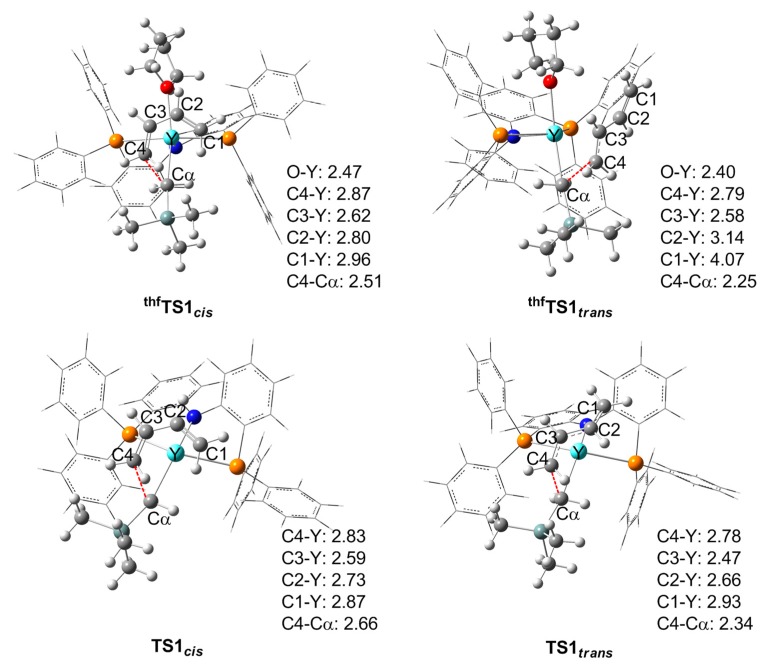
Transition state structures (distances in Å) for the *cis*- and *trans*-insertion of butadiene into the Y–alkyl σ-bond of the cationic species **^thf^A** or **A**.

The transition structures of **^thf^TS1_*cis*_** and **^thf^TS1_*trans*_** for the **^thf^A** case and **TS1_*cis*_** and **TS1_*trans*_** for the **A** case are shown in Figure 3. As indicated in this Figure, *cis*-butadiene coordinates to the metal center via η^4^-pattern, as suggested by the Y∙∙∙C*_i_* (*i* = 1, 2, 3, and 4) distances (2.5–2.9 Å). However, *trans*-butadiene coordinates to the metal center via η^2^-pattern in the **^thf^TS1_*trans*_** due to the repulsive interaction between butadiene and THF moiety. The geometric data in Figure 3 suggest that the barrier difference between *cis* and *trans* insertions could be ascribed to the degree of C4–Cα bond formation in the **TS1** species along with the reaction coordinates. That is, the distances of C4∙∙∙Cα in **TS1_*cis*_** and **^thf^TS1_*cis*_** are much longer than that in **TS1_*trans*_** and **^thf^TS1_*trans*_**, suggesting that the *cis* insertion could reach the transition state earlier than the *trans*-insertion. Moreover, the distances of C4∙∙∙Cα in **TS1** are much longer than that in **^thf^TS1**, also suggesting that the insertion event in the case of no THF could reach the transition state earlier compared to the case of THF-coordinated species.

### 3.3. Chain Propagation Stage

Two mechanisms have been proposed for the chain propagation step [28]. One is the σ-allyl-insertion mechanism (η^1^-σ-metal–allyl form) assumed by Cossée and Arlmann [50,51]. The other is the π-allyl-insertion mechanism (η^3^-π-metal–allyl form) proposed by Taube and coworkers [52]. During this stage, the chain propagation and its stereoselectivity and regioselectivity have been investigated.

**Scheme 2:**
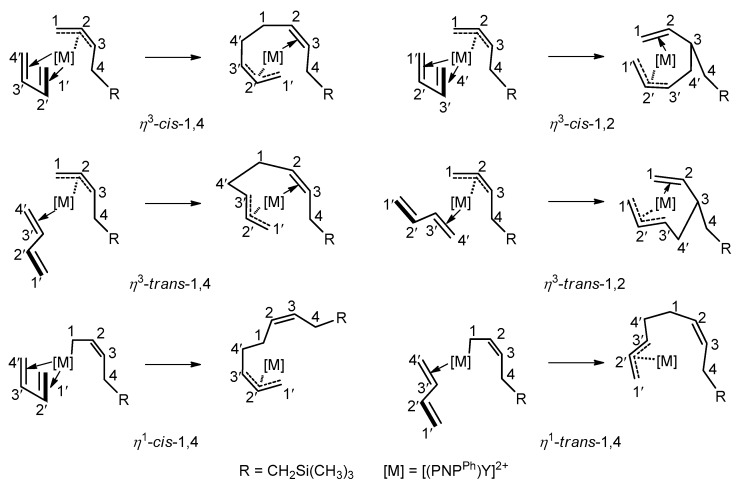
Proposed patterns for insertion of *cis*- and *trans*-butadiene into the *anti*-butenyl–Y linkage, affording 1,4- and 1,2-butadiene polymer unit.

The configurations of the incoming butadiene and butenyl units as well as their enantiofaces involved in chain propagation are of importance for the regio- and stereo-selectivity in the polymerization process. Scheme 2 depicts the stereoisomers of *cis*- or *trans*-butadiene and *anti*-butenyl–Y linkages formed by favorable *cis*-butadiene insertion in the chain initiation step. All the possible isomers of key stationary points, which participate in the various pathways for chain propagation, have been carefully explored. According to Scheme 2, 1,4-units can be obtained through both σ-allyl-insertion and π-allyl-insertion mechanisms, in which butadiene monomer inserts into the terminal carbon atom (C1). However, 1,2-units only can be obtained from the insertion of butadiene into Y–C3 bond, following the π-allyl-insertion mechanism. 

#### 3.3.1. With THF

The energy profile for insertion of the second butadiene into metal–allyl bond of **^thf^P1_*cis*_** is calculated for modeling chain propagation, and the results are summarized in Table 1. As shown in this table, the π-complex **η^3^**-**^thf^C2_*cis*_** with η^3^-*cis* form is more stable than **η^1^**-**^thf^C2_*cis*_** featuring η^1^-*cis* fashion by ca. 8 kcal/mol. The attempts to locate a corresponding **η^1^**-**^thf^C2_*trans*_** complex were fruitless, but resulted in a **η^3^**-**^thf^C2_*trans*_** species instead. These results suggest that the haptotropic shift from η^3^ to η^1^ species is thermodynamically unfavorable. After the formation of the pre-reaction complex (**^thf^C2**), the insertion of butadiene occurs through the transition state **^thf^TS2** and then leads to the insertion product **^thf^P2**. The results indicate that insertion of the second butadiene into **^thf^P1_*cis*_** prefers the π-allyl-insertion rather than σ-allyl-insertion. The η^3^-*cis*-1,4 insertion is computed to be the most favorable process as suggested by the lowest activation barrier of 28.6 kcal/mol for **η^3^**-**^thf^TS2_*cis*-1,4_**. For the η^3^-*cis*-1,4 insertion of isoprene, the lowest activation barrier is 37.6 kcal/mol. However, these energy barriers are too high when compared with the excellent livingness at room temperature in experiment [18]. Moreover, the energy barrier gap between the η^3^-*cis*-1,4 insertion and other insertion fashions is not large enough (∆∆*G*^‡^ = 1.6–3.2 kcal/mol), which is inconsistent with the extremely high *cis*-1,4 selectivity (>99%) observed in the present system [18].

**Figure 4:**
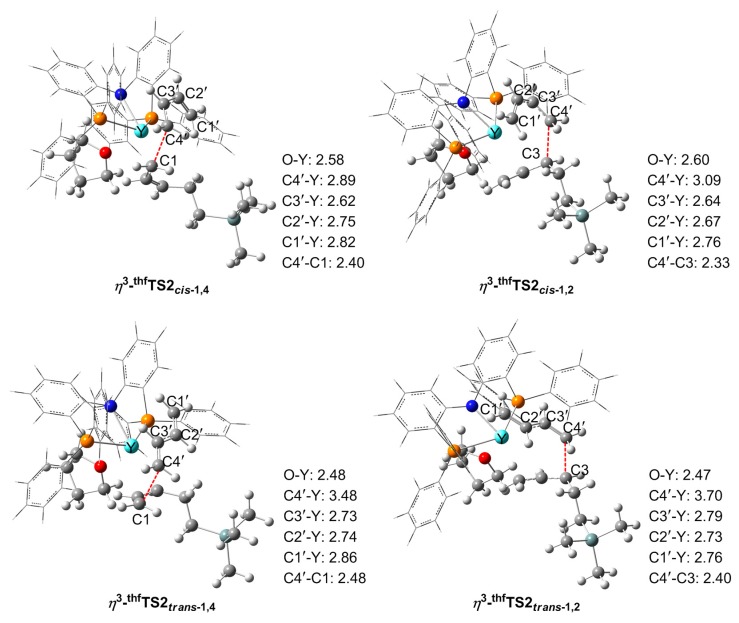
Transition state structures (distances in Å) for the *cis*- and *trans*-insertions of butadiene into the Y–allyl π-bond of the **^thf^P1_*cis*_**.

When the transition structures are analyzed (Figure 4), it is found that the Y···O_thf_ distances of 2.58 and 2.60 Å in **η^3^**-**^thf^TS2*_cis-_*_1,4_** and **η^3^**-**^thf^TS2*_cis-_*_1,2_** are longer than that in **η^3^**-**^thf^TS2*_trans-_*_1,4_** (2.48 Å) and **η^3^**-**^thf^TS2*_trans-_*_1,2_** (2.47 Å). This means that the *cis*-insertion of the second butadiene facilitates the dissociation of THF molecule from the metal center.

#### 3.3.2. Without THF

**Figure 5:**
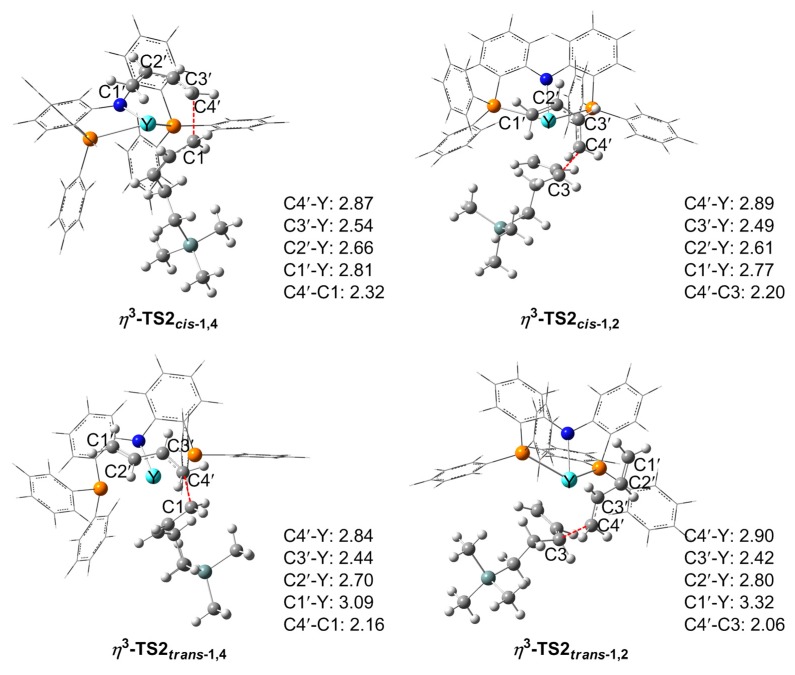
Transition state structures (distances in Å) for the *cis*- and *trans*-insertion of butadiene into the metal–allyl π-bond of the **P1_*cis*_**.

As the insertion of the second monomer dose in the case of **^thf^P1_*cis*_**, both *cis*- and *trans*-insertions of the incoming monomer into the metal–allyl π-bond of **P1_*cis*_** were calculated. The calculated energies are listed in Table 2 and the structures of transition state are shown in Figure 5. Like the insertion into **^thf^P1_*cis*_**, the insertion of the second butadiene into **P1_*cis*_** also prefers the π-allyl-insertion rather than σ-allyl-insertion. The comparison of the energy profile of the most feasible pathways reveals that *cis*-insertions are distinctly preferred against the *trans*-insertions. The significant kinetic gap (∆∆*G*^‡^ = 7.9–10.7 kcal/mol) between the *cis* and *trans* forms indicates that *trans*-insertion into the metal–C bond would be uncompetitive. As shown in Table 2, the *cis*-1,4-insertion into the metal–allyl π-bond to generate an expected *cis*-1,4 sequence is most favorable, which has the lowest energy barrier (∆*G*^‡^ = 6.9 kcal/mol) and is much more exergonic (∆*G* = −16.5 kcal/mol), suggesting that the π-allyl-insertion mechanism works in the current system. These results are in good agreement with the experimental observation [18], in which no *trans*-1,4 polyisoprene (∆∆*G*^‡^ = 14.8 − 6.9 = 7.9 kcal/mol) was observed and small fraction 3,4-polyisoprene (∆∆*G*^‡^ = 10.5 − 6.9 = 3.6 kcal/mol) was included in the resultant polymer.

As shown in Figure 5, the transition states appear at the distance of 2.16–2.32 Å of the emerging C4′−C1 σ-bond to form a 1,4-sequence and that of 2.06–2.20 Å of the emerging C4′–C3 σ-bond to form 1,2-sequence. They are significantly smaller than that for insertion of the second butadiene into **^thf^P1_*cis*_**. Therefore, the distance of the emerging C–C bond in transition state is related to the coordination number of the metal center: the larger the coordination number, the longer of the C–C distance. This is coincident with the previous results reported by Tobisch [53].

The interconversion of *anti* and *syn* forms of the metal–allyl linkage is another important elementary step, which may play a decisive role in *cis-trans* stereoregulation of the polymerization process. Our results indicate that the isomerization of the **^thf^P1_*cis*_** to **^thf^P1_*trans*_** needs a higher energy barrier (∆*G*^‡^ = 23.9 kcal/mol) compared to the insertion of the second monomer and is endergonic (∆*G* = 2.8 kcal/mol). It is noted that many attempts to locate a transition state for interconversion (isomerization) between **η^3^**-**P2_*cis*_** and **η^3^**-**P2_*trans*_** were fruitless. This could be ascribed to the highly steric crowding around the metal center. The DFT study by Tobisch [28] and our previous work [35] showed that allylic isomerization is more kinetically demanding than butadiene or isoprene insertion. Therefore, the interconversion of *anti*- and *syn*-forms could be excluded in the present butadiene polymerization system.

**Figure 6:**
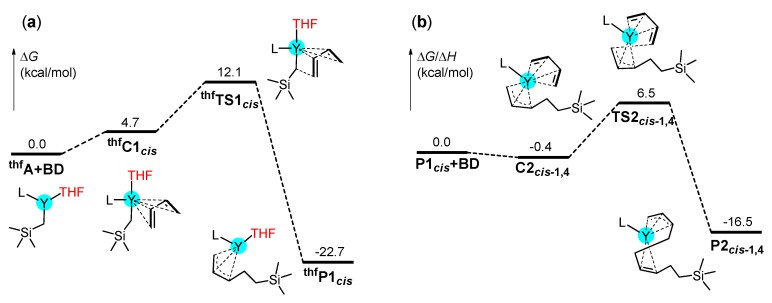
Energy profiles (∆*G* in kcal/mol) for the most favorable pathway: (**a**) Chain initiation during which THF coordinates to a metal center; (**b**) Chain propagation during which there is no THF coordination to the metal.

For better understanding, the most feasible pathway is collected in Figure 6. As shown in this Figure, the insertion of the first monomer is predicted to have a higher energy barrier than that of the second one. The former needs to amount an energy barrier of 12.1 kcal/mol and is driven by thermodynamics (exergonic by 22.7 kcal/mol, Figure 6). After the chain initiation, the THF molecule could dissociate from the metal center. The insertion of the second monomer overcomes a free energy barrier of 6.9 kcal/mol and is exergonic by 16.5 kcal/mol. For the η^3^-*cis*-1,4 insertion of isoprene, the lowest energy barrier is 11.8 kcal/mol and is exergonic by 12.5 kcal/mol. Such energy barrier values are relatively low, accounting for the high activity observed experimentally [18].

### 3.4. Effect of THF

As previously mentioned, the THF-free cationic species could be the true active species during the chain propagation with respect to the observed extremely high *cis*-1,4 selectivity and excellent livingness. However, in general, the coordination of THF molecule to the metal center is relatively strong. The dissociation free energy of THF from metal center is 20.1 kcal/mol for **^thf^A**, which is higher than the free energy barriers for the butadiene insertion into **A** (8.9 kcal/mol, Figure 2) and **^thf^A** (12.1 kcal/mol). For **^thf^P1_*cis*_**, the THF dissociation free energy is 24.8 kcal/mol, which is lower than the free energy barrier for the butadiene insertion into **^thf^P1_*cis*_** (28.6 kcal/mol, Table 1). It is found that the coordination energy of THF is larger than that for butadiene. Thus, butadiene displaces THF molecules only from a small equilibrium fraction of the active species. Once THF molecule dissociates from the metal center, the insertion of butadiene monomer will continuously occur until THF molecule re-coordination. Thus, the butadiene polymerization would be an “intermittent” event. This phenomenon is similar to the coordination of counterions with cationic species [54]. However, under the experimental condition, the concentration of butadiene monomer is significantly higher than that of THF in the solution. In this sense, successive insertion of a monomer could smoothly occur. 

It is noteworthy that the free energy barrier for the butadiene insertion into the THF-coordinating species **^thf^P1_*cis*_** (28.6 kcal/mol) is lower than the sum (24.8 + 6.9 = 31.7 kcal/mol) of the dissociation free energy of THF in **^thf^P1_*cis*_** and the free energy barrier for the butadiene insertion into the THF free-species **P1_*cis*_**. However, in the case of isoprene, the situation is opposite (37.6 vs. 20.9 + 11.8 = 32.7 kcal/mol). These results could be responsible for the previous butadiene polymerization experiment [18], where a certain amount of Al^*i*^Bu_3_ was used for the purpose of purification and possibly facilitating THF dissociation. However, for the polymerization of isoprene, there was no need to introduce aluminum additives to the reaction system. In addition, the experimental study also showed that the reaction of [(PNP^Ph^)Y(CH_2_SiMe_3_)_2_(thf)] with one equivalent of [PhMe_2_NH][B(C_6_F_5_)_4_] in THF resulted in the formation of [(PNP^Ph^)Y–(CH_2_SiMe_3_)(thf)_2_]^+^. However, this *bis*(thf)-coordinated cationic complex was inactive under the experimental conditions [18]. This result indirectly indicated that THF coordination hampered the diene polymerizations.

As discussed above, the current results suggest that, in chain initiation step, the *trans*-monomer insertion was kinetically unfavorable in the case of THF coordination compared with the case of THF-free. The insertion of *cis*-butadiene is more beneficial to the dissociation of THF molecules from the metal center than *trans*-conformer. Additionally, THF could depart from the metal center during chain growth.

### 3.5. The Origin of the Selectivity

To elucidate the origin of the kinetic preference for such a selectivity, we further analyzed the geometries and energies of **η^3^**-**TS2_*cis*-1,4_**, **η^3^**-**TS2_*cis*-1,2_**, and **η^3^**-**TS2_*trans*-1,4_**. An analysis of the energy decomposition of **η^3^**-**TS2** was performed [55]. The energies of the fragments [(PNP^Ph^)Y]^2+^ (**PNP**), butadiene (**Bu**), and [(C_4_H_6_)(CH_2_SiMe_3_)]^−^ (**Chain**) in the geometry they have in the three transition states were evaluated via single-point calculations. Such single-point energies of the fragments and the energy of transition states were used to estimate the interaction energy *E*_int_. These energies, together with the energy of the respective fragments in their optimal geometry, allow for the estimation of the deformation energies of the three fragments, ∆*E*_def_ (**PNP**), ∆*E*_def_ (**Bu**), and ∆*E*_def_ (**Chain**). As the energy of the transition state, ∆*E*_TS_, is evaluated with respect to the energy of the three separated fragments, the relation ∆*E*_TS_ = ∆*E*_int_ + ∆*E*_def_ (**PNP**) + ∆*E*_def_ (**Bu**) + ∆*E*_def_ (**Chain**) holds. 

As shown in Table 3, the interaction of butadiene and propagation chain with the metal center in the *cis*-transition-state is stronger than in the *trans*-analog. Although the formation of *cis*-adducts is less favorable energetically (Table 2) than that of the *trans*-analog, its geometry efficiently prepares for the transition state because the energy barrier for *cis*-insertion is lower than that for *trans*-insertion. Therefore, the higher energy barrier for *trans*-1,4 insertion could be attributed to the weak interaction among the fragments in **η^3^**-**TS2_*trans*-1,4_**, which further could be responsible for the unfavorable *trans*-1,4 selectivity. This is consistent with the previous DFT study for *cis*-1,4 polymerization of butadiene by cationic [Cp*ScR]^+^ complex [29]. The *trans*-1,4 polymerization by terpyridine-iron catalyst was attributed to the electronic ground, while steric factors had a small influence [28]. Structurally, the butadiene is closer to the metal center in the *cis* transition structure than that in the *trans* one (as shown in Figure 5). The C1′–Y distance is 3.08 Å in the **η^3^**-**TS2_*trans*-1,4_**, which is further than in the **η^3^**-**TS2_*cis*-1,4_** (2.81 Å) and **η^3^**-**TS2_*cis*-1,2_** (2.77 Å). Thus, for the non-metallocene metal complexes, the electronic factor of the central metal has a decisive influence on the *cis-* vs. *trans*-insertion, which further determines the stereoselectivity.

Table 3 shows that the higher energy of **η^3^**-**TS2_*cis*-1,2_** could be ascribed to the higher deformation of butadiene. Many experimental studies revealed that the regioselectivity of diene polymerization catalyzed by rare-earth metal complex is sensitive to the degree of exposure of the central metal [7,18,19,56]. Structurally, for the tridentate PNP catalyst in this study, the angle of P–Y–P is 126.7° in the **η^3^**-**TS2_*cis*-1,2_**, which is larger than that in the corresponding transition structure (67.8°) for 3,4-specific isoprene polymerization catalyzed by NSN-tridentate rare-earth metal catalyst [56]. The central metal is less exposed in the PNP catalyst than in the NSN catalyst, which results in higher deformation energy for the 1,2-inserion. In this sense, for the non-metallocene rare-earth metal catalysts, the degree of exposure of the central metal could be vital to the regioselectivity of diene polymerizations.

## 4. Conclusions

Presented herein is a computational study on the mechanism of *cis*-1,4-polymerization of butadiene catalyzed by a cationic rare-earth metal complex [(PNP^Ph^)Y(CH_2_SiMe_3_)(THF)]^+^. The THF-coordinating cationic species might actively work at the chain initiation stage. However, the THF-free cationic species could be the true active species during the chain propagation process with respect to the high activity observed experimentally. Alternative pathways of all crucial elementary steps have been carefully explored by means of DFT calculations. The *cis*-butadiene coordinates to the metal center preferably in an η^4^-mode, however, *trans*-butadiene prefers an η^2^-mode. In the chain initiation step, the generation of the *anti*-η^3^-allyl species via the *cis*-butadiene insertion route is found to be viable on the both kinetic and thermodynamic grounds. The *trans*-1,4 and 1,2-insertions are unfavorable both kinetically and thermodynamically compared with *cis*-1,4-insertion both with and without THF coordinations. The *trans*-monomer insertion was more kinetically impressed in the case of THF-coordinating active species in comparison with the uncoordinating one. Chain growth has been found to follow the π-allyl-insertion mechanism and takes place by sequential *cis*-η^4^-butadiene insertion into the η^3^-allyl terminal group. The analysis of the energy decomposition indicates that the interaction of butadiene with the metal center and the deformation of the incoming butadiene play an important role in determining the stereo- and regioselectivity. The electronic factor of central metal has a decisive influence on the *cis*- vs. *trans*-insertion and the regioselectivity is mainly controlled by steric hindrance. The *cis*-insertion facilitates the de-coordination of THF, which could thus dissociate from the central metal during the chain propagation. This study is successful in rationalizing the experimentally observed preference for *cis*-1,4-polymerization of isoprene and butadiene catalyzed by a rare-earth metal alkyl complex, [(PNP^Ph^)Y(CH_2_SiMe_3_)(THF)]^+^. To the best of our knowledge, this is the first systematic investigation to explore the mechanistic details of the *cis*-1,4-polymerization of isoprene and butadiene catalyzed by non-metallocene rare-earth metal complexes. The current results could be helpful for understanding the factors determining the regio- and stereoselectivity of diene polymerization catalyzed by non-metallocene rare-earth metal complexes.

## Figures and Tables

**Table 1:** Energy profiles ^*a*^ for insertion of the second butadiene into the metal–butenyl bond of **^thf^P1_*cis*_**.

Mechanism	Insertion Fashions	^thf^C2	^thf^TS2	^thf^P2	∆*G*^‡^
π-allyl-insertion	η^3^-*cis*-1,4	13.0	28.6	−4.9	28.6
	η^3^-*cis*-1,4 ^*b*^	15.4	37.6	1.2	37.5
	η^3^-*cis*-1,2	11.8	30.8	−2.7	30.8
	η^3^-*trans*-1,4	9.2	31.8	−3.3	31.8
	η^3^-*trans*-1,2	14.3	30.2	−2.3	30.2
σ-allyl-insertion	η^1^-*cis*-1,4	21.4	29.2	−6.3	29.2
	η^1^-*trans*-1,4	n.a. ^*c*^	35.1	−3.2	35.1

^*a*^ Energies (kcal/mol) are relative to the energy sum of the isolated free reactants, i.e., the **^thf^P1_*cis*_** and *trans*-butadiene monomer; ^*b*^ Isoprene was considered; ^*c*^ The attempts to locate corresponding species were fruitless.

**Table 2:** Energy Profiles ^*a*^ for insertion of the second butadiene into the metal–butenyl bond of **P1_*cis*_**.

Mechanism	Insertion fashions	C2	TS2	P2	∆*G*^‡^
π-allyl-insertion	η^3^-*cis*-1,4	−0.4	6.5	−16.5	6.9
	η^3^-*cis*-1,4 ^*b*^	3.5	11.8	−12.5	11.8
	η^3^-*cis*-1,2	3.5	10.5	−8.6	10.5
	η^3^*-trans*-1,4	−5.4	9.4	−5.3	14.8
	η^3^*-trans*-1,2	−2.1	19.1	−14.0	21.2
σ-allyl-insertion	η^1^-*cis*-1,4	n.a. ^*c*^	19.1	−2.9	≥19.1
	η^1^*-trans*-1,4	−1.8	22.2	−4.0	24.0

^*a*^ Energies (kcal/mol) are relative to the energy sum of the isolated free reactants, i.e., the **P1_*cis*_** and *trans*-butadiene monomer; ^*b*^ Isoprene was considered; ^*c*^ The attempts to locate corresponding species were fruitless.

**Table 3:** Energy decomposition analysis (kcal/mol) of the transition states for the second monomer insertion into **P1_*cis*_**. The relative interaction energies and decomposition energies are given in kcal/mol.

η^3^-TS2	∆*E*_int_	∆*E*_def_ (PNP)	∆*E*_def_ (Bu)	∆*E*_def_ (Chain)	∆*E*_def_^*a*^	∆*E*_TS_
**η^3^-TS2_*cis*-1,4_**	−4.4	2.2	1.2	1.2	4.6	0.2
**η^3^-TS2_*cis*-1,2_**	−5.0	1.0	6.3	0.0	7.3	2.3
**η^3^-TS2_*trans*-1,4_**	0.0	0.0	0.0	2.8	2.8	2.8

^*a*^ ∆*E*_def_ = ∆*E*_def_ (PNP) + ∆*E*_def_ (Bu) + ∆*E*_def_ (Chain).

## Supplementary Materials

The following are available online at www.mdpi.com/2073-4360/9/2/53/s1, Figure S1: the energy profile for the first butadiene 1,2-insertion into the Y–alkyl σ-bond of the cationic species **^thf^A** or **A**; Figure S2: the transition structures for the first butadiene 1,2-insertion into the Y–alkyl σ-bond of the cationic species **^thf^A** or **A**; Optimized coordinates (Å): the optimized Cartesian coordinates with the self-consistent field (SCF) energies (at M06(IEFPCM)/BSII//B3PW91/BSI level) of all structures and the imaginary frequencies of transition states.
