# Supplementary material for: DFT Studies on cis-1,4-Polymerization of Dienes Catalyzed by a Cationic Rare-Earth Metal Complex Bearing an Ancillary PNP Ligand
Source: Polymers (Basel). 2017 Feb 7;9(2):53. doi: 10.3390/polym9020053 (PMC6431986; doi:10.3390/polym9020053)
Supplement: Supplementary file 1 [file polymers-09-00053-s001.pdf]

Supplementary Materials: DFT Studies on *cis*−1,4−Polymerization of Dienes Catalyzed by a Cationic Rare−Earth Metal Complex Bearing an Ancillary PNP Ligand

Xingbao Wang, Xiaohui Kang, Guangli Zhou, Jingping Qu, Zhaomin Hou and Yi Luo

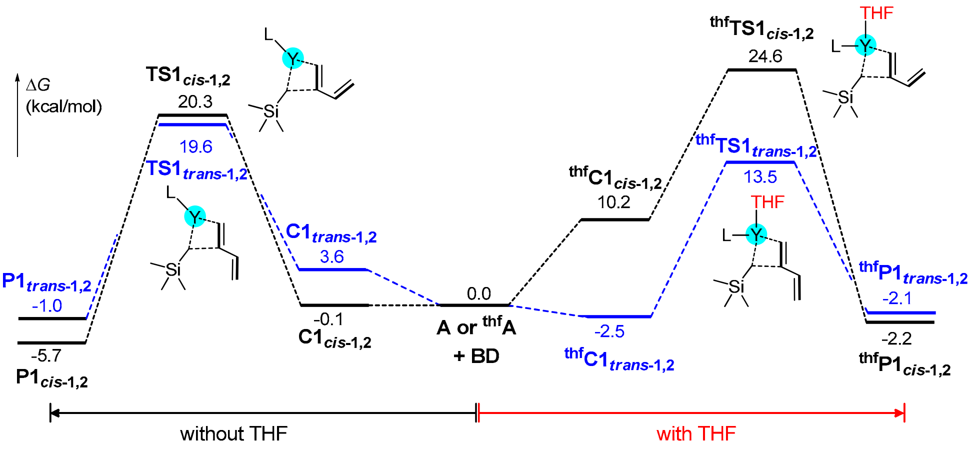

Figure S1. Computed energy profiles (Δ*G* in kcal/mol) for the first butadiene 1,2−insertion into the Y−alkyl σ−bond of the cationic species ^{thf}**A** or **A**.

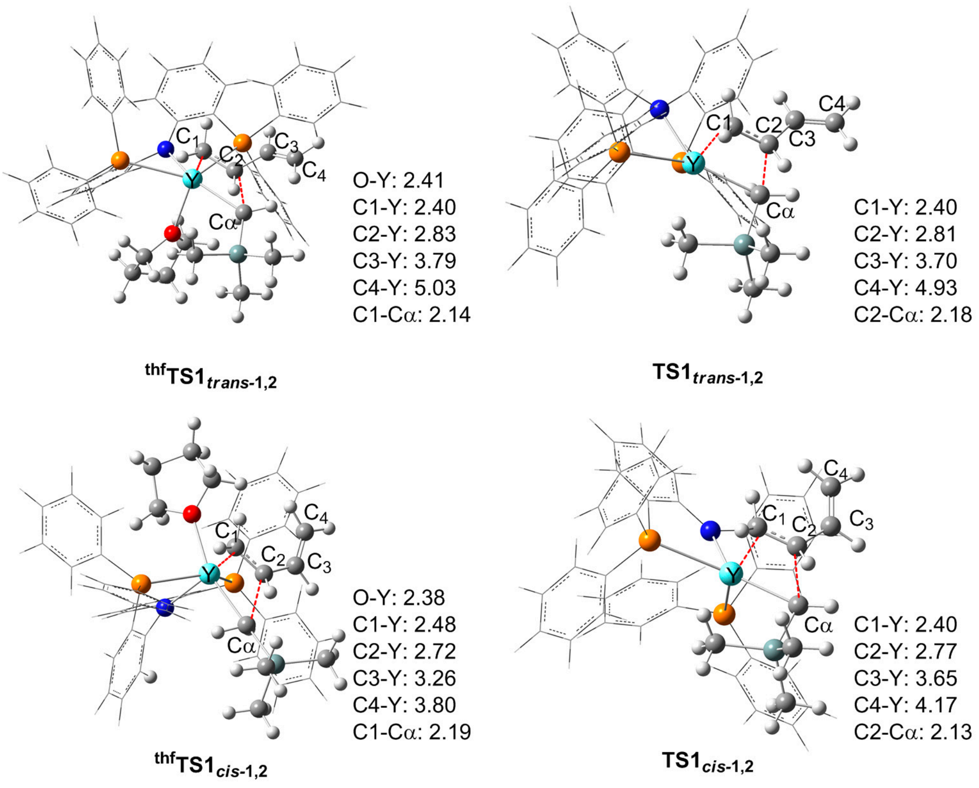

Figure S2. Transition structures (distances in Å) for the 1,2−insertion of butadiene into the Y−alkyl σ−bond of the cationic species ^{thf}**A** or **A**.

Optimized coordinates (Å)

10

cis-butadiene E = −155.8596681 a.u.

C	0.000000000	1.544955000	−0.503232000
C	0.000000000	0.734165000	0.563084000
C	0.000000000	−0.734165000	0.563084000
C	0.000000000	−1.544955000	−0.503232000
H	0.000000000	1.168859000	−1.523654000
H	0.000000000	2.624863000	−0.386752000
H	0.000000000	1.193935000	1.551296000
H	0.000000000	−1.193935000	1.551296000
H	0.000000000	−1.168859000	−1.523654000
H	0.000000000	−2.624863000	−0.386752000

10

trans-butadiene E = −155.8653312 a.u.

C	0.601294000	1.749734000	0.000000000
C	0.601294000	0.409494000	0.000000000
C	−0.601294000	−0.409494000	0.000000000
C	−0.601294000	−1.749734000	0.000000000
H	−0.326353000	2.318814000	0.000000000
H	1.524597000	2.321846000	0.000000000
H	1.551412000	−0.126139000	0.000000000
H	−1.551412000	0.126139000	0.000000000
H	−1.524597000	−2.321846000	0.000000000
H	0.326353000	−2.318814000	0.000000000

13

trans-isoprene E = −195.1536413 a.u.

C	−1.982355000	0.001948000	0.000016000
C	−0.829454000	−0.680686000	0.000053000
H	−2.939726000	−0.511471000	−0.000007000
H	−2.016350000	1.088400000	−0.000084000
C	0.515273000	−0.098486000	−0.000223000
H	−0.865782000	−1.770505000	0.000108000
C	1.590370000	−0.904888000	−0.000022000
H	1.488542000	−1.987825000	0.000186000

H	2.603084000	−0.510145000	0.000229000
C	0.653179000	1.399983000	0.000043000
H	0.171321000	1.841570000	−0.881562000
H	0.172235000	1.841156000	0.882355000
H	1.704590000	1.701593000	−0.000428000

13

THF E = −232.2936148 a.u.

C	−1.175930000	−0.462655000	0.000000000
O	−0.000007000	−1.251995000	0.000000000
C	1.175922000	−0.462664000	0.000000000
C	0.770963000	1.023651000	0.000000000
C	−0.770953000	1.023657000	0.000000000
H	−1.774682000	−0.718572000	−0.885504000
H	−1.774682000	−0.718571000	0.885505000
H	1.774682000	−0.718595000	0.885498000
H	1.774683000	−0.718596000	−0.885497000
H	1.168052000	1.539584000	0.879522000
H	1.168051000	1.539583000	−0.879524000
H	−1.168033000	1.539598000	0.879522000
H	−1.168033000	1.539598000	−0.879523000

97

thf_A E = −1888.854213 a.u.

N	0.117318000	−1.434060000	−0.424017000
O	0.184886000	0.783646000	2.637730000
P	−2.636940000	−0.337081000	−0.229392000
P	2.737061000	−0.042695000	−0.363749000
Si	−0.994608000	4.168611000	−0.667955000
C	0.153675000	2.700862000	−0.913315000
H	1.174329000	3.073828000	−0.697353000
H	0.159111000	2.455879000	−1.991771000
C	−2.688384000	3.864921000	−1.454317000
H	−2.601857000	3.693512000	−2.534762000
H	−3.337373000	4.739096000	−1.319192000
H	−3.207788000	3.004099000	−1.015070000
C	−1.268038000	4.475294000	1.190521000

H	−1.785693000	3.635524000	1.676872000
H	−1.891740000	5.363125000	1.352298000
H	−0.318975000	4.651966000	1.714235000
C	−0.273787000	5.755357000	−1.403302000
H	0.695531000	6.003508000	−0.952943000
H	−0.940241000	6.613229000	−1.248764000
H	−0.115808000	5.649257000	−2.483670000
C	−4.242978000	0.064953000	0.576116000
C	−5.471972000	−0.351896000	0.050112000
H	−5.504248000	−0.931610000	−0.867886000
C	−4.224880000	0.830420000	1.750886000
H	−3.278832000	1.181281000	2.160175000
C	−6.660089000	−0.015973000	0.698297000
H	−7.609305000	−0.340591000	0.280876000
C	−5.412520000	1.156782000	2.400741000
H	−5.387416000	1.751579000	3.309910000
C	−6.632917000	0.733713000	1.873249000
H	−7.561145000	0.995653000	2.373405000
C	−3.028972000	−0.810846000	−1.964118000
C	−2.927260000	0.166375000	−2.962228000
H	−2.612695000	1.174923000	−2.707098000
C	−3.426685000	−2.110748000	−2.302725000
H	−3.496119000	−2.877329000	−1.535357000
C	−3.232148000	−0.151139000	−4.284804000
H	−3.152359000	0.611235000	−5.054949000
C	−3.633863000	−1.444252000	−4.617466000
H	−3.869378000	−1.691408000	−5.649021000
C	−3.730392000	−2.422150000	−3.626930000
H	−4.042644000	−3.430559000	−3.885180000
C	−0.732156000	−2.298108000	0.282705000
C	−2.068852000	−1.903810000	0.564091000
C	−2.886687000	−2.691785000	1.385145000
H	−3.903305000	−2.368473000	1.593304000
C	−1.123486000	−4.313918000	1.598012000
H	−0.751820000	−5.257979000	1.988908000

C	−2.421495000	−3.894971000	1.906183000
H	−3.066646000	−4.507810000	2.528876000
C	−0.291962000	−3.531889000	0.806467000
H	0.719430000	−3.862675000	0.588190000
C	1.012679000	−1.973087000	−1.359859000
C	2.304466000	−1.410822000	−1.507664000
C	1.553002000	−3.519781000	−3.159580000
H	1.249137000	−4.339813000	−3.805375000
C	0.662603000	−3.042397000	−2.204703000
H	−0.327787000	−3.479624000	−2.117535000
C	2.817948000	−2.947177000	−3.310386000
H	3.506508000	−3.317381000	−4.064019000
C	3.185336000	−1.890513000	−2.483147000
H	4.166146000	−1.434336000	−2.588723000
C	4.066080000	0.914513000	−1.206312000
C	3.690274000	1.904367000	−2.125474000
H	2.638920000	2.107241000	−2.310381000
C	4.662584000	2.632419000	−2.807342000
H	4.362275000	3.396606000	−3.519102000
C	5.425858000	0.668644000	−0.975021000
H	5.731685000	−0.095684000	−0.266385000
C	6.015209000	2.386639000	−2.571101000
H	6.772523000	2.959522000	−3.099201000
C	6.394202000	1.405911000	−1.655647000
H	7.446871000	1.209540000	−1.470623000
C	4.025555000	−0.021963000	2.124682000
H	3.921855000	1.059540000	2.051183000
C	3.747812000	−2.228025000	1.174237000
H	3.420581000	−2.870492000	0.361672000
C	3.577061000	−0.842738000	1.077502000
C	4.356479000	−2.783462000	2.300973000
H	4.491914000	−3.860059000	2.362606000
C	4.806120000	−1.962964000	3.334110000
H	5.290939000	−2.397657000	4.203983000
C	4.645084000	−0.578721000	3.241776000

H	5.013061000	0.067390000	4.034898000
C	0.543901000	1.908048000	3.504877000
H	−0.086811000	2.754302000	3.226621000
H	1.594522000	2.153216000	3.315628000
C	0.135859000	−0.459186000	3.427135000
H	0.713053000	−1.216707000	2.892497000
H	−0.912112000	−0.767751000	3.481694000
C	0.325822000	1.391860000	4.916165000
H	0.937777000	1.930329000	5.644842000
H	−0.725295000	1.498207000	5.207212000
C	0.709833000	−0.084625000	4.783964000
H	0.297367000	−0.707383000	5.582284000
H	1.798451000	−0.202666000	4.781870000
Y	−0.003070000	0.709870000	0.311540000

84

A E = −1656.507424 a.u.

N	−0.085096000	−0.748697000	1.280981000
P	−2.597217000	−0.239698000	−0.248167000
P	2.499231000	−0.360547000	−0.175029000
Si	0.230174000	4.069527000	−0.736977000
C	−0.088981000	3.108263000	0.830113000
H	−1.039400000	3.390390000	1.306283000
H	0.707265000	3.260989000	1.573943000
C	0.364047000	2.755672000	−2.191724000
H	1.204864000	2.033556000	−2.159865000
H	0.575288000	3.334122000	−3.099643000
H	−0.572650000	2.219894000	−2.440285000
C	−1.195303000	5.184041000	−1.261081000
H	−2.141653000	4.634387000	−1.334722000
H	−1.006459000	5.652384000	−2.234499000
H	−1.336795000	5.986480000	−0.527366000
C	1.874280000	4.985072000	−0.745840000
H	1.831169000	5.831532000	−0.049897000
H	2.123505000	5.380341000	−1.737707000
H	2.696839000	4.338430000	−0.418226000

C	−4.220022000	0.547465000	−0.604686000
C	−4.336865000	1.935746000	−0.438989000
H	−3.489784000	2.519486000	−0.082692000
C	−5.543240000	2.575551000	−0.709360000
H	−5.628084000	3.650029000	−0.571565000
C	−6.637936000	1.837317000	−1.162212000
H	−7.577203000	2.337706000	−1.380577000
C	−6.524606000	0.459203000	−1.335363000
H	−7.375378000	−0.118140000	−1.686588000
C	−5.321212000	−0.188682000	−1.056027000
H	−5.242671000	−1.263465000	−1.190705000
C	−2.709040000	−1.972635000	−0.843669000
C	−2.425907000	−2.227867000	−2.193005000
H	−2.154444000	−1.411265000	−2.859944000
C	−2.509713000	−3.525927000	−2.691841000
H	−2.295131000	−3.717924000	−3.739560000
C	−2.867450000	−4.575532000	−1.845122000
H	−2.928662000	−5.588573000	−2.232913000
C	−3.147808000	−4.325308000	−0.502083000
H	−3.429852000	−5.142056000	0.156684000
C	−3.070948000	−3.027637000	0.001614000
H	−3.292541000	−2.836062000	1.047769000
C	−2.465033000	−0.297276000	1.593924000
C	−1.184678000	−0.574642000	2.144762000
C	−1.053922000	−0.580910000	3.546863000
H	−0.077750000	−0.782820000	3.978857000
C	−2.143698000	−0.321454000	4.366521000
H	−2.014482000	−0.329528000	5.445621000
C	−3.396987000	−0.032525000	3.816315000
H	−4.246101000	0.172647000	4.461572000
C	−3.550628000	−0.011081000	2.435758000
H	−4.521132000	0.212751000	2.002018000
C	0.764853000	−1.846394000	1.427315000
C	0.412057000	−2.999784000	2.156284000
H	−0.554094000	−3.041057000	2.649092000

C	1.269531000	−4.090308000	2.237258000
H	0.958137000	−4.964228000	2.804039000
C	2.508259000	−4.084789000	1.592990000
H	3.171563000	−4.941908000	1.655223000
C	2.874815000	−2.961299000	0.860889000
H	3.829083000	−2.943208000	0.340395000
C	2.028807000	−1.849739000	0.780107000
C	3.424280000	−0.946854000	−1.653471000
C	2.689600000	−1.317888000	−2.788579000
H	1.602755000	−1.251320000	−2.784597000
C	3.341303000	−1.793099000	−3.923729000
H	2.764266000	−2.081690000	−4.798058000
C	4.732903000	−1.892671000	−3.937895000
H	5.242235000	−2.257843000	−4.825323000
C	5.469136000	−1.521756000	−2.813718000
H	6.552867000	−1.599651000	−2.821631000
C	4.820653000	−1.050674000	−1.672518000
H	5.402060000	−0.760816000	−0.801855000
C	3.714538000	0.571193000	0.852186000
C	4.357869000	1.687913000	0.294945000
H	4.198730000	1.947957000	−0.749731000
C	5.229180000	2.451710000	1.067425000
H	5.735810000	3.305183000	0.625004000
C	5.452353000	2.119394000	2.404975000
H	6.130215000	2.717723000	3.007344000
C	4.809567000	1.016440000	2.963936000
H	4.986798000	0.750570000	4.002501000
C	3.943326000	0.240988000	2.192607000
H	3.454370000	−0.625125000	2.629065000
Y	−0.039097000	0.987073000	−0.118819000

107

thf_C1_cis E = −2044.723648 a.u.

N	−0.023386000	−0.260968000	1.311519000
P	2.733469000	−0.256557000	−0.013425000
P	−2.748692000	−0.344738000	−0.098278000

Si	−0.072884000	4.578438000	−0.438365000
C	−0.032921000	2.636722000	−0.680060000
H	−0.780358000	2.191001000	0.006465000
H	1.001987000	2.280529000	−0.496995000
C	−1.867530000	5.215091000	−0.785778000
H	−2.108199000	5.195452000	−1.862021000
H	−1.957541000	6.259487000	−0.440605000
H	−2.611496000	4.609690000	−0.240311000
C	0.407976000	4.931111000	1.399879000
H	−0.288946000	4.420758000	2.085465000
H	0.368822000	6.015514000	1.600895000
H	1.429433000	4.571747000	1.608794000
C	1.201714000	5.388795000	−1.644993000
H	2.217646000	5.013000000	−1.436896000
H	1.202871000	6.483563000	−1.506189000
H	0.953319000	5.177295000	−2.698852000
C	4.159036000	−1.305943000	−0.699362000
C	3.855190000	−2.352279000	−1.593364000
H	2.820446000	−2.523094000	−1.895886000
C	4.884347000	−3.165964000	−2.095575000
H	4.650868000	−3.973479000	−2.793029000
C	6.216302000	−2.934457000	−1.710491000
H	7.015630000	−3.563702000	−2.105696000
C	6.518941000	−1.887903000	−0.823338000
H	7.553357000	−1.703987000	−0.526887000
C	5.494021000	−1.070379000	−0.314737000
H	5.733786000	−0.248969000	0.363022000
C	3.623773000	1.344033000	0.497989000
C	4.220393000	2.124436000	−0.516351000
H	4.199075000	1.787918000	−1.556415000
C	4.873150000	3.321591000	−0.178684000
H	5.350145000	3.917918000	−0.959187000
C	4.923503000	3.743690000	1.162718000
H	5.434489000	4.672397000	1.422654000
C	4.325517000	2.965212000	2.167943000

H	4.374580000	3.286199000	3.210437000
C	3.674078000	1.761493000	1.840273000
H	3.222626000	1.145035000	2.620225000
C	2.304331000	−1.094669000	1.627366000
C	0.960479000	−0.952189000	2.086616000
C	0.632296000	−1.604280000	3.309558000
H	−0.387141000	−1.523241000	3.689646000
C	1.582969000	−2.338871000	4.025695000
H	1.289372000	−2.828077000	4.957289000
C	2.903400000	−2.466197000	3.553209000
H	3.642253000	−3.041743000	4.112604000
C	3.255019000	−1.840998000	2.347771000
H	4.271091000	−1.934208000	1.957259000
C	−1.040175000	0.429049000	2.046608000
C	−0.753439000	1.121925000	3.256594000
H	0.262214000	1.083066000	3.653059000
C	−1.739868000	1.844672000	3.935245000
H	−1.481564000	2.363543000	4.861095000
C	−3.052214000	1.921877000	3.429927000
H	−3.820463000	2.485465000	3.961308000
C	−3.360188000	1.265904000	2.229423000
H	−4.370103000	1.320903000	1.817488000
C	−2.374180000	0.528258000	1.546910000
C	−4.361873000	0.489643000	−0.654171000
C	−5.622311000	−0.034280000	−0.304472000
H	−5.693393000	−0.955872000	0.275382000
C	−6.790583000	0.634900000	−0.710590000
H	−7.766662000	0.227763000	−0.439940000
C	−6.704842000	1.822808000	−1.455866000
H	−7.614569000	2.338521000	−1.768171000
C	−5.446664000	2.346250000	−1.800055000
H	−5.376897000	3.269340000	−2.379065000
C	−4.275071000	1.680540000	−1.403243000
H	−3.299777000	2.089934000	−1.669886000
C	−3.345567000	−2.072181000	0.433575000

C	−3.287267000	−2.483293000	1.777632000
H	−2.922547000	−1.791893000	2.539804000
C	−3.721459000	−3.774369000	2.130728000
H	−3.683952000	−4.090673000	3.175109000
C	−4.220495000	−4.646669000	1.148724000
H	−4.569350000	−5.642383000	1.428167000
C	−4.285567000	−4.230802000	−0.194195000
H	−4.694530000	−4.899137000	−0.955135000
C	−3.843326000	−2.946536000	−0.556860000
H	−3.914479000	−2.615318000	−1.596615000
C	1.434165000	0.239065000	−3.227608000
C	0.089748000	0.153715000	−3.628053000
H	2.178913000	−0.459270000	−3.616003000
H	1.837739000	1.196941000	−2.870664000
H	−0.227336000	−0.714597000	−4.219998000
C	−0.935806000	0.990794000	−3.114402000
C	−0.597817000	2.352433000	−2.598516000
H	−1.458771000	3.029230000	−2.588383000
H	0.273519000	2.816889000	−3.082677000
Y	0.024475000	−0.072966000	−0.999133000
H	−1.970143000	0.813023000	−3.419697000
O	0.069829000	−2.355564000	−1.301465000
C	−0.084498000	−3.150024000	−2.598721000
C	0.127727000	−3.365139000	−0.134714000
C	0.273554000	−4.593302000	−2.216082000
H	−1.134173000	−3.057478000	−2.915035000
H	0.594889000	−2.698230000	−3.331655000
C	−0.268795000	−4.707452000	−0.765203000
H	1.162541000	−3.353253000	0.233364000
H	−0.571416000	−3.002169000	0.626318000
H	1.366113000	−4.736421000	−2.229691000
H	−0.198423000	−5.316410000	−2.898231000
H	0.182784000	−5.549479000	−0.219089000
H	−1.364050000	−4.815736000	−0.770404000

107

thf_TS1_cis E = −2044.728872 a.u. Imaginary frequency=157i

N	0.004950000	−0.421676000	1.398949000
P	2.745144000	−0.270267000	0.142463000
P	−2.737660000	−0.198690000	0.123585000
Si	0.022196000	4.381754000	−0.808745000
C	−0.086468000	2.509667000	−0.580142000
H	−0.842486000	2.316131000	0.206550000
H	0.898228000	2.248876000	−0.121286000
C	−1.619619000	5.115022000	−1.394830000
H	−1.951091000	4.721972000	−2.362886000
H	−1.516252000	6.201484000	−1.506534000
H	−2.417995000	4.944073000	−0.662376000
C	0.473443000	5.176308000	0.844058000
H	−0.279929000	4.959253000	1.611005000
H	0.548321000	6.267256000	0.751514000
H	1.437522000	4.806521000	1.213004000
C	1.380632000	4.806089000	−2.057894000
H	2.349015000	4.398961000	−1.741711000
H	1.495436000	5.895227000	−2.123412000
H	1.179746000	4.448566000	−3.075317000
C	4.109930000	−1.142914000	−0.739629000
C	3.784757000	−1.992383000	−1.806484000
H	2.747190000	−2.100083000	−2.116001000
C	4.782239000	−2.693709000	−2.480898000
H	4.520501000	−3.349604000	−3.307221000
C	6.116775000	−2.543556000	−2.103621000
H	6.896121000	−3.084003000	−2.633702000
C	6.449346000	−1.692741000	−1.050350000
H	7.488150000	−1.570202000	−0.756041000
C	5.452847000	−0.994419000	−0.369055000
H	5.722220000	−0.330087000	0.447362000
C	3.563432000	1.197485000	0.907138000
C	4.070924000	2.196136000	0.061983000
H	4.008897000	2.082510000	−1.018375000
C	4.679678000	3.327452000	0.599652000

H	5.081431000	4.090511000	−0.061632000
C	4.774889000	3.478541000	1.984544000
H	5.247714000	4.362550000	2.403417000
C	4.268023000	2.491280000	2.827333000
H	4.346821000	2.601862000	3.905525000
C	3.665699000	1.351354000	2.293378000
H	3.286563000	0.577418000	2.954328000
C	2.271941000	−1.368977000	1.531781000
C	0.940686000	−1.263174000	2.016108000
C	0.593043000	−2.099055000	3.101957000
H	−0.414435000	−2.043163000	3.502724000
C	1.501186000	−2.988918000	3.658739000
H	1.185870000	−3.617999000	4.487745000
C	2.802198000	−3.094065000	3.158684000
H	3.510264000	−3.794169000	3.591695000
C	3.174821000	−2.280998000	2.095578000
H	4.182628000	−2.347732000	1.694165000
C	−0.944484000	0.191692000	2.233970000
C	−0.594596000	0.698660000	3.504044000
H	0.430537000	0.587623000	3.844257000
C	−1.523313000	1.337676000	4.313538000
H	−1.210309000	1.719096000	5.282351000
C	−2.843838000	1.506944000	3.889132000
H	−3.571094000	2.006677000	4.522005000
C	−3.211493000	1.033373000	2.635894000
H	−4.231493000	1.169481000	2.287338000
C	−2.285471000	0.380283000	1.810049000
C	−4.241856000	0.787347000	−0.306894000
C	−5.541882000	0.273280000	−0.226067000
H	−5.709088000	−0.748565000	0.099793000
C	−6.635969000	1.073425000	−0.557487000
H	−7.639407000	0.661616000	−0.490316000
C	−6.447247000	2.392628000	−0.963684000
H	−7.301823000	3.012351000	−1.220727000
C	−5.156053000	2.916479000	−1.032866000

H	−4.999331000	3.947067000	−1.339834000
C	−4.061444000	2.118492000	−0.710302000
H	−3.061957000	2.542488000	−0.751446000
C	−3.373754000	−1.922883000	0.331938000
C	−3.257126000	−2.609395000	1.544731000
H	−2.827672000	−2.108822000	2.407442000
C	−3.713388000	−3.923850000	1.656365000
H	−3.623793000	−4.444560000	2.606107000
C	−4.300101000	−4.560376000	0.563678000
H	−4.667500000	−5.578647000	0.657758000
C	−4.428612000	−3.878880000	−0.648299000
H	−4.902181000	−4.362037000	−1.499117000
C	−3.959586000	−2.571806000	−0.766614000
H	−4.085274000	−2.041676000	−1.708953000
C	1.783270000	0.794760000	−3.182251000
C	0.645081000	0.185391000	−3.614387000
H	2.757936000	0.367114000	−3.392613000
H	1.771967000	1.779120000	−2.717602000
H	0.750218000	−0.766915000	−4.131178000
C	−0.696865000	0.647766000	−3.327659000
C	−0.931041000	1.932297000	−2.873991000
H	−1.942324000	2.252928000	−2.651625000
H	−0.217473000	2.717801000	−3.081549000
Y	0.052655000	0.117161000	−0.875339000
H	−1.535688000	−0.029621000	−3.477335000
O	−0.164840000	−2.239741000	−1.566573000
C	−0.831090000	−2.846373000	−2.707621000
C	0.160477000	−3.292826000	−0.592337000
C	−0.452161000	−4.315037000	−2.657688000
H	−1.910633000	−2.707811000	−2.589147000
H	−0.494570000	−2.333559000	−3.609296000
C	−0.433979000	−4.578075000	−1.151577000
H	1.250637000	−3.337171000	−0.516119000
H	−0.258552000	−3.002127000	0.371289000
H	0.539897000	−4.472803000	−3.096731000

H	−1.168362000	−4.941494000	−3.196821000
H	0.162936000	−5.451522000	−0.874622000
H	−1.451820000	−4.724496000	−0.775837000

107

thf_P1_cis E = −2044.787 a.u.

N	−0.016242000	−0.228756000	1.314775000
P	2.713763000	−0.260977000	0.048580000
P	−2.740822000	−0.296544000	0.002149000
Si	0.002766000	4.540410000	−0.487600000
C	−0.119064000	2.696893000	−1.043850000
H	−0.839085000	2.250295000	−0.332314000
H	0.887089000	2.279720000	−0.843515000
C	−1.719766000	5.296687000	−0.622479000
H	−2.094712000	5.307139000	−1.652705000
H	−1.702227000	6.337627000	−0.276850000
H	−2.445573000	4.759061000	−0.000406000
C	0.597075000	4.579525000	1.294963000
H	−0.073956000	4.016527000	1.954065000
H	0.636436000	5.611898000	1.663809000
H	1.602124000	4.153724000	1.394207000
C	1.211198000	5.438283000	−1.620044000
H	2.213781000	4.998440000	−1.562034000
H	1.297627000	6.491050000	−1.324300000
H	0.892079000	5.420512000	−2.668558000
C	4.096645000	−1.246167000	−0.672680000
C	3.786996000	−2.256703000	−1.592397000
H	2.750958000	−2.421859000	−1.877468000
C	4.795377000	−3.040511000	−2.149151000
H	4.545785000	−3.819406000	−2.865196000
C	6.126622000	−2.814836000	−1.799426000
H	6.915002000	−3.419718000	−2.238838000
C	6.444239000	−1.805586000	−0.891456000
H	7.480520000	−1.623590000	−0.619727000
C	5.436506000	−1.022909000	−0.329084000
H	5.695795000	−0.234049000	0.371367000

C	3.531573000	1.315617000	0.558819000
C	4.012646000	2.171939000	−0.443944000
H	3.915284000	1.896940000	−1.492370000
C	4.649605000	3.363320000	−0.103931000
H	5.034405000	4.012027000	−0.886402000
C	4.800769000	3.717580000	1.238220000
H	5.298579000	4.646456000	1.502651000
C	4.317398000	2.874387000	2.237218000
H	4.438570000	3.143103000	3.283239000
C	3.685346000	1.675867000	1.901622000
H	3.324468000	1.014890000	2.684514000
C	2.261216000	−1.115992000	1.609140000
C	0.924271000	−0.958315000	2.061119000
C	0.569015000	−1.634333000	3.249373000
H	−0.447075000	−1.541398000	3.620912000
C	1.481169000	−2.419123000	3.942502000
H	1.161088000	−2.927700000	4.848759000
C	2.791355000	−2.572148000	3.481877000
H	3.502836000	−3.189741000	4.021756000
C	3.168067000	−1.918651000	2.314259000
H	4.181526000	−2.030781000	1.937668000
C	−0.993390000	0.475538000	2.039960000
C	−0.680489000	1.166695000	3.230463000
H	0.337119000	1.123090000	3.607199000
C	−1.636917000	1.901708000	3.918362000
H	−1.351742000	2.423284000	4.828743000
C	−2.949193000	1.987518000	3.445595000
H	−3.696832000	2.563401000	3.982797000
C	−3.283317000	1.323514000	2.271469000
H	−4.298733000	1.381905000	1.888893000
C	−2.330515000	0.569649000	1.571800000
C	−4.289028000	0.515154000	−0.593193000
C	−5.560108000	−0.021460000	−0.351031000
H	−5.666599000	−0.953363000	0.195800000
C	−6.699685000	0.639037000	−0.810256000

H	−7.680381000	0.212468000	−0.617423000
C	−6.584104000	1.840031000	−1.507591000
H	−7.473950000	2.351345000	−1.864271000
C	−5.321909000	2.384142000	−1.745760000
H	−5.224544000	3.321424000	−2.287029000
C	−4.181551000	1.723221000	−1.295972000
H	−3.203045000	2.154028000	−1.487092000
C	−3.297288000	−1.991515000	0.490438000
C	−3.338290000	−2.411995000	1.823997000
H	−3.055752000	−1.723626000	2.615472000
C	−3.760172000	−3.704560000	2.139939000
H	−3.794235000	−4.019823000	3.179444000
C	−4.153898000	−4.581709000	1.130628000
H	−4.494415000	−5.582864000	1.380463000
C	−4.121068000	−4.166536000	−0.202477000
H	−4.443606000	−4.840808000	−0.991894000
C	−3.684921000	−2.882357000	−0.522583000
H	−3.676289000	−2.561219000	−1.562728000
C	1.310804000	0.211865000	−3.242623000
C	−0.046221000	0.266065000	−3.580042000
H	1.964084000	−0.517972000	−3.712782000
H	1.817964000	1.121702000	−2.913412000
H	−0.457572000	−0.578721000	−4.139598000
C	−0.975978000	1.147256000	−3.019175000
C	−0.569312000	2.532087000	−2.517777000
H	−1.423856000	3.199445000	−2.680326000
H	0.236928000	2.919601000	−3.156977000
Y	0.007237000	−0.170297000	−1.013563000
H	−2.011044000	1.035543000	−3.332680000
O	0.044287000	−2.513703000	−1.299901000
C	−0.119166000	−3.232199000	−2.559566000
C	0.154206000	−3.476105000	−0.191965000
C	0.282092000	−4.665130000	−2.253514000
H	−1.171028000	−3.157253000	−2.857089000
H	0.509027000	−2.741329000	−3.305947000

C	−0.179685000	−4.827297000	−0.803512000
H	1.181157000	−3.424059000	0.179930000
H	−0.535295000	−3.158561000	0.590654000
H	1.368412000	−4.785763000	−2.330901000
H	−0.189544000	−5.375200000	−2.938540000
H	0.329840000	−5.642093000	−0.281791000
H	−1.257980000	−5.010838000	−0.758657000

107

thf_C1_trans E = −2044.73424 a.u.

N	0.035742000	−1.077911000	−1.292865000
P	−2.733015000	−0.313176000	−0.492128000
P	2.735172000	−0.392140000	−0.148769000
Si	0.811575000	4.364849000	−0.246860000
C	0.460530000	2.562304000	−0.663667000
H	1.274163000	2.232651000	−1.340729000
H	−0.433783000	2.574337000	−1.327620000
C	1.543999000	4.591920000	1.492439000
H	0.833359000	4.288065000	2.271313000
H	1.785179000	5.647060000	1.672738000
H	2.466075000	4.017847000	1.646093000
C	2.033495000	5.106926000	−1.487538000
H	2.984153000	4.559252000	−1.501788000
H	2.258277000	6.155720000	−1.256671000
H	1.623619000	5.070657000	−2.504434000
C	−0.784671000	5.384364000	−0.323498000
H	−1.245984000	5.316383000	−1.316598000
H	−0.569341000	6.443687000	−0.136145000
H	−1.533822000	5.075973000	0.416250000
C	−4.244691000	−0.546694000	0.539287000
C	−4.087518000	−0.862102000	1.897287000
H	−3.089649000	−0.951948000	2.322508000
C	−5.200566000	−1.061495000	2.710598000
H	−5.067751000	−1.314456000	3.759407000
C	−6.484650000	−0.931432000	2.179668000
H	−7.353662000	−1.080123000	2.814535000

C	−6.648929000	−0.607055000	0.834350000
H	−7.646653000	−0.503274000	0.416683000
C	−5.536231000	−0.416739000	0.014478000
H	−5.675889000	−0.166784000	−1.033243000
C	−3.333517000	0.488481000	−2.041368000
C	−3.558957000	1.872550000	−2.028339000
H	−3.387733000	2.448049000	−1.121148000
C	−4.014717000	2.520327000	−3.174162000
H	−4.191178000	3.592296000	−3.154316000
C	−4.237554000	1.793549000	−4.344198000
H	−4.587481000	2.299784000	−5.239639000
C	−4.011466000	0.417864000	−4.363206000
H	−4.188501000	−0.150929000	−5.272121000
C	−3.562049000	−0.236711000	−3.216372000
H	−3.392989000	−1.309676000	−3.234613000
C	−2.212288000	−2.002914000	−0.979740000
C	−0.861683000	−2.152300000	−1.388976000
C	−0.463994000	−3.427347000	−1.840835000
H	0.558163000	−3.570181000	−2.176721000
C	−1.349205000	−4.498170000	−1.854463000
H	−1.002226000	−5.467950000	−2.202709000
C	−2.667332000	−4.345900000	−1.417505000
H	−3.355308000	−5.185991000	−1.425431000
C	−3.090303000	−3.093683000	−0.984321000
H	−4.117888000	−2.951874000	−0.659436000
C	1.030765000	−0.961972000	−2.286252000
C	0.727709000	−1.127687000	−3.651002000
H	−0.289625000	−1.383181000	−3.932849000
C	1.697142000	−0.949566000	−4.629435000
H	1.428768000	−1.076383000	−5.675171000
C	3.000920000	−0.589134000	−4.281341000
H	3.756271000	−0.439225000	−5.047072000
C	3.322702000	−0.419528000	−2.939379000
H	4.333193000	−0.135105000	−2.660169000
C	2.361222000	−0.617436000	−1.940075000

C	4.299250000	0.580690000	−0.084770000
C	5.558978000	−0.018768000	−0.226238000
H	5.641148000	−1.092965000	−0.367125000
C	6.713043000	0.761326000	−0.179365000
H	7.685570000	0.289262000	−0.290339000
C	6.621998000	2.140448000	0.009985000
H	7.524353000	2.744544000	0.048335000
C	5.372388000	2.741646000	0.154540000
H	5.295918000	3.814775000	0.307085000
C	4.216156000	1.964445000	0.111417000
H	3.243833000	2.433788000	0.228723000
C	3.263390000	−2.059661000	0.456929000
C	3.190516000	−3.208032000	−0.337715000
H	2.846499000	−3.132330000	−1.364811000
C	3.585796000	−4.445093000	0.174569000
H	3.530628000	−5.329439000	−0.454735000
C	4.066823000	−4.544764000	1.478375000
H	4.384718000	−5.506750000	1.870949000
C	4.154715000	−3.400415000	2.273964000
H	4.550555000	−3.467541000	3.284236000
C	3.748057000	−2.166918000	1.769820000
H	3.842355000	−1.277395000	2.389907000
C	−1.823567000	2.195678000	5.663027000
C	−2.261947000	1.974102000	4.414110000
H	−2.341545000	1.795319000	6.529447000
H	−0.945408000	2.808015000	5.859269000
H	−3.159641000	1.377852000	4.250882000
C	−1.619948000	2.526866000	3.232020000
C	−2.079117000	2.374366000	1.973159000
H	−1.612305000	2.901611000	1.142757000
H	−2.997843000	1.826862000	1.774999000
Y	−0.047153000	0.506975000	0.323330000
H	−0.731752000	3.136512000	3.404123000
O	0.087849000	−0.720415000	2.368135000
C	0.634492000	−0.341060000	3.662264000

C	−0.113396000	−2.175274000	2.324488000
C	0.255454000	−1.483338000	4.586554000
H	1.721326000	−0.238448000	3.560726000
H	0.193214000	0.618549000	3.935614000
C	0.391271000	−2.698188000	3.664483000
H	−1.183894000	−2.347218000	2.181080000
H	0.435548000	−2.570020000	1.466488000
H	−0.776919000	−1.362040000	4.933149000
H	0.906781000	−1.537244000	5.463337000
H	−0.187871000	−3.561065000	4.004591000
H	1.439428000	−3.001630000	3.580706000

107

thf_TS1_trans E = −2044.717075 a.u. Imaginary frequency=233i

N	0.027999000	−0.348544000	1.438145000
P	2.755412000	−0.209906000	0.164373000
P	−2.705030000	−0.282116000	0.138886000
Si	−0.360486000	4.381253000	−0.811266000
C	−0.172951000	2.493427000	−0.725301000
H	−0.931277000	2.183419000	0.028285000
H	0.824593000	2.345729000	−0.250679000
C	−2.037738000	4.863347000	−1.533248000
H	−2.152633000	4.569059000	−2.583308000
H	−2.154595000	5.953508000	−1.494306000
H	−2.866257000	4.427797000	−0.962482000
C	−0.234218000	5.055333000	0.946393000
H	−1.007960000	4.630459000	1.596825000
H	−0.352207000	6.146222000	0.957173000
H	0.739705000	4.821999000	1.392960000
C	1.021852000	5.139444000	−1.854006000
H	2.008762000	4.787289000	−1.530196000
H	1.012941000	6.230158000	−1.736850000
H	0.920791000	4.934830000	−2.926162000
C	4.210994000	−1.025527000	−0.617997000
C	4.003094000	−1.894505000	−1.697280000
H	3.001864000	−2.040525000	−2.095099000

C	5.080695000	−2.553114000	−2.287199000
H	4.910889000	−3.223518000	−3.125682000
C	6.374408000	−2.341554000	−1.811879000
H	7.214859000	−2.850074000	−2.276227000
C	6.589451000	−1.468294000	−0.745873000
H	7.596831000	−1.295658000	−0.376908000
C	5.514736000	−0.811222000	−0.149225000
H	5.692883000	−0.128215000	0.676741000
C	3.444262000	1.347795000	0.880850000
C	3.879897000	2.346404000	−0.003643000
H	3.834350000	2.183840000	−1.079018000
C	4.397263000	3.542294000	0.487849000
H	4.745570000	4.304986000	−0.203496000
C	4.471154000	3.757741000	1.865478000
H	4.872290000	4.692043000	2.248490000
C	4.036663000	2.770054000	2.747279000
H	4.100955000	2.930908000	3.820122000
C	3.526768000	1.565806000	2.259710000
H	3.205251000	0.792368000	2.951174000
C	2.301481000	−1.258350000	1.603234000
C	0.974241000	−1.144365000	2.097410000
C	0.649033000	−1.916258000	3.234363000
H	−0.351287000	−1.842208000	3.650043000
C	1.573179000	−2.767765000	3.825988000
H	1.276714000	−3.349957000	4.695148000
C	2.865413000	−2.891652000	3.310664000
H	3.585512000	−3.562292000	3.769660000
C	3.218209000	−2.132628000	2.200540000
H	4.222049000	−2.211449000	1.792019000
C	−0.933788000	0.306189000	2.225612000
C	−0.600432000	0.904624000	3.459298000
H	0.420118000	0.818053000	3.819999000
C	−1.537335000	1.608972000	4.201998000
H	−1.237775000	2.062575000	5.143486000
C	−2.848816000	1.754903000	3.741852000

H	−3.580994000	2.310916000	4.319729000
C	−3.203699000	1.180970000	2.527522000
H	−4.218181000	1.296161000	2.156837000
C	−2.273050000	0.452564000	1.773386000
C	−4.236922000	0.576822000	−0.419836000
C	−5.505094000	0.229718000	0.069282000
H	−5.609750000	−0.575339000	0.791380000
C	−6.637670000	0.908417000	−0.376125000
H	−7.615761000	0.632485000	0.008557000
C	−6.517959000	1.935993000	−1.312140000
H	−7.403580000	2.460601000	−1.659943000
C	−5.261436000	2.285635000	−1.804321000
H	−5.163423000	3.083293000	−2.535517000
C	−4.126923000	1.606086000	−1.363073000
H	−3.150544000	1.882943000	−1.751881000
C	−3.285102000	−2.003939000	0.483766000
C	−3.001656000	−2.647463000	1.692555000
H	−2.476783000	−2.113725000	2.478798000
C	−3.415607000	−3.963727000	1.902313000
H	−3.198185000	−4.450537000	2.849354000
C	−4.120762000	−4.645317000	0.911592000
H	−4.452826000	−5.665770000	1.082095000
C	−4.413815000	−4.005975000	−0.294739000
H	−4.979633000	−4.524822000	−1.064182000
C	−3.993899000	−2.695201000	−0.511109000
H	−4.251401000	−2.198516000	−1.444288000
C	1.668787000	−0.897364000	−4.442918000
C	1.330884000	0.189415000	−3.718790000
H	2.707530000	−1.116541000	−4.667726000
H	0.924820000	−1.552007000	−4.890929000
H	2.148180000	0.831844000	−3.376831000
C	−0.005138000	0.653486000	−3.372748000
C	−0.217589000	1.960941000	−2.915888000
H	−1.206036000	2.396668000	−3.007385000
H	0.596005000	2.674247000	−2.995284000

Y	0.036591000	0.100164000	−0.850366000
H	−0.866784000	0.083254000	−3.724362000
O	−0.168129000	−2.209004000	−1.474399000
C	−0.901445000	−2.766189000	−2.602445000
C	0.301142000	−3.298191000	−0.611416000
C	−0.560606000	−4.246673000	−2.612461000
H	−1.968453000	−2.596825000	−2.424716000
H	−0.584155000	−2.233742000	−3.498669000
C	−0.399920000	−4.546721000	−1.121146000
H	1.388270000	−3.361655000	−0.723780000
H	0.055695000	−3.040910000	0.419492000
H	0.379595000	−4.420313000	−3.148351000
H	−1.342878000	−4.843003000	−3.090302000
H	0.187259000	−5.448232000	−0.924684000
H	−1.378107000	−4.659876000	−0.642599000

107

thf_P1_trans E = −2044.778141 a.u.

N	0.285605000	−0.290903000	1.268977000
P	2.868314000	0.392322000	0.018124000
P	−2.209206000	−1.438555000	−0.019849000
Si	−2.906767000	4.883738000	−0.345985000
C	−1.649157000	3.536176000	−0.838212000
H	−1.334112000	3.009241000	0.076289000
H	−0.757705000	4.064305000	−1.206773000
C	−4.539694000	4.087480000	0.168992000
H	−5.025092000	3.573960000	−0.669868000
H	−5.243476000	4.842275000	0.540273000
H	−4.387915000	3.352261000	0.968302000
C	−2.177543000	5.851575000	1.101800000
H	−2.009905000	5.204370000	1.971209000
H	−2.848942000	6.659920000	1.415584000
H	−1.215987000	6.307732000	0.836389000
C	−3.190662000	6.041956000	−1.810363000
H	−2.254606000	6.509007000	−2.140207000
H	−3.882446000	6.849160000	−1.540533000

H	−3.622276000	5.518752000	−2.672097000
C	4.521468000	−0.048785000	−0.660763000
C	4.596904000	−1.003654000	−1.682940000
H	3.683698000	−1.445022000	−2.075015000
C	5.831577000	−1.375125000	−2.212652000
H	5.881389000	−2.113323000	−3.008900000
C	7.000833000	−0.787517000	−1.731430000
H	7.963357000	−1.071549000	−2.147837000
C	6.932953000	0.175438000	−0.724268000
H	7.841996000	0.642385000	−0.355030000
C	5.700229000	0.547331000	−0.191185000
H	5.654129000	1.309964000	0.581625000
C	3.025215000	2.184769000	0.447597000
C	3.225868000	3.103577000	−0.594220000
H	3.329610000	2.752559000	−1.618435000
C	3.324204000	4.465645000	−0.320969000
H	3.492205000	5.168083000	−1.133094000
C	3.211955000	4.925154000	0.992319000
H	3.288387000	5.987925000	1.205086000
C	3.011380000	4.017136000	2.030349000
H	2.935436000	4.369776000	3.055544000
C	2.918672000	2.650817000	1.762462000
H	2.780758000	1.946957000	2.578149000
C	2.700569000	−0.466537000	1.630134000
C	1.369676000	−0.644761000	2.088918000
C	1.199088000	−1.237659000	3.355635000
H	0.195069000	−1.372744000	3.745213000
C	2.288929000	−1.661568000	4.107586000
H	2.115441000	−2.124932000	5.075760000
C	3.591989000	−1.512892000	3.629900000
H	4.440527000	−1.855030000	4.214677000
C	3.787181000	−0.908951000	2.391390000
H	4.796057000	−0.775270000	2.009776000
C	−0.900888000	0.155049000	1.883224000
C	−0.875635000	1.068755000	2.957485000

H	0.087424000	1.394864000	3.338994000
C	−2.046196000	1.558420000	3.519365000
H	−1.987564000	2.262622000	4.345477000
C	−3.291708000	1.166347000	3.021084000
H	−4.208604000	1.551399000	3.457446000
C	−3.345124000	0.272053000	1.958430000
H	−4.309662000	−0.035547000	1.565384000
C	−2.172760000	−0.251729000	1.395137000
C	−3.942432000	−1.442153000	−0.649906000
C	−4.966974000	−2.114661000	0.033131000
H	−4.748145000	−2.652113000	0.951890000
C	−6.266356000	−2.106160000	−0.468784000
H	−7.053818000	−2.628478000	0.067755000
C	−6.555485000	−1.434529000	−1.657340000
H	−7.569418000	−1.432883000	−2.047835000
C	−5.541087000	−0.772884000	−2.347032000
H	−5.759707000	−0.255857000	−3.277458000
C	−4.239374000	−0.779590000	−1.847300000
H	−3.452833000	−0.268377000	−2.394418000
C	−2.057880000	−3.119935000	0.739800000
C	−1.835940000	−3.313600000	2.106191000
H	−1.772831000	−2.456542000	2.769577000
C	−1.718056000	−4.605243000	2.622463000
H	−1.551014000	−4.745061000	3.687137000
C	−1.829107000	−5.710695000	1.781746000
H	−1.746611000	−6.715131000	2.187768000
C	−2.061333000	−5.524583000	0.417381000
H	−2.169748000	−6.383866000	−0.239526000
C	−2.167816000	−4.236821000	−0.103082000
H	−2.367535000	−4.101219000	−1.164444000
C	1.155847000	0.933031000	−3.156625000
C	0.210203000	1.833669000	−2.655105000
H	2.181996000	1.245410000	−3.321178000
H	0.813645000	0.124404000	−3.811989000
H	0.599534000	2.759048000	−2.222761000

C	−1.125003000	1.514307000	−2.373454000
C	−2.148611000	2.524377000	−1.877921000
H	−3.004093000	1.980535000	−1.457735000
H	−2.549202000	3.066444000	−2.750046000
Y	0.244650000	−0.182372000	−1.007887000
H	−1.546582000	0.709228000	−2.995892000
O	0.966830000	−2.309592000	−1.737879000
C	0.876596000	−2.915317000	−3.061047000
C	1.467513000	−3.294954000	−0.768699000
C	1.685513000	−4.198689000	−2.963780000
H	−0.181033000	−3.110702000	−3.273125000
H	1.267291000	−2.195119000	−3.783558000
C	1.453073000	−4.621642000	−1.511187000
H	2.477322000	−2.986176000	−0.481840000
H	0.811825000	−3.268466000	0.104401000
H	2.747303000	−3.997973000	−3.144903000
H	1.352323000	−4.946762000	−3.688352000
H	2.221866000	−5.302734000	−1.136215000
H	0.479482000	−5.109543000	−1.398753000

107

thf_TS1_anti_syn_isomerism E = −2044.741835 a.u. Imaginary frequency=162i

N	−0.034984000	−0.233809000	1.360483000
P	2.688116000	−0.612902000	0.161498000
P	−2.729164000	−0.509566000	0.094076000
Si	0.038684000	5.149805000	−0.383759000
C	0.210658000	3.311241000	−0.864242000
H	−0.077693000	2.720452000	0.023107000
H	1.280013000	3.112103000	−1.034866000
C	−1.786641000	5.572493000	−0.143053000
H	−2.357312000	5.466843000	−1.073596000
H	−1.906022000	6.608512000	0.196755000
H	−2.249732000	4.922450000	0.609496000
C	0.972411000	5.433000000	1.232280000
H	0.535571000	4.844752000	2.048369000
H	0.938538000	6.488172000	1.529558000

H	2.026779000	5.145429000	1.143745000
C	0.773163000	6.231736000	−1.747493000
H	1.833558000	6.004856000	−1.912660000
H	0.701647000	7.294268000	−1.484786000
H	0.251405000	6.096552000	−2.702443000
C	4.067521000	−1.668987000	−0.455168000
C	3.819560000	−2.576667000	−1.492897000
H	2.827062000	−2.635214000	−1.934292000
C	4.839494000	−3.393549000	−1.978049000
H	4.638433000	−4.092170000	−2.786056000
C	6.120783000	−3.303122000	−1.435730000
H	6.918480000	−3.934855000	−1.816537000
C	6.380400000	−2.390865000	−0.413027000
H	7.380539000	−2.310092000	0.003972000
C	5.361875000	−1.574062000	0.074458000
H	5.576532000	−0.853785000	0.859233000
C	3.531360000	0.957388000	0.648070000
C	3.958528000	1.819263000	−0.372329000
H	3.774475000	1.568201000	−1.414824000
C	4.628850000	2.998894000	−0.057665000
H	4.964733000	3.657718000	−0.853918000
C	4.866075000	3.333213000	1.276711000
H	5.388788000	4.253797000	1.521594000
C	4.432674000	2.484599000	2.294208000
H	4.617259000	2.741688000	3.333859000
C	3.767481000	1.297801000	1.984422000
H	3.441126000	0.634497000	2.780594000
C	2.109488000	−1.364746000	1.735446000
C	0.803450000	−1.010859000	2.171669000
C	0.381005000	−1.520527000	3.416634000
H	−0.607524000	−1.260548000	3.781633000
C	1.193405000	−2.357721000	4.170674000
H	0.825890000	−2.736555000	5.121235000
C	2.460136000	−2.728304000	3.715297000
H	3.089778000	−3.391311000	4.300940000

C	2.908104000	−2.225336000	2.498293000
H	3.896521000	−2.495642000	2.136359000
C	−0.993712000	0.593823000	1.975959000
C	−0.663080000	1.446792000	3.046228000
H	0.351593000	1.428902000	3.433781000
C	−1.605081000	2.309705000	3.592624000
H	−1.318910000	2.959641000	4.415706000
C	−2.905481000	2.361081000	3.082867000
H	−3.637515000	3.042710000	3.505914000
C	−3.255727000	1.526009000	2.027175000
H	−4.265105000	1.555942000	1.626269000
C	−2.322929000	0.634196000	1.482154000
C	−4.299174000	0.122439000	−0.634418000
C	−5.553201000	−0.249762000	−0.131171000
H	−5.623011000	−0.939524000	0.705403000
C	−6.715923000	0.259526000	−0.707177000
H	−7.684730000	−0.033805000	−0.311880000
C	−6.637529000	1.140929000	−1.785247000
H	−7.546108000	1.534895000	−2.232392000
C	−5.392350000	1.512596000	−2.291695000
H	−5.327191000	2.195892000	−3.134236000
C	−4.227233000	1.001581000	−1.723153000
H	−3.258865000	1.288197000	−2.124112000
C	−3.224370000	−2.101162000	0.893266000
C	−3.312951000	−2.256540000	2.280071000
H	−3.104845000	−1.413999000	2.933141000
C	−3.683451000	−3.486457000	2.825721000
H	−3.753066000	−3.596450000	3.904638000
C	−3.974171000	−4.566031000	1.993869000
H	−4.267660000	−5.520536000	2.421983000
C	−3.898195000	−4.415270000	0.608107000
H	−4.141468000	−5.249518000	−0.044903000
C	−3.519818000	−3.191480000	0.060513000
H	−3.482389000	−3.075044000	−1.021559000
C	1.490027000	1.643098000	−4.186887000

C	0.909158000	1.089478000	−3.115289000
H	2.472605000	1.334721000	−4.537878000
H	0.998185000	2.436625000	−4.746180000
H	1.558965000	0.300501000	−2.649654000
C	−0.416351000	1.395480000	−2.478019000
C	−0.612162000	2.868649000	−2.083657000
H	−1.676923000	3.027734000	−1.866647000
H	−0.381445000	3.531265000	−2.934113000
Y	0.002721000	−0.297173000	−0.903478000
H	−1.214208000	1.090052000	−3.177717000
O	−0.065181000	−2.344787000	−2.032900000
C	−0.351150000	−2.493621000	−3.460988000
C	−0.034530000	−3.663835000	−1.387930000
C	−0.053845000	−3.952226000	−3.759066000
H	−1.406287000	−2.246402000	−3.622040000
H	0.276838000	−1.779477000	−3.998937000
C	−0.488279000	−4.641962000	−2.462614000
H	0.994962000	−3.843555000	−1.064727000
H	−0.693163000	−3.629025000	−0.516509000
H	1.017075000	−4.097789000	−3.939757000
H	−0.599244000	−4.308555000	−4.637090000
H	−0.035398000	−5.628044000	−2.329098000
H	−1.576805000	−4.762520000	−2.437886000

107

thf_C1_trans_1,2 E = −2044.747494 a.u.

N	0.035620000	−0.518874000	−1.583506000
P	−2.725373000	−0.103461000	−0.448461000
P	2.723681000	−0.472519000	−0.273234000
Si	0.415798000	4.171186000	0.349969000
C	−0.449019000	2.567427000	0.809667000
H	−1.386161000	2.596117000	0.215843000
H	−0.789773000	2.669317000	1.859392000
C	1.742628000	4.638653000	1.624231000
H	1.289088000	4.883806000	2.593383000
H	2.285793000	5.533129000	1.294424000

H	2.485663000	3.850710000	1.796685000
C	1.215840000	4.034552000	−1.361484000
H	1.989993000	3.259357000	−1.414072000
H	1.682161000	4.984757000	−1.650941000
H	0.468054000	3.795480000	−2.128313000
C	−0.816024000	5.608784000	0.289332000
H	−1.603901000	5.425324000	−0.451778000
H	−0.324185000	6.552884000	0.022868000
H	−1.307113000	5.753887000	1.259798000
C	−3.939581000	−0.865539000	0.715066000
C	−4.276131000	−2.224863000	0.731971000
H	−3.866615000	−2.898673000	−0.014091000
C	−5.156762000	−2.722602000	1.695408000
H	−5.422595000	−3.776610000	1.683875000
C	−5.704145000	−1.874307000	2.655808000
H	−6.396232000	−2.262898000	3.397807000
C	−5.365281000	−0.519746000	2.654634000
H	−5.790602000	0.150641000	3.396804000
C	−4.486156000	−0.020007000	1.696861000
H	−4.236727000	1.039374000	1.701202000
C	−3.683832000	1.191434000	−1.354896000
C	−2.957567000	2.087704000	−2.152597000
H	−1.873146000	2.021769000	−2.202359000
C	−3.620743000	3.059100000	−2.898680000
H	−3.049640000	3.747637000	−3.515683000
C	−5.011767000	3.151224000	−2.848072000
H	−5.528223000	3.913254000	−3.425192000
C	−5.737339000	2.262514000	−2.056972000
H	−6.821553000	2.326095000	−2.019050000
C	−5.079376000	1.282574000	−1.313882000
H	−5.658498000	0.592450000	−0.707578000
C	−2.289858000	−1.333814000	−1.734311000
C	−0.929589000	−1.376510000	−2.139341000
C	−0.584545000	−2.328626000	−3.124234000
H	0.452985000	−2.405561000	−3.434075000

C	−1.538509000	−3.145918000	−3.713055000
H	−1.229106000	−3.862244000	−4.470152000
C	−2.886061000	−3.047301000	−3.354475000
H	−3.637374000	−3.663429000	−3.839198000
C	−3.247984000	−2.138007000	−2.369257000
H	−4.295086000	−2.030733000	−2.097281000
C	1.048288000	−0.059874000	−2.449455000
C	0.766939000	0.365584000	−3.763716000
H	−0.254727000	0.301662000	−4.127278000
C	1.766594000	0.864242000	−4.588558000
H	1.512473000	1.189943000	−5.594113000
C	3.084859000	0.965380000	−4.135131000
H	3.863731000	1.358655000	−4.781530000
C	3.387201000	0.558347000	−2.841185000
H	4.406670000	0.635160000	−2.473322000
C	2.390046000	0.040569000	−2.003311000
C	4.322375000	0.330811000	0.169805000
C	5.532422000	−0.369658000	0.237703000
H	5.564624000	−1.431785000	0.013929000
C	6.706159000	0.297249000	0.590075000
H	7.641212000	−0.254187000	0.639493000
C	6.683786000	1.662146000	0.871190000
H	7.600631000	2.176898000	1.144715000
C	5.481698000	2.366973000	0.798683000
H	5.457003000	3.431940000	1.012664000
C	4.306245000	1.703947000	0.455582000
H	3.375452000	2.263430000	0.394327000
C	3.102238000	−2.277968000	−0.322576000
C	3.218297000	−2.983815000	−1.524676000
H	3.102379000	−2.463220000	−2.471034000
C	3.496610000	−4.350937000	−1.509212000
H	3.588233000	−4.891163000	−2.447661000
C	3.668662000	−5.020140000	−0.298390000
H	3.892302000	−6.083390000	−0.290816000
C	3.563084000	−4.319924000	0.904588000

H	3.710992000	−4.834610000	1.850424000
C	3.274127000	−2.956747000	0.893881000
H	3.205821000	−2.413993000	1.835172000
C	−0.555621000	2.260571000	5.617145000
C	0.267722000	1.325023000	5.120909000
H	−1.143527000	2.086139000	6.513376000
H	−0.653753000	3.238217000	5.149969000
H	0.359033000	0.365166000	5.630650000
C	1.100914000	1.532178000	3.946123000
C	1.923354000	0.608910000	3.409167000
H	2.604838000	0.860054000	2.600265000
H	2.024986000	−0.385572000	3.841478000
Y	0.002797000	0.285667000	0.553499000
H	1.067187000	2.525610000	3.501439000
O	−0.604951000	−1.516773000	1.953968000
C	−1.236187000	−1.530485000	3.273600000
C	−0.489631000	−2.885568000	1.445754000
C	−1.629525000	−2.980958000	3.523248000
H	−0.491774000	−1.168759000	3.986518000
H	−2.086511000	−0.845009000	3.250155000
C	−0.616354000	−3.759327000	2.679836000
H	−1.304516000	−3.052596000	0.734104000
H	0.470093000	−2.969831000	0.931440000
H	−2.647342000	−3.164420000	3.166646000
H	−1.586624000	−3.234425000	4.586195000
H	−0.953404000	−4.769278000	2.430621000
H	0.347687000	−3.837657000	3.195263000

107

thf_TS1_trans_1,2 E = −2044.724758 a.u. Imaginary frequency=332i

N	−0.190856000	−0.383494000	1.528087000
P	2.666807000	−0.198863000	0.383600000
P	−2.778755000	−0.266210000	0.050753000
Si	−0.197449000	4.119249000	−0.370758000
C	0.748467000	2.645547000	−1.143012000
H	1.338307000	2.154289000	−0.329990000

H	1.555603000	3.126322000	−1.703822000
C	−1.223826000	5.014179000	−1.681247000
H	−0.609798000	5.367533000	−2.518563000
H	−1.687472000	5.899965000	−1.229004000
H	−2.035556000	4.403335000	−2.094242000
C	−1.322978000	3.606275000	1.052943000
H	−2.173862000	2.986953000	0.747521000
H	−1.746185000	4.507707000	1.514968000
H	−0.786997000	3.066738000	1.842931000
C	1.111830000	5.310482000	0.284379000
H	1.773492000	4.826779000	1.012378000
H	0.644413000	6.172063000	0.777407000
H	1.740543000	5.694970000	−0.527911000
C	3.777239000	−1.187212000	−0.713171000
C	4.085623000	−2.540434000	−0.527503000
H	3.706321000	−3.074312000	0.337928000
C	4.891724000	−3.213104000	−1.448338000
H	5.133142000	−4.260020000	−1.283113000
C	5.395789000	−2.548479000	−2.564697000
H	6.029261000	−3.073976000	−3.273900000
C	5.085988000	−1.202440000	−2.764629000
H	5.476508000	−0.674240000	−3.630474000
C	4.276579000	−0.529649000	−1.851576000
H	4.045606000	0.521097000	−2.018185000
C	3.749789000	1.083946000	1.159614000
C	3.141146000	1.938878000	2.091257000
H	2.089185000	1.813928000	2.341153000
C	3.884945000	2.930418000	2.725661000
H	3.407215000	3.579985000	3.454363000
C	5.240163000	3.085126000	2.429991000
H	5.820340000	3.859681000	2.923783000
C	5.847970000	2.238372000	1.505872000
H	6.905485000	2.346786000	1.280227000
C	5.108940000	1.238982000	0.870982000
H	5.601101000	0.578254000	0.163636000

C	2.123555000	−1.236667000	1.788529000
C	0.750368000	−1.205551000	2.164448000
C	0.372408000	−2.078889000	3.214654000
H	−0.672634000	−2.118184000	3.505312000
C	1.293293000	−2.860069000	3.894565000
H	0.947951000	−3.507839000	4.696744000
C	2.653232000	−2.811040000	3.571676000
H	3.381957000	−3.391669000	4.129005000
C	3.050573000	−1.998061000	2.520341000
H	4.105350000	−1.930705000	2.264739000
C	−1.247738000	0.090874000	2.329327000
C	−1.039777000	0.542700000	3.650338000
H	−0.041223000	0.481928000	4.074065000
C	−2.079041000	1.060689000	4.412314000
H	−1.874965000	1.403760000	5.423657000
C	−3.371144000	1.158025000	3.889619000
H	−4.181905000	1.566332000	4.485458000
C	−3.600514000	0.734573000	2.586089000
H	−4.595402000	0.822075000	2.157939000
C	−2.562676000	0.203014000	1.809706000
C	−4.317229000	0.578418000	−0.515318000
C	−5.585728000	0.017097000	−0.312079000
H	−5.682499000	−0.949764000	0.173716000
C	−6.728119000	0.692634000	−0.737594000
H	−7.706619000	0.248788000	−0.575097000
C	−6.617827000	1.930994000	−1.369629000
H	−7.510370000	2.453495000	−1.702562000
C	−5.359897000	2.494588000	−1.578718000
H	−5.268138000	3.456496000	−2.075850000
C	−4.215664000	1.819234000	−1.157971000
H	−3.239152000	2.262973000	−1.333252000
C	−3.255986000	−2.051652000	0.040998000
C	−3.362853000	−2.802984000	1.216098000
H	−3.182251000	−2.330504000	2.177641000
C	−3.718458000	−4.151700000	1.155148000

H	−3.805861000	−4.726486000	2.073372000
C	−3.976143000	−4.755256000	−0.074589000
H	−4.262962000	−5.802434000	−0.117999000
C	−3.875485000	−4.008414000	−1.250606000
H	−4.090017000	−4.470922000	−2.210664000
C	−3.509218000	−2.665483000	−1.195619000
H	−3.442309000	−2.087288000	−2.115008000
C	1.794594000	2.823575000	−4.503268000
C	1.275843000	1.843077000	−3.757409000
H	2.676992000	2.666624000	−5.117002000
H	1.341719000	3.812144000	−4.544774000
H	1.753525000	0.860978000	−3.765900000
C	0.028998000	1.957648000	−2.966170000
C	−0.832489000	0.801306000	−2.958840000
H	−1.904786000	0.999530000	−2.970653000
H	−0.545161000	0.009056000	−3.652609000
Y	−0.011701000	0.273988000	−0.734425000
H	−0.445738000	2.931435000	−3.050144000
O	0.354065000	−1.942162000	−1.490662000
C	0.773536000	−2.342562000	−2.830641000
C	0.197224000	−3.133830000	−0.648075000
C	1.108612000	−3.822518000	−2.728702000
H	−0.065298000	−2.156956000	−3.508130000
H	1.625984000	−1.721940000	−3.118131000
C	0.163808000	−4.296525000	−1.623387000
H	1.057352000	−3.177950000	0.025360000
H	−0.716793000	−3.007356000	−0.067838000
H	2.152857000	−3.957067000	−2.430001000
H	0.952640000	−4.338092000	−3.680572000
H	0.488565000	−5.228822000	−1.152865000
H	−0.850837000	−4.441460000	−2.009029000

107

thf_P1_trans_1,2 E = −2044.74619 a.u.

N	−0.054404000	0.058225000	1.502059000
P	2.315472000	−1.288630000	0.188329000

P	−2.747080000	0.019641000	0.228136000
Si	1.930406000	5.268149000	−0.341967000
C	2.002720000	3.457047000	−0.953939000
H	1.912838000	2.796821000	−0.073658000
H	3.029493000	3.328551000	−1.329511000
C	2.407846000	6.399765000	−1.775702000
H	3.401439000	6.154940000	−2.170313000
H	2.432629000	7.447041000	−1.450830000
H	1.695073000	6.335509000	−2.606760000
C	0.222588000	5.746168000	0.301097000
H	−0.536144000	5.704841000	−0.489062000
H	0.239398000	6.773373000	0.686570000
H	−0.107169000	5.092276000	1.116952000
C	3.199858000	5.424892000	1.047896000
H	2.953216000	4.774826000	1.896653000
H	3.242556000	6.453402000	1.426072000
H	4.208217000	5.161216000	0.705913000
C	2.228075000	−2.996244000	−0.516139000
C	1.609787000	−4.051896000	0.165545000
H	1.233211000	−3.899838000	1.172794000
C	1.487734000	−5.304865000	−0.438591000
H	1.013642000	−6.117586000	0.105406000
C	1.984075000	−5.518730000	−1.723366000
H	1.899836000	−6.498502000	−2.185739000
C	2.602266000	−4.471113000	−2.410259000
H	3.003704000	−4.633858000	−3.407285000
C	2.714785000	−3.216155000	−1.815682000
H	3.205388000	−2.409988000	−2.356936000
C	4.118227000	−0.883963000	0.228147000
C	4.474346000	0.439653000	0.525701000
H	3.703209000	1.186541000	0.703004000
C	5.815013000	0.806182000	0.614480000
H	6.078928000	1.833381000	0.851056000
C	6.814146000	−0.143223000	0.398176000
H	7.860271000	0.142849000	0.462961000

C	6.466800000	−1.459821000	0.101750000
H	7.241336000	−2.204613000	−0.060760000
C	5.125191000	−1.833059000	0.018423000
H	4.869226000	−2.864410000	−0.205686000
C	1.832390000	−1.440504000	1.943229000
C	0.693021000	−0.720076000	2.388568000
C	0.350172000	−0.862589000	3.753462000
H	−0.518827000	−0.339142000	4.138547000
C	1.103454000	−1.644513000	4.617128000
H	0.800268000	−1.720119000	5.658643000
C	2.238133000	−2.327108000	4.170392000
H	2.833499000	−2.927148000	4.851638000
C	2.589995000	−2.215936000	2.832471000
H	3.474829000	−2.729669000	2.463646000
C	−0.900024000	1.057269000	2.034373000
C	−0.444629000	1.999656000	2.977655000
H	0.575898000	1.921879000	3.342638000
C	−1.276498000	3.010770000	3.441131000
H	−0.897809000	3.721470000	4.171391000
C	−2.585583000	3.128866000	2.965013000
H	−3.233122000	3.923296000	3.324267000
C	−3.051705000	2.221601000	2.020928000
H	−4.063599000	2.313735000	1.637255000
C	−2.231345000	1.181621000	1.563525000
C	−4.287922000	0.739511000	−0.482637000
C	−5.544464000	0.529125000	0.103056000
H	−5.634739000	−0.086640000	0.993592000
C	−6.683452000	1.104646000	−0.457273000
H	−7.653021000	0.936561000	0.003747000
C	−6.580587000	1.893849000	−1.602862000
H	−7.470781000	2.340622000	−2.037110000
C	−5.334426000	2.107331000	−2.190948000
H	−5.249719000	2.722062000	−3.083148000
C	−4.193909000	1.529216000	−1.636194000
H	−3.224572000	1.703332000	−2.096323000

C	−3.322401000	−1.529366000	1.053807000
C	−3.093572000	−1.779951000	2.409734000
H	−2.609016000	−1.028830000	3.024962000
C	−3.498632000	−2.988052000	2.978099000
H	−3.319622000	−3.170227000	4.034318000
C	−4.137839000	−3.951696000	2.200570000
H	−4.458067000	−4.888800000	2.647635000
C	−4.376182000	−3.705029000	0.847519000
H	−4.888782000	−4.445906000	0.239499000
C	−3.966641000	−2.503278000	0.274849000
H	−4.178942000	−2.310970000	−0.775116000
C	2.360545000	0.958832000	−2.602232000
C	1.398778000	1.863445000	−2.868003000
H	2.524952000	0.102874000	−3.253774000
H	3.083212000	1.099478000	−1.802245000
H	0.760813000	1.694107000	−3.740360000
C	0.984690000	3.048808000	−2.028856000
C	−0.345315000	2.549594000	−1.410127000
H	−0.642361000	3.183604000	−0.563356000
H	−1.151664000	2.636271000	−2.158044000
Y	−0.005399000	0.272192000	−0.765361000
H	0.826136000	3.897048000	−2.714597000
O	−0.558012000	−1.299663000	−2.460316000
C	−0.756040000	−0.998507000	−3.875356000
C	−1.015611000	−2.659841000	−2.177521000
C	−1.217929000	−2.304094000	−4.510250000
H	−1.516978000	−0.213628000	−3.946019000
H	0.189272000	−0.626543000	−4.276989000
C	−1.916766000	−3.011740000	−3.347148000
H	−0.135298000	−3.306190000	−2.122800000
H	−1.522388000	−2.643620000	−1.209215000
H	−0.358399000	−2.889913000	−4.853513000
H	−1.874959000	−2.129290000	−5.366486000
H	−1.997065000	−4.092693000	−3.490332000
H	−2.924516000	−2.607767000	−3.197264000

107

thf_C1_cis_1,2 E = −2044.729627 a.u.

N	0.085959000	−0.270591000	1.454786000
P	2.827506000	0.102090000	0.170829000
P	−2.636157000	−0.492237000	0.172773000
Si	−1.099077000	4.246240000	−0.670136000
C	−0.210545000	2.591566000	−0.548434000
H	−0.071305000	2.464050000	0.549291000
H	0.814613000	2.774005000	−0.941600000
C	−1.787078000	4.610291000	−2.403140000
H	−1.017441000	4.563159000	−3.184343000
H	−2.193505000	5.629402000	−2.424228000
H	−2.606068000	3.939378000	−2.691095000
C	−2.519889000	4.321210000	0.571040000
H	−3.287580000	3.564442000	0.377122000
H	−3.006921000	5.303881000	0.541528000
H	−2.155407000	4.163517000	1.593246000
C	0.103373000	5.656253000	−0.269010000
H	0.512751000	5.551369000	0.743622000
H	−0.393105000	6.633400000	−0.321963000
H	0.949481000	5.679729000	−0.967772000
C	4.011511000	−0.866761000	−0.869232000
C	4.122005000	−2.261396000	−0.799073000
H	3.575111000	−2.812890000	−0.040551000
C	4.964815000	−2.950360000	−1.673075000
H	5.055276000	−4.030596000	−1.591041000
C	5.704575000	−2.258301000	−2.629850000
H	6.366842000	−2.795407000	−3.303139000
C	5.603554000	−0.868596000	−2.706230000
H	6.188980000	−0.318283000	−3.438061000
C	4.763219000	−0.178327000	−1.835790000
H	4.715188000	0.906975000	−1.889689000
C	3.855317000	1.504968000	0.803179000
C	3.232318000	2.721614000	1.106294000
H	2.171470000	2.857007000	0.916138000

C	3.970353000	3.771328000	1.652774000
H	3.477445000	4.712197000	1.881874000
C	5.333573000	3.616266000	1.895692000
H	5.908516000	4.435865000	2.318009000
C	5.960472000	2.407394000	1.591757000
H	7.023811000	2.282148000	1.777761000
C	5.227615000	1.354983000	1.048887000
H	5.729789000	0.422231000	0.808410000
C	2.456651000	−0.922825000	1.650665000
C	1.117062000	−0.946502000	2.122264000
C	0.858127000	−1.724986000	3.273357000
H	−0.160149000	−1.779506000	3.645868000
C	1.866947000	−2.408685000	3.935302000
H	1.620692000	−2.994560000	4.817484000
C	3.187846000	−2.351756000	3.480368000
H	3.981082000	−2.874598000	4.006145000
C	3.467375000	−1.608459000	2.341882000
H	4.491220000	−1.550244000	1.981377000
C	−0.938674000	0.288312000	2.245162000
C	−0.660610000	0.934015000	3.467844000
H	0.366572000	0.969259000	3.819486000
C	−1.668126000	1.525231000	4.219037000
H	−1.413754000	2.015589000	5.155411000
C	−2.992828000	1.510782000	3.775384000
H	−3.778795000	1.978757000	4.360533000
C	−3.290196000	0.898352000	2.563830000
H	−4.313518000	0.895004000	2.198321000
C	−2.287386000	0.282712000	1.803440000
C	−4.231352000	0.224931000	−0.408178000
C	−5.471967000	−0.334741000	−0.074534000
H	−5.521536000	−1.222917000	0.548835000
C	−6.649741000	0.243477000	−0.546429000
H	−7.607413000	−0.197039000	−0.282456000
C	−6.601258000	1.380761000	−1.351871000
H	−7.521313000	1.827441000	−1.718660000

C	−5.369761000	1.943261000	−1.686904000
H	−5.327332000	2.831910000	−2.311024000
C	−4.190170000	1.365100000	−1.221719000
H	−3.230551000	1.808019000	−1.476042000
C	−3.073445000	−2.251416000	0.535943000
C	−3.100027000	−2.761727000	1.838261000
H	−2.872686000	−2.111451000	2.678243000
C	−3.434353000	−4.098736000	2.061013000
H	−3.457816000	−4.484494000	3.076750000
C	−3.750989000	−4.931888000	0.989758000
H	−4.019993000	−5.969575000	1.167187000
C	−3.731078000	−4.427480000	−0.312426000
H	−3.991610000	−5.069467000	−1.150077000
C	−3.386550000	−3.097043000	−0.539500000
H	−3.384152000	−2.708714000	−1.556164000
C	1.570423000	0.684234000	−3.426306000
C	0.540083000	1.551442000	−3.461780000
H	2.597240000	1.033014000	−3.418549000
H	1.437161000	−0.394340000	−3.474664000
H	0.763999000	2.615198000	−3.434303000
C	−0.891141000	1.219383000	−3.477815000
C	−1.430058000	0.015938000	−3.751381000
H	−2.506305000	−0.127123000	−3.726436000
H	−0.831205000	−0.832633000	−4.069140000
Y	0.032522000	0.267494000	−0.760530000
H	−1.553033000	2.047730000	−3.246660000
O	0.295983000	−2.148992000	−1.500401000
C	0.338462000	−2.887636000	−2.747475000
C	0.349240000	−3.108083000	−0.385343000
C	0.912805000	−4.248251000	−2.398759000
H	−0.683062000	−2.971746000	−3.138282000
H	0.954613000	−2.332019000	−3.458045000
C	0.303070000	−4.491688000	−1.018752000
H	1.282222000	−2.927903000	0.152643000
H	−0.491902000	−2.903052000	0.276651000

H	2.005229000	−4.193393000	−2.341388000
H	0.641365000	−5.010145000	−3.134983000
H	0.856391000	−5.225350000	−0.425770000
H	−0.732098000	−4.837566000	−1.108294000

107

thf_TS1_cis_1,2 E = −2044.707117 a.u. Imaginary frequency=327i

N	−0.205361000	−0.340356000	1.499763000
P	2.654392000	−0.240385000	0.325637000
P	−2.805689000	−0.333293000	0.048848000
Si	0.011143000	4.490403000	−0.624642000
C	0.025844000	2.588684000	−0.667938000
H	−0.879664000	2.326499000	−0.075757000
H	0.916535000	2.291163000	−0.073442000
C	1.437002000	5.249641000	−1.602156000
H	2.408379000	4.835715000	−1.304618000
H	1.472721000	6.327727000	−1.401218000
H	1.329420000	5.131942000	−2.686573000
C	−1.633653000	5.132778000	−1.297703000
H	−1.785804000	4.921046000	−2.362908000
H	−1.674263000	6.223075000	−1.182599000
H	−2.484984000	4.719022000	−0.743390000
C	0.170425000	5.012859000	1.182130000
H	−0.618297000	4.570943000	1.802508000
H	0.098670000	6.103417000	1.280017000
H	1.136722000	4.707820000	1.601013000
C	3.713949000	−1.388378000	−0.664750000
C	3.965192000	−2.723511000	−0.324499000
H	3.567091000	−3.137354000	0.596326000
C	4.740178000	−3.531527000	−1.158912000
H	4.937038000	−4.561647000	−0.873154000
C	5.271425000	−3.020910000	−2.341900000
H	5.882617000	−3.649789000	−2.983364000
C	5.016885000	−1.695346000	−2.696334000
H	5.427664000	−1.287764000	−3.616386000
C	4.237349000	−0.888916000	−1.869626000

H	4.041333000	0.141073000	−2.158253000
C	3.795792000	1.077902000	0.946054000
C	3.245934000	2.027521000	1.820849000
H	2.204409000	1.950902000	2.126309000
C	4.036521000	3.055047000	2.328950000
H	3.604344000	3.778410000	3.015343000
C	5.380132000	3.151066000	1.963891000
H	5.996362000	3.953219000	2.360393000
C	5.930163000	2.209345000	1.097301000
H	6.978910000	2.270866000	0.819130000
C	5.144143000	1.174159000	0.588830000
H	5.592764000	0.439648000	−0.073014000
C	2.119516000	−1.125261000	1.838100000
C	0.748609000	−1.074714000	2.215742000
C	0.383511000	−1.826997000	3.359340000
H	−0.660897000	−1.844625000	3.655530000
C	1.316592000	−2.515898000	4.117296000
H	0.983783000	−3.073514000	4.989412000
C	2.674139000	−2.488956000	3.780345000
H	3.412080000	−2.997480000	4.393295000
C	3.058102000	−1.794121000	2.643104000
H	4.110807000	−1.747839000	2.374200000
C	−1.227597000	0.269928000	2.246299000
C	−0.989802000	0.870557000	3.502072000
H	0.007864000	0.820155000	3.928794000
C	−2.001019000	1.521518000	4.196364000
H	−1.775428000	1.975119000	5.158463000
C	−3.292180000	1.611676000	3.668620000
H	−4.080177000	2.122867000	4.213496000
C	−3.550437000	1.041079000	2.428087000
H	−4.545537000	1.110219000	1.996861000
C	−2.541740000	0.373918000	1.720545000
C	−4.315604000	0.506767000	−0.596255000
C	−5.593866000	−0.054575000	−0.482243000
H	−5.724758000	−1.023366000	−0.009412000

C	−6.705828000	0.627795000	−0.975414000
H	−7.692859000	0.182762000	−0.882470000
C	−6.554771000	1.874329000	−1.580556000
H	−7.423250000	2.402178000	−1.964790000
C	−5.285586000	2.442286000	−1.692430000
H	−5.161294000	3.413886000	−2.163012000
C	−4.172131000	1.760154000	−1.207715000
H	−3.187074000	2.209667000	−1.302059000
C	−3.335432000	−2.085049000	0.295680000
C	−3.433612000	−2.663439000	1.565423000
H	−3.231656000	−2.064669000	2.449073000
C	−3.806466000	−4.002011000	1.698710000
H	−3.885268000	−4.441777000	2.689425000
C	−4.091024000	−4.767879000	0.569525000
H	−4.389991000	−5.806987000	0.677166000
C	−4.001039000	−4.194395000	−0.700592000
H	−4.237210000	−4.783509000	−1.583085000
C	−3.616761000	−2.862387000	−0.839142000
H	−3.556959000	−2.419003000	−1.831294000
C	1.823161000	1.171153000	−3.922929000
C	1.190780000	2.048507000	−3.127995000
H	2.875327000	1.298678000	−4.161364000
H	1.313427000	0.346421000	−4.412607000
H	1.756288000	2.883534000	−2.724607000
C	−0.245173000	1.994799000	−2.760643000
C	−1.000057000	0.813910000	−2.966751000
H	−2.075432000	0.899121000	−3.093812000
H	−0.542368000	0.013022000	−3.550128000
Y	−0.045713000	0.145818000	−0.782183000
H	−0.765362000	2.945342000	−2.825493000
O	0.288514000	−2.085385000	−1.533757000
C	0.694271000	−2.540586000	−2.859119000
C	0.124913000	−3.237995000	−0.642136000
C	0.997201000	−4.024081000	−2.706407000
H	−0.143444000	−2.358231000	−3.540850000

H	1.559904000	−1.949136000	−3.166609000
C	0.059918000	−4.437914000	−1.570064000
H	0.993409000	−3.270370000	0.022173000
H	−0.780004000	−3.074679000	−0.055640000
H	2.042854000	−4.169929000	−2.417449000
H	0.816390000	−4.572501000	−3.635165000
H	0.375768000	−5.356728000	−1.068155000
H	−0.962468000	−4.581583000	−1.935982000

107

thf_P1_cis_1,2 E = −2044.751773 a.u.

N	0.031542000	0.118471000	1.436340000
P	2.713537000	−0.534509000	0.160843000
P	−2.605644000	−0.603004000	0.241137000
Si	−0.239998000	5.365194000	−0.516111000
C	0.690038000	3.713322000	−0.774017000
H	0.522749000	3.080842000	0.114368000
H	1.755805000	3.991017000	−0.750790000
C	0.225445000	6.535978000	−1.923543000
H	1.309016000	6.699007000	−1.972865000
H	−0.245837000	7.515894000	−1.778881000
H	−0.100275000	6.163368000	−2.902227000
C	−2.113865000	5.153688000	−0.460372000
H	−2.518667000	4.817783000	−1.421993000
H	−2.592363000	6.111879000	−0.221496000
H	−2.418295000	4.432257000	0.307000000
C	0.362307000	6.076226000	1.126464000
H	0.108112000	5.416693000	1.965001000
H	−0.097870000	7.051807000	1.324755000
H	1.449572000	6.221130000	1.132650000
C	3.228265000	−2.202913000	−0.448154000
C	3.038117000	−3.371903000	0.299544000
H	2.646150000	−3.312101000	1.310667000
C	3.363947000	−4.616267000	−0.243591000
H	3.220101000	−5.514169000	0.351743000
C	3.885324000	−4.708751000	−1.533035000

H	4.149711000	−5.677772000	−1.947601000
C	4.077984000	−3.548381000	−2.286280000
H	4.494049000	−3.610132000	−3.288573000
C	3.743507000	−2.305869000	−1.751870000
H	3.904380000	−1.408832000	−2.345871000
C	4.263015000	0.471625000	0.150859000
C	4.134479000	1.851056000	0.367742000
H	3.150479000	2.291098000	0.513824000
C	5.264033000	2.664277000	0.415464000
H	5.154124000	3.731190000	0.590651000
C	6.532150000	2.110325000	0.237201000
H	7.413203000	2.745238000	0.270031000
C	6.665691000	0.740366000	0.019941000
H	7.651529000	0.302767000	−0.112878000
C	5.537738000	−0.079639000	−0.021196000
H	5.657761000	−1.146663000	−0.184001000
C	2.281901000	−0.744237000	1.922611000
C	0.973885000	−0.382370000	2.341335000
C	0.672808000	−0.583806000	3.708811000
H	−0.320234000	−0.338986000	4.071166000
C	1.615353000	−1.072358000	4.601168000
H	1.334762000	−1.206731000	5.643063000
C	2.911185000	−1.387648000	4.181980000
H	3.652421000	−1.753622000	4.885759000
C	3.228848000	−1.219990000	2.841833000
H	4.232342000	−1.451893000	2.492002000
C	−1.017674000	0.911657000	1.950488000
C	−0.783444000	1.965176000	2.857409000
H	0.231513000	2.141338000	3.203001000
C	−1.822530000	2.766490000	3.313330000
H	−1.609024000	3.568052000	4.015979000
C	−3.130657000	2.559427000	2.866456000
H	−3.941575000	3.188766000	3.221123000
C	−3.384058000	1.537758000	1.958103000
H	−4.395702000	1.375256000	1.596888000

C	−2.349288000	0.706915000	1.509019000
C	−4.266332000	−0.272999000	−0.487008000
C	−5.440627000	−0.800002000	0.067926000
H	−5.391074000	−1.430466000	0.951203000
C	−6.676248000	−0.520240000	−0.513600000
H	−7.581682000	−0.933025000	−0.076885000
C	−6.751484000	0.287124000	−1.648371000
H	−7.716298000	0.502768000	−2.099274000
C	−5.586982000	0.817173000	−2.203729000
H	−5.641548000	1.448782000	−3.086486000
C	−4.348541000	0.535189000	−1.629825000
H	−3.444855000	0.955939000	−2.063738000
C	−2.818023000	−2.191633000	1.158589000
C	−2.624848000	−2.291009000	2.539422000
H	−2.392935000	−1.402299000	3.118022000
C	−2.742300000	−3.526519000	3.177439000
H	−2.593525000	−3.591928000	4.251901000
C	−3.059038000	−4.668875000	2.445115000
H	−3.156166000	−5.628259000	2.945825000
C	−3.263271000	−4.575465000	1.067223000
H	−3.527873000	−5.460164000	0.493778000
C	−3.137218000	−3.345489000	0.426397000
H	−3.319402000	−3.276885000	−0.644336000
C	1.523828000	0.977694000	−3.249636000
C	1.578893000	2.099500000	−2.509139000
H	2.424907000	0.459286000	−3.566395000
H	0.583431000	0.617517000	−3.666328000
H	2.564873000	2.460727000	−2.209674000
C	0.380183000	2.917574000	−2.062896000
C	−0.858940000	2.010922000	−1.889113000
H	−1.633952000	2.552879000	−1.333949000
H	−1.298212000	1.779943000	−2.875664000
Y	0.016307000	0.056296000	−0.848073000
H	0.228820000	3.651954000	−2.875834000
O	−0.272077000	−1.864402000	−2.216653000

C	−1.170542000	−1.856508000	−3.364843000
C	0.084053000	−3.246672000	−1.876881000
C	−0.978045000	−3.214984000	−4.015599000
H	−2.194190000	−1.711349000	−3.000192000
H	−0.892410000	−1.014533000	−4.003468000
C	−0.753885000	−4.119830000	−2.801885000
H	1.156581000	−3.356633000	−2.052546000
H	−0.134293000	−3.397929000	−0.816760000
H	−0.095385000	−3.206718000	−4.664854000
H	−1.843519000	−3.507213000	−4.616579000
H	−0.239111000	−5.051337000	−3.052014000
H	−1.709516000	−4.375825000	−2.332409000

94

C1_cis E = −1812.394692 a.u.

N	0.349985000	−1.492581000	−0.579383000
P	2.717261000	0.031392000	0.257286000
P	−2.383198000	−0.544819000	0.126190000
Si	−1.357225000	3.981136000	0.850687000
C	−0.267914000	2.448261000	0.928587000
H	−0.449039000	1.992424000	1.917303000
H	0.782598000	2.811764000	0.973839000
C	−3.024299000	3.669355000	1.681010000
H	−3.571063000	2.830624000	1.235109000
H	−3.662233000	4.559147000	1.611388000
H	−2.895682000	3.437737000	2.744895000
C	−0.520081000	5.447647000	1.706573000
H	−0.320958000	5.224707000	2.761903000
H	−1.147145000	6.347372000	1.670201000
H	0.440285000	5.696884000	1.237087000
C	−1.706418000	4.525088000	−0.941656000
H	−0.790454000	4.749872000	−1.505708000
H	−2.303939000	5.445460000	−0.946243000
H	−2.284328000	3.773714000	−1.496387000
C	4.329281000	0.916135000	0.187018000
C	4.336048000	2.295369000	−0.057237000

H	3.396619000	2.826690000	−0.191088000
C	5.539057000	2.999963000	−0.094739000
H	5.534318000	4.071193000	−0.277194000
C	6.742877000	2.331250000	0.119818000
H	7.680559000	2.879549000	0.096482000
C	6.743298000	0.960264000	0.382428000
H	7.680086000	0.441172000	0.565620000
C	5.543451000	0.254304000	0.421963000
H	5.550563000	−0.808139000	0.650998000
C	2.819112000	−1.025973000	1.759580000
C	2.484644000	−0.441480000	2.988807000
H	2.157236000	0.595436000	3.030818000
C	2.580863000	−1.184713000	4.163364000
H	2.326099000	−0.725874000	5.114861000
C	3.002330000	−2.513726000	4.116428000
H	3.074898000	−3.093002000	5.032761000
C	3.330779000	−3.098820000	2.894030000
H	3.660490000	−4.133609000	2.856506000
C	3.240164000	−2.359331000	1.715458000
H	3.496516000	−2.819150000	0.764817000
C	2.681631000	−1.119312000	−1.184995000
C	1.443269000	−1.784711000	−1.407651000
C	1.349550000	−2.647188000	−2.522508000
H	0.415692000	−3.175718000	−2.695058000
C	2.423226000	−2.823554000	−3.384688000
H	2.322340000	−3.494932000	−4.233770000
C	3.625986000	−2.137938000	−3.176288000
H	4.463262000	−2.277429000	−3.853923000
C	3.747134000	−1.288202000	−2.082118000
H	4.682668000	−0.763541000	−1.908170000
C	−0.451843000	−2.531255000	−0.098462000
C	0.019090000	−3.851239000	0.053324000
H	1.040984000	−4.085977000	−0.227830000
C	−0.794508000	−4.848417000	0.576643000
H	−0.394450000	−5.853460000	0.685672000

C	−2.103076000	−4.572993000	0.979921000
H	−2.731971000	−5.353784000	1.396587000
C	−2.583892000	−3.275185000	0.849216000
H	−3.594326000	−3.036520000	1.172316000
C	−1.782440000	−2.264211000	0.305884000
C	−3.339407000	−0.239684000	1.673266000
C	−2.611067000	−0.047134000	2.856033000
H	−1.523790000	−0.061579000	2.837144000
C	−3.273700000	0.146368000	4.064515000
H	−2.700950000	0.289736000	4.976847000
C	−4.668797000	0.160809000	4.103180000
H	−5.185534000	0.317913000	5.045917000
C	−5.396926000	−0.029289000	2.930910000
H	−6.483289000	−0.025286000	2.956261000
C	−4.737901000	−0.232644000	1.717773000
H	−5.317504000	−0.389106000	0.812846000
C	−3.634078000	−0.569926000	−1.234591000
C	−4.329781000	0.614617000	−1.528623000
H	−4.182057000	1.501118000	−0.915073000
C	−5.227116000	0.659345000	−2.593606000
H	−5.772924000	1.576057000	−2.801158000
C	−5.425952000	−0.471312000	−3.388955000
H	−6.124348000	−0.435799000	−4.220513000
C	−4.732192000	−1.647109000	−3.107099000
H	−4.890490000	−2.532565000	−3.717089000
C	−3.841725000	−1.700540000	−2.033145000
H	−3.317809000	−2.625513000	−1.809899000
C	−1.295225000	1.332963000	−3.330034000
C	−0.109282000	0.713188000	−3.510813000
H	−1.374443000	2.386016000	−3.061508000
H	−2.229228000	0.809718000	−3.509412000
C	1.219559000	1.297511000	−3.270153000
C	1.474923000	2.468826000	−2.647262000
H	2.496224000	2.809317000	−2.511062000
H	0.690846000	3.157058000	−2.324810000

H	−0.106653000	−0.321433000	−3.848979000
Y	0.103321000	0.769189000	−0.656397000
H	2.061239000	0.684664000	−3.586520000

94

TS1_cis E = −1812.384252 a.u. Imaginary frequency=108i

N	0.439686000	−1.632654000	−0.600562000
P	2.717946000	0.101941000	0.200391000
P	−2.271451000	−0.738025000	0.142212000
Si	−1.611566000	4.216712000	0.563662000
C	−0.613410000	2.668511000	0.152288000
H	−0.801105000	1.964866000	0.998345000
H	0.460712000	2.954169000	0.200555000
C	−3.464621000	3.871805000	0.426201000
H	−3.786311000	3.693959000	−0.607158000
H	−4.038381000	4.730743000	0.795511000
H	−3.754931000	3.002339000	1.028284000
C	−1.233445000	4.752264000	2.336676000
H	−1.509818000	3.973005000	3.057536000
H	−1.791830000	5.658635000	2.602412000
H	−0.167229000	4.970882000	2.473355000
C	−1.128443000	5.623655000	−0.604350000
H	−0.043883000	5.790875000	−0.612109000
H	−1.594103000	6.559897000	−0.273003000
H	−1.452931000	5.456992000	−1.638340000
C	4.310344000	1.016213000	0.101251000
C	4.340480000	2.270203000	−0.521053000
H	3.426121000	2.697505000	−0.925367000
C	5.534488000	2.983553000	−0.613176000
H	5.548326000	3.957285000	−1.095204000
C	6.705004000	2.451126000	−0.074888000
H	7.635370000	3.008372000	−0.140622000
C	6.680214000	1.208152000	0.559074000
H	7.590003000	0.796862000	0.987652000
C	5.488920000	0.491911000	0.651109000
H	5.473356000	−0.468963000	1.158674000

C	2.742538000	−0.719521000	1.847555000
C	2.434891000	0.061760000	2.970705000
H	2.194958000	1.117257000	2.854721000
C	2.455967000	−0.504704000	4.243110000
H	2.225610000	0.107409000	5.110949000
C	2.773599000	−1.854429000	4.400851000
H	2.787197000	−2.296718000	5.393154000
C	3.075660000	−2.634479000	3.285654000
H	3.325859000	−3.684871000	3.407261000
C	3.061701000	−2.071409000	2.009822000
H	3.299413000	−2.681922000	1.143374000
C	2.808664000	−1.235347000	−1.069798000
C	1.608829000	−1.952471000	−1.321040000
C	1.617929000	−2.912943000	−2.352696000
H	0.705362000	−3.467512000	−2.554678000
C	2.763025000	−3.151682000	−3.101357000
H	2.740523000	−3.899303000	−3.890131000
C	3.935359000	−2.430012000	−2.855881000
H	4.829937000	−2.617437000	−3.442647000
C	3.950139000	−1.470239000	−1.850035000
H	4.858193000	−0.906339000	−1.655667000
C	−0.287597000	−2.661695000	0.003503000
C	0.238196000	−3.947942000	0.244356000
H	1.250728000	−4.178450000	−0.070819000
C	−0.511670000	−4.921613000	0.893353000
H	−0.068082000	−5.899037000	1.066767000
C	−1.810115000	−4.660014000	1.335028000
H	−2.388401000	−5.423473000	1.846243000
C	−2.347282000	−3.396652000	1.114656000
H	−3.351669000	−3.166681000	1.461622000
C	−1.608513000	−2.410687000	0.452411000
C	−3.256689000	−0.309104000	1.638926000
C	−2.557335000	0.124868000	2.774891000
H	−1.471947000	0.204049000	2.753199000
C	−3.241740000	0.433507000	3.947536000

H	−2.690248000	0.762990000	4.823969000
C	−4.631951000	0.321343000	3.994983000
H	−5.166736000	0.567404000	4.908140000
C	−5.332270000	−0.109336000	2.869847000
H	−6.414242000	−0.204050000	2.903989000
C	−4.650474000	−0.426896000	1.694675000
H	−5.206964000	−0.767007000	0.826216000
C	−3.489168000	−0.914758000	−1.235529000
C	−4.276436000	0.191134000	−1.595978000
H	−4.226234000	1.108557000	−1.013598000
C	−5.146556000	0.110790000	−2.680922000
H	−5.764726000	0.965905000	−2.941639000
C	−5.227627000	−1.066375000	−3.427600000
H	−5.905371000	−1.127595000	−4.274589000
C	−4.443949000	−2.164595000	−3.077984000
H	−4.511438000	−3.086331000	−3.649716000
C	−3.578495000	−2.093619000	−1.985268000
H	−2.982735000	−2.958442000	−1.707710000
C	−1.302339000	2.496156000	−2.410725000
C	−0.973300000	1.338558000	−3.080468000
H	−0.638291000	3.352874000	−2.385934000
H	−2.329453000	2.686272000	−2.126135000
C	0.370733000	1.008363000	−3.529285000
C	1.513078000	1.625960000	−3.125207000
H	2.484819000	1.286723000	−3.467272000
H	1.496963000	2.557229000	−2.555682000
H	−1.761559000	0.617242000	−3.292259000
Y	0.103283000	0.613473000	−0.840230000
H	0.465811000	0.118371000	−4.150405000

94

P1_cis E = −1812.430133 a.u.

N	0.152756000	−1.849091000	−0.524112000
P	2.615546000	−0.245277000	0.087368000
P	−2.459495000	−0.547962000	0.102813000
Si	−0.286264000	4.723806000	0.392885000

C	−0.367932000	3.024002000	−0.518477000
H	−0.935778000	2.372118000	0.176289000
H	0.691696000	2.701367000	−0.551255000
C	−2.045836000	5.329510000	0.675453000
H	−2.581869000	5.502266000	−0.265094000
H	−2.035948000	6.281759000	1.219516000
H	−2.628992000	4.617497000	1.271903000
C	0.588478000	4.423520000	2.034382000
H	0.038455000	3.711306000	2.661562000
H	0.673997000	5.357606000	2.602518000
H	1.605560000	4.038816000	1.888639000
C	0.689476000	5.925815000	−0.676479000
H	1.707663000	5.566180000	−0.868797000
H	0.776462000	6.897804000	−0.175921000
H	0.205490000	6.101769000	−1.644132000
C	4.267105000	0.515444000	−0.191955000
C	4.408400000	1.449075000	−1.228516000
H	3.555364000	1.702318000	−1.853988000
C	5.642096000	2.048256000	−1.471201000
H	5.744079000	2.766752000	−2.280018000
C	6.740595000	1.731047000	−0.671553000
H	7.701213000	2.204098000	−0.855660000
C	6.603861000	0.809781000	0.365896000
H	7.457196000	0.562168000	0.991305000
C	5.373261000	0.200429000	0.607256000
H	5.275769000	−0.514604000	1.419094000
C	2.672267000	−0.945978000	1.787392000
C	2.372314000	−0.095452000	2.860971000
H	2.115169000	0.946311000	2.677878000
C	2.422296000	−0.573502000	4.168688000
H	2.196340000	0.092221000	4.997329000
C	2.762732000	−1.904429000	4.411758000
H	2.798136000	−2.278320000	5.431267000
C	3.059534000	−2.754049000	3.346692000
H	3.328757000	−3.789790000	3.535046000

C	3.016478000	−2.279298000	2.036325000
H	3.252195000	−2.943445000	1.209543000
C	2.530263000	−1.692336000	−1.065711000
C	1.279489000	−2.353771000	−1.203253000
C	1.195010000	−3.435351000	−2.102975000
H	0.241154000	−3.942919000	−2.219084000
C	2.300525000	−3.842994000	−2.836324000
H	2.207925000	−4.680216000	−3.523210000
C	3.523037000	−3.174470000	−2.710439000
H	4.385405000	−3.493261000	−3.288611000
C	3.630090000	−2.099846000	−1.836695000
H	4.577882000	−1.579359000	−1.733295000
C	−0.674866000	−2.690191000	0.221387000
C	−0.284042000	−3.976890000	0.641679000
H	0.692375000	−4.355672000	0.356419000
C	−1.118804000	−4.757961000	1.431604000
H	−0.780725000	−5.743662000	1.741611000
C	−2.369121000	−4.290783000	1.842063000
H	−3.014491000	−4.902956000	2.464476000
C	−2.770697000	−3.020409000	1.444570000
H	−3.735765000	−2.633708000	1.763309000
C	−1.948427000	−2.226242000	0.637003000
C	−3.168602000	0.229855000	1.621549000
C	−2.265426000	0.635478000	2.616182000
H	−1.196952000	0.465983000	2.488569000
C	−2.726436000	1.217793000	3.793910000
H	−2.018821000	1.516894000	4.562656000
C	−4.095102000	1.411695000	3.987475000
H	−4.455946000	1.869279000	4.904416000
C	−4.996980000	1.009227000	3.004929000
H	−6.064286000	1.147877000	3.154962000
C	−4.539636000	0.417725000	1.826365000
H	−5.254528000	0.100011000	1.073344000
C	−3.871590000	−0.786758000	−1.059430000
C	−4.588811000	0.335100000	−1.505413000

H	−4.353309000	1.323987000	−1.118095000
C	−5.620551000	0.189106000	−2.429195000
H	−6.178797000	1.062123000	−2.756648000
C	−5.934517000	−1.074081000	−2.933689000
H	−6.736627000	−1.186498000	−3.657824000
C	−5.218773000	−2.189210000	−2.503592000
H	−5.461720000	−3.175503000	−2.889624000
C	−4.192979000	−2.050630000	−1.567627000
H	−3.650620000	−2.927738000	−1.227461000
C	−1.030928000	3.090151000	−1.922423000
C	−1.411029000	1.794268000	−2.635471000
H	−0.365431000	3.675559000	−2.572771000
H	−1.948965000	3.678825000	−1.817722000
C	−0.532238000	1.074586000	−3.435688000
C	0.875174000	1.082566000	−3.295797000
H	1.476651000	0.478164000	−3.969808000
H	1.355136000	2.015231000	−2.978849000
H	−2.472917000	1.615982000	−2.779169000
Y	0.059078000	0.303344000	−1.066115000
H	−0.968949000	0.275103000	−4.040238000

94

C1_trans E = −1812.397824 a.u.

N	0.006108000	−0.526254000	1.460594000
P	2.658574000	−0.219107000	0.072195000
P	−2.406243000	−0.804189000	−0.237856000
Si	−1.544799000	4.284373000	0.213334000
C	−0.181693000	3.006019000	0.418767000
H	0.002417000	2.919802000	1.507195000
H	0.740187000	3.453657000	−0.007632000
C	−1.920491000	4.553018000	−1.629322000
H	−1.050039000	4.959009000	−2.160473000
H	−2.737006000	5.274253000	−1.758162000
H	−2.225734000	3.628361000	−2.138475000
C	−3.124405000	3.712051000	1.078861000
H	−3.534939000	2.795123000	0.639665000

H	−3.902247000	4.483404000	1.017918000
H	−2.942630000	3.511054000	2.141546000
C	−1.034070000	5.953666000	0.941454000
H	−0.832266000	5.868989000	2.016395000
H	−1.819481000	6.708772000	0.811269000
H	−0.123435000	6.337185000	0.464669000
C	3.333461000	−1.233943000	−1.315161000
C	2.867467000	−2.540698000	−1.519012000
H	2.198269000	−2.999066000	−0.795848000
C	3.282136000	−3.268364000	−2.634277000
H	2.923758000	−4.284733000	−2.774028000
C	4.162617000	−2.703855000	−3.556276000
H	4.490928000	−3.276324000	−4.419286000
C	4.631183000	−1.404594000	−3.359518000
H	5.329067000	−0.963466000	−4.066299000
C	4.216330000	−0.669851000	−2.249524000
H	4.604281000	0.334393000	−2.096745000
C	4.057635000	0.822137000	0.666657000
C	3.763168000	2.086009000	1.195202000
H	2.738686000	2.451057000	1.206933000
C	4.779693000	2.883716000	1.718833000
H	4.540469000	3.860960000	2.129302000
C	6.097216000	2.428986000	1.710863000
H	6.890124000	3.051824000	2.115634000
C	6.398011000	1.173993000	1.180353000
H	7.424417000	0.817398000	1.171804000
C	5.385147000	0.370954000	0.661478000
H	5.633365000	−0.601329000	0.245144000
C	2.277603000	−1.413278000	1.408951000
C	0.972769000	−1.396026000	1.966084000
C	0.704101000	−2.318665000	2.999857000
H	−0.287027000	−2.346226000	3.440617000
C	1.679174000	−3.192732000	3.460138000
H	1.430677000	−3.886652000	4.259339000
C	2.962653000	−3.196961000	2.908638000

H	3.723622000	−3.879923000	3.273339000
C	3.247031000	−2.306481000	1.881699000
H	4.239678000	−2.298910000	1.439182000
C	−1.166177000	−0.270751000	2.200489000
C	−1.136392000	0.140071000	3.547490000
H	−0.175607000	0.216461000	4.049149000
C	−2.309003000	0.447820000	4.223877000
H	−2.259925000	0.761791000	5.263294000
C	−3.545975000	0.374305000	3.574859000
H	−4.460368000	0.620243000	4.106732000
C	−3.599814000	−0.012251000	2.241274000
H	−4.557477000	−0.063879000	1.731362000
C	−2.426944000	−0.353430000	1.552099000
C	−4.108161000	−0.491892000	−0.861202000
C	−5.130206000	−1.439175000	−0.709896000
H	−4.925548000	−2.388156000	−0.221844000
C	−6.408776000	−1.168941000	−1.193447000
H	−7.196321000	−1.907981000	−1.074322000
C	−6.677061000	0.043173000	−1.830071000
H	−7.674674000	0.248866000	−2.208084000
C	−5.663949000	0.988336000	−1.987244000
H	−5.868393000	1.931521000	−2.486359000
C	−4.382587000	0.719532000	−1.509636000
H	−3.596614000	1.460182000	−1.641563000
C	−2.195564000	−2.630709000	−0.336848000
C	−2.215226000	−3.453564000	0.793607000
H	−2.365009000	−3.021505000	1.778671000
C	−2.051373000	−4.831971000	0.655027000
H	−2.070812000	−5.466636000	1.536801000
C	−1.871017000	−5.394598000	−0.607430000
H	−1.748479000	−6.469181000	−0.711552000
C	−1.854931000	−4.577539000	−1.738923000
H	−1.724061000	−5.013257000	−2.725840000
C	−2.011202000	−3.200239000	−1.605127000
H	−2.011534000	−2.571434000	−2.493488000

C	2.402884000	3.167653000	−3.243530000
C	1.192137000	2.585667000	−3.231952000
H	2.513864000	4.246850000	−3.287898000
H	3.318316000	2.580800000	−3.223879000
H	0.293380000	3.200120000	−3.267776000
C	1.014024000	1.142840000	−3.198608000
C	−0.187079000	0.514565000	−3.182542000
H	−0.253562000	−0.569657000	−3.233257000
H	−1.121854000	1.069619000	−3.272738000
Y	−0.026644000	0.817102000	−0.326422000
H	1.922065000	0.537589000	−3.197942000

94

TS1_trans E = −1812.381695 a.u. Imaginary frequency=198i

N	0.351851000	−1.715543000	0.019993000
P	2.749319000	0.049122000	0.117610000
P	−2.415287000	−0.637065000	0.122095000
Si	−1.258721000	4.326512000	−0.041166000
C	−0.496704000	2.606120000	−0.303175000
H	−0.820220000	2.029762000	0.593343000
H	0.605012000	2.743869000	−0.240188000
C	−3.136692000	4.253326000	−0.204916000
H	−3.469964000	4.025636000	−1.224323000
H	−3.571018000	5.224756000	0.062114000
H	−3.569971000	3.504496000	0.468221000
C	−0.815941000	4.901843000	1.702651000
H	−1.221285000	4.225932000	2.465216000
H	−1.225787000	5.900634000	1.898690000
H	0.269782000	4.960365000	1.847432000
C	−0.535426000	5.552540000	−1.283916000
H	0.561681000	5.528944000	−1.290591000
H	−0.832187000	6.572314000	−1.009573000
H	−0.885336000	5.386516000	−2.309449000
C	4.347399000	0.779681000	−0.430830000
C	4.408665000	1.402744000	−1.684996000
H	3.521393000	1.450701000	−2.312912000

C	5.601868000	1.959039000	−2.139471000
H	5.639796000	2.438241000	−3.114074000
C	6.742827000	1.908309000	−1.338193000
H	7.672469000	2.348279000	−1.688261000
C	6.687237000	1.297955000	−0.085901000
H	7.573413000	1.259772000	0.541659000
C	5.495976000	0.734349000	0.369450000
H	5.461212000	0.264429000	1.348290000
C	2.860201000	−0.064041000	1.950598000
C	2.530143000	1.070667000	2.705145000
H	2.219225000	1.987560000	2.207749000
C	2.613708000	1.035429000	4.095442000
H	2.362890000	1.919939000	4.674754000
C	3.018178000	−0.133946000	4.740332000
H	3.080098000	−0.162363000	5.824706000
C	3.344438000	−1.265398000	3.993405000
H	3.662812000	−2.175645000	4.494292000
C	3.266812000	−1.234248000	2.601487000
H	3.523811000	−2.116959000	2.022369000
C	2.743692000	−1.680113000	−0.518475000
C	1.500063000	−2.362673000	−0.480933000
C	1.450972000	−3.662509000	−1.023371000
H	0.506890000	−4.200243000	−1.004115000
C	2.578814000	−4.253254000	−1.578828000
H	2.507174000	−5.255900000	−1.992659000
C	3.795640000	−3.566102000	−1.621150000
H	4.676132000	−4.030311000	−2.055697000
C	3.870211000	−2.280075000	−1.097312000
H	4.811119000	−1.737165000	−1.125452000
C	−0.446465000	−2.409452000	0.934799000
C	0.038252000	−3.469486000	1.729629000
H	1.072696000	−3.780300000	1.624808000
C	−0.777165000	−4.110828000	2.653567000
H	−0.361797000	−4.918879000	3.250791000
C	−2.107280000	−3.724986000	2.834880000

H	−2.738960000	−4.227469000	3.560895000
C	−2.605058000	−2.674552000	2.073072000
H	−3.633015000	−2.346770000	2.206955000
C	−1.796932000	−2.025859000	1.131568000
C	−3.599449000	0.253149000	1.221775000
C	−4.976428000	0.302674000	0.981529000
H	−5.402101000	−0.207345000	0.122607000
C	−5.814455000	0.998711000	1.854337000
H	−6.883327000	1.027741000	1.660863000
C	−5.288033000	1.643701000	2.971116000
H	−5.943907000	2.181807000	3.649794000
C	−3.915354000	1.590276000	3.220506000
H	−3.500007000	2.081223000	4.096518000
C	−3.074241000	0.903567000	2.349230000
H	−2.008578000	0.852893000	2.564960000
C	−3.427377000	−1.375744000	−1.234921000
C	−3.568579000	−2.760879000	−1.378578000
H	−3.120528000	−3.429160000	−0.648994000
C	−4.293170000	−3.282688000	−2.450716000
H	−4.401095000	−4.359272000	−2.551696000
C	−4.884327000	−2.430938000	−3.382367000
H	−5.451763000	−2.841267000	−4.213046000
C	−4.750080000	−1.048402000	−3.244164000
H	−5.215485000	−0.379542000	−3.963241000
C	−4.018066000	−0.522870000	−2.181670000
H	−3.926535000	0.556682000	−2.076577000
C	−1.058553000	2.301725000	−2.554408000
C	−0.756971000	1.072581000	−3.141571000
H	−2.090866000	2.568610000	−2.364134000
H	−0.385254000	3.140426000	−2.703814000
H	−1.533961000	0.309218000	−3.233067000
C	0.595972000	0.728537000	−3.522825000
C	1.102080000	−0.534797000	−3.542002000
H	2.140615000	−0.728172000	−3.789505000
H	0.461614000	−1.411586000	−3.433047000

Y	0.103149000	0.358285000	−0.935030000
H	1.286528000	1.562777000	−3.674612000

94

P1_trans E = −1812.423255 a.u.

N	0.178016000	−2.037957000	−0.156410000
P	2.595674000	−0.307223000	0.090806000
P	−2.422291000	−0.670845000	0.133486000
Si	−0.348469000	5.181678000	0.236582000
C	−0.505671000	3.440409000	−0.547756000
H	−1.281066000	2.896108000	0.016918000
H	0.455339000	2.922742000	−0.358250000
C	−2.012323000	6.061095000	0.127047000
H	−2.333695000	6.204563000	−0.911452000
H	−1.956390000	7.054546000	0.588366000
H	−2.799515000	5.501666000	0.646940000
C	0.149202000	4.948783000	2.042770000
H	−0.590856000	4.352521000	2.590237000
H	0.232569000	5.915523000	2.553775000
H	1.122109000	4.449517000	2.133442000
C	0.980575000	6.156499000	−0.679951000
H	1.949198000	5.641787000	−0.656860000
H	1.123943000	7.141808000	−0.220136000
H	0.714962000	6.324986000	−1.730297000
C	4.198709000	0.446460000	−0.397927000
C	4.245594000	1.215765000	−1.569114000
H	3.346551000	1.363225000	−2.165243000
C	5.442281000	1.795727000	−1.982650000
H	5.470815000	2.387509000	−2.893465000
C	6.597995000	1.624557000	−1.220024000
H	7.530261000	2.083586000	−1.537043000
C	6.555223000	0.870014000	−0.048115000
H	7.453500000	0.739055000	0.548906000
C	5.361606000	0.279194000	0.364190000
H	5.335244000	−0.305766000	1.279226000
C	2.713156000	−0.664880000	1.887461000

C	2.388434000	0.361511000	2.785114000
H	2.069819000	1.333484000	2.413248000
C	2.486251000	0.145175000	4.157776000
H	2.239611000	0.945271000	4.850352000
C	2.898302000	−1.097497000	4.640082000
H	2.970582000	−1.267154000	5.710779000
C	3.217740000	−2.121728000	3.749263000
H	3.540975000	−3.088931000	4.124355000
C	3.127329000	−1.909776000	2.374135000
H	3.379522000	−2.709082000	1.682806000
C	2.527955000	−1.935595000	−0.786059000
C	1.291988000	−2.642723000	−0.760518000
C	1.219370000	−3.876045000	−1.442170000
H	0.279530000	−4.420928000	−1.434079000
C	2.316361000	−4.379375000	−2.124893000
H	2.232343000	−5.330622000	−2.643941000
C	3.519756000	−3.664871000	−2.168837000
H	4.373841000	−4.061436000	−2.709859000
C	3.617981000	−2.446462000	−1.510926000
H	4.551967000	−1.892199000	−1.535856000
C	−0.667724000	−2.749695000	0.692278000
C	−0.293800000	−3.944133000	1.338036000
H	0.690952000	−4.362692000	1.154460000
C	−1.158504000	−4.578837000	2.222091000
H	−0.835330000	−5.495311000	2.709488000
C	−2.421083000	−4.051539000	2.502292000
H	−3.088713000	−4.550264000	3.198100000
C	−2.807401000	−2.869353000	1.879697000
H	−3.781693000	−2.437036000	2.094260000
C	−1.953215000	−2.225155000	0.978656000
C	−3.292158000	0.342710000	1.403228000
C	−4.683326000	0.486422000	1.446684000
H	−5.308317000	−0.000455000	0.703897000
C	−5.274390000	1.254864000	2.450392000
H	−6.355538000	1.360147000	2.477924000

C	−4.486315000	1.876966000	3.416490000
H	−4.951045000	2.471898000	4.197819000
C	−3.098737000	1.730394000	3.381938000
H	−2.480730000	2.205687000	4.138970000
C	−2.503008000	0.972796000	2.377104000
H	−1.421096000	0.852707000	2.366706000
C	−3.685351000	−1.122994000	−1.133235000
C	−3.946913000	−2.459403000	−1.458841000
H	−3.460664000	−3.258114000	−0.906434000
C	−4.842231000	−2.766953000	−2.483554000
H	−5.042804000	−3.807114000	−2.725681000
C	−5.483581000	−1.749152000	−3.187161000
H	−6.183187000	−1.992993000	−3.981758000
C	−5.229072000	−0.414845000	−2.866210000
H	−5.731248000	0.382885000	−3.406727000
C	−4.328890000	−0.101158000	−1.850912000
H	−4.147640000	0.942516000	−1.602060000
C	−0.825022000	3.400651000	−2.056582000
C	−0.976949000	1.992623000	−2.572010000
H	−1.740688000	3.974878000	−2.250581000
H	−0.023903000	3.905008000	−2.612889000
H	−1.947516000	1.510794000	−2.400766000
C	−0.064475000	1.343671000	−3.374822000
C	−0.025476000	−0.070606000	−3.595057000
H	0.714253000	−0.461863000	−4.290328000
H	−0.993546000	−0.587090000	−3.665632000
Y	0.080374000	−0.045408000	−1.176125000
H	0.833237000	1.907712000	−3.650819000

94

C1_1,2 E = −1812.389632 a.u.

N	−0.081867000	0.236645000	1.557394000
P	2.361045000	0.501135000	−0.038335000
P	−2.350677000	0.401884000	−0.432423000
Si	1.017611000	−3.734321000	−1.953320000
C	1.232148000	−3.335035000	−0.142105000

H	2.291709000	−3.211695000	0.127712000
H	0.859722000	−4.165791000	0.473091000
C	0.026960000	−2.251437000	−2.741094000
H	−1.006261000	−2.100525000	−2.371509000
H	−0.116190000	−2.480643000	−3.804650000
H	0.559790000	−1.281672000	−2.749854000
C	2.605274000	−3.839302000	−2.964599000
H	3.199293000	−2.919835000	−2.906235000
H	2.389063000	−4.028606000	−4.023217000
H	3.231784000	−4.663413000	−2.602410000
C	−0.044914000	−5.262339000	−2.266337000
H	0.479568000	−6.160269000	−1.917536000
H	−0.249490000	−5.394225000	−3.335782000
H	−1.011220000	−5.231464000	−1.748339000
C	4.104723000	0.227823000	−0.573721000
C	4.528187000	−1.079895000	−0.851075000
H	3.838005000	−1.911709000	−0.741051000
C	5.836751000	−1.325531000	−1.259692000
H	6.154813000	−2.343780000	−1.466444000
C	6.731327000	−0.265645000	−1.410913000
H	7.750131000	−0.455830000	−1.737014000
C	6.314076000	1.037398000	−1.146067000
H	7.006628000	1.866440000	−1.263422000
C	5.007553000	1.287434000	−0.726842000
H	4.695870000	2.306977000	−0.521592000
C	2.137829000	2.325564000	−0.072541000
C	1.719107000	2.921939000	−1.268542000
H	1.486659000	2.308217000	−2.135895000
C	1.605688000	4.308129000	−1.355128000
H	1.285577000	4.763986000	−2.287464000
C	1.896514000	5.103443000	−0.247124000
H	1.806645000	6.184255000	−0.314981000
C	2.303883000	4.511992000	0.948693000
H	2.531833000	5.129991000	1.812964000
C	2.428259000	3.126672000	1.038454000

H	2.757400000	2.670570000	1.967908000
C	2.326546000	0.019182000	1.749195000
C	1.046107000	−0.032552000	2.360545000
C	0.964027000	−0.450897000	3.702593000
H	−0.012826000	−0.514124000	4.172515000
C	2.110095000	−0.787817000	4.412429000
H	2.024413000	−1.100632000	5.449940000
C	3.366448000	−0.741116000	3.801033000
H	4.258763000	−1.007502000	4.360102000
C	3.469803000	−0.351141000	2.470317000
H	4.442865000	−0.316012000	1.989100000
C	−1.056436000	1.151670000	1.942364000
C	−0.933889000	1.970013000	3.085804000
H	−0.036056000	1.903223000	3.691199000
C	−1.935703000	2.858809000	3.452212000
H	−1.800701000	3.466078000	4.343880000
C	−3.103913000	2.979862000	2.697074000
H	−3.892192000	3.664776000	2.993972000
C	−3.232040000	2.212575000	1.545176000
H	−4.130326000	2.301492000	0.938906000
C	−2.227488000	1.317728000	1.149902000
C	−2.075375000	1.643815000	−1.779943000
C	−1.756404000	1.165997000	−3.060919000
H	−1.666259000	0.096587000	−3.240664000
C	−1.573665000	2.050096000	−4.124014000
H	−1.339159000	1.665549000	−5.113101000
C	−1.696681000	3.422992000	−3.914482000
H	−1.559266000	4.114235000	−4.741592000
C	−1.996653000	3.905709000	−2.640470000
H	−2.091541000	4.975085000	−2.471996000
C	−2.181635000	3.024726000	−1.576248000
H	−2.404825000	3.414621000	−0.588287000
C	−4.131517000	−0.073795000	−0.600275000
C	−4.754926000	−0.105674000	−1.853927000
H	−4.228446000	0.240976000	−2.738468000

C	−6.068527000	−0.556889000	−1.974297000
H	−6.543726000	−0.566359000	−2.951489000
C	−6.773243000	−0.982677000	−0.849416000
H	−7.797924000	−1.330227000	−0.946414000
C	−6.160616000	−0.949351000	0.402595000
H	−6.707804000	−1.264719000	1.287014000
C	−4.846818000	−0.500392000	0.528475000
H	−4.388825000	−0.449475000	1.512709000
C	−2.108326000	−3.083410000	0.657507000
C	−1.648509000	−3.223140000	1.922862000
H	−3.051661000	−2.585148000	0.445232000
H	−1.634635000	−3.627733000	−0.159030000
H	−0.736995000	−3.796882000	2.093077000
C	−2.309018000	−2.702289000	3.106696000
C	−1.875485000	−2.959047000	4.350247000
H	−2.406689000	−2.595403000	5.224912000
H	−0.984798000	−3.557270000	4.530275000
Y	0.064468000	−1.259069000	−0.090580000
H	−3.213135000	−2.114910000	2.956236000

94

TS1_1,2 E = −1812.367425 a.u. Imaginary frequency=312i

N	0.110855000	−0.680584000	1.428267000
P	−2.269805000	−0.715313000	−0.272350000
P	2.630072000	−0.169322000	−0.093136000
Si	−0.690959000	3.504952000	−2.018697000
C	−1.454269000	3.082082000	−0.342885000
H	−2.188871000	2.252767000	−0.362689000
H	−2.063803000	3.938470000	−0.048925000
C	0.626152000	2.177522000	−2.463250000
H	1.529579000	2.162185000	−1.827718000
H	1.018796000	2.451626000	−3.450730000
H	0.260681000	1.145908000	−2.608099000
C	−1.937130000	3.532811000	−3.430406000
H	−2.397662000	2.552185000	−3.597299000
H	−1.454706000	3.836893000	−4.367956000

H	−2.741227000	4.249461000	−3.225982000
C	0.218595000	5.146485000	−1.894096000
H	−0.465625000	5.957810000	−1.617910000
H	0.668466000	5.415748000	−2.857269000
H	1.024421000	5.122732000	−1.150687000
C	−4.027788000	−0.501968000	−0.797136000
C	−4.391819000	0.629378000	−1.537743000
H	−3.644302000	1.372432000	−1.801232000
C	−5.711003000	0.809273000	−1.952515000
H	−5.981370000	1.690861000	−2.527432000
C	−6.675650000	−0.146086000	−1.639076000
H	−7.702626000	−0.010273000	−1.966855000
C	−6.319272000	−1.283024000	−0.912266000
H	−7.067296000	−2.034438000	−0.674329000
C	−5.003178000	−1.463335000	−0.493729000
H	−4.732407000	−2.358679000	0.059312000
C	−1.975351000	−2.525839000	−0.455087000
C	−1.635254000	−3.001038000	−1.728314000
H	−1.520066000	−2.307219000	−2.558510000
C	−1.455881000	−4.366188000	−1.941457000
H	−1.203116000	−4.728141000	−2.934394000
C	−1.601776000	−5.263687000	−0.883583000
H	−1.461366000	−6.328285000	−1.049905000
C	−1.930693000	−4.793596000	0.387296000
H	−2.046518000	−5.490468000	1.213091000
C	−2.119950000	−3.429088000	0.603459000
H	−2.384405000	−3.069324000	1.594026000
C	−2.285893000	−0.398652000	1.553515000
C	−1.028051000	−0.479393000	2.215361000
C	−0.990650000	−0.243917000	3.608346000
H	−0.036890000	−0.304379000	4.125136000
C	−2.149347000	0.059381000	4.309140000
H	−2.096017000	0.228318000	5.381682000
C	−3.378197000	0.166078000	3.645678000
H	−4.280968000	0.408790000	4.198686000

C	−3.439927000	−0.053216000	2.274907000
H	−4.393478000	0.013558000	1.759333000
C	1.139662000	−1.524098000	1.833493000
C	0.970781000	−2.536084000	2.802471000
H	−0.000537000	−2.668981000	3.268428000
C	2.021357000	−3.369074000	3.163922000
H	1.851733000	−4.134520000	3.917212000
C	3.281768000	−3.240129000	2.574944000
H	4.102222000	−3.887240000	2.869319000
C	3.464757000	−2.267862000	1.599288000
H	4.436872000	−2.151929000	1.126144000
C	2.415071000	−1.421769000	1.221660000
C	2.742430000	−1.115095000	−1.678897000
C	3.084471000	−0.434356000	−2.859421000
H	3.347379000	0.620703000	−2.826886000
C	3.126604000	−1.109579000	−4.076668000
H	3.411822000	−0.576418000	−4.979786000
C	2.811502000	−2.468303000	−4.133755000
H	2.846993000	−2.995438000	−5.083249000
C	2.460142000	−3.146643000	−2.968558000
H	2.220198000	−4.205781000	−3.004812000
C	2.424704000	−2.477183000	−1.744704000
H	2.159101000	−3.017900000	−0.841470000
C	4.308162000	0.551413000	0.170646000
C	5.426982000	0.174143000	−0.581377000
H	5.332572000	−0.560449000	−1.375461000
C	6.673586000	0.739040000	−0.310959000
H	7.536668000	0.438619000	−0.898944000
C	6.814662000	1.676876000	0.709977000
H	7.787446000	2.113923000	0.917598000
C	5.704557000	2.049680000	1.468654000
H	5.809613000	2.774488000	2.271450000
C	4.456805000	1.493807000	1.198761000
H	3.600751000	1.779630000	1.804685000
C	0.743017000	2.697496000	1.770263000

C	−0.372431000	3.544294000	1.486626000
H	0.722239000	2.154459000	2.716473000
H	1.725425000	3.087892000	1.497196000
H	−0.158987000	4.499267000	1.011921000
C	−1.509198000	3.565850000	2.424265000
C	−2.214304000	4.666520000	2.707417000
H	−3.003301000	4.658181000	3.453668000
H	−2.013342000	5.619631000	2.221209000
Y	0.025956000	1.138366000	0.092475000
H	−1.725463000	2.633782000	2.946341000

94

P1_1,2 E = −1812.398652 a.u.

N	0.226874000	−1.479632000	−1.090461000
P	2.685388000	−0.255186000	0.053170000
P	−2.433014000	−0.729849000	0.109226000
Si	−0.287520000	4.138555000	1.247907000
C	0.080107000	4.023083000	−0.611340000
H	1.121561000	3.679149000	−0.725132000
H	0.081359000	5.040727000	−1.025419000
C	−0.198404000	2.354382000	1.944675000
H	−1.039321000	1.702506000	1.644116000
H	−0.284353000	2.379215000	3.038213000
H	0.779994000	1.876067000	1.759832000
C	1.048481000	5.149497000	2.101188000
H	2.047068000	4.723518000	1.945923000
H	0.875133000	5.207372000	3.182371000
H	1.064879000	6.176665000	1.717604000
C	−2.001518000	4.831545000	1.582010000
H	−2.056399000	5.880189000	1.266754000
H	−2.245793000	4.794786000	2.650382000
H	−2.781367000	4.289279000	1.035841000
C	4.223216000	0.755652000	0.016390000
C	4.226102000	1.933969000	−0.746007000
H	3.349926000	2.212217000	−1.329386000
C	5.357876000	2.743714000	−0.782127000

H	5.355117000	3.649200000	−1.382995000
C	6.491096000	2.393185000	−0.045947000
H	7.372202000	3.028369000	−0.069797000
C	6.490640000	1.227936000	0.717973000
H	7.371778000	0.950219000	1.289878000
C	5.362616000	0.407393000	0.749912000
H	5.373450000	−0.501653000	1.343997000
C	2.980063000	−1.612259000	1.255379000
C	2.707534000	−1.373170000	2.609569000
H	2.327702000	−0.404867000	2.929733000
C	2.934871000	−2.370410000	3.555689000
H	2.728472000	−2.177236000	4.604893000
C	3.423988000	−3.613615000	3.153893000
H	3.597611000	−4.392719000	3.891058000
C	3.691251000	−3.856737000	1.806749000
H	4.074305000	−4.824038000	1.493239000
C	3.472396000	−2.860346000	0.856703000
H	3.684459000	−3.052504000	−0.191351000
C	2.567797000	−1.027311000	−1.619263000
C	1.311233000	−1.578198000	−1.985236000
C	1.180126000	−2.130769000	−3.274197000
H	0.220005000	−2.544522000	−3.569885000
C	2.250514000	−2.141154000	−4.157927000
H	2.121649000	−2.572254000	−5.147315000
C	3.482276000	−1.587168000	−3.793462000
H	4.316346000	−1.596233000	−4.488965000
C	3.632359000	−1.023026000	−2.532726000
H	4.584429000	−0.587538000	−2.243091000
C	−0.572079000	−2.594595000	−0.829117000
C	−0.140644000	−3.914737000	−1.076187000
H	0.849024000	−4.079620000	−1.489968000
C	−0.954211000	−5.003871000	−0.794196000
H	−0.584693000	−6.005323000	−1.000284000
C	−2.229220000	−4.830937000	−0.249646000
H	−2.865464000	−5.685137000	−0.039679000

C	−2.666817000	−3.541147000	0.022220000
H	−3.654482000	−3.386588000	0.449528000
C	−1.857207000	−2.431361000	−0.250798000
C	−2.847237000	−0.694756000	1.906818000
C	−3.647821000	0.344247000	2.408219000
H	−4.104390000	1.060296000	1.728561000
C	−3.888512000	0.448406000	3.776351000
H	−4.524929000	1.245620000	4.151193000
C	−3.321656000	−0.471180000	4.660336000
H	−3.513578000	−0.390555000	5.726715000
C	−2.517513000	−1.498108000	4.169300000
H	−2.081452000	−2.223202000	4.851302000
C	−2.278792000	−1.613096000	2.799376000
H	−1.664970000	−2.427734000	2.425728000
C	−4.028955000	−0.516443000	−0.783712000
C	−5.258581000	−0.901449000	−0.233331000
H	−5.308571000	−1.292073000	0.779246000
C	−6.427172000	−0.774911000	−0.981977000
H	−7.377545000	−1.075278000	−0.549174000
C	−6.378401000	−0.268112000	−2.280576000
H	−7.291998000	−0.171398000	−2.860606000
C	−5.157454000	0.117213000	−2.832913000
H	−5.114699000	0.516218000	−3.842536000
C	−3.986717000	−0.001891000	−2.086455000
H	−3.039512000	0.302532000	−2.526132000
C	−0.064360000	2.106084000	−2.286205000
C	−0.870218000	3.124193000	−1.456751000
H	0.804932000	2.574889000	−2.761794000
H	−0.685097000	1.664576000	−3.082530000
H	−1.550774000	2.559199000	−0.751662000
C	−1.830183000	3.938647000	−2.283877000
C	−3.161239000	3.889810000	−2.214243000
H	−3.787559000	4.491237000	−2.867196000
H	−3.677259000	3.241018000	−1.507938000
Y	0.034715000	0.651145000	−0.490860000

H	−1.347344000	4.594765000	−3.010361000

94

C1_cis_1,2 E = −1812.396139 a.u.

N	0.173309000	−1.527969000	−0.441824000
P	2.693264000	−0.117975000	0.173611000
P	−2.535345000	−0.380107000	0.147538000
Si	−0.520700000	4.155110000	0.600950000
C	−0.259015000	2.346381000	1.023041000
H	−1.087264000	2.050106000	1.686669000
H	0.656925000	2.292770000	1.642334000
C	−1.988510000	4.340576000	−0.591984000
H	−1.809674000	3.856182000	−1.562631000
H	−2.202710000	5.396466000	−0.798357000
H	−2.897982000	3.901353000	−0.162206000
C	−0.879020000	5.207544000	2.129036000
H	−1.787635000	4.865340000	2.639354000
H	−1.019383000	6.264759000	1.871116000
H	−0.054482000	5.147528000	2.849931000
C	1.030272000	4.858065000	−0.241102000
H	1.899666000	4.792378000	0.425628000
H	0.896255000	5.916479000	−0.496457000
H	1.287998000	4.332757000	−1.171031000
C	4.358739000	0.629435000	−0.062240000
C	4.450405000	1.941318000	−0.544697000
H	3.546322000	2.506558000	−0.759318000
C	5.696123000	2.542800000	−0.721836000
H	5.757023000	3.563417000	−1.089892000
C	6.858391000	1.839781000	−0.409996000
H	7.829387000	2.308964000	−0.542185000
C	6.774716000	0.539009000	0.089210000
H	7.679378000	−0.004501000	0.347686000
C	5.532150000	−0.064128000	0.268287000
H	5.475012000	−1.068556000	0.679390000
C	2.786479000	−0.972687000	1.802469000
C	2.525501000	−0.216961000	2.953524000

H	2.256879000	0.833700000	2.869492000
C	2.615824000	−0.806895000	4.212442000
H	2.416387000	−0.214390000	5.101211000
C	2.959580000	−2.153739000	4.329702000
H	3.027789000	−2.613673000	5.311704000
C	3.216742000	−2.909462000	3.186476000
H	3.487574000	−3.958096000	3.276037000
C	3.130525000	−2.323974000	1.923985000
H	3.332786000	−2.918229000	1.036871000
C	2.533632000	−1.456717000	−1.088146000
C	1.239008000	−2.026133000	−1.218595000
C	1.051802000	−3.022907000	−2.198066000
H	0.070217000	−3.478981000	−2.297712000
C	2.096603000	−3.424446000	−3.022631000
H	1.922485000	−4.196164000	−3.768118000
C	3.359224000	−2.836343000	−2.907505000
H	4.172871000	−3.146912000	−3.556363000
C	3.569672000	−1.853701000	−1.944469000
H	4.551097000	−1.398475000	−1.844213000
C	−0.655999000	−2.439765000	0.221822000
C	−0.225779000	−3.733745000	0.579364000
H	0.780140000	−4.048108000	0.320078000
C	−1.056774000	−4.603320000	1.274776000
H	−0.685853000	−5.590380000	1.539335000
C	−2.345641000	−4.219757000	1.650349000
H	−2.989576000	−4.898867000	2.200748000
C	−2.788320000	−2.944728000	1.314992000
H	−3.783060000	−2.622071000	1.612418000
C	−1.969212000	−2.062634000	0.600207000
C	−3.516302000	0.174648000	1.611042000
C	−2.848992000	0.285220000	2.840715000
H	−1.797974000	0.017404000	2.920272000
C	−3.533028000	0.714304000	3.974609000
H	−3.007874000	0.789312000	4.922997000
C	−4.886547000	1.044973000	3.893720000

H	−5.418929000	1.381947000	4.778838000
C	−5.553677000	0.934580000	2.676061000
H	−6.610092000	1.180071000	2.607976000
C	−4.874746000	0.499859000	1.536845000
H	−5.413044000	0.405618000	0.598687000
C	−3.766066000	−0.585858000	−1.216121000
C	−4.290242000	0.572864000	−1.812920000
H	−4.013098000	1.555072000	−1.433606000
C	−5.182935000	0.476251000	−2.878670000
H	−5.594921000	1.378931000	−3.322273000
C	−5.546413000	−0.777674000	−3.373369000
H	−6.238524000	−0.853739000	−4.207416000
C	−5.022014000	−1.930997000	−2.791860000
H	−5.306567000	−2.909195000	−3.170297000
C	−4.137715000	−1.839278000	−1.715511000
H	−3.740847000	−2.743343000	−1.263032000
C	−0.972024000	−0.046677000	−3.333664000
C	−0.819492000	1.296651000	−3.286486000
H	−0.133701000	−0.740940000	−3.381994000
H	−1.964138000	−0.484708000	−3.377550000
C	0.454723000	2.023185000	−3.195670000
C	1.687262000	1.474204000	−3.108655000
H	2.568935000	2.103479000	−3.041157000
H	1.869832000	0.402185000	−3.180468000
H	−1.715515000	1.915336000	−3.301279000
Y	0.058209000	0.735465000	−0.642261000
H	0.372099000	3.108811000	−3.171683000

94

TS1_cis_1,2 E = −1812.366083 a.u. Imaginary frequency=318i

N	−0.084449000	0.361231000	1.556888000
P	2.260376000	0.669251000	−0.187318000
P	−2.481817000	0.294061000	−0.240656000
Si	1.000762000	−3.428765000	−2.125626000
C	1.316770000	−3.302051000	−0.256013000
H	2.072885000	−2.555195000	0.066894000

H	1.785443000	−4.250300000	0.019549000
C	−0.202303000	−2.026612000	−2.658605000
H	−1.205672000	−2.066792000	−2.197736000
H	−0.401022000	−2.185060000	−3.726402000
H	0.194746000	−0.997157000	−2.617186000
C	2.556300000	−3.256771000	−3.167338000
H	3.040262000	−2.282669000	−3.036538000
H	2.320860000	−3.374168000	−4.232685000
H	3.287478000	−4.031121000	−2.906368000
C	0.125800000	−5.054203000	−2.482344000
H	0.724139000	−5.909068000	−2.145053000
H	−0.028679000	−5.173203000	−3.561596000
H	−0.857026000	−5.122587000	−2.001812000
C	4.012338000	0.535611000	−0.765447000
C	4.565996000	−0.737844000	−0.970679000
H	3.981772000	−1.630477000	−0.759407000
C	5.871774000	−0.877406000	−1.434911000
H	6.289734000	−1.870023000	−1.580220000
C	6.636793000	0.254383000	−1.718148000
H	7.653189000	0.146853000	−2.086623000
C	6.091045000	1.522109000	−1.528578000
H	6.681016000	2.407984000	−1.747715000
C	4.787770000	1.665701000	−1.052254000
H	4.378433000	2.660243000	−0.904926000
C	1.924540000	2.479250000	−0.159881000
C	1.337138000	3.061279000	−1.289703000
H	1.048191000	2.442227000	−2.136196000
C	1.122715000	4.437907000	−1.336717000
H	0.670854000	4.882018000	−2.219004000
C	1.481228000	5.237478000	−0.252258000
H	1.311967000	6.310315000	−0.287974000
C	2.057712000	4.660276000	0.879646000
H	2.338268000	5.281872000	1.725748000
C	2.283244000	3.285934000	0.927285000
H	2.742286000	2.841783000	1.806389000

C	2.333373000	0.182707000	1.603323000
C	1.091210000	0.132365000	2.293677000
C	1.096631000	−0.244739000	3.652270000
H	0.151511000	−0.296445000	4.185086000
C	2.285234000	−0.547106000	4.302900000
H	2.264610000	−0.825900000	5.353301000
C	3.501957000	−0.507960000	3.614771000
H	4.430128000	−0.744001000	4.127348000
C	3.520996000	−0.154226000	2.270220000
H	4.466240000	−0.111270000	1.736737000
C	−1.095547000	1.200146000	2.012946000
C	−0.956881000	2.049328000	3.131109000
H	−0.010819000	2.080741000	3.661631000
C	−2.007125000	2.848789000	3.563926000
H	−1.860112000	3.484756000	4.433443000
C	−3.239211000	2.843717000	2.906259000
H	−4.062885000	3.455625000	3.260812000
C	−3.385736000	2.046932000	1.776557000
H	−4.331449000	2.037667000	1.239674000
C	−2.332287000	1.247860000	1.314215000
C	−2.481554000	1.531874000	−1.616844000
C	−2.375212000	1.059466000	−2.934903000
H	−2.306301000	−0.008773000	−3.129012000
C	−2.381945000	1.947025000	−4.009534000
H	−2.312607000	1.567166000	−5.025472000
C	−2.482207000	3.319017000	−3.778060000
H	−2.490475000	4.012709000	−4.614422000
C	−2.574062000	3.795993000	−2.471261000
H	−2.653599000	4.863759000	−2.285423000
C	−2.570845000	2.910696000	−1.393569000
H	−2.636363000	3.295079000	−0.380678000
C	−4.192284000	−0.406792000	−0.220441000
C	−5.073547000	−0.276997000	−1.299465000
H	−4.792041000	0.303138000	−2.173213000
C	−6.332759000	−0.876237000	−1.250748000

H	−7.012622000	−0.761585000	−2.090735000
C	−6.722019000	−1.606663000	−0.130117000
H	−7.704666000	−2.068799000	−0.094459000
C	−5.849078000	−1.733892000	0.951157000
H	−6.150054000	−2.291817000	1.833834000
C	−4.590234000	−1.140880000	0.906937000
H	−3.925435000	−1.230134000	1.762047000
C	−1.436786000	−2.969482000	1.007519000
C	−0.310617000	−3.849494000	1.011312000
H	−1.719598000	−2.514137000	1.957813000
H	−2.286950000	−3.253985000	0.386862000
H	−0.386728000	−4.714912000	0.355671000
C	0.472267000	−4.117232000	2.244183000
C	0.107480000	−3.777339000	3.485238000
H	0.725203000	−4.047054000	4.336802000
H	−0.816688000	−3.250399000	3.704248000
Y	−0.052039000	−1.270645000	0.027885000
H	1.395754000	−4.678817000	2.113801000

94

P1_cis_1,2 E = −1812.405394 a.u.

N	−0.299220000	−0.113540000	1.399706000
P	1.811456000	−1.529550000	−0.154229000
P	−2.757965000	0.360522000	−0.209309000
Si	3.788911000	3.923611000	0.186407000
C	2.871254000	2.417046000	−0.568595000
H	2.424461000	1.852777000	0.272581000
H	3.657971000	1.765080000	−0.977044000
C	4.705883000	4.802679000	−1.208340000
H	5.410619000	4.134072000	−1.717440000
H	5.283318000	5.647698000	−0.814104000
H	4.021925000	5.206605000	−1.964578000
C	2.609227000	5.121176000	1.039229000
H	1.902663000	5.577109000	0.336469000
H	3.176394000	5.935744000	1.506852000
H	2.029244000	4.630939000	1.830479000

C	5.014615000	3.236936000	1.445939000
H	4.504822000	2.705308000	2.259197000
H	5.596967000	4.046449000	1.902082000
H	5.726923000	2.543416000	0.982962000
C	0.970400000	−2.757801000	−1.252370000
C	0.151761000	−3.779653000	−0.755529000
H	0.022545000	−3.897157000	0.316268000
C	−0.489622000	−4.651731000	−1.635643000
H	−1.114052000	−5.447703000	−1.239244000
C	−0.324726000	−4.512553000	−3.013106000
H	−0.819317000	−5.200090000	−3.693840000
C	0.483023000	−3.491996000	−3.517492000
H	0.622104000	−3.384564000	−4.590017000
C	1.120622000	−2.613865000	−2.643334000
H	1.766213000	−1.833917000	−3.043645000
C	3.609854000	−1.910364000	−0.259695000
C	4.496664000	−1.051372000	0.406402000
H	4.118033000	−0.198796000	0.965026000
C	5.866700000	−1.296037000	0.372635000
H	6.546018000	−0.631223000	0.899194000
C	6.365047000	−2.390674000	−0.335331000
H	7.434780000	−2.578329000	−0.364389000
C	5.487965000	−3.244557000	−1.000965000
H	5.870599000	−4.102387000	−1.547293000
C	4.113051000	−3.010211000	−0.963601000
H	3.438902000	−3.689032000	−1.477484000
C	1.297001000	−1.939881000	1.551192000
C	0.261254000	−1.159013000	2.130482000
C	−0.178703000	−1.526825000	3.418690000
H	−0.991909000	−0.975660000	3.880018000
C	0.407754000	−2.583055000	4.104878000
H	0.043730000	−2.832127000	5.098558000
C	1.448707000	−3.323003000	3.539869000
H	1.910718000	−4.139733000	4.085858000
C	1.879889000	−2.997990000	2.258777000

H	2.685415000	−3.564993000	1.798517000
C	−1.120678000	0.852667000	2.011497000
C	−0.752018000	1.539765000	3.185839000
H	0.172534000	1.262981000	3.684956000
C	−1.546035000	2.557678000	3.695817000
H	−1.239835000	3.069693000	4.604405000
C	−2.722422000	2.939992000	3.042176000
H	−3.338534000	3.739473000	3.443088000
C	−3.099559000	2.288874000	1.874735000
H	−4.010133000	2.583513000	1.361029000
C	−2.325092000	1.237015000	1.361512000
C	−4.100412000	1.364711000	−0.969112000
C	−5.441891000	0.967457000	−0.928640000
H	−5.725495000	0.048884000	−0.423524000
C	−6.420025000	1.751440000	−1.540445000
H	−7.459118000	1.435621000	−1.506158000
C	−6.069278000	2.933840000	−2.189352000
H	−6.834356000	3.541419000	−2.664657000
C	−4.733445000	3.336163000	−2.229881000
H	−4.455318000	4.257934000	−2.733718000
C	−3.751800000	2.552288000	−1.629750000
H	−2.713044000	2.876003000	−1.663140000
C	−3.531031000	−1.235119000	0.270111000
C	−4.063523000	−1.444684000	1.547603000
H	−4.000942000	−0.662809000	2.299216000
C	−4.677977000	−2.658009000	1.853936000
H	−5.090783000	−2.816596000	2.846492000
C	−4.768837000	−3.661445000	0.889537000
H	−5.253078000	−4.603818000	1.130677000
C	−4.239241000	−3.455337000	−0.384488000
H	−4.310551000	−4.234514000	−1.138400000
C	−3.615467000	−2.248523000	−0.693929000
H	−3.201058000	−2.095642000	−1.688010000
C	0.656637000	1.257809000	−3.406497000
C	1.747727000	1.630356000	−2.705863000

H	0.713133000	0.513175000	−4.196152000
H	−0.300682000	1.772613000	−3.297029000
H	2.696546000	1.141966000	−2.937164000
C	1.795832000	2.720631000	−1.642103000
C	0.420653000	2.965353000	−0.985673000
H	0.530822000	3.613218000	−0.109994000
H	−0.266475000	3.477318000	−1.676188000
Y	0.027645000	0.669797000	−0.667347000
H	2.128104000	3.606586000	−2.215492000

117

theta3_thf_C2_cis_1,4 E = −2200.65044 a.u.

N	−1.074057000	1.123144000	1.137977000
P	−2.888157000	−1.055870000	0.058300000
P	1.767884000	1.794199000	0.329602000
Si	4.901170000	−4.051259000	−1.378612000
C	3.983531000	−2.567251000	−2.145100000
H	3.628542000	−2.871149000	−3.140668000
H	4.708810000	−1.758752000	−2.319252000
C	3.701515000	−5.499869000	−1.185143000
H	2.875968000	−5.262568000	−0.502227000
H	4.213323000	−6.381559000	−0.780769000
H	3.263061000	−5.788425000	−2.148268000
C	6.311296000	−4.531822000	−2.534749000
H	5.938243000	−4.807087000	−3.528493000
H	6.869878000	−5.390603000	−2.143389000
H	7.022490000	−3.707171000	−2.664040000
C	5.593613000	−3.581354000	0.317443000
H	6.279453000	−2.728838000	0.244579000
H	6.150263000	−4.416997000	0.758759000
H	4.797592000	−3.309548000	1.021898000
C	−4.188847000	−1.569113000	−1.149353000
C	−4.003210000	−1.269383000	−2.504728000
H	−3.091197000	−0.772387000	−2.821604000
C	−4.969297000	−1.614100000	−3.447708000
H	−4.810846000	−1.380702000	−4.497458000

C	−6.131043000	−2.272531000	−3.045744000
H	−6.882246000	−2.549433000	−3.780281000
C	−6.321834000	−2.582087000	−1.699729000
H	−7.223590000	−3.098477000	−1.381813000
C	−5.357880000	−2.234067000	−0.754313000
H	−5.515230000	−2.486947000	0.290296000
C	−3.151799000	−2.226356000	1.466954000
C	−2.942938000	−3.597111000	1.248218000
H	−2.676285000	−3.958201000	0.256492000
C	−3.102964000	−4.508235000	2.289125000
H	−2.948921000	−5.568324000	2.106228000
C	−3.462956000	−4.060856000	3.561508000
H	−3.586376000	−4.772189000	4.373469000
C	−3.672291000	−2.701337000	3.785029000
H	−3.964744000	−2.349677000	4.770948000
C	−3.519710000	−1.785205000	2.742933000
H	−3.703032000	−0.728818000	2.918331000
C	−3.436598000	0.583482000	0.683838000
C	−2.436749000	1.462402000	1.178072000
C	−2.870910000	2.731623000	1.627239000
H	−2.131551000	3.435312000	1.998100000
C	−4.207075000	3.103994000	1.589934000
H	−4.491414000	4.094138000	1.938118000
C	−5.179797000	2.229890000	1.096654000
H	−6.225508000	2.520718000	1.063716000
C	−4.781978000	0.977857000	0.644515000
H	−5.524303000	0.289136000	0.250325000
C	−0.286910000	1.595403000	2.200909000
C	−0.786079000	1.659666000	3.522328000
H	−1.812539000	1.357625000	3.707644000
C	−0.003693000	2.099275000	4.582204000
H	−0.432854000	2.128343000	5.580926000
C	1.322372000	2.490448000	4.379257000
H	1.933929000	2.839216000	5.205961000
C	1.848373000	2.415570000	3.094285000

H	2.883215000	2.697290000	2.918977000
C	1.070177000	1.973261000	2.015785000
C	3.593016000	1.648966000	0.579606000
C	4.496266000	2.592681000	0.077327000
H	4.137564000	3.466822000	−0.456914000
C	5.868107000	2.424130000	0.269652000
H	6.558354000	3.166058000	−0.122851000
C	6.350002000	1.320260000	0.968848000
H	7.418392000	1.194412000	1.121614000
C	5.453806000	0.379556000	1.478156000
H	5.820956000	−0.481294000	2.030154000
C	4.084568000	0.536943000	1.279839000
H	3.398897000	−0.197468000	1.695597000
C	1.560208000	3.435530000	−0.495068000
C	1.165529000	4.581275000	0.204953000
H	0.968111000	4.522229000	1.271628000
C	1.033701000	5.799332000	−0.462865000
H	0.730164000	6.684970000	0.089030000
C	1.304256000	5.885424000	−1.828272000
H	1.211996000	6.838166000	−2.342594000
C	1.701118000	4.746913000	−2.532067000
H	1.924214000	4.811984000	−3.594042000
C	1.818038000	3.524834000	−1.871526000
H	2.130643000	2.639771000	−2.422580000
C	2.442746000	−2.213637000	3.609834000
C	1.413969000	−1.393152000	3.353190000
H	1.311842000	−0.465406000	3.913539000
C	0.336535000	−1.707883000	2.413224000
C	0.478996000	−2.389214000	1.256811000
H	−0.397967000	−2.743294000	0.715867000
H	1.450076000	−2.752090000	0.932225000
Y	−0.160328000	−0.150395000	−0.638849000
H	−0.660030000	−1.374017000	2.699546000
O	−1.135257000	1.409126000	−2.197311000
C	−1.223392000	1.301997000	−3.649552000

C	−1.792887000	2.658392000	−1.774396000
C	−2.294741000	2.293768000	−4.064384000
H	−0.247268000	1.569535000	−4.069644000
H	−1.453744000	0.264862000	−3.898105000
C	−2.079243000	3.421782000	−3.056201000
H	−2.708777000	2.377917000	−1.251684000
H	−1.118309000	3.171893000	−1.089577000
H	−3.292324000	1.855057000	−3.948780000
H	−2.175321000	2.609807000	−5.104632000
H	−2.949953000	4.074450000	−2.948045000
H	−1.219848000	4.037799000	−3.341101000
H	3.200993000	−1.952797000	4.342831000
H	2.537248000	−3.185090000	3.130166000
C	2.788907000	−2.013073000	−1.339044000
C	2.060975000	−0.945477000	−2.131026000
H	2.103027000	−2.837596000	−1.110866000
H	3.161758000	−1.625600000	−0.381494000
H	2.713681000	−0.165174000	−2.523545000
C	0.875507000	−1.159760000	−2.823888000
C	−0.226774000	−1.955669000	−2.431154000
H	0.684435000	−0.456584000	−3.636562000
H	−1.047984000	−2.091414000	−3.130436000
H	−0.047176000	−2.835426000	−1.805423000

117

theta3_thf_TS2_cis_1,4 E = −2200.633721 a.u. Imaginary frequency=148i

N	−1.344113000	−1.401583000	−0.868437000
P	−2.913279000	1.072747000	−0.019369000
P	1.538024000	−2.097278000	−0.222338000
Si	5.663486000	4.288472000	0.264059000
C	4.249851000	3.508398000	1.281454000
H	3.644440000	4.319190000	1.710994000
H	4.690714000	2.977086000	2.137590000
C	4.927607000	5.396038000	−1.076999000
H	4.322797000	4.831199000	−1.797582000
H	5.719157000	5.898610000	−1.645800000

H	4.290247000	6.176677000	−0.644064000
C	6.730614000	5.305659000	1.438357000
H	6.146626000	6.090027000	1.934595000
H	7.551239000	5.796818000	0.901790000
H	7.177359000	4.679581000	2.220064000
C	6.693683000	2.919254000	−0.534464000
H	7.097514000	2.228135000	0.215793000
H	7.544964000	3.342357000	−1.081124000
H	6.106318000	2.334499000	−1.253705000
C	−3.688124000	1.865521000	1.464105000
C	−2.945565000	1.963198000	2.648557000
H	−1.931054000	1.575211000	2.681119000
C	−3.498255000	2.546510000	3.787990000
H	−2.909385000	2.620393000	4.698654000
C	−4.799593000	3.044739000	3.755481000
H	−5.229673000	3.504601000	4.640976000
C	−5.544951000	2.959235000	2.579812000
H	−6.558229000	3.350501000	2.547032000
C	−4.994110000	2.377488000	1.439617000
H	−5.578273000	2.336389000	0.524733000
C	−3.764750000	1.944215000	−1.415457000
C	−3.752529000	3.347340000	−1.461674000
H	−3.312667000	3.917390000	−0.645999000
C	−4.332378000	4.023316000	−2.532923000
H	−4.326539000	5.110002000	−2.550626000
C	−4.923984000	3.308328000	−3.575357000
H	−5.376023000	3.836400000	−4.410347000
C	−4.941370000	1.915680000	−3.535397000
H	−5.411538000	1.352960000	−4.337535000
C	−4.366782000	1.234432000	−2.461101000
H	−4.401581000	0.149067000	−2.429369000
C	−3.555829000	−0.644128000	−0.086785000
C	−2.683168000	−1.661916000	−0.558754000
C	−3.220789000	−2.970919000	−0.622063000
H	−2.590479000	−3.779948000	−0.977632000

C	−4.522985000	−3.251053000	−0.236499000
H	−4.883023000	−4.275402000	−0.296238000
C	−5.364171000	−2.240745000	0.237709000
H	−6.381883000	−2.459563000	0.546236000
C	−4.867021000	−0.946790000	0.307407000
H	−5.505038000	−0.149880000	0.679623000
C	−0.744472000	−2.216695000	−1.846432000
C	−1.428004000	−2.565232000	−3.032924000
H	−2.458100000	−2.241751000	−3.152312000
C	−0.814647000	−3.292830000	−4.043249000
H	−1.375175000	−3.534598000	−4.942918000
C	0.517235000	−3.694209000	−3.917309000
H	1.005301000	−4.256569000	−4.707803000
C	1.215071000	−3.360252000	−2.762106000
H	2.254060000	−3.659961000	−2.666617000
C	0.604187000	−2.646177000	−1.722249000
C	3.324175000	−2.395376000	−0.599350000
C	3.849980000	−3.693745000	−0.697037000
H	3.204013000	−4.554884000	−0.549080000
C	5.202445000	−3.887151000	−0.967214000
H	5.596621000	−4.896933000	−1.043679000
C	6.049874000	−2.790574000	−1.132341000
H	7.105324000	−2.945042000	−1.339151000
C	5.542136000	−1.498187000	−1.018736000
H	6.199945000	−0.640554000	−1.130519000
C	4.187096000	−1.304160000	−0.750030000
H	3.799056000	−0.295349000	−0.645810000
C	1.230116000	−3.418313000	1.044335000
C	0.169797000	−4.323152000	0.934247000
H	−0.492936000	−4.278051000	0.075849000
C	−0.024152000	−5.304189000	1.908231000
H	−0.846959000	−6.006781000	1.805543000
C	0.842176000	−5.395743000	2.996582000
H	0.697097000	−6.168405000	3.746673000
C	1.905186000	−4.498074000	3.111530000

H	2.594028000	−4.571685000	3.949055000
C	2.095545000	−3.512605000	2.145011000
H	2.945635000	−2.839104000	2.230754000
C	1.732649000	0.559243000	−2.462969000
C	0.465575000	0.516846000	−2.968713000
H	0.188938000	−0.342898000	−3.574773000
C	−0.585036000	1.434768000	−2.617832000
C	−0.308158000	2.683872000	−2.074516000
H	−1.118752000	3.385146000	−1.917556000
H	0.669601000	3.122156000	−2.241553000
Y	−0.030709000	0.446300000	−0.265819000
H	−1.610547000	1.164358000	−2.851166000
O	−0.417927000	−0.407420000	2.135129000
C	0.520668000	−0.353216000	3.242554000
C	−1.485832000	−1.353527000	2.475689000
C	−0.236899000	−0.884217000	4.446760000
H	1.381942000	−0.985455000	3.000265000
H	0.852696000	0.678678000	3.364103000
C	−1.101512000	−1.971167000	3.812233000
H	−2.416652000	−0.783135000	2.537386000
H	−1.558329000	−2.079894000	1.668625000
H	−0.858053000	−0.096455000	4.888954000
H	0.437828000	−1.262135000	5.220344000
H	−1.983399000	−2.223789000	4.407927000
H	−0.517024000	−2.883473000	3.659401000
H	2.471701000	−0.179472000	−2.750745000
H	2.105748000	1.420551000	−1.902993000
C	3.326073000	2.544498000	0.503303000
C	2.212427000	2.045109000	1.377543000
H	2.924158000	3.051213000	−0.382744000
H	3.930645000	1.702853000	0.139782000
H	2.530759000	1.487825000	2.257575000
C	0.896743000	2.415174000	1.291593000
C	0.233881000	3.128775000	0.225895000
H	0.244869000	2.075862000	2.100011000

H	−0.738123000	3.536287000	0.497901000
H	0.849630000	3.853488000	−0.299252000

117

theta3_thf_P2_cis_1,4 E = −2200.685665 a.u.

N	1.646617000	1.351032000	−0.708390000
P	2.724220000	−1.385164000	−0.023490000
P	−1.099437000	2.506622000	−0.183474000
Si	−6.361807000	−3.657547000	0.167167000
C	−4.677768000	−3.169382000	0.926733000
H	−4.250237000	−4.058993000	1.411127000
H	−4.866290000	−2.446930000	1.735268000
C	−6.090861000	−4.930653000	−1.199498000
H	−5.507490000	−4.525616000	−2.035459000
H	−7.050840000	−5.266912000	−1.609423000
H	−5.567599000	−5.818741000	−0.824579000
C	−7.413626000	−4.394593000	1.547336000
H	−6.944892000	−5.288661000	1.975707000
H	−8.401687000	−4.689691000	1.174113000
H	−7.571802000	−3.678568000	2.362876000
C	−7.198249000	−2.119160000	−0.541018000
H	−7.330613000	−1.343343000	0.223094000
H	−8.192882000	−2.365988000	−0.931534000
H	−6.625648000	−1.684284000	−1.369517000
C	3.308362000	−2.510245000	1.325306000
C	2.502119000	−2.679560000	2.459099000
H	1.552428000	−2.155018000	2.526609000
C	2.907201000	−3.509098000	3.503611000
H	2.270127000	−3.635403000	4.375347000
C	4.123368000	−4.185866000	3.424162000
H	4.438472000	−4.837615000	4.234377000
C	4.930377000	−4.030150000	2.297593000
H	5.877438000	−4.558561000	2.228701000
C	4.527043000	−3.200430000	1.252563000
H	5.157587000	−3.098776000	0.374038000
C	3.564410000	−2.069962000	−1.524836000

C	3.375340000	−3.419434000	−1.860251000
H	2.786897000	−4.065984000	−1.213228000
C	3.954490000	−3.948202000	−3.010782000
H	3.810411000	−4.997821000	−3.253095000
C	4.718045000	−3.133245000	−3.848375000
H	5.167860000	−3.545610000	−4.747329000
C	4.906103000	−1.791912000	−3.523324000
H	5.507052000	−1.154193000	−4.166223000
C	4.335404000	−1.260745000	−2.365523000
H	4.505078000	−0.219196000	−2.109640000
C	3.588810000	0.215992000	0.268267000
C	2.945258000	1.397471000	−0.185007000
C	3.644476000	2.613142000	−0.024212000
H	3.183972000	3.533402000	−0.369185000
C	4.896325000	2.657396000	0.575342000
H	5.396059000	3.616474000	0.687987000
C	5.507425000	1.492184000	1.043083000
H	6.483679000	1.527542000	1.517217000
C	4.846097000	0.280031000	0.881324000
H	5.313903000	−0.635945000	1.231969000
C	1.273760000	2.321270000	−1.662519000
C	2.111214000	2.636353000	−2.751434000
H	3.084559000	2.158099000	−2.813731000
C	1.709609000	3.525668000	−3.739075000
H	2.378736000	3.741434000	−4.568168000
C	0.448708000	4.123727000	−3.682203000
H	0.127300000	4.811429000	−4.458908000
C	−0.395707000	3.828301000	−2.617864000
H	−1.379129000	4.287090000	−2.573099000
C	0.004295000	2.953278000	−1.598977000
C	−2.793134000	3.090998000	−0.638222000
C	−3.125015000	4.454371000	−0.619503000
H	−2.379343000	5.192638000	−0.337207000
C	−4.413611000	4.869274000	−0.948045000
H	−4.658231000	5.927975000	−0.933013000

C	−5.387296000	3.930113000	−1.289501000
H	−6.392542000	4.256015000	−1.542459000
C	−5.070083000	2.573125000	−1.297410000
H	−5.827358000	1.837262000	−1.554453000
C	−3.780448000	2.155512000	−0.970379000
H	−3.538484000	1.097188000	−0.975782000
C	−0.653637000	3.704484000	1.160424000
C	0.415793000	4.599298000	1.054814000
H	1.009789000	4.625166000	0.146314000
C	0.706159000	5.477397000	2.100747000
H	1.536406000	6.172049000	2.003811000
C	−0.073630000	5.477532000	3.255282000
H	0.147117000	6.169757000	4.063189000
C	−1.148977000	4.593286000	3.364812000
H	−1.772519000	4.600599000	4.255317000
C	−1.432533000	3.707076000	2.328062000
H	−2.284403000	3.035140000	2.416310000
C	−1.495119000	−0.073054000	−2.329346000
C	−0.327787000	−0.730035000	−2.787420000
H	0.415223000	−0.093259000	−3.277798000
C	0.104674000	−1.995477000	−2.420374000
C	−0.839202000	−3.139957000	−2.106962000
H	−0.627194000	−3.949121000	−2.820558000
H	−1.868482000	−2.823358000	−2.312865000
Y	0.023103000	−0.177579000	−0.301714000
H	1.102377000	−2.282511000	−2.741514000
O	0.107292000	−0.008439000	2.175697000
C	−0.794322000	−0.422699000	3.236822000
C	1.155735000	0.853219000	2.739490000
C	−0.068889000	−0.111545000	4.536451000
H	−1.724497000	0.151216000	3.143882000
H	−1.009206000	−1.486363000	3.104948000
C	0.724799000	1.142215000	4.168225000
H	2.094644000	0.294488000	2.694012000
H	1.230075000	1.743344000	2.113257000

H	0.605567000	−0.932078000	4.805669000
H	−0.764762000	0.043525000	5.365771000
H	1.585132000	1.310798000	4.821810000
H	0.087467000	2.031237000	4.203159000
H	−1.762388000	0.872869000	−2.791540000
H	−2.349288000	−0.686545000	−2.028779000
C	−3.648959000	−2.584570000	−0.062213000
C	−2.364554000	−2.252629000	0.639068000
H	−3.470999000	−3.288651000	−0.880542000
H	−4.062685000	−1.671826000	−0.515381000
H	−2.489520000	−1.586373000	1.495280000
C	−1.138092000	−2.764890000	0.402259000
C	−0.774309000	−3.741501000	−0.691683000
H	−0.345064000	−2.533806000	1.124391000
H	0.235395000	−4.122325000	−0.501933000
H	−1.444341000	−4.611352000	−0.643988000

117

theta3_thf_C2_cis_1,2 E = −2200.653446 a.u.

N	−1.121150000	1.078201000	1.258746000
P	−2.809221000	−1.007925000	−0.086768000
P	1.527646000	2.189078000	0.289398000
Si	5.297462000	−4.091853000	−0.902947000
C	4.041016000	−2.961640000	−1.785741000
H	3.509503000	−3.567213000	−2.534214000
H	4.606825000	−2.212865000	−2.361249000
C	4.400260000	−5.561630000	−0.125365000
H	3.658665000	−5.244198000	0.617371000
H	5.107125000	−6.229785000	0.381208000
H	3.875338000	−6.153421000	−0.885203000
C	6.528495000	−4.710326000	−2.191801000
H	6.023154000	−5.259551000	−2.995256000
H	7.263989000	−5.388372000	−1.742377000
H	7.081184000	−3.882667000	−2.652556000
C	6.212758000	−3.122315000	0.438352000
H	6.732820000	−2.252458000	0.018297000

H	6.970157000	−3.749389000	0.924226000
H	5.538043000	−2.761833000	1.224295000
C	−3.898595000	−1.425433000	−1.517625000
C	−3.419335000	−1.225457000	−2.819171000
H	−2.411924000	−0.846559000	−2.971934000
C	−4.218669000	−1.523233000	−3.922496000
H	−3.833910000	−1.369899000	−4.927422000
C	−5.503097000	−2.031249000	−3.736337000
H	−6.124864000	−2.268264000	−4.595264000
C	−5.983525000	−2.248533000	−2.444739000
H	−6.980035000	−2.655181000	−2.295130000
C	−5.186431000	−1.953316000	−1.340823000
H	−5.562109000	−2.151334000	−0.340643000
C	−3.226785000	−2.332125000	1.138560000
C	−3.182555000	−3.676137000	0.736803000
H	−2.940016000	−3.931986000	−0.291392000
C	−3.476621000	−4.690895000	1.645398000
H	−3.456947000	−5.727488000	1.319197000
C	−3.799456000	−4.377320000	2.967211000
H	−4.029509000	−5.169595000	3.674266000
C	−3.835853000	−3.044415000	3.373596000
H	−4.097431000	−2.793808000	4.398231000
C	−3.555157000	−2.023862000	2.463830000
H	−3.615132000	−0.985995000	2.779315000
C	−3.448017000	0.539822000	0.658697000
C	−2.491573000	1.374216000	1.300542000
C	−2.993739000	2.545371000	1.916198000
H	−2.305891000	3.206719000	2.433464000
C	−4.342013000	2.874430000	1.877864000
H	−4.674847000	3.791016000	2.359115000
C	−5.264211000	2.054921000	1.223809000
H	−6.317238000	2.316793000	1.187081000
C	−4.803249000	0.890391000	0.622556000
H	−5.506252000	0.240075000	0.109514000
C	−0.326564000	1.589627000	2.302590000

C	−0.729294000	1.483581000	3.651842000
H	−1.702191000	1.052183000	3.870676000
C	0.083957000	1.910907000	4.692133000
H	−0.260939000	1.804675000	5.717625000
C	1.343073000	2.455018000	4.428854000
H	1.986649000	2.782725000	5.239807000
C	1.767636000	2.565911000	3.110075000
H	2.752307000	2.972809000	2.901568000
C	0.951448000	2.155023000	2.046251000
C	3.349694000	2.462769000	0.370491000
C	3.904968000	3.697800000	0.741170000
H	3.257622000	4.538512000	0.975716000
C	5.287528000	3.855175000	0.799261000
H	5.708578000	4.814250000	1.088780000
C	6.129554000	2.787432000	0.483688000
H	7.207779000	2.914566000	0.528143000
C	5.586120000	1.562790000	0.101455000
H	6.237931000	0.732814000	−0.156941000
C	4.201688000	1.403235000	0.040770000
H	3.777334000	0.456441000	−0.279686000
C	0.927754000	3.806712000	−0.391460000
C	−0.151925000	4.488950000	0.177947000
H	−0.632720000	4.094293000	1.066840000
C	−0.600777000	5.688493000	−0.376085000
H	−1.434893000	6.212692000	0.082912000
C	0.027713000	6.221848000	−1.500442000
H	−0.314266000	7.162750000	−1.922871000
C	1.109297000	5.549160000	−2.072029000
H	1.616179000	5.966892000	−2.938094000
C	1.553979000	4.346211000	−1.525892000
H	2.415931000	3.847230000	−1.963402000
C	1.424911000	−1.347060000	2.077600000
C	0.327966000	−2.066077000	2.403654000
H	−0.534723000	−1.536586000	2.810939000
C	0.194914000	−3.520608000	2.296654000

C	1.213102000	−4.380889000	2.440637000
H	1.057392000	−5.453902000	2.375862000
H	2.222416000	−4.045877000	2.667617000
Y	−0.029311000	−0.285805000	−0.326677000
H	−0.813009000	−3.904237000	2.153824000
O	−0.699215000	1.090494000	−2.166531000
C	0.098379000	1.416724000	−3.341841000
C	−1.854885000	1.998336000	−2.100791000
C	−0.860567000	2.138455000	−4.269988000
H	0.922945000	2.064067000	−3.026620000
H	0.500070000	0.485767000	−3.743897000
C	−1.703484000	2.947773000	−3.282544000
H	−2.751746000	1.378386000	−2.175184000
H	−1.833118000	2.497625000	−1.132026000
H	−1.478778000	1.419233000	−4.819434000
H	−0.333614000	2.763038000	−4.996782000
H	−2.675754000	3.239923000	−3.689064000
H	−1.171105000	3.854440000	−2.979946000
H	1.496906000	−0.296304000	2.349500000
H	2.315350000	−1.824512000	1.678550000
C	3.004850000	−2.236273000	−0.901814000
C	2.055974000	−1.407886000	−1.756740000
H	2.439969000	−2.976917000	−0.321490000
H	3.542337000	−1.617176000	−0.171380000
H	2.571841000	−0.753779000	−2.462468000
C	0.820559000	−1.901936000	−2.192834000
C	−0.121479000	−2.620246000	−1.441510000
H	0.440385000	−1.498554000	−3.136125000
H	−1.016788000	−2.988578000	−1.933193000
H	0.198661000	−3.201283000	−0.571989000

117

theta3_thf_TS2_cis_1,2 E = −2200.63101 a.u. Imaginary frequency=220i

N	1.329819000	−1.303716000	−0.987158000
P	−1.489684000	−2.223780000	−0.160313000
P	2.829250000	1.195084000	−0.130071000

Si	−5.844105000	4.101372000	0.509196000
C	−4.032438000	3.828363000	1.044454000
H	−4.029319000	3.137644000	1.899448000
H	−3.642621000	4.782629000	1.428548000
C	−6.583450000	2.456089000	−0.058329000
H	−6.078501000	2.065627000	−0.950690000
H	−7.643532000	2.574100000	−0.313380000
H	−6.519666000	1.693587000	0.728029000
C	−6.794960000	4.754841000	2.001084000
H	−6.769548000	4.047241000	2.838551000
H	−7.848174000	4.930928000	1.751188000
H	−6.378881000	5.705189000	2.356453000
C	−5.915184000	5.351661000	−0.903834000
H	−5.459067000	6.307726000	−0.619308000
H	−6.954658000	5.557905000	−1.186404000
H	−5.401574000	4.991221000	−1.803569000
C	−2.369935000	−2.943661000	1.306931000
C	−1.975442000	−2.550667000	2.593610000
H	−1.180034000	−1.820968000	2.711788000
C	−2.588053000	−3.091986000	3.721572000
H	−2.271641000	−2.777426000	4.712935000
C	−3.610622000	−4.029814000	3.580487000
H	−4.093197000	−4.447977000	4.459559000
C	−4.010327000	−4.426832000	2.306182000
H	−4.803671000	−5.159899000	2.186876000
C	−3.394857000	−3.890170000	1.175161000
H	−3.716645000	−4.212034000	0.189711000
C	−2.596930000	−2.702466000	−1.562881000
C	−3.888396000	−2.154398000	−1.614996000
H	−4.240569000	−1.507128000	−0.814114000
C	−4.736010000	−2.446961000	−2.680230000
H	−5.737367000	−2.025270000	−2.704352000
C	−4.300211000	−3.279130000	−3.713296000
H	−4.960594000	−3.504592000	−4.546077000
C	−3.018073000	−3.822287000	−3.669602000

H	−2.675668000	−4.476535000	−4.467010000
C	−2.168198000	−3.538858000	−2.598523000
H	−1.174929000	−3.977092000	−2.566892000
C	0.006527000	−3.279430000	−0.340218000
C	1.221299000	−2.683466000	−0.771914000
C	2.338682000	−3.543127000	−0.895795000
H	3.287475000	−3.125384000	−1.217665000
C	2.259586000	−4.897534000	−0.607639000
H	3.149373000	−5.513832000	−0.711755000
C	1.060383000	−5.468001000	−0.172858000
H	0.996819000	−6.527348000	0.056712000
C	−0.052193000	−4.648360000	−0.038539000
H	−0.992250000	−5.072524000	0.304885000
C	2.271696000	−0.873562000	−1.941703000
C	2.412806000	−1.536734000	−3.180875000
H	1.818162000	−2.427442000	−3.362537000
C	3.272605000	−1.070015000	−4.165362000
H	3.345657000	−1.606409000	−5.108147000
C	4.020858000	0.090818000	−3.958842000
H	4.685981000	0.467813000	−4.730040000
C	3.899328000	0.764198000	−2.749035000
H	4.469130000	1.674395000	−2.592584000
C	3.054715000	0.294202000	−1.733231000
C	3.493446000	2.903675000	−0.384554000
C	4.859692000	3.148528000	−0.596593000
H	5.563807000	2.321126000	−0.619976000
C	5.326019000	4.451259000	−0.753337000
H	6.385052000	4.627154000	−0.921614000
C	4.440406000	5.527806000	−0.683674000
H	4.808178000	6.543282000	−0.802304000
C	3.087689000	5.298506000	−0.442466000
H	2.398135000	6.134945000	−0.363825000
C	2.619421000	3.993125000	−0.289590000
H	1.568275000	3.820590000	−0.074974000
C	4.134107000	0.498017000	0.989423000

C	4.823117000	−0.682541000	0.694324000
H	4.610942000	−1.217298000	−0.226772000
C	5.804358000	−1.161736000	1.564127000
H	6.338156000	−2.076265000	1.319393000
C	6.113333000	−0.462509000	2.729693000
H	6.886652000	−0.830478000	3.398408000
C	5.433768000	0.720091000	3.027765000
H	5.678964000	1.279028000	3.927136000
C	4.446262000	1.194293000	2.167074000
H	3.942710000	2.130883000	2.397383000
C	−0.632398000	3.014982000	−1.625635000
C	0.195130000	2.033478000	−2.187582000
H	1.249257000	2.246510000	−2.338780000
C	−0.355384000	0.845572000	−2.775190000
C	−1.583452000	0.330803000	−2.462980000
H	−1.949253000	−0.569174000	−2.940084000
H	−2.321006000	0.901708000	−1.892186000
Y	−0.048609000	0.359675000	−0.166322000
H	0.303638000	0.260987000	−3.413827000
O	0.861950000	−0.471594000	2.117118000
C	0.954558000	0.318287000	3.327084000
C	1.627641000	−1.706464000	2.296244000
C	1.373130000	−0.651344000	4.419986000
H	1.706586000	1.103369000	3.178293000
H	−0.017458000	0.780586000	3.512328000
C	2.299776000	−1.598749000	3.658457000
H	0.919809000	−2.538673000	2.247184000
H	2.331623000	−1.788402000	1.470267000
H	0.500394000	−1.186505000	4.811818000
H	1.862883000	−0.142665000	5.255352000
H	2.399549000	−2.576961000	4.137428000
H	3.297948000	−1.161376000	3.561551000
H	−0.198592000	3.983871000	−1.407109000
H	−1.668572000	3.033060000	−1.945155000
C	−3.087267000	3.299394000	−0.050872000

C	−1.670764000	3.109532000	0.453181000
H	−3.483620000	2.356828000	−0.455045000
H	−3.084202000	4.010582000	−0.886595000
H	−1.133230000	4.039272000	0.631187000
C	−1.386408000	2.106635000	1.418555000
C	−2.002076000	0.877973000	1.576687000
H	−0.533257000	2.320289000	2.070831000
H	−1.803362000	0.264741000	2.447273000
H	−2.908984000	0.629555000	1.027545000

117

theta3_thf_P2_cis_1,2 E = −2200.682955 a.u.

N	2.155067000	0.745867000	−0.598862000
P	2.083212000	−2.243210000	−0.067639000
P	0.024120000	2.813113000	−0.090958000
Si	−7.593037000	−2.108641000	0.252521000
C	−5.865159000	−1.312968000	0.418931000
H	−5.349447000	−1.827568000	1.243396000
H	−6.007148000	−0.272383000	0.750570000
C	−7.405757000	−3.921400000	−0.241266000
H	−6.927609000	−4.034182000	−1.221716000
H	−8.385533000	−4.410610000	−0.300913000
H	−6.806631000	−4.479115000	0.489113000
C	−8.449841000	−1.985197000	1.929735000
H	−7.889743000	−2.514955000	2.709942000
H	−9.453375000	−2.425907000	1.891646000
H	−8.563413000	−0.942599000	2.250746000
C	−8.596983000	−1.184033000	−1.052066000
H	−8.706098000	−0.122017000	−0.800273000
H	−9.606344000	−1.605483000	−1.132594000
H	−8.140519000	−1.246649000	−2.047246000
C	2.145272000	−3.510730000	1.280741000
C	1.325010000	−3.335284000	2.403112000
H	0.673593000	−2.466816000	2.454191000
C	1.339743000	−4.258601000	3.447309000
H	0.696434000	−4.112838000	4.311551000

C	2.170935000	−5.375684000	3.377344000
H	2.180371000	−6.100073000	4.187093000
C	2.983061000	−5.565605000	2.259687000
H	3.627955000	−6.438032000	2.197182000
C	2.970528000	−4.642451000	1.215049000
H	3.595937000	−4.811253000	0.342927000
C	2.575227000	−3.223637000	−1.556199000
C	1.766204000	−4.301464000	−1.945192000
H	0.900541000	−4.579192000	−1.347115000
C	2.075613000	−5.037985000	−3.085349000
H	1.447077000	−5.876660000	−3.372872000
C	3.189394000	−4.699802000	−3.856126000
H	3.428710000	−5.273152000	−4.747482000
C	3.994651000	−3.628475000	−3.475998000
H	4.867494000	−3.365518000	−4.067776000
C	3.692124000	−2.892350000	−2.329133000
H	4.332347000	−2.067169000	−2.031424000
C	3.509672000	−1.117676000	0.245491000
C	3.376197000	0.243325000	−0.136069000
C	4.503334000	1.075611000	0.031843000
H	4.432111000	2.118112000	−0.262499000
C	5.686121000	0.594892000	0.576977000
H	6.528478000	1.271484000	0.698554000
C	5.795946000	−0.737510000	0.982029000
H	6.717981000	−1.112056000	1.416471000
C	4.705145000	−1.581725000	0.810031000
H	4.781656000	−2.623250000	1.110842000
C	2.151475000	1.749547000	−1.580397000
C	3.032134000	1.718121000	−2.682793000
H	3.787016000	0.938275000	−2.723389000
C	2.929545000	2.640599000	−3.713638000
H	3.617929000	2.581897000	−4.552955000
C	1.936101000	3.624349000	−3.690935000
H	1.846835000	4.338266000	−4.504412000
C	1.065634000	3.685675000	−2.609591000

H	0.297652000	4.452846000	−2.586689000
C	1.173057000	2.782160000	−1.541758000
C	−1.280166000	4.056700000	−0.499047000
C	−0.985999000	5.427763000	−0.558442000
H	0.026828000	5.775711000	−0.373460000
C	−1.989954000	6.350345000	−0.841694000
H	−1.750457000	7.409258000	−0.888485000
C	−3.299432000	5.918306000	−1.057422000
H	−4.081503000	6.640710000	−1.274567000
C	−3.603832000	4.560647000	−0.983324000
H	−4.624301000	4.220806000	−1.139238000
C	−2.599019000	3.634769000	−0.702462000
H	−2.841508000	2.578093000	−0.637901000
C	0.958213000	3.704202000	1.239824000
C	2.291094000	4.100891000	1.097348000
H	2.819436000	3.896852000	0.171211000
C	2.936768000	4.781998000	2.130833000
H	3.971568000	5.090114000	2.006595000
C	2.255393000	5.081311000	3.308681000
H	2.756994000	5.620739000	4.107423000
C	0.920596000	4.698339000	3.453407000
H	0.377543000	4.945823000	4.361999000
C	0.277415000	4.007780000	2.428570000
H	−0.769443000	3.732521000	2.542825000
C	−2.908108000	−0.615203000	−2.104296000
C	−1.737468000	0.349168000	−2.253845000
H	−2.034179000	1.379802000	−2.443789000
C	−0.533389000	−0.040912000	−2.870609000
C	0.117686000	−1.263242000	−2.731321000
H	1.079158000	−1.416324000	−3.209781000
H	−0.432425000	−2.178543000	−2.509563000
Y	0.072640000	−0.109394000	−0.360139000
H	0.071447000	0.771631000	−3.281134000
O	0.197332000	−0.056613000	2.131451000
C	−0.865475000	−0.040173000	3.118622000

C	1.463307000	0.319402000	2.773926000
C	−0.168241000	−0.059833000	4.469521000
H	−1.458335000	0.872379000	2.979513000
H	−1.500404000	−0.912234000	2.945432000
C	1.097519000	0.750274000	4.186551000
H	2.108135000	−0.563900000	2.762034000
H	1.918568000	1.110532000	2.177294000
H	0.083634000	−1.086935000	4.756724000
H	−0.791079000	0.371209000	5.258479000
H	1.905366000	0.542545000	4.893673000
H	0.885076000	1.823469000	4.214804000
H	−3.641358000	−0.365279000	−2.885705000
H	−2.593972000	−1.640847000	−2.336237000
C	−5.006526000	−1.341902000	−0.849922000
C	−3.647988000	−0.595229000	−0.760859000
H	−4.801623000	−2.380744000	−1.143526000
H	−5.560716000	−0.888053000	−1.682328000
H	−3.878746000	0.450915000	−0.509046000
C	−2.835574000	−1.160983000	0.373327000
C	−1.921147000	−2.147535000	0.299693000
H	−3.082194000	−0.776156000	1.363281000
H	−1.470068000	−2.573611000	1.193565000
H	−1.680602000	−2.644792000	−0.638425000

117

theta3_thf_C2_trans_1,4 E = −2200.657535 a.u.

N	−1.201371000	1.213045000	1.106703000
P	−3.003231000	−0.974631000	0.089966000
P	1.685239000	1.761459000	0.325624000
Si	5.050873000	−3.740553000	−1.435328000
C	3.431084000	−3.032407000	−2.144903000
H	2.725375000	−3.860618000	−2.302456000
H	3.666570000	−2.659148000	−3.153557000
C	4.779910000	−4.385692000	0.321248000
H	4.484186000	−3.582455000	1.006456000
H	5.698426000	−4.835814000	0.717277000

H	4.000417000	−5.157491000	0.348642000
C	5.590582000	−5.160833000	−2.554720000
H	4.841455000	−5.961355000	−2.580778000
H	6.532675000	−5.602687000	−2.208078000
H	5.747538000	−4.820554000	−3.585417000
C	6.383219000	−2.401247000	−1.424102000
H	6.579492000	−2.029917000	−2.437493000
H	7.329818000	−2.793871000	−1.032781000
H	6.102270000	−1.540786000	−0.805355000
C	−4.180803000	−1.514756000	−1.222872000
C	−3.870363000	−1.229076000	−2.559746000
H	−2.945454000	−0.709693000	−2.801360000
C	−4.737127000	−1.606761000	−3.583275000
H	−4.486188000	−1.383731000	−4.617115000
C	−5.919438000	−2.282511000	−3.281788000
H	−6.592364000	−2.584393000	−4.079616000
C	−6.232407000	−2.576427000	−1.955260000
H	−7.151239000	−3.105176000	−1.716509000
C	−5.368991000	−2.196353000	−0.928499000
H	−5.617503000	−2.437495000	0.101203000
C	−3.360561000	−2.108318000	1.503646000
C	−3.097630000	−3.478468000	1.349896000
H	−2.729383000	−3.860536000	0.400675000
C	−3.324193000	−4.358514000	2.404948000
H	−3.128523000	−5.419535000	2.273844000
C	−3.802835000	−3.879304000	3.625881000
H	−3.977160000	−4.566705000	4.449087000
C	−4.061708000	−2.519359000	3.784277000
H	−4.441632000	−2.143102000	4.730384000
C	−3.843813000	−1.633996000	2.727561000
H	−4.060270000	−0.576820000	2.851876000
C	−3.560967000	0.688086000	0.619061000
C	−2.558181000	1.577306000	1.089346000
C	−2.987034000	2.873671000	1.457863000
H	−2.249970000	3.587620000	1.813454000

C	−4.318194000	3.257439000	1.369848000
H	−4.597291000	4.268285000	1.657613000
C	−5.292775000	2.369633000	0.905618000
H	−6.333335000	2.671527000	0.834188000
C	−4.900772000	1.090544000	0.530891000
H	−5.641212000	0.387842000	0.157108000
C	−0.429125000	1.733844000	2.156270000
C	−0.952466000	1.878554000	3.462173000
H	−1.987381000	1.603232000	3.641804000
C	−0.181015000	2.355845000	4.512130000
H	−0.626317000	2.443316000	5.500202000
C	1.156316000	2.709007000	4.313685000
H	1.760221000	3.087472000	5.132960000
C	1.705060000	2.557617000	3.045975000
H	2.747710000	2.813121000	2.878221000
C	0.938252000	2.075389000	1.975294000
C	3.502237000	1.701508000	0.654454000
C	4.337133000	2.812562000	0.477282000
H	3.932076000	3.749471000	0.107372000
C	5.695943000	2.725973000	0.780552000
H	6.333076000	3.594597000	0.637571000
C	6.232833000	1.537422000	1.271396000
H	7.291279000	1.473683000	1.508316000
C	5.405538000	0.429714000	1.457971000
H	5.815291000	−0.500524000	1.842021000
C	4.050271000	0.508336000	1.144577000
H	3.419424000	−0.363272000	1.294623000
C	1.471911000	3.324211000	−0.641038000
C	0.733180000	4.404443000	−0.147754000
H	0.287836000	4.345858000	0.841328000
C	0.588404000	5.563736000	−0.911940000
H	0.019189000	6.400694000	−0.516031000
C	1.187470000	5.657665000	−2.167505000
H	1.086427000	6.567299000	−2.753312000
C	1.929591000	4.583893000	−2.664575000

H	2.411503000	4.656736000	−3.636208000
C	2.063241000	3.419439000	−1.910674000
H	2.658436000	2.593918000	−2.296690000
C	3.420008000	−2.315161000	3.641485000
C	2.307596000	−2.545989000	2.926267000
H	2.149746000	−3.523032000	2.470504000
C	1.251246000	−1.561437000	2.768580000
C	0.062096000	−1.804295000	2.174739000
H	−0.739410000	−1.070772000	2.243054000
H	−0.195921000	−2.792447000	1.799522000
Y	−0.249650000	−0.308305000	−0.465863000
H	1.425821000	−0.580130000	3.211198000
O	−0.976705000	1.078726000	−2.271470000
C	−0.393873000	1.179012000	−3.607126000
C	−1.921416000	2.193146000	−2.076842000
C	−1.342814000	2.072566000	−4.384223000
H	0.598285000	1.628321000	−3.505971000
H	−0.300556000	0.170715000	−4.010154000
C	−1.786182000	3.068184000	−3.312904000
H	−2.917217000	1.752810000	−1.980193000
H	−1.652288000	2.694856000	−1.148052000
H	−2.195632000	1.495591000	−4.760041000
H	−0.848394000	2.546777000	−5.236792000
H	−2.728192000	3.568452000	−3.554523000
H	−1.019237000	3.834090000	−3.159357000
H	4.174992000	−3.082982000	3.781870000
H	3.597188000	−1.356057000	4.123573000
C	2.712245000	−1.906824000	−1.366630000
C	1.569232000	−1.340141000	−2.192757000
H	2.364362000	−2.303479000	−0.400468000
H	3.422449000	−1.106985000	−1.127415000
H	1.902099000	−0.738978000	−3.040161000
C	0.348012000	−1.997815000	−2.357232000
C	−0.358267000	−2.723709000	−1.384117000
H	−0.235977000	−1.729499000	−3.242039000

H	−1.296923000	−3.193390000	−1.662404000
H	0.188646000	−3.230850000	−0.585972000

117

theta3_thf_TS2_trans_1,4 E = −2200.626351 a.u. Imaginary frequency=249i

N	−1.389220000	−1.501963000	−0.842024000
P	−2.904067000	1.028960000	−0.060940000
P	1.492295000	−2.189036000	−0.259538000
Si	5.456917000	4.272210000	0.286223000
C	3.971710000	3.356611000	1.054014000
H	3.354323000	4.092305000	1.589962000
H	4.358105000	2.676556000	1.828483000
C	4.844848000	5.563310000	−0.951801000
H	4.328889000	5.105128000	−1.804619000
H	5.682576000	6.143832000	−1.356842000
H	4.152948000	6.273401000	−0.482286000
C	6.409318000	5.121999000	1.675504000
H	5.781947000	5.846341000	2.208777000
H	7.277406000	5.665402000	1.283213000
H	6.779688000	4.398543000	2.411809000
C	6.583764000	3.037612000	−0.598756000
H	6.899495000	2.229446000	0.072415000
H	7.490989000	3.531458000	−0.967248000
H	6.088446000	2.582833000	−1.465509000
C	−3.733502000	1.975319000	1.289077000
C	−3.055038000	2.152692000	2.502450000
H	−2.053291000	1.748740000	2.626137000
C	−3.653780000	2.842370000	3.555445000
H	−3.115405000	2.977310000	4.489870000
C	−4.935738000	3.369328000	3.404602000
H	−5.400839000	3.913047000	4.222198000
C	−5.616785000	3.202482000	2.199193000
H	−6.615327000	3.612959000	2.075750000
C	−5.021611000	2.510885000	1.145446000
H	−5.558678000	2.397437000	0.207957000
C	−3.651695000	1.708067000	−1.610188000

C	−3.693630000	3.096042000	−1.814682000
H	−3.368913000	3.773463000	−1.028337000
C	−4.183390000	3.616093000	−3.010680000
H	−4.225939000	4.692752000	−3.152685000
C	−4.623455000	2.758449000	−4.019930000
H	−5.004169000	3.165497000	−4.952516000
C	−4.582388000	1.379407000	−3.823381000
H	−4.935044000	0.707051000	−4.600889000
C	−4.101438000	0.853651000	−2.623733000
H	−4.092465000	−0.221402000	−2.468829000
C	−3.539232000	−0.688957000	0.061123000
C	−2.688899000	−1.745093000	−0.366566000
C	−3.198725000	−3.057222000	−0.221543000
H	−2.582836000	−3.896854000	−0.526757000
C	−4.460855000	−3.303667000	0.300917000
H	−4.801382000	−4.332032000	0.395291000
C	−5.284156000	−2.253987000	0.713052000
H	−6.271656000	−2.444794000	1.121991000
C	−4.808413000	−0.954749000	0.591869000
H	−5.429194000	−0.125625000	0.920002000
C	−0.864772000	−2.481424000	−1.716930000
C	−1.622583000	−2.970778000	−2.799919000
H	−2.640484000	−2.613364000	−2.927515000
C	−1.097616000	−3.888706000	−3.700909000
H	−1.712212000	−4.236045000	−4.527602000
C	0.212201000	−4.349979000	−3.559614000
H	0.628974000	−5.062514000	−4.265225000
C	0.985595000	−3.870301000	−2.508435000
H	2.014252000	−4.203588000	−2.409643000
C	0.465131000	−2.952817000	−1.586782000
C	3.250902000	−2.377305000	−0.792251000
C	3.909609000	−3.615803000	−0.753063000
H	3.389974000	−4.498652000	−0.390438000
C	5.237622000	−3.718422000	−1.160984000
H	5.737233000	−4.682918000	−1.129148000

C	5.925873000	−2.587840000	−1.602768000
H	6.962810000	−2.670595000	−1.916605000
C	5.285200000	−1.350571000	−1.628801000
H	5.822410000	−0.464359000	−1.955453000
C	3.955362000	−1.246088000	−1.221516000
H	3.465152000	−0.276353000	−1.224211000
C	1.410377000	−3.367734000	1.168753000
C	0.443576000	−4.375234000	1.251840000
H	−0.271365000	−4.508486000	0.445057000
C	0.411835000	−5.231449000	2.354507000
H	−0.337736000	−6.017004000	2.402581000
C	1.347437000	−5.094292000	3.378874000
H	1.328889000	−5.770603000	4.229151000
C	2.317793000	−4.093175000	3.300696000
H	3.060651000	−3.990351000	4.087512000
C	2.346200000	−3.231974000	2.205993000
H	3.124858000	−2.474726000	2.141323000
C	0.945012000	−0.046231000	−2.871360000
C	1.018482000	1.278704000	−2.549113000
H	1.993536000	1.744502000	−2.408331000
C	−0.138867000	2.090861000	−2.289085000
C	−0.088975000	3.396616000	−1.860318000
H	−0.993288000	3.990028000	−1.802342000
H	0.847034000	3.941209000	−1.893465000
Y	−0.055572000	0.323537000	−0.213812000
H	−1.118572000	1.654593000	−2.506204000
O	−0.281970000	−0.326854000	2.171169000
C	0.636075000	−0.088878000	3.278886000
C	−1.308166000	−1.284752000	2.603147000
C	−0.101736000	−0.558338000	4.519578000
H	1.541588000	−0.679457000	3.104201000
H	0.890966000	0.972451000	3.289587000
C	−0.882247000	−1.757759000	3.983932000
H	−2.263151000	−0.752176000	2.625027000
H	−1.353408000	−2.080917000	1.861168000

H	−0.780676000	0.221369000	4.884086000
H	0.587369000	−0.816509000	5.328637000
H	−1.744846000	−2.022103000	4.602208000
H	−0.232291000	−2.634795000	3.905350000
H	1.835653000	−0.630221000	−3.074407000
H	−0.006219000	−0.510378000	−3.125641000
C	3.077755000	2.557360000	0.082685000
C	1.906386000	1.896132000	0.776447000
H	2.715262000	3.227371000	−0.707857000
H	3.693591000	1.800783000	−0.421107000
H	2.202917000	1.127505000	1.496578000
C	0.729390000	2.601972000	1.089292000
C	0.240534000	3.793991000	0.565997000
H	0.029833000	2.107364000	1.778284000
H	−0.731506000	4.154935000	0.887739000
H	0.918714000	4.543480000	0.171635000

117

theta3_thf_P2_trans_1,4 E = −2200.683403 a.u.

N	2.022624000	1.046643000	−0.650057000
P	2.381633000	−1.874901000	−0.039528000
P	−0.453241000	2.758201000	−0.238233000
Si	−7.039752000	−2.685919000	0.099420000
C	−5.356660000	−2.337355000	0.931929000
H	−5.107687000	−3.189110000	1.581038000
H	−5.483630000	−1.474973000	1.604158000
C	−6.872481000	−4.156933000	−1.072269000
H	−6.174235000	−3.953491000	−1.893319000
H	−7.840307000	−4.403655000	−1.525270000
H	−6.520258000	−5.053242000	−0.547235000
C	−8.286786000	−3.073988000	1.459073000
H	−7.992306000	−3.961030000	2.032580000
H	−9.279631000	−3.270838000	1.037070000
H	−8.389002000	−2.240449000	2.164370000
C	−7.585474000	−1.155838000	−0.865502000
H	−7.667971000	−0.276833000	−0.214415000

H	−8.569492000	−1.317239000	−1.322075000
H	−6.890239000	−0.909528000	−1.677588000
C	2.653770000	−3.193557000	1.223946000
C	1.866255000	−3.181796000	2.383832000
H	1.110741000	−2.410282000	2.515938000
C	2.046430000	−4.147517000	3.372348000
H	1.428868000	−4.130239000	4.266668000
C	3.011101000	−5.141136000	3.208287000
H	3.148267000	−5.898609000	3.975064000
C	3.795552000	−5.163419000	2.055674000
H	4.547676000	−5.936525000	1.923481000
C	3.620249000	−4.196335000	1.066811000
H	4.231625000	−4.226731000	0.169472000
C	3.008266000	−2.620062000	−1.612321000
C	2.440821000	−3.816605000	−2.077344000
H	1.683482000	−4.327612000	−1.487409000
C	2.858031000	−4.371588000	−3.284337000
H	2.419987000	−5.304720000	−3.628540000
C	3.835370000	−3.731775000	−4.048239000
H	4.157966000	−4.163801000	−4.991500000
C	4.399517000	−2.541557000	−3.594091000
H	5.167241000	−2.043623000	−4.180309000
C	3.991781000	−1.987202000	−2.380309000
H	4.450382000	−1.069428000	−2.024628000
C	3.591650000	−0.551470000	0.355884000
C	3.260556000	0.767508000	−0.053429000
C	4.206393000	1.777377000	0.230184000
H	3.993626000	2.798049000	−0.071555000
C	5.397625000	1.496324000	0.887453000
H	6.092706000	2.307447000	1.090660000
C	5.703837000	0.196544000	1.295064000
H	6.634154000	−0.021490000	1.810741000
C	4.792956000	−0.818096000	1.022525000
H	5.014533000	−1.837052000	1.328937000
C	1.959469000	2.150878000	−1.527769000

C	2.939649000	2.347278000	−2.521174000
H	3.777263000	1.657330000	−2.568621000
C	2.844918000	3.382630000	−3.441184000
H	3.616147000	3.495896000	−4.198847000
C	1.757714000	4.258456000	−3.411089000
H	1.674030000	5.062807000	−4.135956000
C	0.774533000	4.083080000	−2.444453000
H	−0.081722000	4.750520000	−2.428647000
C	0.867423000	3.056589000	−1.495684000
C	−1.973801000	3.628280000	−0.825182000
C	−2.077036000	5.027598000	−0.801776000
H	−1.249719000	5.628846000	−0.434345000
C	−3.243665000	5.654876000	−1.233227000
H	−3.309555000	6.739390000	−1.215355000
C	−4.324719000	4.894970000	−1.680852000
H	−5.234420000	5.386684000	−2.014402000
C	−4.238838000	3.503981000	−1.690121000
H	−5.082148000	2.907371000	−2.027702000
C	−3.070642000	2.874478000	−1.261661000
H	−3.011793000	1.789317000	−1.268019000
C	0.025801000	3.799883000	1.220948000
C	1.300657000	4.359898000	1.353186000
H	2.033854000	4.226266000	0.563460000
C	1.626315000	5.112512000	2.482990000
H	2.617763000	5.549245000	2.569861000
C	0.680632000	5.320725000	3.485244000
H	0.931854000	5.918297000	4.357309000
C	−0.596536000	4.770367000	3.357510000
H	−1.344525000	4.943275000	4.127133000
C	−0.919729000	4.008192000	2.236861000
H	−1.924840000	3.603400000	2.136148000
C	−0.768158000	0.523339000	−2.654235000
C	−1.335484000	−0.747157000	−2.469259000
H	−2.423749000	−0.787403000	−2.357519000
C	−0.637096000	−1.901561000	−2.128635000

C	−1.365279000	−3.184575000	−1.803924000
H	−0.933549000	−4.022703000	−2.365905000
H	−2.401094000	−3.093808000	−2.154309000
Y	0.051540000	−0.088521000	−0.335679000
H	0.403165000	−1.984604000	−2.454404000
O	0.101132000	0.102133000	2.134722000
C	−0.961739000	0.092869000	3.124890000
C	1.318274000	0.665171000	2.737255000
C	−0.261262000	0.217971000	4.467238000
H	−1.624656000	0.944706000	2.931376000
H	−1.520221000	−0.838716000	3.011196000
C	0.915278000	1.133766000	4.127837000
H	2.062206000	−0.135603000	2.761659000
H	1.671686000	1.469423000	2.091201000
H	0.090510000	−0.762527000	4.808101000
H	−0.921118000	0.629500000	5.236188000
H	1.743078000	1.046437000	4.837057000
H	0.594226000	2.179588000	4.101635000
H	−1.389612000	1.353260000	−2.972911000
H	0.269737000	0.584708000	−2.998920000
C	−4.183994000	−2.074560000	−0.032627000
C	−2.914028000	−1.781550000	0.711817000
H	−4.046173000	−2.932408000	−0.698647000
H	−4.431726000	−1.216052000	−0.675505000
H	−3.011929000	−0.993703000	1.462468000
C	−1.735480000	−2.428899000	0.619635000
C	−1.384158000	−3.569871000	−0.306229000
H	−0.959269000	−2.155023000	1.343417000
H	−0.397941000	−3.950199000	−0.015963000
H	−2.085754000	−4.403223000	−0.158920000

117

theta3_thf_C2_trans_1,2 E = −2200.650324 a.u.

N	1.160915000	−1.201328000	1.025690000
P	2.791532000	1.000624000	−0.170698000
P	−1.651549000	−2.052597000	0.258224000

Si	−5.050793000	4.535713000	−0.797759000
C	−4.001400000	3.248153000	−1.733530000
H	−3.480512000	3.766679000	−2.551562000
H	−4.681839000	2.533821000	−2.221163000
C	−3.915747000	5.846973000	−0.046533000
H	−3.243873000	5.425991000	0.711796000
H	−4.499286000	6.636984000	0.441565000
H	−3.295849000	6.324730000	−0.815029000
C	−6.231177000	5.338578000	−2.031318000
H	−5.688682000	5.829066000	−2.848495000
H	−6.852536000	6.100770000	−1.545665000
H	−6.907630000	4.600318000	−2.478667000
C	−6.040882000	3.693684000	0.575558000
H	−6.715224000	2.927637000	0.172782000
H	−6.661052000	4.422771000	1.111072000
H	−5.391052000	3.214260000	1.317955000
C	3.860913000	1.561847000	−1.565772000
C	3.445389000	1.311098000	−2.880651000
H	2.493702000	0.816837000	−3.062113000
C	4.235541000	1.705254000	−3.959151000
H	3.902462000	1.509775000	−4.975146000
C	5.443989000	2.363744000	−3.734400000
H	6.057022000	2.678015000	−4.574598000
C	5.858577000	2.628599000	−2.429542000
H	6.795871000	3.148442000	−2.250405000
C	5.072130000	2.232558000	−1.348811000
H	5.398033000	2.455088000	−0.336431000
C	3.193603000	2.203127000	1.175461000
C	2.775690000	3.534824000	1.025264000
H	2.237617000	3.839781000	0.131005000
C	3.070436000	4.478968000	2.006317000
H	2.753571000	5.510405000	1.875503000
C	3.779931000	4.102126000	3.148834000
H	4.015519000	4.840378000	3.910732000
C	4.194684000	2.780413000	3.303114000

H	4.756778000	2.484847000	4.185229000
C	3.902746000	1.831282000	2.322348000
H	4.237423000	0.805148000	2.444340000
C	3.461292000	−0.610454000	0.380366000
C	2.528591000	−1.512723000	0.958186000
C	3.040245000	−2.762209000	1.378147000
H	2.366068000	−3.483058000	1.830226000
C	4.380231000	−3.094347000	1.225598000
H	4.722731000	−4.072039000	1.556226000
C	5.281080000	−2.199463000	0.644072000
H	6.326944000	−2.462812000	0.518594000
C	4.807992000	−0.961376000	0.225269000
H	5.490431000	−0.252569000	−0.236740000
C	0.437433000	−1.741958000	2.099912000
C	0.991568000	−1.826089000	3.398118000
H	2.020444000	−1.512095000	3.544707000
C	0.250833000	−2.274890000	4.482154000
H	0.718382000	−2.321633000	5.463023000
C	−1.087732000	−2.645212000	4.326562000
H	−1.674550000	−2.984839000	5.174813000
C	−1.657072000	−2.578651000	3.060246000
H	−2.696155000	−2.864046000	2.928767000
C	−0.914978000	−2.154508000	1.948724000
C	−3.476768000	−2.178587000	0.490666000
C	−4.100356000	−3.378663000	0.867644000
H	−3.508899000	−4.278389000	1.014939000
C	−5.481313000	−3.425936000	1.041704000
H	−5.955204000	−4.359043000	1.334344000
C	−6.254809000	−2.281953000	0.837206000
H	−7.332183000	−2.323425000	0.972139000
C	−5.645333000	−1.091047000	0.447988000
H	−6.244398000	−0.201459000	0.274159000
C	−4.262627000	−1.042312000	0.270296000
H	−3.791064000	−0.122544000	−0.063063000
C	−1.256912000	−3.680521000	−0.539455000

C	−0.220556000	−4.500896000	−0.083225000
H	0.352458000	−4.211483000	0.791992000
C	0.060598000	−5.705956000	−0.729355000
H	0.861710000	−6.339730000	−0.358100000
C	−0.692372000	−6.105146000	−1.832794000
H	−0.479606000	−7.049565000	−2.326251000
C	−1.731300000	−5.293214000	−2.291381000
H	−2.334433000	−5.605154000	−3.140131000
C	−2.009390000	−4.086260000	−1.652727000
H	−2.840233000	−3.476627000	−2.001672000
C	−1.208250000	1.635530000	1.806457000
C	−0.637680000	1.262785000	2.976287000
H	−0.961073000	0.333543000	3.444307000
C	0.356615000	2.033068000	3.695309000
C	0.789994000	1.673639000	4.913506000
H	1.509672000	2.274962000	5.460353000
H	0.429309000	0.769981000	5.398849000
Y	0.025645000	0.296507000	−0.417134000
H	0.730264000	2.942170000	3.227173000
O	0.572232000	−1.068736000	−2.325948000
C	−0.298535000	−1.355570000	−3.461091000
C	1.688814000	−2.026595000	−2.321857000
C	0.549975000	−2.181500000	−4.410476000
H	−1.160857000	−1.918887000	−3.091844000
H	−0.634607000	−0.406326000	−3.879260000
C	1.390013000	−3.012904000	−3.440587000
H	2.603627000	−1.455072000	−2.502072000
H	1.731228000	−2.482950000	−1.333343000
H	1.184407000	−1.531985000	−5.024706000
H	−0.064440000	−2.791018000	−5.078992000
H	2.308566000	−3.399852000	−3.890757000
H	0.809487000	−3.858956000	−3.060019000
H	−2.070254000	1.090795000	1.421649000
H	−0.994829000	2.608576000	1.366510000
C	−2.964962000	2.475881000	−0.892076000

C	−2.131235000	1.556754000	−1.770404000
H	−2.315183000	3.196708000	−0.382020000
H	−3.495690000	1.932033000	−0.099446000
H	−2.723895000	0.913470000	−2.423186000
C	−0.879144000	1.926073000	−2.262556000
C	0.154044000	2.599769000	−1.584970000
H	−0.572249000	1.456971000	−3.200676000
H	1.038454000	2.893273000	−2.143139000
H	−0.092426000	3.261610000	−0.750278000

117

theta3_thf_TS2_trans_1,2 E = −2200.624523 a.u. Imaginary frequency=294i

N	−2.129692000	0.756274000	−0.697989000
P	0.004012000	2.832979000	−0.097630000
P	−2.050268000	−2.211730000	−0.120012000
Si	7.545232000	−1.892208000	0.316709000
C	5.748328000	−2.264881000	0.833944000
H	5.479926000	−1.592293000	1.661331000
H	5.715518000	−3.281313000	1.254003000
C	7.665211000	−0.113381000	−0.312894000
H	7.065679000	0.043346000	−1.218140000
H	8.701955000	0.142659000	−0.562852000
H	7.323906000	0.604026000	0.443688000
C	8.648767000	−2.107800000	1.832351000
H	8.360219000	−1.425821000	2.641368000
H	9.698453000	−1.903536000	1.588756000
H	8.596262000	−3.130230000	2.225614000
C	8.085077000	−3.085775000	−1.044561000
H	8.015140000	−4.129587000	−0.714954000
H	9.127995000	−2.902850000	−1.330666000
H	7.477191000	−2.981294000	−1.951574000
C	0.733223000	3.822341000	1.285281000
C	1.022178000	3.183711000	2.498484000
H	0.825342000	2.120515000	2.605836000
C	1.563564000	3.899056000	3.565222000
H	1.789511000	3.391544000	4.499570000

C	1.828233000	5.261127000	3.429317000
H	2.255873000	5.818602000	4.258094000
C	1.552270000	5.903566000	2.222904000
H	1.762546000	6.963646000	2.109262000
C	1.010893000	5.190347000	1.154564000
H	0.815837000	5.698534000	0.214430000
C	0.549031000	3.775066000	−1.591092000
C	1.923823000	3.836906000	−1.867035000
H	2.638969000	3.380962000	−1.185280000
C	2.382850000	4.494793000	−3.005014000
H	3.449755000	4.544677000	−3.205932000
C	1.475145000	5.089172000	−3.884173000
H	1.833878000	5.600072000	−4.773431000
C	0.109375000	5.028824000	−3.615307000
H	−0.600807000	5.495615000	−4.292704000
C	−0.355627000	4.375130000	−2.472530000
H	−1.421332000	4.339164000	−2.265967000
C	−1.814976000	3.086948000	0.012491000
C	−2.658595000	2.008883000	−0.364515000
C	−4.051693000	2.234928000	−0.311545000
H	−4.723890000	1.430379000	−0.594350000
C	−4.577543000	3.450647000	0.103269000
H	−5.656781000	3.578537000	0.136276000
C	−3.737770000	4.497712000	0.491225000
H	−4.148595000	5.445958000	0.824549000
C	−2.362701000	4.303398000	0.443040000
H	−1.696746000	5.107944000	0.743933000
C	−2.765920000	0.001123000	−1.692069000
C	−3.329119000	0.599393000	−2.842396000
H	−3.338363000	1.683008000	−2.912667000
C	−3.840743000	−0.163265000	−3.882199000
H	−4.257510000	0.336354000	−4.753131000
C	−3.800718000	−1.559325000	−3.829139000
H	−4.187392000	−2.156976000	−4.649074000
C	−3.261794000	−2.175592000	−2.707134000

H	−3.231262000	−3.259691000	−2.657793000
C	−2.767562000	−1.422988000	−1.630493000
C	−1.867577000	−4.008155000	−0.494456000
C	−2.982319000	−4.845160000	−0.659560000
H	−3.986694000	−4.438309000	−0.575971000
C	−2.807630000	−6.202582000	−0.916180000
H	−3.676247000	−6.842425000	−1.045778000
C	−1.522645000	−6.741736000	−0.998400000
H	−1.390077000	−7.802207000	−1.194247000
C	−0.411087000	−5.922184000	−0.813925000
H	0.589842000	−6.343283000	−0.858567000
C	−0.582984000	−4.561323000	−0.559345000
H	0.286556000	−3.930377000	−0.393049000
C	−3.416240000	−2.220844000	1.129020000
C	−4.610409000	−1.518340000	0.942270000
H	−4.770694000	−0.956217000	0.027368000
C	−5.608565000	−1.560050000	1.917015000
H	−6.535892000	−1.015732000	1.759387000
C	−5.426661000	−2.308865000	3.078156000
H	−6.209819000	−2.348023000	3.830247000
C	−4.240188000	−3.020578000	3.265663000
H	−4.099226000	−3.620976000	4.160684000
C	−3.236641000	−2.971804000	2.300508000
H	−2.324586000	−3.546961000	2.447031000
C	2.226762000	−2.508188000	−1.929306000
C	1.176855000	−1.653789000	−2.221353000
H	0.171713000	−2.077124000	−2.324098000
C	1.374857000	−0.281481000	−2.590539000
C	0.376405000	0.604045000	−2.890781000
H	0.603794000	1.625374000	−3.171622000
H	−0.641688000	0.263377000	−3.080311000
Y	−0.018310000	−0.088184000	−0.248767000
H	2.401134000	0.084346000	−2.546732000
O	−0.579815000	0.047431000	2.152184000
C	0.001831000	−0.722550000	3.240642000

C	−1.779741000	0.740766000	2.639004000
C	−0.599272000	−0.143498000	4.510059000
H	−0.277341000	−1.774830000	3.105101000
H	1.088079000	−0.625705000	3.182896000
C	−2.010338000	0.226583000	4.051977000
H	−1.563930000	1.812679000	2.618901000
H	−2.592371000	0.518934000	1.948108000
H	−0.044490000	0.747393000	4.825464000
H	−0.585237000	−0.862821000	5.333794000
H	−2.480509000	0.985046000	4.684171000
H	−2.655196000	−0.657461000	4.038176000
H	2.058084000	−3.576669000	−1.852638000
H	3.234383000	−2.205198000	−2.187327000
C	4.690727000	−2.141515000	−0.281045000
C	3.303961000	−2.471486000	0.213700000
H	4.714624000	−1.127350000	−0.700603000
H	4.962509000	−2.821408000	−1.101380000
H	3.153598000	−3.517177000	0.483908000
C	2.467332000	−1.554765000	0.836919000
C	2.392943000	−0.153410000	0.664048000
H	1.696038000	−2.007286000	1.474472000
H	2.031370000	0.427694000	1.518885000
H	3.196545000	0.353785000	0.132103000

117

theta3_thf_P2_trans_1,2 E = −2200.68143 a.u.

N	2.041952000	1.191247000	−0.414270000
P	2.632043000	−1.800326000	−0.096145000
P	−0.585531000	2.643486000	−0.155887000
Si	−7.302708000	−2.941035000	0.201347000
C	−5.584016000	−2.122564000	0.357128000
H	−5.056321000	−2.636047000	1.174240000
H	−5.736974000	−1.085875000	0.696034000
C	−7.096124000	−4.745610000	−0.313246000
H	−6.629225000	−4.841892000	−1.300800000
H	−8.069619000	−5.248106000	−0.365096000

H	−6.479648000	−5.302035000	0.403500000
C	−8.144737000	−2.847267000	1.887947000
H	−7.570676000	−3.379595000	2.656162000
H	−9.142935000	−3.300247000	1.853794000
H	−8.268331000	−1.810170000	2.222779000
C	−8.332655000	−2.016844000	−1.083508000
H	−8.456426000	−0.959772000	−0.817961000
H	−9.335818000	−2.453416000	−1.160739000
H	−7.883669000	−2.060692000	−2.083099000
C	2.879697000	−3.141417000	1.156048000
C	1.994558000	−3.227861000	2.239400000
H	1.182428000	−2.510488000	2.326049000
C	2.149277000	−4.221278000	3.204640000
H	1.455788000	−4.280022000	4.039818000
C	3.186822000	−5.146424000	3.093466000
H	3.304953000	−5.925297000	3.841735000
C	4.067591000	−5.073063000	2.015234000
H	4.875564000	−5.793690000	1.921679000
C	3.916128000	−4.078684000	1.049596000
H	4.601000000	−4.039049000	0.207327000
C	3.452304000	−2.491969000	−1.599576000
C	2.874181000	−3.616311000	−2.207524000
H	1.993565000	−4.082022000	−1.769009000
C	3.431294000	−4.155027000	−3.363973000
H	2.980875000	−5.030837000	−3.823481000
C	4.565001000	−3.569907000	−3.931464000
H	4.997424000	−3.987626000	−4.836450000
C	5.141212000	−2.451526000	−3.332955000
H	6.026798000	−1.995963000	−3.768009000
C	4.589780000	−1.912543000	−2.169045000
H	5.048567000	−1.045669000	−1.702654000
C	3.697407000	−0.406230000	0.463020000
C	3.279625000	0.922932000	0.180579000
C	4.142124000	1.964577000	0.591191000
H	3.853022000	2.990998000	0.387515000

C	5.332295000	1.709141000	1.256838000
H	5.959573000	2.543310000	1.561662000
C	5.719585000	0.399099000	1.551128000
H	6.647786000	0.197880000	2.077494000
C	4.895261000	−0.645342000	1.151951000
H	5.184764000	−1.669845000	1.370499000
C	1.948769000	2.277891000	−1.297321000
C	2.975671000	2.587808000	−2.214108000
H	3.891409000	2.004683000	−2.187795000
C	2.825757000	3.597273000	−3.154756000
H	3.635582000	3.800781000	−3.850881000
C	1.639660000	4.332227000	−3.227749000
H	1.517274000	5.114219000	−3.971213000
C	0.613523000	4.049720000	−2.334390000
H	−0.310810000	4.617161000	−2.385260000
C	0.756386000	3.050378000	−1.361094000
C	−2.085471000	3.543006000	−0.751732000
C	−2.204365000	4.934945000	−0.617948000
H	−1.406191000	5.507402000	−0.153159000
C	−3.348282000	5.588452000	−1.070107000
H	−3.429548000	6.666805000	−0.963264000
C	−4.386915000	4.862667000	−1.654902000
H	−5.278673000	5.375390000	−2.004902000
C	−4.281827000	3.479009000	−1.781474000
H	−5.091667000	2.908342000	−2.228152000
C	−3.138143000	2.822431000	−1.327800000
H	−3.066974000	1.742804000	−1.416373000
C	−0.167456000	3.601538000	1.373756000
C	1.028800000	4.310967000	1.516921000
H	1.751377000	4.322287000	0.706410000
C	1.284353000	5.029373000	2.686385000
H	2.214832000	5.582329000	2.784505000
C	0.344731000	5.055130000	3.715009000
H	0.540597000	5.625294000	4.618986000
C	−0.856646000	4.357313000	3.574227000

H	−1.601458000	4.387875000	4.365403000
C	−1.108449000	3.627775000	2.414262000
H	−2.056026000	3.103290000	2.306252000
C	−2.739629000	−1.292306000	−2.253444000
C	−1.456286000	−0.487840000	−2.333057000
H	−1.575693000	0.578302000	−2.556352000
C	−0.282193000	−1.080869000	−2.853863000
C	0.949459000	−0.464701000	−2.971200000
H	1.817367000	−1.020482000	−3.306992000
H	1.020857000	0.621413000	−3.054164000
Y	0.218535000	−0.205363000	−0.433699000
H	−0.288183000	−2.171109000	−2.959288000
O	0.190176000	−0.208275000	2.045542000
C	−0.906969000	−0.537996000	2.939623000
C	1.258470000	0.447240000	2.811791000
C	−0.355462000	−0.381513000	4.348329000
H	−1.730364000	0.158434000	2.740816000
H	−1.232599000	−1.555746000	2.714884000
C	0.658445000	0.749023000	4.174639000
H	2.094821000	−0.255090000	2.872991000
H	1.565308000	1.332696000	2.255807000
H	0.142315000	−1.303472000	4.669975000
H	−1.142839000	−0.151115000	5.071657000
H	1.422063000	0.762075000	4.957361000
H	0.157250000	1.721670000	4.165846000
H	−3.487473000	−0.891904000	−2.952809000
H	−2.546266000	−2.318490000	−2.600503000
C	−4.741885000	−2.133854000	−0.923010000
C	−3.395487000	−1.361059000	−0.866305000
H	−4.525627000	−3.168087000	−1.224003000
H	−5.318805000	−1.689683000	−1.745500000
H	−3.640421000	−0.334427000	−0.545302000
C	−2.512806000	−1.945337000	0.198996000
C	−1.394911000	−2.680486000	0.043689000
H	−2.871491000	−1.789890000	1.216726000

H	−0.897510000	−3.141848000	0.894539000
H	−1.017300000	−2.956552000	−0.939240000

117

theta1_thf_C2_cis_1,4 E = −2200.634694 a.u.

N	1.425823000	0.418655000	1.325245000
P	−0.091540000	2.779006000	0.246947000
P	1.869771000	−2.330964000	0.155244000
Si	−7.419621000	−1.696926000	−0.477403000
C	−5.827557000	−2.427897000	0.262510000
H	−5.934027000	−3.522723000	0.295572000
H	−5.753741000	−2.107957000	1.312157000
C	−7.649701000	−2.301418000	−2.253962000
H	−6.821002000	−1.993878000	−2.903479000
H	−8.572211000	−1.897464000	−2.688615000
H	−7.718301000	−3.395216000	−2.298835000
C	−8.881821000	−2.250416000	0.580003000
H	−8.973750000	−3.343180000	0.594209000
H	−9.826601000	−1.845854000	0.197222000
H	−8.775398000	−1.914275000	1.618521000
C	−7.313171000	0.193306000	−0.469060000
H	−7.159705000	0.578393000	0.546684000
H	−8.237263000	0.640488000	−0.855032000
H	−6.488889000	0.558299000	−1.094609000
C	−0.121137000	4.272138000	−0.836521000
C	0.440818000	4.179670000	−2.117675000
H	0.875847000	3.238930000	−2.448634000
C	0.453067000	5.284749000	−2.965963000
H	0.891602000	5.204214000	−3.957373000
C	−0.106348000	6.491905000	−2.546341000
H	−0.103506000	7.352513000	−3.209517000
C	−0.673939000	6.589866000	−1.276773000
H	−1.112245000	7.527842000	−0.946837000
C	−0.682682000	5.487091000	−0.423052000
H	−1.131581000	5.571337000	0.562543000
C	−1.412800000	3.089012000	1.497427000

C	−2.749882000	3.096222000	1.074313000
H	−2.991833000	2.951822000	0.023925000
C	−3.776414000	3.303949000	1.991162000
H	−4.808831000	3.314080000	1.652393000
C	−3.478837000	3.493333000	3.341753000
H	−4.280584000	3.649978000	4.058101000
C	−2.152526000	3.483789000	3.768190000
H	−1.916127000	3.637310000	4.817717000
C	−1.119994000	3.284598000	2.851126000
H	−0.087793000	3.289748000	3.188764000
C	1.488821000	2.869950000	1.177049000
C	2.025926000	1.644346000	1.650753000
C	3.218053000	1.715134000	2.402652000
H	3.650999000	0.795713000	2.784797000
C	3.848669000	2.927466000	2.651148000
H	4.770295000	2.937037000	3.228013000
C	3.321489000	4.124437000	2.159325000
H	3.818558000	5.070690000	2.350762000
C	2.141521000	4.084392000	1.424999000
H	1.712556000	5.006783000	1.041746000
C	1.449508000	−0.595474000	2.302508000
C	1.225032000	−0.313546000	3.665005000
H	1.061089000	0.717290000	3.964431000
C	1.192880000	−1.323237000	4.617925000
H	1.007751000	−1.066634000	5.657953000
C	1.376125000	−2.658046000	4.249872000
H	1.345366000	−3.447785000	4.994493000
C	1.591862000	−2.964560000	2.911470000
H	1.729195000	−3.999341000	2.610387000
C	1.640241000	−1.954932000	1.940882000
C	1.577381000	−4.146752000	−0.018043000
C	2.621610000	−5.072207000	0.119672000
H	3.631408000	−4.731261000	0.328061000
C	2.371276000	−6.437267000	−0.011405000
H	3.188281000	−7.145392000	0.097943000

C	1.080774000	−6.892388000	−0.278665000
H	0.889039000	−7.956940000	−0.381614000
C	0.037012000	−5.977630000	−0.412254000
H	−0.971875000	−6.325831000	−0.616958000
C	0.282234000	−4.611317000	−0.287183000
H	−0.544728000	−3.912746000	−0.382055000
C	3.681875000	−2.167775000	−0.174775000
C	4.598062000	−1.812400000	0.820233000
H	4.250556000	−1.630779000	1.833279000
C	5.957709000	−1.713570000	0.519272000
H	6.662875000	−1.442674000	1.300573000
C	6.412947000	−1.979959000	−0.770891000
H	7.473415000	−1.915892000	−0.998864000
C	5.503811000	−2.341760000	−1.767550000
H	5.855835000	−2.567050000	−2.771107000
C	4.144287000	−2.425317000	−1.474376000
H	3.444110000	−2.723675000	−2.252726000
C	−1.842180000	−0.591026000	0.538497000
C	−2.220824000	−2.008592000	0.674707000
H	−1.499166000	−2.623268000	1.219251000
C	−3.310800000	−2.661425000	0.209916000
C	−4.516646000	−2.066496000	−0.463580000
H	−4.582957000	−2.415371000	−1.506554000
H	−4.418210000	−0.973544000	−0.509701000
Y	0.232516000	−0.002094000	−0.585303000
H	−3.380004000	−3.731915000	0.411633000
O	2.050855000	0.792453000	−2.150784000
C	2.330902000	0.650143000	−3.569772000
C	3.234241000	1.364670000	−1.489103000
C	3.550090000	1.516261000	−3.839725000
H	2.533224000	−0.407375000	−3.775426000
H	1.445402000	0.964187000	−4.126223000
C	4.335090000	1.367661000	−2.537368000
H	2.969235000	2.374643000	−1.164446000
H	3.461264000	0.749945000	−0.617308000

H 3.253965000 2.559441000 −3.999128000

H 4.103221000 1.180543000 −4.721537000

H 5.048149000 2.179709000 −2.370024000

H 4.882673000 0.420124000 −2.520741000

H −2.696039000 0.064664000 0.336142000

H −1.397317000 −0.236274000 1.488596000

C −0.892009000 −2.027088000 −2.566689000

C −0.620856000 −0.931924000 −3.311742000

H −1.624272000 −2.042108000 −1.756675000

H −0.414020000 −2.974189000 −2.799258000

H 0.100550000 −1.027225000 −4.120993000

C −1.171034000 0.407973000 −3.099861000

C −2.268203000 0.692226000 −2.363439000

H −0.653855000 1.233878000 −3.587005000

H −2.596376000 1.719880000 −2.238455000

H −2.902468000 −0.083068000 −1.948581000

117

theta1_thf_TS2_cis_1,4 E = −2200.62655 a.u. Imaginary frequency=96i

N 1.276098000 0.542370000 1.338510000

P −0.627593000 2.628583000 0.244220000

P 2.289255000 −2.026958000 0.087874000

Si −7.176795000 −2.327214000 −0.604726000

C −5.609164000 −2.836316000 0.346551000

H −5.750258000 −3.866907000 0.705521000

H −5.529704000 −2.217806000 1.252387000

C −7.404498000 −3.435201000 −2.118103000

H −6.574880000 −3.337774000 −2.828834000

H −8.325065000 −3.175733000 −2.654976000

H −7.477728000 −4.492400000 −1.835318000

C −8.658092000 −2.503898000 0.550595000

H −8.775873000 −3.538390000 0.894914000

H −9.589443000 −2.217375000 0.047262000

H −8.554552000 −1.869553000 1.439288000

C −7.024145000 −0.526020000 −1.166794000

H −6.869067000 0.145672000 −0.313332000

H	−7.935444000	−0.198079000	−1.681325000
H	−6.188695000	−0.384694000	−1.863872000
C	−0.846136000	4.153770000	−0.769273000
C	−0.173421000	4.245112000	−1.996039000
H	0.439025000	3.414431000	−2.341175000
C	−0.288381000	5.389918000	−2.781803000
H	0.237151000	5.451336000	−3.731308000
C	−1.087516000	6.450914000	−2.355289000
H	−1.184388000	7.340982000	−2.970782000
C	−1.766963000	6.363835000	−1.141110000
H	−2.392991000	7.186696000	−0.806793000
C	−1.648420000	5.222181000	−0.348754000
H	−2.185371000	5.162167000	0.593667000
C	−2.100800000	2.596115000	1.354300000
C	−3.365527000	2.392933000	0.782148000
H	−3.469979000	2.293678000	−0.296485000
C	−4.499598000	2.342348000	1.588029000
H	−5.476492000	2.196689000	1.135148000
C	−4.380452000	2.479053000	2.972201000
H	−5.265523000	2.435389000	3.600903000
C	−3.125768000	2.676460000	3.544983000
H	−3.029611000	2.790016000	4.621456000
C	−1.987055000	2.738070000	2.740961000
H	−1.013471000	2.908106000	3.191516000
C	0.817353000	2.961583000	1.325701000
C	1.569650000	1.843349000	1.773509000
C	2.669107000	2.114984000	2.619390000
H	3.264659000	1.283773000	2.984494000
C	3.007096000	3.410698000	2.984456000
H	3.866973000	3.572015000	3.630168000
C	2.266807000	4.502486000	2.522045000
H	2.533924000	5.516190000	2.805021000
C	1.177187000	4.265952000	1.693329000
H	0.589295000	5.102659000	1.324836000
C	1.458817000	−0.493433000	2.273200000

C	1.119145000	−0.329355000	3.632770000
H	0.731232000	0.630095000	3.961711000
C	1.262316000	−1.363323000	4.548425000
H	0.982502000	−1.196701000	5.585679000
C	1.744332000	−2.610462000	4.145836000
H	1.856610000	−3.420120000	4.860672000
C	2.069759000	−2.804572000	2.808477000
H	2.434393000	−3.773439000	2.479097000
C	1.935637000	−1.768542000	1.874438000
C	2.270429000	−3.866752000	−0.122241000
C	3.442642000	−4.633238000	−0.166408000
H	4.414051000	−4.157292000	−0.078695000
C	3.373450000	−6.018487000	−0.316682000
H	4.291541000	−6.599058000	−0.349194000
C	2.138462000	−6.655589000	−0.416512000
H	2.088319000	−7.734575000	−0.533960000
C	0.966709000	−5.901659000	−0.357412000
H	−0.001959000	−6.389911000	−0.423422000
C	1.031645000	−4.517738000	−0.214221000
H	0.108646000	−3.950303000	−0.145067000
C	4.072950000	−1.585875000	−0.140692000
C	4.825347000	−0.987201000	0.874741000
H	4.373643000	−0.808549000	1.846039000
C	6.160430000	−0.644235000	0.653779000
H	6.736620000	−0.186452000	1.453593000
C	6.760201000	−0.906598000	−0.577068000
H	7.804038000	−0.653035000	−0.740937000
C	6.018454000	−1.512062000	−1.593709000
H	6.484374000	−1.737636000	−2.549576000
C	4.680991000	−1.840302000	−1.380428000
H	4.119144000	−2.335371000	−2.170544000
C	−1.572399000	−1.188754000	0.198387000
C	−1.983537000	−2.510174000	0.671743000
H	−1.275011000	−2.997028000	1.342759000
C	−3.110953000	−3.195063000	0.380066000

C	−4.286469000	−2.750930000	−0.440952000
H	−4.363898000	−3.386045000	−1.336600000
H	−4.139086000	−1.725729000	−0.806060000
Y	0.353453000	0.091971000	−0.755221000
H	−3.221365000	−4.181623000	0.832814000
O	2.239277000	1.106274000	−1.944505000
C	2.853509000	0.766709000	−3.218382000
C	3.122254000	2.034320000	−1.221479000
C	3.855203000	1.875390000	−3.484847000
H	3.347688000	−0.204428000	−3.112306000
H	2.061941000	0.695384000	−3.964701000
C	4.374542000	2.167403000	−2.076456000
H	2.582724000	2.980536000	−1.120617000
H	3.308630000	1.616029000	−0.232264000
H	3.354765000	2.753383000	−3.909526000
H	4.640333000	1.560043000	−4.177742000
H	4.824192000	3.159624000	−1.980797000
H	5.119484000	1.421493000	−1.781897000
H	−2.407990000	−0.561809000	−0.136106000
H	−1.057242000	−0.657226000	1.027081000
C	−1.016689000	−1.940682000	−2.105584000
C	−0.212938000	−1.195278000	−2.955576000
H	−2.085170000	−1.766495000	−2.062873000
H	−0.698876000	−2.925365000	−1.785589000
H	0.728961000	−1.613733000	−3.306105000
C	−0.589172000	0.127692000	−3.394649000
C	−1.564948000	0.896888000	−2.831362000
H	0.020711000	0.587229000	−4.170321000
H	−1.753910000	1.905170000	−3.184166000
H	−2.282191000	0.496240000	−2.117320000

117

theta1_thf_P2_cis_1,4 E = −2200.678069 a.u.

N	−1.569273000	−1.255644000	0.947001000
P	1.331235000	−1.556273000	0.627929000
P	−2.829726000	1.335565000	0.140503000

Si	6.825114000	1.921427000	−0.985405000
C	5.334531000	2.527557000	0.036089000
H	5.681622000	3.362602000	0.663410000
H	5.028546000	1.737858000	0.737419000
C	7.569663000	3.381778000	−1.924274000
H	6.852582000	3.835817000	−2.619032000
H	8.437655000	3.064909000	−2.515163000
H	7.909768000	4.167483000	−1.238765000
C	8.106007000	1.200006000	0.198095000
H	8.438255000	1.944781000	0.931314000
H	8.994395000	0.854571000	−0.344332000
H	7.704095000	0.343846000	0.753370000
C	6.268430000	0.598718000	−2.215748000
H	5.811105000	−0.259241000	−1.708328000
H	7.120317000	0.222883000	−2.795750000
H	5.537974000	0.995054000	−2.931825000
C	2.928998000	−2.332634000	0.133826000
C	3.146344000	−2.613749000	−1.222415000
H	2.387161000	−2.355719000	−1.956440000
C	4.331430000	−3.218253000	−1.635831000
H	4.490074000	−3.432732000	−2.689308000
C	5.317030000	−3.534196000	−0.699840000
H	6.244727000	−3.998689000	−1.022630000
C	5.111407000	−3.247581000	0.648930000
H	5.877117000	−3.490305000	1.380811000
C	3.922443000	−2.651229000	1.068155000
H	3.770680000	−2.435149000	2.121551000
C	1.585744000	−0.988921000	2.363129000
C	2.080901000	0.304688000	2.576098000
H	2.296012000	0.960482000	1.735569000
C	2.309713000	0.761965000	3.873012000
H	2.697848000	1.764953000	4.028726000
C	2.040564000	−0.064535000	4.963584000
H	2.216744000	0.293565000	5.974280000
C	1.546209000	−1.352141000	4.755906000

H	1.338958000	−1.999702000	5.603728000
C	1.318398000	−1.815656000	3.461050000
H	0.935248000	−2.820269000	3.304705000
C	0.147844000	−2.967834000	0.756057000
C	−1.209395000	−2.611678000	0.973916000
C	−2.144535000	−3.657760000	1.122530000
H	−3.184297000	−3.407917000	1.317995000
C	−1.752913000	−4.988340000	1.032477000
H	−2.496365000	−5.772358000	1.155317000
C	−0.419096000	−5.325970000	0.782330000
H	−0.117488000	−6.367131000	0.713193000
C	0.525134000	−4.313080000	0.646607000
H	1.568744000	−4.563725000	0.476973000
C	−2.413631000	−0.749656000	1.935351000
C	−2.603344000	−1.385413000	3.182335000
H	−2.089567000	−2.319801000	3.383374000
C	−3.409251000	−0.828148000	4.165942000
H	−3.519822000	−1.349440000	5.113774000
C	−4.057290000	0.392836000	3.963796000
H	−4.679953000	0.829117000	4.738787000
C	−3.874795000	1.046909000	2.751550000
H	−4.351197000	2.009480000	2.580924000
C	−3.075836000	0.491508000	1.742718000
C	−2.720207000	3.129667000	0.571224000
C	−3.420423000	4.114412000	−0.133691000
H	−4.111381000	3.838311000	−0.924668000
C	−3.255153000	5.461258000	0.191346000
H	−3.811653000	6.216302000	−0.357393000
C	−2.393543000	5.836518000	1.219809000
H	−2.271683000	6.885692000	1.474268000
C	−1.697194000	4.857879000	1.930354000
H	−1.036895000	5.140972000	2.745946000
C	−1.854573000	3.511902000	1.607606000
H	−1.324747000	2.757495000	2.184823000
C	−4.417229000	1.146163000	−0.794361000

C	−5.569943000	0.615817000	−0.201478000
H	−5.558126000	0.324581000	0.844470000
C	−6.735761000	0.459340000	−0.950994000
H	−7.625362000	0.050987000	−0.478772000
C	−6.765949000	0.830279000	−2.295084000
H	−7.678256000	0.711635000	−2.873239000
C	−5.621395000	1.356229000	−2.894683000
H	−5.637625000	1.650430000	−3.940892000
C	−4.450836000	1.504894000	−2.151698000
H	−3.560788000	1.906249000	−2.628970000
C	1.022982000	2.255328000	−0.580103000
C	1.757118000	3.354798000	0.141469000
H	1.130392000	3.993343000	0.762959000
C	3.058941000	3.653965000	0.037026000
C	4.115610000	2.981072000	−0.790153000
H	4.444890000	3.702594000	−1.553807000
H	3.701387000	2.129491000	−1.343963000
Y	−0.714133000	−0.288155000	−0.974768000
H	3.414883000	4.512504000	0.609804000
O	−1.901666000	−1.886542000	−2.227267000
C	−1.295141000	−2.888486000	−3.101963000
C	−3.323093000	−2.182847000	−2.045362000
C	−2.350695000	−3.972381000	−3.260138000
H	−1.057532000	−2.396358000	−4.050472000
H	−0.374538000	−3.238139000	−2.629769000
C	−3.654807000	−3.180665000	−3.141304000
H	−3.443792000	−2.610249000	−1.045815000
H	−3.867848000	−1.239985000	−2.116297000
H	−2.269851000	−4.704233000	−2.449402000
H	−2.250952000	−4.499755000	−4.212855000
H	−4.512986000	−3.804644000	−2.877021000
H	−3.883673000	−2.663373000	−4.080044000
H	1.709882000	1.423624000	−0.783118000
H	0.240610000	1.892279000	0.118924000
C	0.358830000	2.751592000	−1.882583000

C	−0.543172000	1.796419000	−2.654325000
H	1.168512000	3.098668000	−2.541937000
H	−0.229599000	3.640181000	−1.629273000
H	−1.435496000	2.270176000	−3.057778000
C	−0.121872000	0.652742000	−3.334770000
C	0.929311000	−0.214264000	−2.987109000
H	−0.837109000	0.263064000	−4.065552000
H	1.134092000	−1.058224000	−3.641920000
H	1.814466000	0.176734000	−2.484084000

117

theta1_thf_C2_trans_1,4 E = −2200.65354 a.u.

N	0.769215000	−1.277829000	1.218021000
P	2.925110000	0.604647000	0.254181000
P	−1.971454000	−1.828902000	0.074504000
Si	−4.884372000	4.592286000	−0.198387000
C	−4.059457000	3.151669000	0.735721000
H	−4.805888000	2.356931000	0.883023000
H	−3.801466000	3.504799000	1.744718000
C	−5.464944000	4.017814000	−1.905138000
H	−4.630862000	3.693012000	−2.539562000
H	−5.975781000	4.831114000	−2.434826000
H	−6.173603000	3.183771000	−1.830282000
C	−6.365668000	5.179019000	0.811776000
H	−7.100263000	4.377211000	0.953897000
H	−6.878875000	6.011191000	0.314887000
H	−6.059853000	5.527394000	1.805573000
C	−3.647566000	6.007428000	−0.404331000
H	−3.246790000	6.333399000	0.563229000
H	−4.124348000	6.877289000	−0.872044000
H	−2.798642000	5.724538000	−1.039264000
C	4.248097000	0.876353000	−1.016703000
C	3.981576000	0.540472000	−2.351779000
H	3.009688000	0.133715000	−2.618667000
C	4.950160000	0.718125000	−3.338348000
H	4.731003000	0.450491000	−4.369110000

C	6.198574000	1.243995000	−3.004331000
H	6.953518000	1.387594000	−3.772432000
C	6.472017000	1.584305000	−1.680639000
H	7.443045000	1.991987000	−1.412400000
C	5.505260000	1.402336000	−0.691302000
H	5.731809000	1.674904000	0.335091000
C	3.513609000	1.627636000	1.679649000
C	3.523268000	3.024311000	1.542529000
H	3.203997000	3.481445000	0.608601000
C	3.940241000	3.832009000	2.597033000
H	3.949842000	4.912309000	2.478092000
C	4.337750000	3.256976000	3.805590000
H	4.657825000	3.888400000	4.629899000
C	4.322552000	1.871730000	3.950093000
H	4.634481000	1.417550000	4.886901000
C	3.915783000	1.057223000	2.891624000
H	3.921653000	−0.022163000	3.009947000
C	3.183223000	−1.130206000	0.811829000
C	2.052744000	−1.834652000	1.299543000
C	2.271234000	−3.133957000	1.804216000
H	1.428599000	−3.693734000	2.197103000
C	3.533687000	−3.712980000	1.795765000
H	3.657198000	−4.720634000	2.185076000
C	4.633960000	−3.022955000	1.282389000
H	5.619582000	−3.478565000	1.269677000
C	4.447160000	−1.733428000	0.796949000
H	5.296054000	−1.177224000	0.407729000
C	−0.172115000	−1.609015000	2.212832000
C	0.176305000	−1.619295000	3.579119000
H	1.210959000	−1.434247000	3.853183000
C	−0.776004000	−1.836098000	4.565142000
H	−0.474597000	−1.830797000	5.609553000
C	−2.116382000	−2.034614000	4.223650000
H	−2.866162000	−2.189357000	4.993954000
C	−2.483018000	−2.034522000	2.882854000

H	−3.523970000	−2.186897000	2.614818000
C	−1.529502000	−1.850924000	1.871661000
C	−3.817619000	−1.812704000	−0.018642000
C	−4.580549000	−2.920904000	0.381676000
H	−4.090071000	−3.806154000	0.777530000
C	−5.967529000	−2.900848000	0.258548000
H	−6.548539000	−3.762789000	0.575354000
C	−6.608143000	−1.783709000	−0.279479000
H	−7.689965000	−1.773706000	−0.380859000
C	−5.857132000	−0.687137000	−0.697391000
H	−6.349594000	0.179428000	−1.130140000
C	−4.468269000	−0.702495000	−0.568086000
H	−3.886515000	0.151962000	−0.899583000
C	−1.630006000	−3.558817000	−0.508885000
C	−1.012352000	−4.523995000	0.292259000
H	−0.719544000	−4.276132000	1.307751000
C	−0.796131000	−5.813292000	−0.197859000
H	−0.320384000	−6.556153000	0.437024000
C	−1.203548000	−6.153425000	−1.486053000
H	−1.044191000	−7.160635000	−1.861185000
C	−1.831603000	−5.198493000	−2.288104000
H	−2.169911000	−5.462100000	−3.287019000
C	−2.036915000	−3.907737000	−1.805985000
H	−2.547187000	−3.177756000	−2.431671000
C	0.118050000	2.410468000	1.345567000
C	−1.030746000	1.717147000	1.749673000
H	−0.914190000	1.087182000	2.635701000
C	−2.180370000	1.486706000	0.984901000
C	−2.796873000	2.549122000	0.087375000
H	−3.065872000	2.161481000	−0.906967000
H	−2.068395000	3.351984000	−0.081419000
Y	0.004793000	0.341145000	−0.164689000
H	−2.907544000	0.809070000	1.430836000
O	0.716342000	−0.917754000	−2.238179000
C	0.242403000	−0.845066000	−3.607341000

C	1.411184000	−2.198189000	−2.039013000
C	1.069243000	−1.860307000	−4.377978000
H	−0.826501000	−1.094915000	−3.624082000
H	0.380125000	0.178720000	−3.962064000
C	1.254367000	−2.969601000	−3.342476000
H	2.455618000	−1.967746000	−1.813886000
H	0.957724000	−2.695439000	−1.181001000
H	2.034136000	−1.427478000	−4.666120000
H	0.562743000	−2.196411000	−5.287173000
H	2.124404000	−3.600781000	−3.543620000
H	0.369010000	−3.610927000	−3.302081000
H	0.050157000	3.182737000	0.574231000
H	0.933055000	2.540082000	2.048751000
C	−0.471149000	2.402076000	−2.455201000
C	0.843800000	2.700090000	−2.535690000
H	−1.178988000	3.108179000	−2.032136000
H	−0.898095000	1.524969000	−2.936703000
H	1.531992000	2.002915000	−3.014549000
C	1.426719000	3.946863000	−2.065989000
C	2.718871000	4.262479000	−2.242855000
H	0.752744000	4.657996000	−1.589426000
H	3.116405000	5.220424000	−1.920441000
H	3.413959000	3.578652000	−2.724298000

117

theta1_thf_TS2_trans_1,4 E = −2200.61775 a.u. Imaginary frequency=266i

N	−1.177435000	−0.837123000	1.230818000
P	1.393675000	−2.118401000	0.312209000
P	−2.829475000	1.433623000	0.089396000
Si	6.567825000	2.948226000	−0.754012000
C	5.017927000	3.468726000	0.223177000
H	5.159840000	4.512182000	0.542230000
H	4.963767000	2.884160000	1.152991000
C	6.753516000	4.035174000	−2.287096000
H	5.908580000	3.924472000	−2.977514000
H	7.662634000	3.772146000	−2.841382000

H	6.828456000	5.096426000	−2.020752000
C	8.071722000	3.150056000	0.367234000
H	8.187591000	4.188770000	0.699153000
H	8.994159000	2.867572000	−0.154376000
H	7.995882000	2.523133000	1.263980000
C	6.416224000	1.138089000	−1.288292000
H	6.319278000	0.465056000	−0.427195000
H	7.306968000	0.823611000	−1.845683000
H	5.551820000	0.975030000	−1.944393000
C	2.328311000	−3.432556000	−0.587050000
C	1.790463000	−3.945520000	−1.776298000
H	0.842665000	−3.566061000	−2.151525000
C	2.457161000	−4.943963000	−2.481057000
H	2.027312000	−5.338054000	−3.398277000
C	3.677707000	−5.433225000	−2.012588000
H	4.201760000	−6.208108000	−2.565163000
C	4.220495000	−4.925058000	−0.834662000
H	5.168692000	−5.303907000	−0.462946000
C	3.549645000	−3.931462000	−0.120437000
H	3.978897000	−3.550143000	0.800955000
C	2.489122000	−1.645890000	1.722611000
C	3.548097000	−0.758757000	1.484808000
H	3.718842000	−0.363395000	0.485679000
C	4.401736000	−0.390574000	2.522349000
H	5.223811000	0.292732000	2.328693000
C	4.199089000	−0.895502000	3.806947000
H	4.861954000	−0.603163000	4.616718000
C	3.146646000	−1.776829000	4.048841000
H	2.988909000	−2.176945000	5.046841000
C	2.294495000	−2.154737000	3.011628000
H	1.482210000	−2.849560000	3.204134000
C	−0.019565000	−3.000132000	1.091734000
C	−1.114087000	−2.207516000	1.526790000
C	−2.159161000	−2.875932000	2.202296000
H	−3.003599000	−2.295788000	2.562138000

C	−2.133019000	−4.249434000	2.405844000
H	−2.962576000	−4.725116000	2.923414000
C	−1.063539000	−5.021513000	1.943835000
H	−1.045146000	−6.095830000	2.100920000
C	−0.011953000	−4.387477000	1.291929000
H	0.837846000	−4.969329000	0.944729000
C	−1.715200000	0.003112000	2.220832000
C	−1.440112000	−0.200566000	3.589483000
H	−0.816772000	−1.042010000	3.876512000
C	−1.932269000	0.657414000	4.563550000
H	−1.690205000	0.471340000	5.606989000
C	−2.716008000	1.759279000	4.213502000
H	−3.099669000	2.432166000	4.974581000
C	−2.991789000	1.988433000	2.870800000
H	−3.593209000	2.846393000	2.583541000
C	−2.507070000	1.127395000	1.876923000
C	−3.290670000	3.227492000	0.026075000
C	−4.620612000	3.668778000	0.018501000
H	−5.436688000	2.953693000	0.040489000
C	−4.910124000	5.033065000	−0.011252000
H	−5.946680000	5.359497000	−0.017626000
C	−3.880859000	5.972224000	−0.025886000
H	−4.110482000	7.033884000	−0.050865000
C	−2.554578000	5.542338000	−0.001618000
H	−1.744377000	6.266621000	−0.003294000
C	−2.262649000	4.180506000	0.021993000
H	−1.226656000	3.860048000	0.058510000
C	−4.433779000	0.576265000	−0.266415000
C	−5.003093000	−0.326297000	0.637573000
H	−4.526159000	−0.499169000	1.597692000
C	−6.196841000	−0.979692000	0.326221000
H	−6.633159000	−1.671381000	1.042147000
C	−6.840125000	−0.730203000	−0.885196000
H	−7.777201000	−1.228114000	−1.119129000
C	−6.284277000	0.177116000	−1.789455000

H	−6.790181000	0.393064000	−2.727035000
C	−5.085522000	0.820047000	−1.486069000
H	−4.676873000	1.546121000	−2.186598000
C	0.928179000	1.872312000	0.157757000
C	1.372895000	3.204045000	0.595680000
H	0.671602000	3.732717000	1.240683000
C	2.516540000	3.845530000	0.284218000
C	3.677866000	3.349522000	−0.528893000
H	3.739442000	3.939238000	−1.456781000
H	3.519074000	2.308185000	−0.841112000
Y	−0.337762000	−0.049470000	−0.793375000
H	2.648550000	4.846007000	0.698105000
O	−1.945436000	−1.320056000	−2.098536000
C	−2.635013000	−0.986514000	−3.338683000
C	−2.463008000	−2.592798000	−1.584853000
C	−3.298188000	−2.276606000	−3.789026000
H	−3.372827000	−0.211330000	−3.114611000
H	−1.899396000	−0.598634000	−4.045928000
C	−3.664861000	−2.925906000	−2.454197000
H	−1.663614000	−3.335485000	−1.679641000
H	−2.705838000	−2.454842000	−0.531199000
H	−2.592494000	−2.901935000	−4.348485000
H	−4.165055000	−2.086153000	−4.427810000
H	−3.823731000	−4.005309000	−2.529024000
H	−4.571089000	−2.470390000	−2.041744000
H	1.780039000	1.194912000	−0.041204000
H	0.319164000	1.442496000	0.976619000
C	0.396164000	2.384460000	−1.905432000
C	0.090990000	1.350045000	−2.814931000
H	1.414890000	2.758464000	−1.874679000
H	−0.349497000	3.147963000	−1.710159000
H	−0.906766000	1.263752000	−3.242414000
C	1.118388000	0.377857000	−3.089758000
C	0.989778000	−0.919833000	−3.481493000
H	2.127877000	0.696972000	−2.812735000

H	1.866376000	−1.549132000	−3.593538000
H	0.045639000	−1.342116000	−3.812299000

117

theta1_thf_P2_trans_1,2 E = −2200.676618 a.u.

N	2.431630000	0.704774000	0.757573000
P	0.146632000	2.482899000	0.424373000
P	1.656243000	−2.163104000	0.396371000
Si	−8.210875000	−0.355080000	−0.615102000
C	−6.898116000	−1.600050000	−0.026297000
H	−7.417471000	−2.461965000	0.418968000
H	−6.331220000	−1.142491000	0.798134000
C	−9.292124000	−1.134377000	−1.953731000
H	−8.710671000	−1.418756000	−2.839069000
H	−10.069219000	−0.434419000	−2.284218000
H	−9.798163000	−2.035253000	−1.585651000
C	−9.278249000	0.125612000	0.867111000
H	−9.782382000	−0.748417000	1.296963000
H	−10.055753000	0.844120000	0.580318000
H	−8.679999000	0.587169000	1.662217000
C	−7.358393000	1.185545000	−1.307747000
H	−6.710698000	1.658071000	−0.558550000
H	−8.096360000	1.933662000	−1.622016000
H	−6.741168000	0.950029000	−2.183422000
C	−0.837645000	3.941326000	−0.130399000
C	−0.606868000	4.482677000	−1.404703000
H	0.182744000	4.078560000	−2.032977000
C	−1.374185000	5.547935000	−1.868817000
H	−1.179890000	5.963185000	−2.854206000
C	−2.390903000	6.077128000	−1.072925000
H	−2.993090000	6.904603000	−1.437642000
C	−2.628937000	5.541565000	0.190810000
H	−3.416179000	5.951466000	0.817689000
C	−1.856355000	4.480798000	0.664056000
H	−2.048820000	4.079087000	1.654179000
C	−0.333176000	2.218422000	2.182011000

C	−1.388547000	1.336082000	2.451578000
H	−1.896110000	0.824972000	1.635442000
C	−1.805920000	1.121997000	3.764530000
H	−2.631239000	0.444223000	3.965862000
C	−1.164885000	1.778438000	4.815016000
H	−1.487102000	1.609146000	5.838804000
C	−0.112134000	2.654649000	4.551125000
H	0.385490000	3.169559000	5.368501000
C	0.303185000	2.878111000	3.239494000
H	1.119400000	3.566624000	3.039241000
C	1.903244000	3.058393000	0.455411000
C	2.868274000	2.036662000	0.661524000
C	4.228156000	2.408911000	0.685465000
H	4.978632000	1.640860000	0.854200000
C	4.610218000	3.734773000	0.517734000
H	5.665235000	3.995761000	0.551197000
C	3.652215000	4.731627000	0.307180000
H	3.954551000	5.767482000	0.183029000
C	2.304516000	4.389030000	0.272999000
H	1.553677000	5.159566000	0.122482000
C	2.831344000	−0.082423000	1.834279000
C	3.471589000	0.438458000	2.980661000
H	3.675136000	1.502727000	3.038175000
C	3.822808000	−0.377434000	4.047936000
H	4.309181000	0.068206000	4.912378000
C	3.545420000	−1.746273000	4.034922000
H	3.817341000	−2.378958000	4.874180000
C	2.894145000	−2.279234000	2.929222000
H	2.639963000	−3.336311000	2.909867000
C	2.545492000	−1.474060000	1.836941000
C	0.515516000	−3.419703000	1.122897000
C	0.545395000	−4.770343000	0.760975000
H	1.271882000	−5.130085000	0.038358000
C	−0.349259000	−5.669661000	1.343039000
H	−0.314404000	−6.718398000	1.060655000

C	−1.273070000	−5.229143000	2.288153000
H	−1.964360000	−5.932582000	2.743975000
C	−1.304462000	−3.881830000	2.652860000
H	−2.019770000	−3.534175000	3.393196000
C	−0.419119000	−2.978590000	2.071561000
H	−0.439073000	−1.934592000	2.376434000
C	2.897985000	−3.130861000	−0.576446000
C	4.200865000	−3.359460000	−0.117047000
H	4.500978000	−2.996682000	0.861749000
C	5.112169000	−4.053678000	−0.913289000
H	6.119415000	−4.231384000	−0.545706000
C	4.733662000	−4.527369000	−2.169779000
H	5.445448000	−5.072678000	−2.783524000
C	3.436343000	−4.305692000	−2.634335000
H	3.132526000	−4.679763000	−3.608487000
C	2.525378000	−3.603736000	−1.845808000
H	1.514364000	−3.439390000	−2.213269000
C	−2.733427000	−1.801931000	−0.927333000
C	−3.583274000	−2.945700000	−0.452244000
H	−3.027817000	−3.761303000	0.013212000
C	−4.913415000	−3.071441000	−0.544237000
C	−5.911460000	−2.097754000	−1.102334000
H	−6.476875000	−2.593846000	−1.904674000
H	−5.406300000	−1.244133000	−1.569048000
Y	1.158702000	0.259549000	−1.099141000
H	−5.354880000	−3.983907000	−0.138224000
O	3.036874000	0.495044000	−2.516713000
C	3.228450000	1.585200000	−3.471837000
C	4.317969000	−0.168924000	−2.260893000
C	4.730783000	1.803158000	−3.522119000
H	2.824784000	1.258747000	−4.436124000
H	2.661315000	2.447452000	−3.114719000
C	5.267489000	0.385952000	−3.311086000
H	4.622542000	0.092136000	−1.242961000
H	4.153334000	−1.245653000	−2.331454000

H	5.048036000	2.462954000	−2.707372000
H	5.046391000	2.246661000	−4.470487000
H	6.306401000	0.365574000	−2.970843000
H	5.200055000	−0.194779000	−4.237874000
H	−3.355220000	−1.035350000	−1.401674000
H	−2.248247000	−1.322291000	−0.059959000
C	−1.636337000	−2.265997000	−1.895067000
C	−0.689704000	−1.187452000	−2.374523000
H	−2.121293000	−2.731422000	−2.767456000
H	−1.066526000	−3.071906000	−1.413731000
H	0.128470000	−1.555692000	−3.012939000
C	−1.030110000	0.150038000	−2.540203000
C	−0.165803000	1.146493000	−3.047675000
H	−1.937788000	0.499125000	−2.043524000
H	−0.556239000	2.153232000	−3.163328000
H	0.539968000	0.844476000	−3.828529000

104

theta3_C2_cis_1,4 E = −1968.317881 a.u.

N	−0.736906000	−1.852656000	0.584022000
P	−2.693180000	0.409884000	0.176275000
P	2.096489000	−1.213158000	0.185526000
Si	2.954158000	4.654095000	−0.570898000
C	1.412983000	3.918976000	−1.417025000
H	1.612484000	3.855606000	−2.496267000
H	0.593254000	4.644665000	−1.310991000
C	4.441608000	3.518120000	−0.835124000
H	4.298698000	2.519190000	−0.405256000
H	5.341078000	3.943163000	−0.373241000
H	4.654616000	3.389689000	−1.903621000
C	3.296674000	6.332615000	−1.359449000
H	3.498951000	6.238626000	−2.433136000
H	4.169832000	6.812937000	−0.901503000
H	2.446343000	7.014787000	−1.240842000
C	2.626958000	4.876192000	1.277747000
H	1.785509000	5.558134000	1.450600000

H	3.502986000	5.303317000	1.780822000
H	2.391017000	3.927811000	1.775427000
C	−2.829833000	2.085287000	0.947642000
C	−1.796015000	2.477454000	1.812182000
H	−0.979670000	1.793602000	2.036002000
C	−1.824856000	3.728849000	2.420777000
H	−1.024448000	4.017020000	3.096909000
C	−2.881281000	4.606094000	2.169647000
H	−2.903714000	5.582658000	2.645348000
C	−3.912245000	4.219789000	1.316916000
H	−4.745432000	4.891444000	1.128214000
C	−3.891106000	2.963676000	0.707780000
H	−4.713357000	2.671079000	0.062109000
C	−4.293968000	0.127864000	−0.700524000
C	−4.594823000	0.902757000	−1.833126000
H	−3.916352000	1.691393000	−2.152703000
C	−5.767464000	0.676653000	−2.551280000
H	−5.997177000	1.291989000	−3.417213000
C	−6.642619000	−0.338318000	−2.159515000
H	−7.555125000	−0.516690000	−2.721544000
C	−6.343099000	−1.118691000	−1.044392000
H	−7.022457000	−1.907686000	−0.733088000
C	−5.176239000	−0.887446000	−0.314444000
H	−4.956295000	−1.493501000	0.559438000
C	−2.652446000	−0.718438000	1.609178000
C	−1.647436000	−1.719940000	1.639722000
C	−1.587001000	−2.513052000	2.808965000
H	−0.816430000	−3.271022000	2.897538000
C	−2.478311000	−2.336216000	3.858311000
H	−2.387768000	−2.970364000	4.736924000
C	−3.468110000	−1.352032000	3.809006000
H	−4.160744000	−1.213712000	4.633397000
C	−3.532865000	−0.539654000	2.685105000
H	−4.273809000	0.254821000	2.634771000
C	0.037738000	−3.028895000	0.533335000

C	−0.526602000	−4.318695000	0.588323000
H	−1.595895000	−4.417467000	0.755918000
C	0.259445000	−5.453513000	0.416728000
H	−0.205437000	−6.435383000	0.456648000
C	1.632264000	−5.340564000	0.180574000
H	2.242827000	−6.228036000	0.042781000
C	2.212891000	−4.076469000	0.128777000
H	3.279961000	−3.978129000	−0.050859000
C	1.434349000	−2.927841000	0.316176000
C	3.814066000	−1.399403000	−0.455398000
C	4.890752000	−1.693048000	0.392898000
H	4.728441000	−1.800709000	1.461823000
C	6.173594000	−1.840568000	−0.131360000
H	7.003089000	−2.067077000	0.532996000
C	6.394102000	−1.695201000	−1.501151000
H	7.396465000	−1.807086000	−1.905175000
C	5.328953000	−1.396853000	−2.350119000
H	5.498804000	−1.272110000	−3.416248000
C	4.045383000	−1.244309000	−1.828773000
H	3.221242000	−0.996474000	−2.493658000
C	2.315299000	−0.642788000	1.927460000
C	2.117832000	−1.493496000	3.020193000
H	1.825795000	−2.526706000	2.858553000
C	2.303308000	−1.018765000	4.318601000
H	2.149742000	−1.686825000	5.161760000
C	2.689334000	0.302823000	4.534675000
H	2.836430000	0.668708000	5.547131000
C	2.892833000	1.154108000	3.447934000
H	3.204277000	2.182368000	3.610563000
C	2.701924000	0.686455000	2.149816000
H	2.871572000	1.354829000	1.309378000
C	0.539114000	−2.421252000	−3.565815000
C	−0.364503000	−2.884380000	−2.666566000
H	−0.039209000	−3.610876000	−1.927476000
C	−1.769086000	−2.480715000	−2.602555000

C	−2.322131000	−1.453575000	−3.290925000
H	−3.366174000	−1.191646000	−3.156989000
H	−1.771587000	−0.902003000	−4.050490000
Y	−0.340407000	−0.463329000	−1.249037000
H	−2.387993000	−3.022255000	−1.889434000
H	1.572943000	−2.751815000	−3.524829000
H	0.256939000	−1.819342000	−4.420834000
C	0.912687000	2.540037000	−0.926242000
C	−0.322116000	2.115164000	−1.700510000
H	1.729624000	1.808886000	−1.028868000
H	0.677092000	2.598381000	0.144213000
H	−1.212020000	2.700328000	−1.476489000
C	−0.264558000	1.517818000	−2.961679000
C	0.747247000	0.662885000	−3.429776000
H	−1.183582000	1.548242000	−3.553472000
H	0.691627000	0.309056000	−4.453839000
H	1.763620000	0.767405000	−3.047678000

104

theta3_TS2_cis_1,4 E = −1968.309717 a.u. Imaginary frequency=185i

N	−0.671646000	−1.869734000	0.595255000
P	−2.743754000	0.293713000	0.131932000
P	2.153305000	−1.199942000	0.155048000
Si	2.744431000	4.793215000	−0.459962000
C	1.231447000	4.001400000	−1.312588000
H	1.389872000	4.054386000	−2.399072000
H	0.357922000	4.637345000	−1.108004000
C	4.285460000	3.756131000	−0.807081000
H	4.210653000	2.735362000	−0.412465000
H	5.171629000	4.214167000	−0.351197000
H	4.477114000	3.680525000	−1.884601000
C	2.960919000	6.521039000	−1.180646000
H	3.147272000	6.488915000	−2.260735000
H	3.810060000	7.036615000	−0.716011000
H	2.070791000	7.139875000	−1.015550000
C	2.438602000	4.912296000	1.400371000

H	1.581035000	5.560513000	1.617646000
H	3.309477000	5.339715000	1.912154000
H	2.236045000	3.934561000	1.853700000
C	−2.840919000	1.985392000	0.885563000
C	−1.823197000	2.349662000	1.781400000
H	−1.050620000	1.632656000	2.052203000
C	−1.817229000	3.613574000	2.365053000
H	−1.031888000	3.877624000	3.068226000
C	−2.822479000	4.532217000	2.058120000
H	−2.820129000	5.517162000	2.516866000
C	−3.836690000	4.175186000	1.172967000
H	−4.631898000	4.878806000	0.941412000
C	−3.849431000	2.907497000	0.587719000
H	−4.660670000	2.638305000	−0.081644000
C	−4.390616000	0.017815000	−0.654998000
C	−4.702107000	0.735667000	−1.820846000
H	−3.999453000	1.466637000	−2.216770000
C	−5.910943000	0.523885000	−2.479787000
H	−6.145594000	1.093878000	−3.374874000
C	−6.814183000	−0.422688000	−1.993832000
H	−7.754799000	−0.592703000	−2.510225000
C	−6.503817000	−1.150402000	−0.846652000
H	−7.202222000	−1.890427000	−0.465187000
C	−5.299201000	−0.932351000	−0.176304000
H	−5.068303000	−1.504334000	0.717149000
C	−2.632538000	−0.794992000	1.594535000
C	−1.590832000	−1.757631000	1.642407000
C	−1.501459000	−2.533448000	2.821453000
H	−0.708249000	−3.267669000	2.913319000
C	−2.391484000	−2.367675000	3.872871000
H	−2.278775000	−2.985969000	4.760177000
C	−3.407707000	−1.410601000	3.815307000
H	−4.097000000	−1.277049000	4.643315000
C	−3.506907000	−0.621763000	2.677500000
H	−4.272985000	0.148140000	2.620604000

C	0.114944000	−3.037413000	0.522041000
C	−0.436080000	−4.332864000	0.569678000
H	−1.504467000	−4.442431000	0.737120000
C	0.363608000	−5.458879000	0.406756000
H	−0.090573000	−6.445765000	0.445466000
C	1.736898000	−5.331137000	0.183389000
H	2.358943000	−6.212068000	0.055178000
C	2.303688000	−4.060975000	0.129724000
H	3.370726000	−3.951607000	−0.043275000
C	1.510779000	−2.920641000	0.307988000
C	3.871154000	−1.385496000	−0.488906000
C	4.965690000	−1.591209000	0.361937000
H	4.819611000	−1.630454000	1.437716000
C	6.245840000	−1.741728000	−0.168812000
H	7.088783000	−1.899697000	0.498442000
C	6.446218000	−1.689433000	−1.548034000
H	7.446040000	−1.804781000	−1.957279000
C	5.362565000	−1.482572000	−2.401094000
H	5.515077000	−1.435325000	−3.476056000
C	4.082924000	−1.325748000	−1.872676000
H	3.243097000	−1.155452000	−2.542322000
C	2.378895000	−0.604426000	1.887822000
C	2.177067000	−1.438782000	2.992183000
H	1.878436000	−2.472091000	2.844251000
C	2.367413000	−0.948401000	4.284084000
H	2.209627000	−1.604221000	5.136017000
C	2.764672000	0.372567000	4.482428000
H	2.916789000	0.750433000	5.489730000
C	2.973537000	1.207646000	3.384143000
H	3.294696000	2.234978000	3.532903000
C	2.775658000	0.724620000	2.092661000
H	2.949410000	1.380601000	1.243163000
C	0.442103000	−1.593839000	−3.832318000
C	−0.007210000	−2.501281000	−2.865935000
H	0.704495000	−3.172036000	−2.390600000

C	−1.404120000	−2.644848000	−2.541123000
C	−2.370281000	−1.726100000	−2.838331000
H	−3.394809000	−1.866924000	−2.515159000
H	−2.200892000	−0.910335000	−3.543250000
Y	−0.346680000	−0.559744000	−1.270636000
H	−1.666218000	−3.481168000	−1.896233000
H	1.480337000	−1.639850000	−4.144639000
H	−0.252537000	−1.273617000	−4.603049000
C	0.884636000	2.541571000	−0.934263000
C	−0.325961000	2.102519000	−1.728659000
H	1.754599000	1.899240000	−1.125650000
H	0.673976000	2.485773000	0.141270000
H	−1.244641000	2.626383000	−1.471242000
C	−0.285596000	1.484758000	−2.965112000
C	0.768579000	0.674282000	−3.487917000
H	−1.202577000	1.513929000	−3.559179000
H	0.820467000	0.602399000	−4.568466000
H	1.755215000	0.759200000	−3.027606000

104

theta3_P2_cis_1,4 E = −1968.346385 a.u.

N	−1.189453000	−1.761814000	0.495729000
P	−2.749252000	0.766526000	0.179547000
P	1.749220000	−1.647151000	0.195421000
Si	4.196110000	4.427317000	0.118209000
C	2.438308000	4.048803000	−0.530305000
H	2.324080000	4.521787000	−1.515890000
H	1.715936000	4.552012000	0.129097000
C	5.476338000	3.659951000	−1.037259000
H	5.408456000	2.565380000	−1.062437000
H	6.491964000	3.912075000	−0.708990000
H	5.365518000	4.028184000	−2.064423000
C	4.393588000	6.300609000	0.165904000
H	4.272835000	6.743835000	−0.829752000
H	5.388883000	6.581177000	0.530944000
H	3.656431000	6.768350000	0.829484000

C	4.387791000	3.704736000	1.852933000
H	3.613616000	4.077694000	2.534651000
H	5.359359000	3.979234000	2.281310000
H	4.338347000	2.608945000	1.848692000
C	−2.740777000	2.397347000	1.050607000
C	−1.725355000	2.620248000	1.994499000
H	−1.011334000	1.831618000	2.226449000
C	−1.649751000	3.834073000	2.672404000
H	−0.869320000	3.990588000	3.412518000
C	−2.579541000	4.841794000	2.410059000
H	−2.520820000	5.788582000	2.939731000
C	−3.588559000	4.625282000	1.474440000
H	−4.322493000	5.401265000	1.274253000
C	−3.673150000	3.408157000	0.796154000
H	−4.474305000	3.247909000	0.080721000
C	−4.282875000	0.743057000	−0.845134000
C	−4.325556000	1.548550000	−1.994098000
H	−3.488014000	2.200410000	−2.238158000
C	−5.445466000	1.537824000	−2.822000000
H	−5.473242000	2.174081000	−3.702554000
C	−6.525603000	0.706483000	−2.522288000
H	−7.396886000	0.692340000	−3.171155000
C	−6.483046000	−0.105971000	−1.390816000
H	−7.321779000	−0.755126000	−1.154327000
C	−5.368671000	−0.089043000	−0.551711000
H	−5.345450000	−0.723323000	0.329249000
C	−2.967374000	−0.460877000	1.520997000
C	−2.160941000	−1.626255000	1.483875000
C	−2.344992000	−2.576984000	2.507994000
H	−1.732432000	−3.473308000	2.514937000
C	−3.276206000	−2.371253000	3.517871000
H	−3.386472000	−3.122833000	4.295676000
C	−4.051979000	−1.209921000	3.558561000
H	−4.770325000	−1.049996000	4.356765000
C	−3.884168000	−0.257439000	2.559948000

H	−4.471377000	0.657639000	2.579649000
C	−0.692769000	−3.012430000	0.109402000
C	−1.518519000	−4.130394000	−0.142549000
H	−2.582051000	−4.051709000	0.064583000
C	−0.989409000	−5.300758000	−0.663125000
H	−1.645715000	−6.146618000	−0.851029000
C	0.375483000	−5.396106000	−0.967077000
H	0.782084000	−6.314206000	−1.381071000
C	1.209574000	−4.313808000	−0.722371000
H	2.271474000	−4.388488000	−0.938400000
C	0.699256000	−3.129425000	−0.164343000
C	3.448582000	−2.102999000	−0.350345000
C	4.271942000	−2.935761000	0.420546000
H	3.917534000	−3.325531000	1.370837000
C	5.552169000	−3.257736000	−0.024394000
H	6.184823000	−3.903507000	0.578454000
C	6.022511000	−2.751071000	−1.236567000
H	7.022462000	−3.002776000	−1.579093000
C	5.212682000	−1.914548000	−2.003308000
H	5.579431000	−1.511290000	−2.943485000
C	3.932546000	−1.586724000	−1.558998000
H	3.305904000	−0.925386000	−2.151820000
C	1.851406000	−1.576481000	2.035389000
C	1.542751000	−2.674918000	2.845779000
H	1.207886000	−3.605218000	2.395366000
C	1.666281000	−2.577484000	4.231337000
H	1.427322000	−3.435309000	4.854261000
C	2.097719000	−1.387684000	4.816358000
H	2.193710000	−1.315907000	5.896231000
C	2.408058000	−0.290215000	4.013274000
H	2.749334000	0.637729000	4.464205000
C	2.282398000	−0.382951000	2.628807000
H	2.533379000	0.474406000	2.008347000
C	0.542738000	0.395419000	−4.312745000
C	0.635569000	−0.955281000	−3.621337000

H	1.600403000	−1.453374000	−3.699086000
C	−0.464282000	−1.777989000	−3.387828000
C	−1.780228000	−1.354796000	−3.093429000
H	−2.550383000	−2.105613000	−2.937714000
H	−2.153177000	−0.413161000	−3.509557000
Y	−0.477201000	−0.465364000	−1.178237000
H	−0.236827000	−2.823121000	−3.161380000
H	1.084771000	0.320041000	−5.265796000
H	−0.499643000	0.606380000	−4.588588000
C	2.080024000	2.550712000	−0.641192000
C	0.671526000	2.385051000	−1.130573000
H	2.790081000	2.041188000	−1.300887000
H	2.171562000	2.083496000	0.348540000
H	−0.099588000	2.728015000	−0.435006000
C	0.264299000	1.993007000	−2.360023000
C	1.107635000	1.603693000	−3.545081000
H	−0.810089000	2.078901000	−2.573061000
H	1.142011000	2.467944000	−4.226210000
H	2.142351000	1.408458000	−3.242846000

104

theta3_C2_cis_1,2 E = −1968.312843 a.u.

N	−0.736906000	−1.852656000	0.584022000
P	−2.693180000	0.409884000	0.176275000
P	2.096489000	−1.213158000	0.185526000
Si	2.954158000	4.654095000	−0.570898000
C	1.412983000	3.918976000	−1.417025000
H	1.612484000	3.855606000	−2.496267000
H	0.593254000	4.644665000	−1.310991000
C	4.441608000	3.518120000	−0.835124000
H	4.298698000	2.519190000	−0.405256000
H	5.341078000	3.943163000	−0.373241000
H	4.654616000	3.389689000	−1.903621000
C	3.296674000	6.332615000	−1.359449000
H	3.498951000	6.238626000	−2.433136000
H	4.169832000	6.812937000	−0.901503000

H	2.446343000	7.014787000	−1.240842000
C	2.626958000	4.876192000	1.277747000
H	1.785509000	5.558134000	1.450600000
H	3.502986000	5.303317000	1.780822000
H	2.391017000	3.927811000	1.775427000
C	−2.829833000	2.085287000	0.947642000
C	−1.796015000	2.477454000	1.812182000
H	−0.979670000	1.793602000	2.036002000
C	−1.824856000	3.728849000	2.420777000
H	−1.024448000	4.017020000	3.096909000
C	−2.881281000	4.606094000	2.169647000
H	−2.903714000	5.582658000	2.645348000
C	−3.912245000	4.219789000	1.316916000
H	−4.745432000	4.891444000	1.128214000
C	−3.891106000	2.963676000	0.707780000
H	−4.713357000	2.671079000	0.062109000
C	−4.293968000	0.127864000	−0.700524000
C	−4.594823000	0.902757000	−1.833126000
H	−3.916352000	1.691393000	−2.152703000
C	−5.767464000	0.676653000	−2.551280000
H	−5.997177000	1.291989000	−3.417213000
C	−6.642619000	−0.338318000	−2.159515000
H	−7.555125000	−0.516690000	−2.721544000
C	−6.343099000	−1.118691000	−1.044392000
H	−7.022457000	−1.907686000	−0.733088000
C	−5.176239000	−0.887446000	−0.314444000
H	−4.956295000	−1.493501000	0.559438000
C	−2.652446000	−0.718438000	1.609178000
C	−1.647436000	−1.719940000	1.639722000
C	−1.587001000	−2.513052000	2.808965000
H	−0.816430000	−3.271022000	2.897538000
C	−2.478311000	−2.336216000	3.858311000
H	−2.387768000	−2.970364000	4.736924000
C	−3.468110000	−1.352032000	3.809006000
H	−4.160744000	−1.213712000	4.633397000

C	−3.532865000	−0.539654000	2.685105000
H	−4.273809000	0.254821000	2.634771000
C	0.037738000	−3.028895000	0.533335000
C	−0.526602000	−4.318695000	0.588323000
H	−1.595895000	−4.417467000	0.755918000
C	0.259445000	−5.453513000	0.416728000
H	−0.205437000	−6.435383000	0.456648000
C	1.632264000	−5.340564000	0.180574000
H	2.242827000	−6.228036000	0.042781000
C	2.212891000	−4.076469000	0.128777000
H	3.279961000	−3.978129000	−0.050859000
C	1.434349000	−2.927841000	0.316176000
C	3.814066000	−1.399403000	−0.455398000
C	4.890752000	−1.693048000	0.392898000
H	4.728441000	−1.800709000	1.461823000
C	6.173594000	−1.840568000	−0.131360000
H	7.003089000	−2.067077000	0.532996000
C	6.394102000	−1.695201000	−1.501151000
H	7.396465000	−1.807086000	−1.905175000
C	5.328953000	−1.396853000	−2.350119000
H	5.498804000	−1.272110000	−3.416248000
C	4.045383000	−1.244309000	−1.828773000
H	3.221242000	−0.996474000	−2.493658000
C	2.315299000	−0.642788000	1.927460000
C	2.117832000	−1.493496000	3.020193000
H	1.825795000	−2.526706000	2.858553000
C	2.303308000	−1.018765000	4.318601000
H	2.149742000	−1.686825000	5.161760000
C	2.689334000	0.302823000	4.534675000
H	2.836430000	0.668708000	5.547131000
C	2.892833000	1.154108000	3.447934000
H	3.204277000	2.182368000	3.610563000
C	2.701924000	0.686455000	2.149816000
H	2.871572000	1.354829000	1.309378000
C	0.539114000	−2.421252000	−3.565815000

C	−0.364503000	−2.884380000	−2.666566000
H	−0.039209000	−3.610876000	−1.927476000
C	−1.769086000	−2.480715000	−2.602555000
C	−2.322131000	−1.453575000	−3.290925000
H	−3.366174000	−1.191646000	−3.156989000
H	−1.771587000	−0.902003000	−4.050490000
Y	−0.340407000	−0.463329000	−1.249037000
H	−2.387993000	−3.022255000	−1.889434000
H	1.572943000	−2.751815000	−3.524829000
H	0.256939000	−1.819342000	−4.420834000
C	0.912687000	2.540037000	−0.926242000
C	−0.322116000	2.115164000	−1.700510000
H	1.729624000	1.808886000	−1.028868000
H	0.677092000	2.598381000	0.144213000
H	−1.212020000	2.700328000	−1.476489000
C	−0.264558000	1.517818000	−2.961679000
C	0.747247000	0.662885000	−3.429776000
H	−1.183582000	1.548242000	−3.553472000
H	0.691627000	0.309056000	−4.453839000
H	1.763620000	0.767405000	−3.047678000

104

theta3_TS2_cis_1,2 E = −1968.304219 a.u. Imaginary frequency=241i

N	−0.671646000	−1.869734000	0.595255000
P	−2.743754000	0.293713000	0.131932000
P	2.153305000	−1.199942000	0.155048000
Si	2.744431000	4.793215000	−0.459962000
C	1.231447000	4.001400000	−1.312588000
H	1.389872000	4.054386000	−2.399072000
H	0.357922000	4.637345000	−1.108004000
C	4.285460000	3.756131000	−0.807081000
H	4.210653000	2.735362000	−0.412465000
H	5.171629000	4.214167000	−0.351197000
H	4.477114000	3.680525000	−1.884601000
C	2.960919000	6.521039000	−1.180646000
H	3.147272000	6.488915000	−2.260735000

H	3.810060000	7.036615000	−0.716011000
H	2.070791000	7.139875000	−1.015550000
C	2.438602000	4.912296000	1.400371000
H	1.581035000	5.560513000	1.617646000
H	3.309477000	5.339715000	1.912154000
H	2.236045000	3.934561000	1.853700000
C	−2.840919000	1.985392000	0.885563000
C	−1.823197000	2.349662000	1.781400000
H	−1.050620000	1.632656000	2.052203000
C	−1.817229000	3.613574000	2.365053000
H	−1.031888000	3.877624000	3.068226000
C	−2.822479000	4.532217000	2.058120000
H	−2.820129000	5.517162000	2.516866000
C	−3.836690000	4.175186000	1.172967000
H	−4.631898000	4.878806000	0.941412000
C	−3.849431000	2.907497000	0.587719000
H	−4.660670000	2.638305000	−0.081644000
C	−4.390616000	0.017815000	−0.654998000
C	−4.702107000	0.735667000	−1.820846000
H	−3.999453000	1.466637000	−2.216770000
C	−5.910943000	0.523885000	−2.479787000
H	−6.145594000	1.093878000	−3.374874000
C	−6.814183000	−0.422688000	−1.993832000
H	−7.754799000	−0.592703000	−2.510225000
C	−6.503817000	−1.150402000	−0.846652000
H	−7.202222000	−1.890427000	−0.465187000
C	−5.299201000	−0.932351000	−0.176304000
H	−5.068303000	−1.504334000	0.717149000
C	−2.632538000	−0.794992000	1.594535000
C	−1.590832000	−1.757631000	1.642407000
C	−1.501459000	−2.533448000	2.821453000
H	−0.708249000	−3.267669000	2.913319000
C	−2.391484000	−2.367675000	3.872871000
H	−2.278775000	−2.985969000	4.760177000
C	−3.407707000	−1.410601000	3.815307000

H	−4.097000000	−1.277049000	4.643315000
C	−3.506907000	−0.621763000	2.677500000
H	−4.272985000	0.148140000	2.620604000
C	0.114944000	−3.037413000	0.522041000
C	−0.436080000	−4.332864000	0.569678000
H	−1.504467000	−4.442431000	0.737120000
C	0.363608000	−5.458879000	0.406756000
H	−0.090573000	−6.445765000	0.445466000
C	1.736898000	−5.331137000	0.183389000
H	2.358943000	−6.212068000	0.055178000
C	2.303688000	−4.060975000	0.129724000
H	3.370726000	−3.951607000	−0.043275000
C	1.510779000	−2.920641000	0.307988000
C	3.871154000	−1.385496000	−0.488906000
C	4.965690000	−1.591209000	0.361937000
H	4.819611000	−1.630454000	1.437716000
C	6.245840000	−1.741728000	−0.168812000
H	7.088783000	−1.899697000	0.498442000
C	6.446218000	−1.689433000	−1.548034000
H	7.446040000	−1.804781000	−1.957279000
C	5.362565000	−1.482572000	−2.401094000
H	5.515077000	−1.435325000	−3.476056000
C	4.082924000	−1.325748000	−1.872676000
H	3.243097000	−1.155452000	−2.542322000
C	2.378895000	−0.604426000	1.887822000
C	2.177067000	−1.438782000	2.992183000
H	1.878436000	−2.472091000	2.844251000
C	2.367413000	−0.948401000	4.284084000
H	2.209627000	−1.604221000	5.136017000
C	2.764672000	0.372567000	4.482428000
H	2.916789000	0.750433000	5.489730000
C	2.973537000	1.207646000	3.384143000
H	3.294696000	2.234978000	3.532903000
C	2.775658000	0.724620000	2.092661000
H	2.949410000	1.380601000	1.243163000

C	0.442103000	−1.593839000	−3.832318000
C	−0.007210000	−2.501281000	−2.865935000
H	0.704495000	−3.172036000	−2.390600000
C	−1.404120000	−2.644848000	−2.541123000
C	−2.370281000	−1.726100000	−2.838331000
H	−3.394809000	−1.866924000	−2.515159000
H	−2.200892000	−0.910335000	−3.543250000
Y	−0.346680000	−0.559744000	−1.270636000
H	−1.666218000	−3.481168000	−1.896233000
H	1.480337000	−1.639850000	−4.144639000
H	−0.252537000	−1.273617000	−4.603049000
C	0.884636000	2.541571000	−0.934263000
C	−0.325961000	2.102519000	−1.728659000
H	1.754599000	1.899240000	−1.125650000
H	0.673976000	2.485773000	0.141270000
H	−1.244641000	2.626383000	−1.471242000
C	−0.285596000	1.484758000	−2.965112000
C	0.768579000	0.674282000	−3.487917000
H	−1.202577000	1.513929000	−3.559179000
H	0.820467000	0.602399000	−4.568466000
H	1.755215000	0.759200000	−3.027606000

104

theta3_P2_cis_1,2 E = −1968.334975 a.u.

N	−1.189453000	−1.761814000	0.495729000
P	−2.749252000	0.766526000	0.179547000
P	1.749220000	−1.647151000	0.195421000
Si	4.196110000	4.427317000	0.118209000
C	2.438308000	4.048803000	−0.530305000
H	2.324080000	4.521787000	−1.515890000
H	1.715936000	4.552012000	0.129097000
C	5.476338000	3.659951000	−1.037259000
H	5.408456000	2.565380000	−1.062437000
H	6.491964000	3.912075000	−0.708990000
H	5.365518000	4.028184000	−2.064423000
C	4.393588000	6.300609000	0.165904000

H	4.272835000	6.743835000	−0.829752000
H	5.388883000	6.581177000	0.530944000
H	3.656431000	6.768350000	0.829484000
C	4.387791000	3.704736000	1.852933000
H	3.613616000	4.077694000	2.534651000
H	5.359359000	3.979234000	2.281310000
H	4.338347000	2.608945000	1.848692000
C	−2.740777000	2.397347000	1.050607000
C	−1.725355000	2.620248000	1.994499000
H	−1.011334000	1.831618000	2.226449000
C	−1.649751000	3.834073000	2.672404000
H	−0.869320000	3.990588000	3.412518000
C	−2.579541000	4.841794000	2.410059000
H	−2.520820000	5.788582000	2.939731000
C	−3.588559000	4.625282000	1.474440000
H	−4.322493000	5.401265000	1.274253000
C	−3.673150000	3.408157000	0.796154000
H	−4.474305000	3.247909000	0.080721000
C	−4.282875000	0.743057000	−0.845134000
C	−4.325556000	1.548550000	−1.994098000
H	−3.488014000	2.200410000	−2.238158000
C	−5.445466000	1.537824000	−2.822000000
H	−5.473242000	2.174081000	−3.702554000
C	−6.525603000	0.706483000	−2.522288000
H	−7.396886000	0.692340000	−3.171155000
C	−6.483046000	−0.105971000	−1.390816000
H	−7.321779000	−0.755126000	−1.154327000
C	−5.368671000	−0.089043000	−0.551711000
H	−5.345450000	−0.723323000	0.329249000
C	−2.967374000	−0.460877000	1.520997000
C	−2.160941000	−1.626255000	1.483875000
C	−2.344992000	−2.576984000	2.507994000
H	−1.732432000	−3.473308000	2.514937000
C	−3.276206000	−2.371253000	3.517871000
H	−3.386472000	−3.122833000	4.295676000

C	−4.051979000	−1.209921000	3.558561000
H	−4.770325000	−1.049996000	4.356765000
C	−3.884168000	−0.257439000	2.559948000
H	−4.471377000	0.657639000	2.579649000
C	−0.692769000	−3.012430000	0.109402000
C	−1.518519000	−4.130394000	−0.142549000
H	−2.582051000	−4.051709000	0.064583000
C	−0.989409000	−5.300758000	−0.663125000
H	−1.645715000	−6.146618000	−0.851029000
C	0.375483000	−5.396106000	−0.967077000
H	0.782084000	−6.314206000	−1.381071000
C	1.209574000	−4.313808000	−0.722371000
H	2.271474000	−4.388488000	−0.938400000
C	0.699256000	−3.129425000	−0.164343000
C	3.448582000	−2.102999000	−0.350345000
C	4.271942000	−2.935761000	0.420546000
H	3.917534000	−3.325531000	1.370837000
C	5.552169000	−3.257736000	−0.024394000
H	6.184823000	−3.903507000	0.578454000
C	6.022511000	−2.751071000	−1.236567000
H	7.022462000	−3.002776000	−1.579093000
C	5.212682000	−1.914548000	−2.003308000
H	5.579431000	−1.511290000	−2.943485000
C	3.932546000	−1.586724000	−1.558998000
H	3.305904000	−0.925386000	−2.151820000
C	1.851406000	−1.576481000	2.035389000
C	1.542751000	−2.674918000	2.845779000
H	1.207886000	−3.605218000	2.395366000
C	1.666281000	−2.577484000	4.231337000
H	1.427322000	−3.435309000	4.854261000
C	2.097719000	−1.387684000	4.816358000
H	2.193710000	−1.315907000	5.896231000
C	2.408058000	−0.290215000	4.013274000
H	2.749334000	0.637729000	4.464205000
C	2.282398000	−0.382951000	2.628807000

H	2.533379000	0.474406000	2.008347000
C	0.542738000	0.395419000	−4.312745000
C	0.635569000	−0.955281000	−3.621337000
H	1.600403000	−1.453374000	−3.699086000
C	−0.464282000	−1.777989000	−3.387828000
C	−1.780228000	−1.354796000	−3.093429000
H	−2.550383000	−2.105613000	−2.937714000
H	−2.153177000	−0.413161000	−3.509557000
Y	−0.477201000	−0.465364000	−1.178237000
H	−0.236827000	−2.823121000	−3.161380000
H	1.084771000	0.320041000	−5.265796000
H	−0.499643000	0.606380000	−4.588588000
C	2.080024000	2.550712000	−0.641192000
C	0.671526000	2.385051000	−1.130573000
H	2.790081000	2.041188000	−1.300887000
H	2.171562000	2.083496000	0.348540000
H	−0.099588000	2.728015000	−0.435006000
C	0.264299000	1.993007000	−2.360023000
C	1.107635000	1.603693000	−3.545081000
H	−0.810089000	2.078901000	−2.573061000
H	1.142011000	2.467944000	−4.226210000
H	2.142351000	1.408458000	−3.242846000

104

theta3_C2_trans_1,4 E = −1968.326074 a.u.

N	−1.049410000	−1.547282000	−0.680684000
P	−2.837129000	0.601404000	0.267351000
P	1.757851000	−1.512506000	0.164996000
Si	4.659535000	3.844223000	0.456510000
C	2.834655000	3.566829000	0.926295000
H	2.227464000	4.321603000	0.406822000
H	2.727811000	3.799146000	1.996241000
C	4.901118000	3.521123000	−1.394942000
H	4.671457000	2.482375000	−1.664536000
H	5.940261000	3.709545000	−1.690659000
H	4.266952000	4.177502000	−2.004271000

C	5.079487000	5.641549000	0.840491000
H	4.446347000	6.333764000	0.272554000
H	6.122878000	5.867730000	0.589935000
H	4.941399000	5.864539000	1.905235000
C	5.776384000	2.691011000	1.449535000
H	5.641679000	2.837736000	2.528124000
H	6.832428000	2.884823000	1.224820000
H	5.584569000	1.633871000	1.230501000
C	−3.279570000	1.286914000	1.925331000
C	−2.467888000	0.919395000	3.010164000
H	−1.630017000	0.240922000	2.861785000
C	−2.743613000	1.397261000	4.287972000
H	−2.114023000	1.099917000	5.122457000
C	−3.825096000	2.254646000	4.497379000
H	−4.037039000	2.630712000	5.494419000
C	−4.634154000	2.621975000	3.424879000
H	−5.483166000	3.281717000	3.582446000
C	−4.366547000	2.140450000	2.142286000
H	−5.012870000	2.427033000	1.318136000
C	−4.084649000	1.300425000	−0.903939000
C	−4.063982000	2.678671000	−1.172695000
H	−3.367526000	3.327091000	−0.646775000
C	−4.946869000	3.231634000	−2.097863000
H	−4.930083000	4.301890000	−2.286333000
C	−5.850680000	2.413382000	−2.778086000
H	−6.537573000	2.844295000	−3.501278000
C	−5.873177000	1.043744000	−2.520124000
H	−6.580378000	0.402773000	−3.039881000
C	−4.997311000	0.487247000	−1.586710000
H	−5.029465000	−0.579595000	−1.386226000
C	−3.199714000	−1.180429000	0.397622000
C	−2.230460000	−2.060866000	−0.140886000
C	−2.495700000	−3.443568000	−0.042631000
H	−1.771711000	−4.154699000	−0.427224000
C	−3.656926000	−3.912633000	0.558810000

H	−3.820273000	−4.985790000	0.621717000
C	−4.600543000	−3.033800000	1.095738000
H	−5.500807000	−3.407518000	1.573738000
C	−4.357453000	−1.667598000	1.015037000
H	−5.070214000	−0.963507000	1.437937000
C	−0.274020000	−2.372691000	−1.512943000
C	−0.804631000	−3.044875000	−2.634581000
H	−1.875856000	−2.998913000	−2.814607000
C	0.018105000	−3.763649000	−3.493785000
H	−0.416641000	−4.271507000	−4.351008000
C	1.396628000	−3.832890000	−3.266655000
H	2.036576000	−4.396943000	−3.938931000
C	1.941574000	−3.188681000	−2.160050000
H	3.008605000	−3.255436000	−1.966643000
C	1.121140000	−2.476063000	−1.274861000
C	3.587781000	−1.735226000	0.090967000
C	4.219906000	−2.830371000	0.695142000
H	3.636118000	−3.559489000	1.249590000
C	5.601295000	−2.986317000	0.592391000
H	6.082119000	−3.837909000	1.065953000
C	6.363507000	−2.055769000	−0.113135000
H	7.440100000	−2.180013000	−0.190099000
C	5.742853000	−0.959699000	−0.711808000
H	6.333483000	−0.225406000	−1.252729000
C	4.362741000	−0.796801000	−0.603060000
H	3.888584000	0.072340000	−1.051669000
C	1.235807000	−2.429986000	1.672742000
C	0.860306000	−3.777477000	1.642231000
H	0.846554000	−4.317318000	0.699422000
C	0.506078000	−4.428890000	2.823181000
H	0.215828000	−5.475671000	2.794472000
C	0.526421000	−3.742415000	4.036610000
H	0.251383000	−4.253835000	4.954925000
C	0.900769000	−2.398870000	4.071586000
H	0.921652000	−1.862620000	5.016566000

C	1.250386000	−1.741813000	2.893534000
H	1.544149000	−0.694768000	2.924437000
C	1.932937000	0.980271000	−3.247562000
C	0.723987000	0.418501000	−3.467282000
H	0.651665000	−0.662773000	−3.597400000
C	−0.522542000	1.178345000	−3.523218000
C	−1.737700000	0.593244000	−3.474653000
H	−2.649663000	1.181346000	−3.494863000
H	−1.844953000	−0.489088000	−3.445763000
Y	−0.084000000	0.545953000	−0.729857000
H	−0.446756000	2.262466000	−3.584250000
H	2.833887000	0.375060000	−3.221978000
H	2.057040000	2.057910000	−3.158943000
C	2.220891000	2.170190000	0.682886000
C	0.775894000	2.085255000	1.147838000
H	2.318594000	1.934894000	−0.399071000
H	2.813879000	1.402522000	1.193341000
H	0.631244000	1.863866000	2.203769000
C	−0.232410000	2.824471000	0.512945000
C	−0.244601000	3.147628000	−0.849590000
H	−1.164749000	2.946458000	1.065102000
H	−1.095094000	3.674457000	−1.271507000
H	0.699246000	3.321974000	−1.370109000

104

theta3_TS2_trans_1,4 E = −1968.304891 a.u. Imaginary frequency=286i

N	−0.665691000	−1.872039000	0.645446000
P	−2.657494000	0.345448000	0.161574000
P	2.114281000	−1.177632000	0.170196000
Si	2.694258000	4.740102000	−0.506742000
C	1.285762000	3.919877000	−1.500259000
H	1.594636000	3.887315000	−2.554782000
H	0.410874000	4.585154000	−1.464370000
C	4.244187000	3.664392000	−0.598671000
H	4.104221000	2.680066000	−0.135709000
H	5.081791000	4.148425000	−0.081883000

H	4.553985000	3.501324000	−1.638310000
C	3.031772000	6.421622000	−1.288947000
H	3.341240000	6.322782000	−2.336278000
H	3.833289000	6.949192000	−0.757964000
H	2.142914000	7.063278000	−1.263989000
C	2.163873000	4.964919000	1.292828000
H	1.276101000	5.604292000	1.366143000
H	2.961341000	5.439700000	1.877194000
H	1.923980000	4.010808000	1.777622000
C	−2.673456000	2.067706000	0.849459000
C	−1.728926000	2.397208000	1.833935000
H	−1.054089000	1.636885000	2.220834000
C	−1.674857000	3.689385000	2.351659000
H	−0.950387000	3.928113000	3.125582000
C	−2.554087000	4.669299000	1.888957000
H	−2.514962000	5.675032000	2.298330000
C	−3.492241000	4.347768000	0.910617000
H	−4.191188000	5.100642000	0.555960000
C	−3.554439000	3.053784000	0.391605000
H	−4.308832000	2.814693000	−0.351967000
C	−4.320831000	0.134657000	−0.611733000
C	−4.528757000	0.705502000	−1.877103000
H	−3.732715000	1.273241000	−2.356613000
C	−5.750331000	0.553703000	−2.530100000
H	−5.903133000	1.008426000	−3.505349000
C	−6.771457000	−0.186934000	−1.933525000
H	−7.722616000	−0.311623000	−2.443511000
C	−6.565672000	−0.769665000	−0.683894000
H	−7.356415000	−1.351034000	−0.217407000
C	−5.347761000	−0.610085000	−0.022041000
H	−5.197036000	−1.075716000	0.947019000
C	−2.562149000	−0.707716000	1.652161000
C	−1.558171000	−1.712076000	1.703187000
C	−1.475838000	−2.471635000	2.892054000
H	−0.703639000	−3.228586000	2.982630000

C	−2.339213000	−2.251319000	3.955772000
H	−2.234370000	−2.856128000	4.853210000
C	−3.315919000	−1.253510000	3.898873000
H	−3.980172000	−1.074628000	4.738736000
C	−3.405684000	−0.479692000	2.749391000
H	−4.134366000	0.326119000	2.698760000
C	0.110970000	−3.039646000	0.549114000
C	−0.428300000	−4.339038000	0.631411000
H	−1.482560000	−4.458342000	0.866982000
C	0.368826000	−5.457774000	0.417213000
H	−0.074044000	−6.448205000	0.484778000
C	1.725011000	−5.320364000	0.106984000
H	2.342979000	−6.197636000	−0.060119000
C	2.281681000	−4.047418000	0.026204000
H	3.337872000	−3.931283000	−0.200853000
C	1.493834000	−2.912467000	0.256513000
C	3.836544000	−1.314154000	−0.480872000
C	4.957157000	−1.015870000	0.303573000
H	4.835326000	−0.730878000	1.344119000
C	6.237310000	−1.086732000	−0.246462000
H	7.099393000	−0.853431000	0.372479000
C	6.412806000	−1.458679000	−1.577622000
H	7.411577000	−1.514589000	−2.001482000
C	5.301334000	−1.760779000	−2.365368000
H	5.431274000	−2.055958000	−3.403221000
C	4.021739000	−1.681174000	−1.822519000
H	3.162493000	−1.922745000	−2.443697000
C	2.308435000	−0.663272000	1.929709000
C	2.459597000	−1.604206000	2.955182000
H	2.443065000	−2.665929000	2.725990000
C	2.634214000	−1.180584000	4.271595000
H	2.751832000	−1.916696000	5.062268000
C	2.662203000	0.180663000	4.573652000
H	2.799921000	0.507253000	5.600759000
C	2.516303000	1.122339000	3.555498000

H	2.545019000	2.184030000	3.785538000
C	2.336030000	0.702847000	2.238317000
H	2.226529000	1.442354000	1.449275000
C	0.397888000	−1.719345000	−3.827573000
C	−0.437937000	−2.468145000	−2.969029000
H	−0.026825000	−3.272853000	−2.358055000
C	−1.863610000	−2.220190000	−2.961040000
C	−2.736231000	−2.578112000	−1.983153000
H	−3.786508000	−2.317996000	−2.050319000
H	−2.434500000	−3.218185000	−1.157758000
Y	−0.392080000	−0.692259000	−1.296601000
H	−2.246373000	−1.600530000	−3.777909000
H	1.416242000	−2.061768000	−3.987764000
H	−0.067057000	−1.303541000	−4.719308000
C	0.858705000	2.499126000	−1.062705000
C	−0.225104000	2.000303000	−1.989774000
H	1.732027000	1.835618000	−1.071939000
H	0.484430000	2.534852000	−0.031874000
H	−1.159596000	2.557393000	−1.927306000
C	−0.042502000	1.217748000	−3.105957000
C	1.038783000	0.292527000	−3.368489000
H	−0.862561000	1.201062000	−3.830431000
H	1.363122000	0.253426000	−4.404433000
H	1.907058000	0.351740000	−2.704238000

104

theta3_P2_trans_1,4 E = −1968.325647 a.u.

N	−0.621402000	−2.003201000	0.431802000
P	−2.557160000	0.299311000	0.238215000
P	2.132134000	−1.060546000	0.227368000
Si	2.471480000	4.708577000	−0.714243000
C	1.120728000	3.840559000	−1.740333000
H	1.531490000	3.620319000	−2.735806000
H	0.309955000	4.564070000	−1.910325000
C	4.025550000	3.633878000	−0.654174000
H	3.852461000	2.650922000	−0.199359000

H	4.815360000	4.124963000	−0.072650000
H	4.422323000	3.463134000	−1.662494000
C	2.873874000	6.346188000	−1.558136000
H	3.224340000	6.194419000	−2.586133000
H	3.661776000	6.885631000	−1.018693000
H	1.996027000	7.002024000	−1.600493000
C	1.838940000	5.034276000	1.037220000
H	0.957116000	5.686286000	1.024735000
H	2.606374000	5.530987000	1.643312000
H	1.553563000	4.110600000	1.555244000
C	−2.629779000	1.897385000	1.167112000
C	−1.598493000	2.154547000	2.083761000
H	−0.814608000	1.418328000	2.249760000
C	−1.589046000	3.338898000	2.815469000
H	−0.793033000	3.521241000	3.532111000
C	−2.600799000	4.283309000	2.635238000
H	−2.593358000	5.206579000	3.207946000
C	−3.625419000	4.032366000	1.725972000
H	−4.423731000	4.756947000	1.589303000
C	−3.644095000	2.843965000	0.993853000
H	−4.462553000	2.655764000	0.305853000
C	−4.186117000	0.156983000	−0.623989000
C	−4.454116000	1.009207000	−1.707994000
H	−3.721801000	1.754159000	−2.009779000
C	−5.660468000	0.915749000	−2.399622000
H	−5.862459000	1.592062000	−3.226162000
C	−6.604282000	−0.044405000	−2.031580000
H	−7.543608000	−0.120110000	−2.572311000
C	−6.338111000	−0.904011000	−0.967042000
H	−7.069940000	−1.652442000	−0.675050000
C	−5.137725000	−0.804571000	−0.262418000
H	−4.944898000	−1.474093000	0.570513000
C	−2.532565000	−0.964530000	1.554920000
C	−1.534883000	−1.973661000	1.492291000
C	−1.484732000	−2.875186000	2.580407000

H	−0.719032000	−3.642597000	2.602009000
C	−2.379091000	−2.792786000	3.639211000
H	−2.296172000	−3.509394000	4.452853000
C	−3.360822000	−1.801006000	3.682819000
H	−4.053134000	−1.734743000	4.516352000
C	−3.414745000	−0.884108000	2.641404000
H	−4.145458000	−0.078946000	2.669831000
C	0.268260000	−3.095018000	0.351714000
C	−0.157471000	−4.436360000	0.292547000
H	−1.219342000	−4.654809000	0.375908000
C	0.757330000	−5.472741000	0.134323000
H	0.399763000	−6.498439000	0.091481000
C	2.125303000	−5.206115000	0.024375000
H	2.836516000	−6.018301000	−0.094670000
C	2.569752000	−3.888011000	0.074241000
H	3.630487000	−3.668133000	−0.010634000
C	1.657086000	−2.839284000	0.241655000
C	3.892332000	−1.037046000	−0.315919000
C	4.954364000	−0.997899000	0.596432000
H	4.757262000	−0.971024000	1.664204000
C	6.270569000	−0.989772000	0.135997000
H	7.088750000	−0.957095000	0.850293000
C	6.537324000	−1.024163000	−1.231906000
H	7.564352000	−1.015949000	−1.586358000
C	5.483842000	−1.064232000	−2.145773000
H	5.687215000	−1.086391000	−3.213084000
C	4.167424000	−1.063998000	−1.690875000
H	3.350565000	−1.086344000	−2.408787000
C	2.166382000	−0.533793000	1.993073000
C	2.093056000	−1.452109000	3.046097000
H	2.001881000	−2.513902000	2.836959000
C	2.144291000	−1.006006000	4.366474000
H	2.088416000	−1.725314000	5.178972000
C	2.273353000	0.353961000	4.644499000
H	2.317882000	0.696865000	5.674556000

C	2.350415000	1.273841000	3.598107000
H	2.461988000	2.334083000	3.808789000
C	2.290407000	0.833742000	2.277401000
H	2.357574000	1.556794000	1.468023000
C	0.637901000	−3.045689000	−3.160781000
C	−0.670639000	−2.919976000	−2.856557000
H	−1.090028000	−3.534847000	−2.060988000
C	−1.579462000	−1.989861000	−3.517785000
C	−2.832290000	−1.756956000	−3.076872000
H	−3.493025000	−1.054113000	−3.573408000
H	−3.244281000	−2.293259000	−2.224645000
Y	−0.268498000	−0.500582000	−1.305505000
H	−1.207896000	−1.466898000	−4.398163000
H	1.266938000	−3.752318000	−2.627745000
H	1.093324000	−2.480053000	−3.969153000
C	0.495551000	2.548951000	−1.162170000
C	−0.555096000	1.973827000	−2.094778000
H	1.311992000	1.826379000	−0.971953000
H	0.043013000	2.765510000	−0.186651000
H	−1.504952000	2.504341000	−2.093782000
C	−0.226610000	1.246995000	−3.240002000
C	0.923144000	0.448978000	−3.396002000
H	−1.031090000	1.102880000	−3.965512000
H	1.063637000	−0.076826000	−4.337054000
H	1.856025000	0.758720000	−2.920868000

104

theta3_C2_trans_1,2 E = −1968.318039 a.u.

N	0.878388000	−1.343243000	−1.118720000
P	2.954829000	0.547051000	−0.002976000
P	−1.977694000	−1.540145000	−0.095158000
Si	−3.891414000	4.631130000	0.436648000
C	−2.972520000	3.690004000	−0.942160000
H	−2.159284000	4.324372000	−1.321546000
H	−3.662294000	3.561086000	−1.788446000
C	−2.713421000	4.940773000	1.885049000

H	−2.376505000	4.006861000	2.353149000
H	−3.206674000	5.529918000	2.667638000
H	−1.825013000	5.501305000	1.568219000
C	−4.482246000	6.274128000	−0.271681000
H	−3.642995000	6.884806000	−0.625612000
H	−5.021011000	6.859843000	0.482766000
H	−5.162641000	6.123930000	−1.118365000
C	−5.364838000	3.609847000	1.034376000
H	−6.054077000	3.383126000	0.212007000
H	−5.933888000	4.154300000	1.797597000
H	−5.054173000	2.656505000	1.480272000
C	4.123908000	1.970850000	−0.010022000
C	3.625790000	3.263687000	0.191908000
H	2.559951000	3.413754000	0.338695000
C	4.487669000	4.360175000	0.203561000
H	4.089600000	5.358847000	0.361593000
C	5.855836000	4.172564000	0.017896000
H	6.528419000	5.025856000	0.028008000
C	6.363458000	2.886229000	−0.172158000
H	7.430909000	2.736003000	−0.308974000
C	5.505613000	1.789070000	−0.181841000
H	5.912468000	0.789703000	−0.312315000
C	3.850960000	−0.717383000	1.009077000
C	4.509979000	−0.310014000	2.179565000
H	4.568009000	0.744814000	2.437656000
C	5.130339000	−1.249317000	3.002482000
H	5.654085000	−0.918717000	3.895653000
C	5.090005000	−2.604798000	2.673184000
H	5.579098000	−3.335776000	3.311220000
C	4.429305000	−3.014996000	1.515947000
H	4.399906000	−4.068000000	1.248751000
C	3.811558000	−2.078343000	0.687480000
H	3.305606000	−2.409701000	−0.212694000
C	2.894111000	−0.087795000	−1.741917000
C	1.836014000	−0.972056000	−2.085915000

C	1.725283000	−1.373995000	−3.434432000
H	0.917717000	−2.044652000	−3.713807000
C	2.619493000	−0.927903000	−4.396337000
H	2.505950000	−1.258449000	−5.425620000
C	3.647094000	−0.044851000	−4.053103000
H	4.343991000	0.311438000	−4.805975000
C	3.768348000	0.377627000	−2.735461000
H	4.556212000	1.075481000	−2.469944000
C	0.378181000	−2.650371000	−1.124349000
C	1.151650000	−3.745437000	−1.569492000
H	2.160995000	−3.567621000	−1.928696000
C	0.645038000	−5.037517000	−1.580630000
H	1.275907000	−5.849809000	−1.933021000
C	−0.660608000	−5.300262000	−1.158643000
H	−1.064159000	−6.307677000	−1.190277000
C	−1.438659000	−4.241853000	−0.707960000
H	−2.466157000	−4.422240000	−0.400424000
C	−0.933242000	−2.934168000	−0.663419000
C	−2.563531000	−1.952625000	1.610216000
C	−3.364113000	−1.001607000	2.265825000
H	−3.661047000	−0.090733000	1.748737000
C	−3.805045000	−1.219196000	3.569774000
H	−4.435333000	−0.480533000	4.057860000
C	−3.437541000	−2.384778000	4.244372000
H	−3.779190000	−2.556535000	5.261310000
C	−2.632814000	−3.327492000	3.606190000
H	−2.345763000	−4.238309000	4.125140000
C	−2.195996000	−3.115739000	2.297344000
H	−1.566213000	−3.857472000	1.816466000
C	−3.488910000	−1.661814000	−1.157954000
C	−4.778241000	−1.848548000	−0.650170000
H	−4.940396000	−1.955359000	0.417981000
C	−5.869167000	−1.918638000	−1.518298000
H	−6.866066000	−2.069911000	−1.112977000
C	−5.682890000	−1.806339000	−2.893839000

H	−6.534208000	−1.863877000	−3.566386000
C	−4.396696000	−1.629101000	−3.406517000
H	−4.242071000	−1.552206000	−4.479462000
C	−3.306057000	−1.553778000	−2.544978000
H	−2.305788000	−1.431520000	−2.954528000
C	−2.400428000	2.301039000	−0.579040000
C	−1.695158000	1.623965000	−1.736193000
H	−3.210411000	1.650621000	−0.226325000
H	−1.727066000	2.443121000	0.294869000
C	−0.448250000	2.025865000	−2.206545000
C	0.534417000	2.687991000	−1.439155000
H	1.489715000	2.909902000	−1.904504000
H	0.220358000	3.417403000	−0.687717000
H	−2.329377000	1.042593000	−2.400118000
C	−0.260017000	1.414514000	2.516844000
C	0.645818000	0.497812000	2.941907000
H	0.054675000	2.416821000	2.227167000
H	−1.328871000	1.243398000	2.642447000
C	0.316150000	−0.797802000	3.504748000
C	1.260408000	−1.679577000	3.872254000
H	0.994426000	−2.636928000	4.310265000
H	2.320458000	−1.472084000	3.746734000
H	1.707467000	0.739357000	2.881977000
Y	0.075764000	0.533719000	−0.188226000
H	−0.737318000	−1.032241000	3.641876000
H	−0.114899000	1.575129000	−3.144288000

104

theta3_TS2_trans_1,2 E = −1968.292829 a.u. Imaginary frequency=291i

N	1.285701000	−1.637231000	−0.051905000
P	−1.715627000	−1.647628000	0.021332000
P	3.031258000	0.745191000	0.281146000
Si	−5.301127000	3.550107000	0.345670000
C	−3.399831000	3.481700000	0.515247000
H	−3.128818000	3.239301000	1.551377000
H	−3.045993000	4.511612000	0.353615000

C	−6.043483000	1.818369000	0.427756000
H	−5.684483000	1.177689000	−0.386687000
H	−7.136481000	1.864508000	0.347329000
H	−5.802564000	1.317277000	1.372019000
C	−5.965210000	4.613097000	1.754328000
H	−5.736497000	4.175051000	2.733397000
H	−7.054724000	4.718892000	1.687705000
H	−5.536363000	5.622224000	1.734057000
C	−5.723814000	4.343853000	−1.316100000
H	−5.306504000	5.355032000	−1.396051000
H	−6.810269000	4.428946000	−1.439669000
H	−5.348677000	3.760237000	−2.166012000
C	−3.265810000	−1.574345000	1.019792000
C	−3.198238000	−1.019158000	2.307005000
H	−2.249877000	−0.658366000	2.699408000
C	−4.332470000	−0.971273000	3.112871000
H	−4.265675000	−0.551569000	4.113070000
C	−5.547526000	−1.471143000	2.640816000
H	−6.431655000	−1.437506000	3.271338000
C	−5.621165000	−2.018595000	1.361924000
H	−6.562611000	−2.414737000	0.991364000
C	−4.486360000	−2.072262000	0.551506000
H	−4.553810000	−2.515234000	−0.437595000
C	−2.165618000	−2.503625000	−1.550504000
C	−2.871232000	−1.786322000	−2.529369000
H	−3.179139000	−0.759300000	−2.339039000
C	−3.205423000	−2.389826000	−3.740015000
H	−3.761829000	−1.831313000	−4.488030000
C	−2.823955000	−3.709072000	−3.989309000
H	−3.079728000	−4.178590000	−4.935119000
C	−2.118021000	−4.424001000	−3.022547000
H	−1.825269000	−5.453172000	−3.212222000
C	−1.789594000	−3.827181000	−1.805216000
H	−1.246104000	−4.389618000	−1.051308000
C	−0.630644000	−2.782128000	0.957307000

C	0.771088000	−2.620365000	0.800892000
C	1.598534000	−3.464190000	1.575083000
H	2.675878000	−3.351470000	1.509524000
C	1.064534000	−4.424874000	2.423841000
H	1.740138000	−5.049887000	3.002744000
C	−0.316731000	−4.586815000	2.551788000
H	−0.731307000	−5.341329000	3.213264000
C	−1.154545000	−3.752372000	1.821723000
H	−2.232315000	−3.850426000	1.920578000
C	2.593581000	−1.854663000	−0.541420000
C	2.954468000	−3.074021000	−1.147516000
H	2.205244000	−3.856637000	−1.228532000
C	4.239728000	−3.288787000	−1.627460000
H	4.484681000	−4.241039000	−2.090978000
C	5.216837000	−2.295390000	−1.514345000
H	6.227479000	−2.471243000	−1.871143000
C	4.880448000	−1.077381000	−0.934719000
H	5.635694000	−0.302150000	−0.836862000
C	3.581078000	−0.842008000	−0.467625000
C	4.005945000	2.117512000	−0.471558000
C	4.104783000	3.329357000	0.232607000
H	3.729772000	3.401401000	1.250744000
C	4.703675000	4.442208000	−0.353673000
H	4.784069000	5.367611000	0.210369000
C	5.203601000	4.368082000	−1.654052000
H	5.674547000	5.235346000	−2.108245000
C	5.100312000	3.173841000	−2.364719000
H	5.492275000	3.104849000	−3.376036000
C	4.502868000	2.056169000	−1.781567000
H	4.437920000	1.132076000	−2.347329000
C	3.584302000	0.652185000	2.042222000
C	4.874954000	1.026898000	2.438891000
H	5.573443000	1.438997000	1.716066000
C	5.269130000	0.876804000	3.766802000
H	6.271307000	1.171820000	4.065890000

C	4.385317000	0.347230000	4.707197000
H	4.696724000	0.232292000	5.741779000
C	3.104376000	−0.038919000	4.316162000
H	2.415079000	−0.461093000	5.042541000
C	2.703533000	0.113525000	2.989604000
H	1.710619000	−0.210915000	2.689565000
C	−2.660824000	2.541475000	−0.458175000
C	−1.194441000	2.925483000	−0.620082000
H	−3.136603000	2.583913000	−1.446811000
H	−2.777514000	1.500236000	−0.121292000
C	−0.281979000	2.730162000	0.482306000
C	−0.370211000	1.806750000	1.501551000
H	0.381569000	1.787128000	2.284403000
H	−1.303947000	1.280847000	1.705291000
H	−1.095433000	3.939348000	−1.008327000
C	−0.599121000	2.338228000	−2.506810000
C	−0.117600000	1.044241000	−2.864277000
H	−1.596622000	2.599347000	−2.849366000
H	0.116702000	3.150757000	−2.631124000
C	1.297105000	0.826652000	−3.129659000
C	1.879684000	−0.363562000	−3.401441000
H	2.944878000	−0.444544000	−3.587841000
H	1.300179000	−1.280239000	−3.494261000
H	−0.825327000	0.290384000	−3.217038000
Y	0.293259000	0.371229000	−0.578595000
H	1.939068000	1.709961000	−3.070785000
H	0.636923000	3.321794000	0.439886000

104

theta3_P2_trans_1,2 E = −1968.342423 a.u.

N	−1.711610000	1.619725000	−0.219095000
P	1.237059000	2.018867000	0.035845000
P	−2.889070000	−1.068571000	0.229275000
Si	5.833785000	−3.144995000	0.486896000
C	3.952107000	−3.461337000	0.440051000
H	3.507469000	−3.051331000	1.357561000

H	3.809873000	−4.551023000	0.508362000
C	6.189434000	−1.302749000	0.258456000
H	5.857198000	−0.941826000	−0.722866000
H	7.266767000	−1.107882000	0.326104000
H	5.696931000	−0.693129000	1.025548000
C	6.476195000	−3.720388000	2.164483000
H	6.016937000	−3.158820000	2.987092000
H	7.561903000	−3.585469000	2.239475000
H	6.265835000	−4.783803000	2.330690000
C	6.665815000	−4.126055000	−0.895207000
H	6.477860000	−5.201749000	−0.792696000
H	7.752724000	−3.981215000	−0.875136000
H	6.317173000	−3.820872000	−1.889366000
C	2.612925000	1.961187000	1.264234000
C	2.348332000	1.388448000	2.518056000
H	1.349379000	1.028233000	2.755188000
C	3.350693000	1.313331000	3.481170000
H	3.132941000	0.878625000	4.453082000
C	4.627709000	1.803547000	3.201730000
H	5.409103000	1.747157000	3.954506000
C	4.894385000	2.373888000	1.958791000
H	5.883473000	2.766612000	1.739546000
C	3.892657000	2.454410000	0.990179000
H	4.109948000	2.912952000	0.030183000
C	1.886393000	2.949317000	−1.419771000
C	2.772659000	2.293644000	−2.289151000
H	3.098926000	1.277059000	−2.074510000
C	3.261854000	2.944327000	−3.419721000
H	3.954693000	2.431952000	−4.081702000
C	2.856877000	4.249893000	−3.700725000
H	3.232443000	4.756375000	−4.585473000
C	1.970400000	4.902803000	−2.845387000
H	1.655595000	5.920237000	−3.061003000
C	1.485270000	4.259006000	−1.707217000
H	0.797818000	4.774433000	−1.043048000

C	−0.044039000	3.068796000	0.806964000
C	−1.400552000	2.745952000	0.541921000
C	−2.380442000	3.577484000	1.124122000
H	−3.429794000	3.360013000	0.952150000
C	−2.029373000	4.657336000	1.924221000
H	−2.816442000	5.268489000	2.359211000
C	−0.690923000	4.956962000	2.187665000
H	−0.422144000	5.799106000	2.817836000
C	0.294206000	4.151581000	1.627888000
H	1.342392000	4.362221000	1.825324000
C	−2.982572000	1.513503000	−0.814165000
C	−3.550560000	2.548820000	−1.583135000
H	−3.009062000	3.485847000	−1.677735000
C	−4.770332000	2.377399000	−2.222897000
H	−5.182243000	3.192040000	−2.813055000
C	−5.462359000	1.165173000	−2.129298000
H	−6.415101000	1.035014000	−2.634048000
C	−4.921007000	0.126304000	−1.382559000
H	−5.452060000	−0.818166000	−1.304572000
C	−3.700311000	0.292438000	−0.711373000
C	−3.860327000	−2.584847000	−0.153033000
C	−4.973540000	−2.959395000	0.610746000
H	−5.278535000	−2.356745000	1.461640000
C	−5.690377000	−4.109173000	0.283253000
H	−6.551973000	−4.393717000	0.880989000
C	−5.305723000	−4.890655000	−0.805894000
H	−5.866450000	−5.786638000	−1.057294000
C	−4.197839000	−4.524194000	−1.570047000
H	−3.893553000	−5.132470000	−2.417571000
C	−3.474646000	−3.379694000	−1.241202000
H	−2.608222000	−3.103864000	−1.839121000
C	−3.175457000	−0.754861000	2.020436000
C	−3.967928000	0.302102000	2.477758000
H	−4.453031000	0.963434000	1.766051000
C	−4.140469000	0.501496000	3.847615000

H	−4.759477000	1.323298000	4.197090000
C	−3.527498000	−0.350870000	4.764501000
H	−3.666092000	−0.193372000	5.830543000
C	−2.740205000	−1.411014000	4.312436000
H	−2.268757000	−2.083834000	5.023739000
C	−2.560220000	−1.611028000	2.946020000
H	−1.958528000	−2.450568000	2.602018000
C	3.218202000	−2.937312000	−0.802335000
C	1.754126000	−3.393414000	−0.922017000
H	3.736049000	−3.293203000	−1.703254000
H	3.280927000	−1.840166000	−0.841918000
C	0.784133000	−2.747627000	0.039067000
C	0.996728000	−1.726589000	0.897075000
H	0.234349000	−1.442687000	1.624453000
H	1.968307000	−1.248899000	1.003186000
H	1.734310000	−4.471013000	−0.691386000
C	1.176787000	−3.217328000	−2.346269000
C	0.866641000	−1.770891000	−2.629584000
H	1.888333000	−3.633722000	−3.074737000
H	0.256230000	−3.809119000	−2.444053000
C	−0.255495000	−1.295493000	−3.281835000
C	−0.607804000	0.077398000	−3.380018000
H	−1.516820000	0.351114000	−3.909159000
H	0.203238000	0.809394000	−3.484940000
H	1.709531000	−1.074254000	−2.512571000
Y	−0.440413000	−0.124387000	−0.946624000
H	−1.034338000	−2.026224000	−3.521524000
H	−0.208657000	−3.209095000	0.043844000

104

theta1_TS2_cis_1,4 E = −1968.283555 a.u. Imaginary frequency=58i

N	1.924490000	1.329744000	−0.685782000
P	−0.758435000	2.349862000	0.133366000
P	2.603824000	−1.397492000	0.234854000
Si	−6.753597000	−2.542325000	0.343553000
C	−5.095039000	−3.298502000	0.892981000

H	−5.272792000	−4.346573000	1.175298000
H	−4.771349000	−2.795450000	1.815897000
C	−7.425240000	−3.470769000	−1.157744000
H	−6.742423000	−3.420457000	−2.014700000
H	−8.384625000	−3.048384000	−1.480418000
H	−7.594828000	−4.530396000	−0.931169000
C	−7.968210000	−2.664844000	1.780938000
H	−8.122248000	−3.706657000	2.086486000
H	−8.947648000	−2.254762000	1.506397000
H	−7.612896000	−2.111521000	2.658650000
C	−6.497658000	−0.721321000	−0.115397000
H	−6.063721000	−0.152096000	0.716214000
H	−7.455280000	−0.251896000	−0.372057000
H	−5.838144000	−0.611803000	−0.985867000
C	−2.382392000	3.208618000	0.055908000
C	−3.495814000	2.515193000	−0.434212000
H	−3.391807000	1.485428000	−0.766883000
C	−4.744837000	3.132315000	−0.485281000
H	−5.603933000	2.582569000	−0.859737000
C	−4.888385000	4.445723000	−0.041016000
H	−5.861847000	4.927159000	−0.074770000
C	−3.785237000	5.139699000	0.458711000
H	−3.898982000	6.160233000	0.813904000
C	−2.536410000	4.525258000	0.512766000
H	−1.685924000	5.064245000	0.921512000
C	−0.016194000	2.881691000	1.732170000
C	−0.433569000	2.221180000	2.896538000
H	−1.167280000	1.418847000	2.838542000
C	0.073574000	2.602303000	4.136913000
H	−0.260705000	2.093299000	5.036968000
C	1.006951000	3.636110000	4.221548000
H	1.404191000	3.931394000	5.188733000
C	1.428641000	4.290606000	3.065059000
H	2.154403000	5.096768000	3.129361000
C	0.920328000	3.916901000	1.821321000

H	1.252442000	4.429432000	0.922870000
C	0.287624000	3.071339000	−1.208766000
C	1.539748000	2.446105000	−1.456291000
C	2.312597000	2.920471000	−2.535914000
H	3.271365000	2.449547000	−2.736003000
C	1.865261000	3.963876000	−3.335530000
H	2.483376000	4.308860000	−4.160405000
C	0.623345000	4.560332000	−3.095400000
H	0.273715000	5.374808000	−3.723166000
C	−0.162046000	4.105791000	−2.042559000
H	−1.128371000	4.564191000	−1.852655000
C	3.200929000	1.286331000	−0.120068000
C	4.022474000	2.422131000	0.028744000
H	3.672549000	3.384318000	−0.331088000
C	5.266068000	2.334551000	0.643222000
H	5.867996000	3.234352000	0.744173000
C	5.742082000	1.120741000	1.142669000
H	6.712093000	1.060357000	1.626471000
C	4.943196000	−0.010417000	1.018620000
H	5.286336000	−0.962419000	1.416554000
C	3.695041000	0.059378000	0.390511000
C	2.774131000	−2.314872000	1.825568000
C	3.578988000	−3.451142000	1.966678000
H	4.130503000	−3.839896000	1.115939000
C	3.680959000	−4.085440000	3.205041000
H	4.309293000	−4.966254000	3.306144000
C	2.987148000	−3.590943000	4.307731000
H	3.068947000	−4.088223000	5.270252000
C	2.190166000	−2.453318000	4.174532000
H	1.652369000	−2.059661000	5.032763000
C	2.080192000	−1.820307000	2.939434000
H	1.467947000	−0.924933000	2.850515000
C	3.353875000	−2.485310000	−1.055026000
C	4.400756000	−2.044800000	−1.873243000
H	4.849469000	−1.070074000	−1.703794000

C	4.879907000	−2.863058000	−2.897548000
H	5.697001000	−2.514929000	−3.523739000
C	4.324449000	−4.123516000	−3.109933000
H	4.704269000	−4.760032000	−3.904290000
C	3.283411000	−4.570610000	−2.293718000
H	2.853742000	−5.556870000	−2.448099000
C	2.793781000	−3.753909000	−1.277336000
H	1.988611000	−4.112435000	−0.639085000
C	−1.156290000	−1.837901000	0.271957000
C	−1.499349000	−3.213902000	0.597850000
H	−0.691564000	−3.807020000	1.027743000
C	−2.680650000	−3.834902000	0.383713000
C	−3.951996000	−3.235628000	−0.140973000
H	−4.258838000	−3.778581000	−1.047174000
H	−3.792614000	−2.194395000	−0.450051000
Y	0.213278000	−0.146296000	−0.842230000
H	−2.748861000	−4.886939000	0.662460000
H	−2.037497000	−1.183419000	0.168941000
H	−0.468553000	−1.419679000	1.044795000
C	−0.616948000	−2.423804000	−2.132026000
C	0.105095000	−1.568504000	−2.952956000
H	−1.700641000	−2.394797000	−2.083187000
H	−0.171678000	−3.348872000	−1.786278000
H	1.128947000	−1.834258000	−3.212906000
C	−0.409547000	−0.314633000	−3.460957000
C	−1.547109000	0.307003000	−3.031698000
H	0.239354000	0.231956000	−4.145004000
H	−1.832391000	1.279169000	−3.418669000
H	−2.288005000	−0.206646000	−2.417124000

104

theta1_P2_cis_1,4 E = −1968.323624 a.u.

N	1.734753000	1.693432000	−0.333688000
P	−1.242003000	1.960977000	0.029373000
P	2.970288000	−0.985217000	0.185259000
Si	−6.433897000	−2.693860000	0.314911000

C	−4.690479000	−3.360726000	0.702109000
H	−4.821889000	−4.357257000	1.150845000
H	−4.231424000	−2.744960000	1.489287000
C	−7.326267000	−3.907692000	−0.823271000
H	−6.811712000	−4.032671000	−1.783772000
H	−8.342340000	−3.558335000	−1.043612000
H	−7.414620000	−4.899038000	−0.362536000
C	−7.369613000	−2.533311000	1.945248000
H	−7.436080000	−3.497199000	2.464096000
H	−8.394034000	−2.178875000	1.779263000
H	−6.881545000	−1.823833000	2.624499000
C	−6.314307000	−1.007626000	−0.529776000
H	−5.797489000	−0.271596000	0.097775000
H	−7.314010000	−0.610566000	−0.744296000
H	−5.777585000	−1.069421000	−1.484301000
C	−2.962930000	2.458987000	−0.392069000
C	−3.485739000	2.069597000	−1.633500000
H	−2.878819000	1.485059000	−2.320232000
C	−4.776472000	2.442811000	−1.998862000
H	−5.171899000	2.142614000	−2.965348000
C	−5.561515000	3.192467000	−1.121837000
H	−6.571013000	3.477690000	−1.404607000
C	−5.050524000	3.571511000	0.118207000
H	−5.659226000	4.154043000	0.804363000
C	−3.754110000	3.210449000	0.484997000
H	−3.362850000	3.515636000	1.451031000
C	−1.022337000	2.365670000	1.810158000
C	−1.385731000	1.394726000	2.754458000
H	−1.789024000	0.437457000	2.427300000
C	−1.252887000	1.656458000	4.116647000
H	−1.543205000	0.902812000	4.843665000
C	−0.747574000	2.884691000	4.543524000
H	−0.640026000	3.088007000	5.605375000
C	−0.381977000	3.852081000	3.607792000
H	0.008597000	4.810216000	3.939675000

C	−0.518647000	3.597411000	2.243956000
H	−0.235841000	4.355545000	1.518946000
C	−0.159647000	3.131306000	−0.914469000
C	1.231423000	2.849935000	−0.966823000
C	2.054845000	3.698850000	−1.731973000
H	3.118805000	3.483889000	−1.783253000
C	1.524693000	4.786085000	−2.412788000
H	2.182335000	5.425740000	−2.995523000
C	0.152550000	5.051842000	−2.365113000
H	−0.261123000	5.902768000	−2.898440000
C	−0.682259000	4.220171000	−1.628927000
H	−1.748691000	4.422032000	−1.591544000
C	2.839514000	1.783441000	0.519905000
C	3.272999000	3.000647000	1.081840000
H	2.753568000	3.918851000	0.827101000
C	4.339988000	3.041588000	1.971043000
H	4.641278000	3.998914000	2.389015000
C	5.014356000	1.876958000	2.343153000
H	5.843620000	1.913948000	3.042870000
C	4.598103000	0.664221000	1.805982000
H	5.101812000	−0.255658000	2.093037000
C	3.534203000	0.606818000	0.898357000
C	2.977460000	−2.168705000	1.602535000
C	3.987609000	−3.114065000	1.812110000
H	4.822404000	−3.185330000	1.121263000
C	3.927728000	−3.969830000	2.912600000
H	4.717831000	−4.699690000	3.067044000
C	2.866937000	−3.886961000	3.812301000
H	2.824622000	−4.554949000	4.668061000
C	1.860317000	−2.941076000	3.613718000
H	1.034061000	−2.867008000	4.315626000
C	1.912758000	−2.090872000	2.512593000
H	1.130656000	−1.344067000	2.379762000
C	4.310241000	−1.544574000	−0.950979000
C	5.352880000	−0.691139000	−1.329285000

H	5.423948000	0.304607000	−0.901813000
C	6.311400000	−1.121494000	−2.247812000
H	7.118693000	−0.452088000	−2.532425000
C	6.241565000	−2.402956000	−2.789774000
H	6.992642000	−2.736398000	−3.500452000
C	5.205594000	−3.260140000	−2.414079000
H	5.149157000	−4.263153000	−2.828502000
C	4.239546000	−2.831629000	−1.507508000
H	3.437297000	−3.508151000	−1.220673000
C	−0.618179000	−2.653758000	−0.850244000
C	−1.199884000	−3.918267000	−0.279887000
H	−0.449915000	−4.652287000	0.015396000
C	−2.489717000	−4.252396000	−0.153723000
C	−3.723268000	−3.468827000	−0.492868000
H	−4.227346000	−3.985422000	−1.323833000
H	−3.475016000	−2.467647000	−0.867541000
Y	0.517920000	−0.022831000	−1.025810000
H	−2.693001000	−5.244096000	0.254140000
H	−1.401775000	−1.874745000	−0.877480000
H	0.182182000	−2.347057000	−0.139459000
C	−0.000008000	−2.887637000	−2.260221000
C	0.991182000	−1.872438000	−2.820005000
H	−0.840291000	−3.016315000	−2.957120000
H	0.510370000	−3.856138000	−2.216841000
H	1.997424000	−2.241103000	−2.998123000
C	0.619226000	−0.710620000	−3.487305000
C	−0.587273000	−0.005710000	−3.278309000
H	1.415572000	−0.182964000	−4.019382000
H	−0.775529000	0.894082000	−3.858269000
H	−1.486102000	−0.579541000	−3.026872000

104

theta1_C2_trans_1,4 E = −1968.321524 a.u.

N	−1.013213000	−1.502386000	−0.858151000
P	1.758794000	−1.701223000	0.179799000
P	−2.889140000	0.585476000	0.156954000

Si	4.455038000	4.208928000	0.647803000
C	2.565063000	4.104094000	0.892948000
H	2.144888000	5.108985000	0.742107000
H	2.362878000	3.856438000	1.944154000
C	4.850717000	4.341573000	−1.196798000
H	4.540635000	3.447945000	−1.753449000
H	5.929312000	4.461708000	−1.354879000
H	4.357402000	5.208394000	−1.653607000
C	5.073042000	5.742846000	1.551318000
H	4.635432000	6.659361000	1.137955000
H	6.163143000	5.834042000	1.472966000
H	4.823071000	5.707850000	2.618528000
C	5.271924000	2.668865000	1.374288000
H	5.070088000	2.587883000	2.449587000
H	6.361132000	2.710297000	1.250885000
H	4.926930000	1.741200000	0.901736000
C	3.594884000	−1.849779000	0.100061000
C	4.319428000	−0.879593000	−0.606043000
H	3.797453000	−0.051826000	−1.081343000
C	5.705854000	−0.969098000	−0.709627000
H	6.258289000	−0.212025000	−1.259641000
C	6.383383000	−2.022329000	−0.094786000
H	7.465380000	−2.088790000	−0.167573000
C	5.670892000	−2.984524000	0.619228000
H	6.195688000	−3.803904000	1.102844000
C	4.282156000	−2.902437000	0.717505000
H	3.735405000	−3.656222000	1.276604000
C	1.260117000	−2.761635000	1.600141000
C	1.340880000	−2.223248000	2.891924000
H	1.678185000	−1.200087000	3.037378000
C	0.998133000	−2.999725000	3.996516000
H	1.069757000	−2.578098000	4.995567000
C	0.564254000	−4.314048000	3.819369000
H	0.294294000	−4.918204000	4.681200000
C	0.479990000	−4.852332000	2.536110000

H	0.145685000	−5.876655000	2.395291000
C	0.827587000	−4.081237000	1.427267000
H	0.765021000	−4.507471000	0.430035000
C	1.152132000	−2.549822000	−1.339662000
C	−0.196182000	−2.293387000	−1.694718000
C	−0.661874000	−2.809579000	−2.921377000
H	−1.691982000	−2.617680000	−3.210458000
C	0.164288000	−3.568901000	−3.742559000
H	−0.225551000	−3.959050000	−4.679375000
C	1.487893000	−3.828843000	−3.373829000
H	2.129614000	−4.429578000	−4.011847000
C	1.977771000	−3.307972000	−2.179976000
H	3.008321000	−3.494513000	−1.890580000
C	−2.262144000	−2.030918000	−0.507298000
C	−2.555174000	−3.410757000	−0.576286000
H	−1.796071000	−4.099343000	−0.932085000
C	−3.789826000	−3.909702000	−0.182231000
H	−3.969210000	−4.980185000	−0.246826000
C	−4.788784000	−3.065262000	0.307673000
H	−5.750256000	−3.461048000	0.620003000
C	−4.518181000	−1.706170000	0.402608000
H	−5.269745000	−1.030226000	0.803470000
C	−3.281928000	−1.187303000	−0.003692000
C	−3.568817000	1.036687000	1.818116000
C	−4.508064000	2.054072000	2.016621000
H	−4.913500000	2.601886000	1.171574000
C	−4.950710000	2.358331000	3.305159000
H	−5.685732000	3.146320000	3.445764000
C	−4.464732000	1.650915000	4.401804000
H	−4.813942000	1.888336000	5.402851000
C	−3.534958000	0.627870000	4.208606000
H	−3.161754000	0.061741000	5.057913000
C	−3.085936000	0.323250000	2.926837000
H	−2.376986000	−0.489601000	2.786861000
C	−3.943231000	1.478923000	−1.070548000

C	−4.792464000	0.801063000	−1.952785000
H	−4.877853000	−0.280208000	−1.899569000
C	−5.537541000	1.511701000	−2.894719000
H	−6.196638000	0.975049000	−3.571883000
C	−5.446773000	2.900695000	−2.963207000
H	−6.033823000	3.450494000	−3.693611000
C	−4.600082000	3.584608000	−2.089311000
H	−4.527005000	4.668052000	−2.134974000
C	−3.844394000	2.877912000	−1.155566000
H	−3.181435000	3.419007000	−0.483005000
C	0.323495000	1.265212000	1.964269000
C	−0.234752000	2.427548000	1.384951000
H	−1.261598000	2.668280000	1.660915000
C	0.301564000	3.123120000	0.308862000
C	1.782214000	3.124066000	−0.011841000
H	1.943470000	3.409395000	−1.059455000
H	2.230440000	2.115812000	0.102484000
Y	−0.020283000	0.515035000	−0.400174000
H	−0.261278000	3.974673000	−0.068208000
H	1.410275000	1.169455000	2.016681000
H	−0.202669000	0.803069000	2.794662000
C	−0.615053000	2.428659000	−2.649702000
C	−0.043546000	1.267405000	−3.054154000
H	−0.014498000	3.311430000	−2.450439000
H	−1.693528000	2.547751000	−2.610007000
H	−0.677460000	0.426459000	−3.345625000
C	1.399674000	1.082018000	−3.179916000
C	1.973131000	−0.075879000	−3.556450000
H	2.023699000	1.953866000	−2.981310000
H	3.049938000	−0.162885000	−3.664323000
H	1.382961000	−0.957761000	−3.793236000

104

theta1_TS2_trans_1,4 E = −1968.280142 a.u. Imaginary frequency=210i

N	1.847680000	1.518197000	−0.113007000
P	−1.044541000	2.246284000	0.133485000

P	2.789857000	−1.284066000	0.145725000
Si	−6.586038000	−2.921844000	0.099766000
C	−4.886624000	−3.604136000	0.626001000
H	−5.002859000	−4.675070000	0.849177000
H	−4.603350000	−3.133963000	1.579101000
C	−7.158163000	−3.771501000	−1.486698000
H	−6.474516000	−3.587517000	−2.324382000
H	−8.147361000	−3.405978000	−1.788107000
H	−7.237555000	−4.857384000	−1.354837000
C	−7.810924000	−3.258764000	1.493823000
H	−7.904997000	−4.332866000	1.693797000
H	−8.810204000	−2.883312000	1.242489000
H	−7.502148000	−2.773645000	2.427735000
C	−6.451858000	−1.057296000	−0.193016000
H	−6.111189000	−0.532888000	0.708516000
H	−7.425896000	−0.634835000	−0.468160000
H	−5.753786000	−0.817229000	−1.004796000
C	−2.658225000	2.976913000	−0.363333000
C	−3.252659000	2.542383000	−1.555954000
H	−2.763265000	1.782829000	−2.161743000
C	−4.467141000	3.079863000	−1.975248000
H	−4.919563000	2.738733000	−2.902475000
C	−5.105050000	4.047069000	−1.198254000
H	−6.056185000	4.461913000	−1.520194000
C	−4.524726000	4.476178000	−0.005361000
H	−5.021546000	5.226604000	0.603436000
C	−3.305115000	3.946048000	0.413912000
H	−2.861264000	4.284003000	1.346054000
C	−0.913431000	2.513058000	1.949047000
C	−1.520951000	1.576197000	2.797280000
H	−2.055450000	0.724740000	2.379268000
C	−1.457981000	1.738070000	4.179563000
H	−1.935759000	1.011642000	4.831381000
C	−0.780940000	2.830195000	4.723347000
H	−0.728171000	2.954429000	5.801427000

C	−0.172660000	3.762352000	3.883355000
H	0.352556000	4.614819000	4.305518000
C	−0.236922000	3.607718000	2.498946000
H	0.236878000	4.337472000	1.848224000
C	0.248298000	3.298746000	−0.655743000
C	1.568950000	2.780133000	−0.679884000
C	2.557394000	3.532613000	−1.344011000
H	3.572764000	3.146694000	−1.373290000
C	2.251665000	4.741689000	−1.955518000
H	3.036018000	5.297266000	−2.463126000
C	0.945371000	5.238921000	−1.933699000
H	0.707604000	6.184619000	−2.411941000
C	−0.050869000	4.511375000	−1.292219000
H	−1.069566000	4.888892000	−1.274564000
C	2.942712000	1.413152000	0.753211000
C	3.492142000	2.518996000	1.434332000
H	3.068802000	3.505293000	1.274511000
C	4.553663000	2.367099000	2.318192000
H	4.943195000	3.244427000	2.828775000
C	5.112445000	1.112135000	2.567905000
H	5.939236000	0.998975000	3.262337000
C	4.582392000	0.005097000	1.915335000
H	4.993139000	−0.983514000	2.104534000
C	3.519351000	0.144939000	1.016039000
C	2.969095000	−2.710615000	1.300500000
C	3.953794000	−3.692809000	1.144328000
H	4.650395000	−3.641788000	0.312931000
C	4.047750000	−4.741118000	2.060371000
H	4.816619000	−5.498082000	1.931357000
C	3.166903000	−4.815141000	3.137380000
H	3.243452000	−5.632797000	3.848699000
C	2.188003000	−3.834272000	3.303075000
H	1.502455000	−3.882731000	4.144752000
C	2.086327000	−2.789994000	2.387881000
H	1.328420000	−2.023129000	2.536546000

C	3.901926000	−1.649547000	−1.282892000
C	4.968437000	−0.808782000	−1.622043000
H	5.201320000	0.049687000	−0.998465000
C	5.741304000	−1.081310000	−2.751711000
H	6.570756000	−0.426281000	−3.004496000
C	5.460641000	−2.191413000	−3.546397000
H	6.067934000	−2.402097000	−4.422297000
C	4.401407000	−3.036646000	−3.211099000
H	4.183660000	−3.908944000	−3.821522000
C	3.620897000	−2.763499000	−2.090447000
H	2.804732000	−3.434606000	−1.830198000
C	−0.943031000	−1.965473000	−0.016051000
C	−1.274918000	−3.359790000	0.306828000
H	−0.449216000	−3.941243000	0.717193000
C	−2.448343000	−3.990527000	0.109862000
C	−3.742145000	−3.419270000	−0.390791000
H	−4.013703000	−3.925005000	−1.329558000
H	−3.632511000	−2.355088000	−0.638147000
Y	0.354790000	−0.042405000	−0.843894000
H	−2.492569000	−5.045169000	0.384541000
H	−1.835265000	−1.324255000	−0.127795000
H	−0.316487000	−1.569976000	0.816285000
C	−0.350577000	−2.356656000	−2.118744000
C	0.274986000	−1.361046000	−2.895861000
H	−1.418022000	−2.527986000	−2.239568000
H	0.203751000	−3.252865000	−1.860295000
H	1.353787000	−1.402543000	−3.066106000
C	−0.456114000	−0.203974000	−3.349082000
C	0.072244000	1.045866000	−3.501919000
H	−1.543339000	−0.308500000	−3.415446000
H	−0.540227000	1.890198000	−3.800261000
H	1.150364000	1.217648000	−3.486003000

104

theta1_P2_trans_1,4 E = −1968.321992 a.u.

N	2.222231000	1.424667000	−0.407954000

P	−0.581630000	2.331034000	0.107945000
P	2.735691000	−1.413813000	0.206763000
Si	−7.327965000	−1.786730000	0.208395000
C	−5.817906000	−2.682518000	0.944310000
H	−6.187547000	−3.468050000	1.620415000
H	−5.265823000	−1.979632000	1.585509000
C	−8.432124000	−3.024409000	−0.693777000
H	−7.913363000	−3.505140000	−1.531810000
H	−9.322403000	−2.530735000	−1.102024000
H	−8.777226000	−3.816606000	−0.018438000
C	−8.287525000	−0.974541000	1.616226000
H	−8.627195000	−1.715065000	2.350361000
H	−9.177102000	−0.454794000	1.240206000
H	−7.674937000	−0.236984000	2.148885000
C	−6.745960000	−0.457465000	−1.008023000
H	−6.066893000	0.257231000	−0.526158000
H	−7.597879000	0.110196000	−1.401939000
H	−6.223180000	−0.896474000	−1.866884000
C	−2.208103000	3.099570000	−0.264920000
C	−3.007369000	2.527040000	−1.263889000
H	−2.667882000	1.640534000	−1.796583000
C	−4.240880000	3.088983000	−1.584848000
H	−4.854527000	2.639885000	−2.360617000
C	−4.690330000	4.217961000	−0.899355000
H	−5.656078000	4.651721000	−1.143357000
C	−3.904366000	4.785413000	0.103123000
H	−4.255389000	5.661881000	0.640758000
C	−2.665500000	4.231610000	0.421825000
H	−2.060261000	4.676439000	1.206691000
C	−0.142483000	2.843775000	1.814951000
C	−0.642788000	2.081603000	2.879671000
H	−1.267748000	1.211960000	2.685437000
C	−0.354078000	2.443884000	4.193346000
H	−0.747857000	1.853101000	5.015805000
C	0.440426000	3.561647000	4.450051000

H	0.668601000	3.841281000	5.474800000
C	0.940971000	4.320476000	3.392702000
H	1.557151000	5.192980000	3.591952000
C	0.651916000	3.966086000	2.075597000
H	1.042313000	4.561321000	1.254796000
C	0.625904000	3.154970000	−1.031577000
C	1.937433000	2.606722000	−1.113367000
C	2.858692000	3.214391000	−1.991998000
H	3.860559000	2.800159000	−2.064426000
C	2.495052000	4.310500000	−2.760163000
H	3.223614000	4.759703000	−3.429990000
C	1.195724000	4.827590000	−2.694440000
H	0.914347000	5.682423000	−3.302349000
C	0.267215000	4.244654000	−1.842636000
H	−0.740061000	4.646675000	−1.783330000
C	3.381618000	1.285404000	0.355483000
C	4.166024000	2.374384000	0.780117000
H	3.886010000	3.381061000	0.485369000
C	5.278591000	2.178633000	1.590762000
H	5.858688000	3.041561000	1.908132000
C	5.648906000	0.899899000	2.010761000
H	6.515500000	0.754754000	2.648413000
C	4.883175000	−0.188488000	1.604781000
H	5.150528000	−1.189492000	1.933727000
C	3.767640000	−0.009991000	0.781513000
C	2.631687000	−2.587703000	1.621303000
C	3.554454000	−3.624854000	1.806729000
H	4.351392000	−3.783392000	1.085852000
C	3.449193000	−4.463169000	2.915875000
H	4.168585000	−5.266022000	3.052241000
C	2.428745000	−4.272558000	3.846420000
H	2.349184000	−4.928706000	4.708701000
C	1.508867000	−3.238835000	3.669126000
H	0.712129000	−3.086591000	4.392140000
C	1.607091000	−2.402442000	2.559725000

H	0.889199000	−1.594239000	2.432934000
C	3.739821000	−2.270344000	−1.085215000
C	4.828528000	−1.636101000	−1.696507000
H	5.152868000	−0.659394000	−1.349053000
C	5.510577000	−2.265626000	−2.738214000
H	6.358699000	−1.768617000	−3.201442000
C	5.116454000	−3.528950000	−3.175177000
H	5.653458000	−4.018316000	−3.982942000
C	4.034669000	−4.168159000	−2.567427000
H	3.728749000	−5.157297000	−2.897265000
C	3.343700000	−3.540841000	−1.533602000
H	2.509355000	−4.052685000	−1.059141000
C	−1.704325000	−2.792477000	−0.269938000
C	−2.418442000	−3.862278000	0.505006000
H	−1.763672000	−4.546324000	1.047170000
C	−3.738729000	−4.077692000	0.570934000
C	−4.848522000	−3.304254000	−0.080648000
H	−5.405633000	−3.993330000	−0.732925000
H	−4.449553000	−2.522456000	−0.738239000
Y	0.600948000	0.061344000	−1.085929000
H	−4.074315000	−4.918569000	1.180772000
H	−2.408866000	−2.022957000	−0.611827000
H	−0.987311000	−2.291272000	0.408728000
C	−0.913964000	−3.335634000	−1.478823000
C	−0.101578000	−2.277112000	−2.194478000
H	−1.620618000	−3.819084000	−2.167170000
H	−0.244433000	−4.131373000	−1.130833000
H	0.959465000	−2.493408000	−2.359381000
C	−0.660971000	−1.257066000	−2.944072000
C	0.057797000	−0.142191000	−3.460243000
H	−1.752955000	−1.168859000	−2.928450000
H	−0.470258000	0.586961000	−4.070509000
H	1.089007000	−0.322121000	−3.795963000

97

P1_trans_1,4_isoprene E = −1851.721475 a.u.

N	0.291849000	−1.825031000	−0.435253000
P	2.694031000	−0.104714000	0.148901000
P	−2.393250000	−0.663717000	0.200968000
Si	−0.793580000	4.675742000	0.480509000
C	−0.634212000	2.975068000	−0.417369000
H	−1.166542000	2.270726000	0.253961000
H	0.455984000	2.779059000	−0.379965000
C	−2.619961000	5.060513000	0.717181000
H	−3.146591000	5.182874000	−0.236378000
H	−2.739960000	5.998626000	1.272643000
H	−3.128864000	4.274626000	1.288136000
C	0.069999000	4.483292000	2.144232000
H	−0.394455000	3.696565000	2.751252000
H	0.014097000	5.414354000	2.721044000
H	1.133335000	4.240868000	2.024172000
C	0.054556000	5.988571000	−0.567138000
H	1.110626000	5.750533000	−0.743584000
H	0.020235000	6.960292000	−0.059681000
H	−0.430192000	6.114605000	−1.542121000
C	4.309318000	0.723833000	−0.149975000
C	4.395314000	1.676237000	−1.175521000
H	3.521256000	1.905721000	−1.780456000
C	5.601566000	2.323949000	−1.431962000
H	5.661383000	3.057494000	−2.231470000
C	6.726426000	2.036339000	−0.658325000
H	7.665242000	2.547235000	−0.853584000
C	6.644211000	1.095967000	0.367776000
H	7.518196000	0.871336000	0.973045000
C	5.441647000	0.438578000	0.623550000
H	5.386072000	−0.290676000	1.426662000
C	2.800964000	−0.796656000	1.851107000
C	2.496776000	0.051167000	2.925953000
H	2.212938000	1.086091000	2.742815000
C	2.577436000	−0.419507000	4.234820000
H	2.349086000	0.244905000	5.063900000

C	2.952085000	−1.741208000	4.478378000
H	3.011396000	−2.109648000	5.498775000
C	3.252998000	−2.588244000	3.412452000
H	3.549269000	−3.616556000	3.601018000
C	3.180027000	−2.120227000	2.100893000
H	3.419820000	−2.781759000	1.273264000
C	2.657029000	−1.559550000	−0.997364000
C	1.434671000	−2.274167000	−1.125691000
C	1.390355000	−3.355731000	−2.029104000
H	0.458994000	−3.904894000	−2.138365000
C	2.506090000	−3.714029000	−2.772853000
H	2.443493000	−4.553063000	−3.461009000
C	3.699706000	−2.994443000	−2.654617000
H	4.570829000	−3.274546000	−3.239673000
C	3.766334000	−1.918130000	−1.778654000
H	4.691349000	−1.357121000	−1.681676000
C	−0.501017000	−2.715663000	0.291039000
C	−0.049843000	−3.987117000	0.699083000
H	0.946378000	−4.312818000	0.416932000
C	−0.849301000	−4.819671000	1.472457000
H	−0.463584000	−5.790953000	1.772271000
C	−2.124301000	−4.421666000	1.880682000
H	−2.741694000	−5.073490000	2.491096000
C	−2.585590000	−3.167895000	1.497166000
H	−3.569961000	−2.832752000	1.815439000
C	−1.799884000	−2.323224000	0.703900000
C	−3.106706000	0.055962000	1.747674000
C	−2.203800000	0.420852000	2.758794000
H	−1.136155000	0.245118000	2.631138000
C	−2.664799000	0.967457000	3.953480000
H	−1.957587000	1.233343000	4.734719000
C	−4.032671000	1.166475000	4.148586000
H	−4.393332000	1.595398000	5.079361000
C	−4.933722000	0.804790000	3.149694000
H	−6.000553000	0.946344000	3.300609000

C	−4.476625000	0.248983000	1.953529000
H	−5.191519000	−0.038972000	1.188619000
C	−3.816875000	−0.936975000	−0.939172000
C	−4.549344000	0.173253000	−1.389352000
H	−4.310538000	1.169986000	−1.024470000
C	−5.597142000	0.006785000	−2.291447000
H	−6.166768000	0.871144000	−2.622333000
C	−5.911582000	−1.265985000	−2.770956000
H	−6.726157000	−1.394747000	−3.478283000
C	−5.179287000	−2.369534000	−2.338609000
H	−5.421314000	−3.362912000	−2.706664000
C	−4.137440000	−2.210120000	−1.423818000
H	−3.580457000	−3.077959000	−1.083697000
C	−1.213245000	2.953951000	−1.860818000
C	−1.395674000	1.624669000	−2.596239000
H	−0.588733000	3.620817000	−2.472136000
H	−2.202677000	3.422706000	−1.814638000
C	−0.395016000	1.028205000	−3.365822000
C	0.977156000	1.253735000	−3.086914000
H	1.727822000	0.771017000	−3.708718000
H	1.269920000	2.243907000	−2.721199000
Y	0.090474000	0.336736000	−0.934472000
H	−2.422945000	1.336644000	−2.805868000
C	−0.766481000	−0.078117000	−4.328427000
H	−0.755543000	0.324328000	−5.349304000
H	−0.046846000	−0.904706000	−4.304994000
H	−1.768653000	−0.475093000	−4.138300000

110

theta3_C2_trans_1,4_isoprene E = −2046.896924 a.u.

N	−0.662132000	−1.622648000	0.789569000
P	−2.715206000	0.556429000	0.275195000
P	2.179014000	−1.260824000	0.157973000
Si	3.330448000	4.558116000	−0.447869000
C	1.752037000	3.876354000	−1.269518000
H	1.930399000	3.829234000	−2.353771000

H	0.955467000	4.622977000	−1.133673000
C	4.774727000	3.368662000	−0.716206000
H	4.613841000	2.386278000	−0.256243000
H	5.697016000	3.778931000	−0.286952000
H	4.958081000	3.206838000	−1.785703000
C	3.730275000	6.215019000	−1.256435000
H	3.917236000	6.102599000	−2.331167000
H	4.627268000	6.663542000	−0.812471000
H	2.910017000	6.932763000	−1.135699000
C	3.032195000	4.815550000	1.401734000
H	2.241895000	5.556410000	1.574322000
H	3.938665000	5.185264000	1.896315000
H	2.733738000	3.890738000	1.910019000
C	−2.977584000	2.317046000	0.785586000
C	−1.913141000	2.966859000	1.428632000
H	−0.989236000	2.430050000	1.631303000
C	−2.039490000	4.289647000	1.841913000
H	−1.210647000	4.777162000	2.348172000
C	−3.228251000	4.984302000	1.611924000
H	−3.327146000	6.017431000	1.933379000
C	−4.290025000	4.343975000	0.978224000
H	−5.223569000	4.873524000	0.807828000
C	−4.169850000	3.014833000	0.568069000
H	−5.015194000	2.525833000	0.094455000
C	−4.371537000	−0.012400000	−0.322375000
C	−4.881748000	0.515492000	−1.519420000
H	−4.322016000	1.273063000	−2.064996000
C	−6.114199000	0.090780000	−2.011708000
H	−6.505053000	0.518130000	−2.931452000
C	−6.844240000	−0.879890000	−1.323197000
H	−7.804208000	−1.213161000	−1.707724000
C	−6.340032000	−1.414245000	−0.138949000
H	−6.906875000	−2.165314000	0.404799000
C	−5.111114000	−0.982292000	0.363019000
H	−4.731330000	−1.396154000	1.292286000

C	−2.425690000	−0.301742000	1.865349000
C	−1.427778000	−1.308520000	1.919355000
C	−1.213749000	−1.923153000	3.173571000
H	−0.439421000	−2.676952000	3.267131000
C	−1.952086000	−1.568710000	4.294073000
H	−1.746482000	−2.065182000	5.239289000
C	−2.933248000	−0.576849000	4.226660000
H	−3.506141000	−0.297439000	5.105536000
C	−3.151370000	0.056327000	3.010184000
H	−3.896198000	0.845127000	2.937994000
C	0.013580000	−2.859354000	0.779254000
C	−0.634296000	−4.083146000	1.039278000
H	−1.673503000	−4.070238000	1.357443000
C	0.033787000	−5.294632000	0.891642000
H	−0.495339000	−6.223112000	1.091276000
C	1.369072000	−5.327335000	0.481378000
H	1.885899000	−6.274874000	0.360751000
C	2.038579000	−4.128781000	0.247141000
H	3.084679000	−4.140632000	−0.047061000
C	1.380825000	−2.903741000	0.409443000
C	3.829623000	−1.631156000	−0.582868000
C	4.930173000	−2.000858000	0.202766000
H	4.832721000	−2.063635000	1.282913000
C	6.157235000	−2.279289000	−0.396803000
H	7.004702000	−2.563443000	0.221138000
C	6.300106000	−2.190186000	−1.781427000
H	7.259441000	−2.404309000	−2.244388000
C	5.212748000	−1.815351000	−2.569372000
H	5.322081000	−1.732273000	−3.647465000
C	3.985924000	−1.531550000	−1.971888000
H	3.147688000	−1.221154000	−2.590111000
C	2.576755000	−0.710693000	1.878159000
C	2.562694000	−1.600042000	2.959440000
H	2.286959000	−2.639123000	2.805305000
C	2.914043000	−1.159276000	4.234927000

H	2.903514000	−1.858374000	5.066934000
C	3.282030000	0.169043000	4.441874000
H	3.556895000	0.509895000	5.436270000
C	3.301986000	1.058694000	3.368016000
H	3.597058000	2.092942000	3.522169000
C	2.948084000	0.623040000	2.092638000
H	2.973015000	1.322527000	1.261961000
C	0.442664000	−2.431104000	−3.382268000
C	−0.502580000	−2.851079000	−2.508062000
H	−0.220314000	−3.567113000	−1.738147000
C	−1.917270000	−2.440476000	−2.530075000
C	−2.297623000	−1.274438000	−3.108606000
H	−3.336111000	−0.960580000	−3.106100000
H	−1.602289000	−0.665552000	−3.684947000
Y	−0.337558000	−0.384340000	−1.145778000
H	1.468339000	−2.775340000	−3.288994000
H	0.202066000	−1.846018000	−4.262666000
C	1.227504000	2.501333000	−0.798685000
C	−0.051846000	2.130497000	−1.543103000
H	2.015716000	1.750266000	−0.945886000
H	1.035146000	2.534465000	0.281853000
H	−0.877499000	2.807588000	−1.319357000
C	−0.026044000	1.631429000	−2.866861000
C	0.912416000	0.686210000	−3.295493000
H	0.907611000	0.359787000	−4.331591000
H	1.883901000	0.622098000	−2.804367000
C	−1.169885000	2.003598000	−3.784987000
H	−0.984005000	3.012611000	−4.174249000
H	−1.263502000	1.335334000	−4.647102000
H	−2.130261000	2.043465000	−3.257862000
C	−2.898130000	−3.355573000	−1.855978000
H	−2.977427000	−4.287203000	−2.431658000
H	−2.557135000	−3.629632000	−0.852563000
H	−3.891385000	−2.906918000	−1.785258000

110

theta3_TS2_trans_1,4_isoprene E = −2046.887531 a.u. Imaginary frequency=154i

N	−0.236484000	−1.340820000	1.032706000
P	−2.852619000	−0.055299000	0.156456000
P	2.531037000	−0.917888000	0.044521000
Si	1.703981000	5.244654000	−0.224238000
C	0.441394000	4.113903000	−1.102243000
H	0.533397000	4.296087000	−2.182861000
H	−0.562800000	4.470619000	−0.830260000
C	3.444036000	4.879448000	−0.864612000
H	3.757181000	3.846504000	−0.673074000
H	4.182128000	5.537035000	−0.389454000
H	3.508472000	5.047891000	−1.946659000
C	1.241244000	7.019701000	−0.663806000
H	1.271857000	7.188483000	−1.746911000
H	1.935866000	7.731451000	−0.201481000
H	0.232525000	7.273579000	−0.316459000
C	1.612033000	5.003819000	1.645140000
H	0.604031000	5.213649000	2.023851000
H	2.298005000	5.690068000	2.156974000
H	1.879230000	3.985595000	1.950652000
C	−3.681399000	1.609512000	0.181739000
C	−2.989903000	2.690225000	0.752676000
H	−2.024777000	2.529518000	1.226275000
C	−3.546513000	3.966882000	0.756046000
H	−3.005197000	4.787667000	1.219337000
C	−4.798477000	4.188438000	0.181305000
H	−5.234213000	5.183674000	0.186273000
C	−5.492025000	3.121859000	−0.384970000
H	−6.475319000	3.279571000	−0.820055000
C	−4.940386000	1.839659000	−0.384206000
H	−5.513169000	1.019228000	−0.804149000
C	−4.258121000	−1.254363000	0.000714000
C	−4.912878000	−1.382419000	−1.234962000
H	−4.601203000	−0.778704000	−2.085333000
C	−5.968975000	−2.278517000	−1.389343000

H	−6.473958000	−2.357617000	−2.348465000
C	−6.370950000	−3.074770000	−0.316480000
H	−7.190850000	−3.777406000	−0.436838000
C	−5.715923000	−2.965092000	0.908411000
H	−6.024232000	−3.582054000	1.748295000
C	−4.667230000	−2.058767000	1.070089000
H	−4.172560000	−1.975399000	2.032832000
C	−2.254474000	−0.231439000	1.881759000
C	−1.021298000	−0.891803000	2.104066000
C	−0.601815000	−1.035810000	3.444851000
H	0.344397000	−1.525343000	3.648471000
C	−1.361454000	−0.554190000	4.500911000
H	−0.996157000	−0.680059000	5.517161000
C	−2.575496000	0.098126000	4.270488000
H	−3.170909000	0.475309000	5.096443000
C	−3.007516000	0.257286000	2.960487000
H	−3.949442000	0.762189000	2.762835000
C	0.573114000	−2.472580000	1.250691000
C	0.080374000	−3.631980000	1.882911000
H	−0.935376000	−3.624923000	2.268111000
C	0.861634000	−4.775127000	1.998019000
H	0.450074000	−5.655584000	2.485153000
C	2.156520000	−4.807886000	1.473238000
H	2.761580000	−5.706502000	1.549941000
C	2.669992000	−3.668861000	0.860427000
H	3.682156000	−3.680810000	0.466560000
C	1.905157000	−2.498650000	0.766502000
C	4.140271000	−1.347460000	−0.759293000
C	5.276737000	−1.664069000	0.000354000
H	5.225349000	−1.653980000	1.085685000
C	6.478395000	−1.977018000	−0.630394000
H	7.352009000	−2.221882000	−0.032359000
C	6.564486000	−1.967898000	−2.023312000
H	7.504832000	−2.207835000	−2.511853000
C	5.446381000	−1.636377000	−2.785737000

H	5.512654000	−1.611474000	−3.870232000
C	4.242596000	−1.323955000	−2.154702000
H	3.380493000	−1.042028000	−2.751055000
C	3.100909000	0.044818000	1.519599000
C	3.092098000	−0.480058000	2.815807000
H	2.713347000	−1.482104000	2.993276000
C	3.593856000	0.270380000	3.879975000
H	3.586016000	−0.148172000	4.882920000
C	4.115247000	1.543096000	3.657851000
H	4.511884000	2.122489000	4.486984000
C	4.135426000	2.068203000	2.365072000
H	4.552182000	3.054739000	2.182309000
C	3.625075000	1.325810000	1.302965000
H	3.660020000	1.736633000	0.296691000
C	0.767244000	−1.592763000	−3.647893000
C	0.558094000	−2.522913000	−2.629316000
H	1.419192000	−3.010833000	−2.176466000
C	−0.762903000	−2.985396000	−2.249888000
C	−1.877385000	−2.232467000	−2.509228000
H	−2.861606000	−2.570477000	−2.208303000
H	−1.856386000	−1.386806000	−3.199977000
Y	−0.205801000	−0.581053000	−1.125322000
H	1.774874000	−1.445906000	−4.017936000
H	−0.016254000	−1.422830000	−4.380442000
C	0.500240000	2.586939000	−0.858303000
C	−0.595820000	1.934702000	−1.684245000
H	1.494022000	2.208071000	−1.127320000
H	0.370450000	2.386326000	0.214935000
H	−1.589439000	2.323391000	−1.466470000
C	−0.424177000	1.386763000	−2.955112000
C	0.774680000	0.750795000	−3.383525000
H	0.916791000	0.642372000	−4.453703000
H	1.702197000	0.959106000	−2.847535000
C	−1.610391000	1.360070000	−3.895501000
H	−1.582572000	2.260951000	−4.522209000

H	−1.599153000	0.500907000	−4.575185000
H	−2.562674000	1.375558000	−3.355353000
C	−0.863534000	−4.284938000	−1.499707000
H	−0.774839000	−5.110680000	−2.217858000
H	−0.059866000	−4.403590000	−0.767407000
H	−1.823757000	−4.383409000	−0.986852000

110

theta3_P2_trans_1,4_isoprene E = −2046.925077 a.u.

N	−1.100074000	−1.715696000	0.417128000
P	−2.910676000	0.662458000	0.309895000
P	1.805553000	−1.471650000	0.198487000
Si	4.633095000	3.816629000	0.368168000
C	2.801961000	3.813836000	−0.178598000
H	2.720531000	4.373427000	−1.121355000
H	2.227729000	4.392998000	0.559951000
C	5.674802000	2.875233000	−0.893193000
H	5.393943000	1.816911000	−0.955890000
H	6.736490000	2.912637000	−0.620403000
H	5.581597000	3.309815000	−1.896150000
C	5.190639000	5.614917000	0.463137000
H	5.104986000	6.114761000	−0.509147000
H	6.239104000	5.684914000	0.777066000
H	4.593448000	6.186015000	1.184106000
C	4.782298000	3.002172000	2.066027000
H	4.153287000	3.506747000	2.809908000
H	5.816674000	3.053526000	2.426910000
H	4.500799000	1.942242000	2.046605000
C	−3.094915000	2.281132000	1.186116000
C	−2.096880000	2.639329000	2.106953000
H	−1.292530000	1.943234000	2.338873000
C	−2.150033000	3.866632000	2.762204000
H	−1.380170000	4.126709000	3.484025000
C	−3.194926000	4.755042000	2.501178000
H	−3.236870000	5.712416000	3.013060000
C	−4.189414000	4.403853000	1.591338000

H	−5.013192000	5.084565000	1.393990000
C	−4.143580000	3.172168000	0.935224000
H	−4.934341000	2.905519000	0.240218000
C	−4.499901000	0.384689000	−0.589426000
C	−4.763271000	1.132471000	−1.747258000
H	−4.053717000	1.884215000	−2.085046000
C	−5.938670000	0.929887000	−2.466781000
H	−6.136237000	1.521911000	−3.356455000
C	−6.854762000	−0.035786000	−2.048512000
H	−7.768657000	−0.199089000	−2.612936000
C	−6.593077000	−0.790525000	−0.906589000
H	−7.303494000	−1.543705000	−0.576544000
C	−5.422968000	−0.582670000	−0.176454000
H	−5.230999000	−1.173488000	0.713854000
C	−2.859947000	−0.575643000	1.655283000
C	−1.971050000	−1.669606000	1.505026000
C	−1.972977000	−2.649999000	2.518725000
H	−1.294792000	−3.493500000	2.439243000
C	−2.804255000	−2.537678000	3.625229000
H	−2.771571000	−3.309207000	4.390568000
C	−3.661419000	−1.444617000	3.775835000
H	−4.303135000	−1.357274000	4.647250000
C	−3.677979000	−0.467317000	2.787562000
H	−4.337404000	0.391513000	2.887356000
C	−0.538025000	−2.939810000	0.015103000
C	−1.310740000	−4.092030000	−0.234434000
H	−2.381117000	−4.054760000	−0.051336000
C	−0.717294000	−5.253519000	−0.710274000
H	−1.331321000	−6.131747000	−0.892754000
C	0.657664000	−5.297862000	−0.971030000
H	1.114634000	−6.208054000	−1.348601000
C	1.438814000	−4.172952000	−0.732868000
H	2.509139000	−4.206157000	−0.917790000
C	0.858725000	−3.002069000	−0.224153000
C	3.524462000	−1.788111000	−0.392187000

C	4.470050000	−2.478432000	0.377990000
H	4.207956000	−2.844431000	1.366446000
C	5.755649000	−2.690649000	−0.117384000
H	6.482792000	−3.225249000	0.487839000
C	6.109478000	−2.218819000	−1.381220000
H	7.113181000	−2.385152000	−1.762652000
C	5.175439000	−1.526492000	−2.151446000
H	5.448459000	−1.151087000	−3.134098000
C	3.891389000	−1.306132000	−1.656064000
H	3.168483000	−0.755967000	−2.253263000
C	1.916515000	−1.545992000	2.042535000
C	1.843883000	−2.763150000	2.732409000
H	1.688938000	−3.689345000	2.185606000
C	1.970690000	−2.791690000	4.120492000
H	1.917631000	−3.741538000	4.645959000
C	2.165351000	−1.608714000	4.832585000
H	2.262378000	−1.633942000	5.914462000
C	2.234430000	−0.393226000	4.153163000
H	2.386968000	0.531867000	4.702465000
C	2.109298000	−0.362321000	2.765281000
H	2.170767000	0.590270000	2.246034000
C	0.356385000	1.169540000	−4.099614000
C	0.544107000	−0.250251000	−3.578735000
H	1.525608000	−0.670657000	−3.799188000
C	−0.502292000	−1.183387000	−3.502666000
C	−1.809742000	−0.834477000	−3.093551000
H	−2.581229000	−1.599704000	−3.060790000
H	−2.193280000	0.165958000	−3.316530000
Y	−0.479262000	−0.172157000	−1.094624000
H	0.846233000	1.216920000	−5.082878000
H	−0.703221000	1.363864000	−4.303587000
C	2.151370000	2.424947000	−0.361682000
C	0.700528000	2.582182000	−0.737487000
H	2.704811000	1.846817000	−1.108644000
H	2.228690000	1.865057000	0.577280000

H	0.051661000	2.914297000	0.079098000
C	0.165088000	2.613126000	−1.986560000
C	0.941660000	2.313253000	−3.248496000
H	0.943189000	3.230417000	−3.856601000
H	1.988708000	2.091891000	−3.021134000
C	−0.167897000	−2.651160000	−3.638485000
H	0.890825000	−2.855365000	−3.455506000
H	−0.768010000	−3.284751000	−2.978924000
H	−0.388871000	−2.950679000	−4.671695000
C	−1.233314000	3.144993000	−2.195797000
H	−1.857774000	2.489923000	−2.815122000
H	−1.750814000	3.339494000	−1.252084000
H	−1.169686000	4.097158000	−2.741388000

110

P1_thf_trans_1,4_isoprene E = −2084.07518 a.u.

N	−0.014479000	−0.171821000	1.374792000
P	2.713262000	−0.308393000	0.079813000
P	−2.758718000	−0.228744000	0.090978000
Si	0.240037000	4.529494000	−0.382205000
C	0.010059000	2.692358000	−0.919326000
H	−0.716799000	2.287777000	−0.191001000
H	0.994587000	2.220641000	−0.734571000
C	−1.454053000	5.358774000	−0.409978000
H	−1.892147000	5.393123000	−1.414297000
H	−1.369805000	6.395990000	−0.062831000
H	−2.162854000	4.849692000	0.254400000
C	0.945197000	4.562709000	1.359522000
H	0.301158000	4.020556000	2.061389000
H	1.031909000	5.595909000	1.717977000
H	1.943671000	4.113118000	1.401474000
C	1.407674000	5.375741000	−1.595292000
H	2.394018000	4.897239000	−1.597909000
H	1.553429000	6.425680000	−1.313132000
H	1.021867000	5.365084000	−2.621272000
C	4.039950000	−1.377914000	−0.630875000

C	3.669531000	−2.399281000	−1.514812000
H	2.621562000	−2.526206000	−1.772986000
C	4.631407000	−3.241394000	−2.068656000
H	4.334076000	−4.028140000	−2.757431000
C	5.978002000	−3.063906000	−1.751357000
H	6.730728000	−3.714285000	−2.188311000
C	6.356712000	−2.044028000	−0.879477000
H	7.405213000	−1.898864000	−0.633649000
C	5.395058000	−1.202655000	−0.320996000
H	5.702585000	−0.404846000	0.349132000
C	3.622921000	1.238382000	0.523744000
C	4.102817000	2.048091000	−0.517444000
H	3.939699000	1.760670000	−1.554388000
C	4.820768000	3.207298000	−0.232038000
H	5.202822000	3.819556000	−1.044640000
C	5.055142000	3.576208000	1.094129000
H	5.615983000	4.479972000	1.316025000
C	4.572070000	2.780591000	2.131582000
H	4.755997000	3.061630000	3.165140000
C	3.858348000	1.614311000	1.850304000
H	3.496985000	0.990168000	2.662637000
C	2.251742000	−1.090803000	1.675747000
C	0.923318000	−0.887497000	2.136473000
C	0.568536000	−1.510962000	3.353812000
H	−0.441659000	−1.383246000	3.731038000
C	1.471232000	−2.288237000	4.066745000
H	1.150679000	−2.755528000	4.994800000
C	2.772540000	−2.486964000	3.597896000
H	3.477210000	−3.099028000	4.152810000
C	3.148658000	−1.886635000	2.402109000
H	4.154758000	−2.036368000	2.018922000
C	−0.970154000	0.576354000	2.083080000
C	−0.632069000	1.303020000	3.245918000
H	0.388079000	1.252066000	3.614805000
C	−1.566459000	2.080987000	3.916331000

H	−1.261813000	2.627538000	4.805540000
C	−2.881294000	2.178399000	3.452535000
H	−3.611672000	2.787898000	3.976230000
C	−3.239713000	1.482194000	2.304764000
H	−4.256710000	1.550361000	1.927859000
C	−2.309676000	0.683739000	1.623221000
C	−4.272434000	0.625611000	−0.536844000
C	−5.556476000	0.083594000	−0.402749000
H	−5.699323000	−0.873131000	0.089921000
C	−6.664286000	0.771995000	−0.898597000
H	−7.655607000	0.340486000	−0.788950000
C	−6.504112000	2.006579000	−1.523896000
H	−7.369190000	2.539952000	−1.908141000
C	−5.228556000	2.557387000	−1.651979000
H	−5.096313000	3.522543000	−2.133640000
C	−4.119811000	1.869140000	−1.166720000
H	−3.131400000	2.307457000	−1.268853000
C	−3.385022000	−1.876790000	0.648368000
C	−3.511793000	−2.215724000	1.999864000
H	−3.248133000	−1.491551000	2.765576000
C	−3.992361000	−3.474192000	2.365367000
H	−4.091314000	−3.726771000	3.417742000
C	−4.360430000	−4.397443000	1.387860000
H	−4.745940000	−5.371664000	1.675887000
C	−4.241869000	−4.063917000	0.037075000
H	−4.542066000	−4.774668000	−0.728799000
C	−3.745828000	−2.814699000	−0.331323000
H	−3.666021000	−2.556964000	−1.386023000
C	1.211007000	0.154916000	−3.230090000
C	−0.145965000	0.283684000	−3.557021000
H	1.841774000	−0.552859000	−3.762183000
H	1.748696000	1.020188000	−2.833891000
C	−0.984435000	1.190106000	−2.880893000
C	−0.474181000	2.546029000	−2.383067000
H	−1.292262000	3.260924000	−2.534289000

H	0.341106000	2.884686000	−3.037929000
Y	−0.022788000	−0.188204000	−0.956399000
O	−0.026342000	−2.558345000	−1.079712000
C	−0.080913000	−3.382998000	−2.282155000
C	−0.010127000	−3.427285000	0.109653000
C	0.267486000	−4.788567000	−1.820502000
H	−1.097720000	−3.325630000	−2.685178000
H	0.620034000	−2.961912000	−3.005490000
C	−0.324932000	−4.819027000	−0.411208000
H	0.989681000	−3.360002000	0.546486000
H	−0.744997000	−3.037340000	0.814065000
H	1.354220000	−4.924484000	−1.785575000
H	−0.151856000	−5.550415000	−2.483637000
H	0.116317000	−5.594146000	0.221462000
H	−1.407375000	−4.978813000	−0.447965000
H	−2.035473000	1.170748000	−3.164031000
C	−0.783355000	−0.700020000	−4.512774000
H	−0.160892000	−1.581951000	−4.689709000
H	−1.778607000	−1.021405000	−4.183175000
H	−0.917948000	−0.201415000	−5.480875000

123

theta3_thf_C2_trans_1,4_isoprene E = −2279.226566 a.u.

N	−1.099355000	1.297764000	1.133856000
P	−2.979656000	−0.756480000	−0.030934000
P	1.822352000	1.864581000	0.419069000
Si	5.137200000	−3.874316000	−1.176980000
C	3.853311000	−2.759797000	−2.039666000
H	3.351176000	−3.378482000	−2.798318000
H	4.388588000	−1.979273000	−2.600589000
C	4.310688000	−5.499669000	−0.680602000
H	3.443574000	−5.338505000	−0.028745000
H	5.009591000	−6.152626000	−0.143908000
H	3.960659000	−6.048918000	−1.563117000
C	6.533337000	−4.221355000	−2.397393000
H	6.157510000	−4.696347000	−3.311659000

H	7.281366000	−4.894553000	−1.961334000
H	7.050519000	−3.299801000	−2.690464000
C	5.835383000	−3.022571000	0.360175000
H	6.262439000	−2.043118000	0.114340000
H	6.631165000	−3.627624000	0.811385000
H	5.066047000	−2.869591000	1.127355000
C	−4.130943000	−1.163516000	−1.417947000
C	−3.784250000	−0.757326000	−2.714051000
H	−2.848036000	−0.230161000	−2.882142000
C	−4.627708000	−1.022966000	−3.790548000
H	−4.347464000	−0.703232000	−4.790978000
C	−5.825603000	−1.707815000	−3.585724000
H	−6.481145000	−1.922111000	−4.425241000
C	−6.176308000	−2.119463000	−2.301217000
H	−7.108272000	−2.653157000	−2.135303000
C	−5.336206000	−1.849060000	−1.220859000
H	−5.621532000	−2.176585000	−0.225482000
C	−3.530546000	−1.895582000	1.318380000
C	−3.290836000	−3.271457000	1.182590000
H	−2.782197000	−3.652043000	0.300675000
C	−3.722663000	−4.160765000	2.164893000
H	−3.548129000	−5.226683000	2.041880000
C	−4.384604000	−3.685436000	3.298830000
H	−4.721527000	−4.379793000	4.063716000
C	−4.618283000	−2.318687000	3.442233000
H	−5.138549000	−1.943363000	4.319477000
C	−4.195857000	−1.425355000	2.456225000
H	−4.393344000	−0.363192000	2.567742000
C	−3.459203000	0.922884000	0.515165000
C	−2.430298000	1.738538000	1.057368000
C	−2.806353000	3.050865000	1.433162000
H	−2.051410000	3.715620000	1.841556000
C	−4.106764000	3.513509000	1.289640000
H	−4.340065000	4.533175000	1.587160000
C	−5.107130000	2.695215000	0.757480000

H	−6.123815000	3.058492000	0.642062000
C	−4.767912000	1.404986000	0.370926000
H	−5.526123000	0.753887000	−0.057092000
C	−0.346309000	1.819662000	2.198162000
C	−0.905836000	1.969989000	3.488799000
H	−1.946367000	1.696299000	3.637553000
C	−0.166641000	2.455580000	4.557879000
H	−0.639995000	2.549395000	5.532299000
C	1.175015000	2.809176000	4.393958000
H	1.756612000	3.191581000	5.227359000
C	1.758389000	2.652143000	3.143348000
H	2.805011000	2.907947000	3.005575000
C	1.023119000	2.165141000	2.051385000
C	3.629560000	1.820479000	0.829833000
C	4.471176000	2.926937000	0.654102000
H	4.083491000	3.850962000	0.237531000
C	5.815041000	2.857376000	1.021720000
H	6.454169000	3.724487000	0.877988000
C	6.332755000	1.690562000	1.579464000
H	7.378730000	1.640498000	1.869244000
C	5.499951000	0.588138000	1.769063000
H	5.892665000	−0.324594000	2.207871000
C	4.160865000	0.650347000	1.390519000
H	3.525687000	−0.215203000	1.553001000
C	1.653036000	3.453162000	−0.517464000
C	0.879679000	4.515769000	−0.039022000
H	0.375569000	4.427223000	0.918931000
C	0.775960000	5.698499000	−0.773599000
H	0.177822000	6.519661000	−0.387310000
C	1.453284000	5.836658000	−1.984400000
H	1.384284000	6.764424000	−2.545891000
C	2.233048000	4.782862000	−2.465626000
H	2.777361000	4.888760000	−3.400556000
C	2.325228000	3.595162000	−1.741990000
H	2.955134000	2.789066000	−2.114038000

C	1.063480000	−1.447291000	2.017078000
C	0.262024000	−2.318212000	2.677176000
C	0.267180000	−3.762487000	2.390902000
C	1.365988000	−4.482377000	2.132770000
H	1.311596000	−5.554131000	1.963820000
H	2.356430000	−4.033926000	2.122893000
Y	−0.150116000	−0.256582000	−0.417617000
H	−0.695080000	−4.266891000	2.474708000
O	−0.707292000	1.268834000	−2.185479000
C	0.015782000	1.420645000	−3.444005000
C	−1.636785000	2.402414000	−2.036014000
C	−0.878518000	2.295159000	−4.300805000
H	0.971353000	1.908655000	−3.231431000
H	0.191298000	0.425605000	−3.844676000
C	−1.435052000	3.275359000	−3.267844000
H	−2.643402000	1.982143000	−1.979162000
H	−1.401043000	2.905410000	−1.099351000
H	−1.680077000	1.700375000	−4.754092000
H	−0.321323000	2.786472000	−5.103608000
H	−2.371060000	3.745245000	−3.582746000
H	−0.706785000	4.065953000	−3.062314000
H	1.154144000	−0.420705000	2.368200000
H	1.787995000	−1.810866000	1.288598000
C	2.781618000	−2.090601000	−1.152134000
C	1.693704000	−1.465702000	−2.023785000
H	2.334316000	−2.845164000	−0.491086000
H	3.282070000	−1.355379000	−0.508823000
H	2.115482000	−0.937088000	−2.882281000
C	0.463514000	−2.106219000	−2.267847000
C	−0.331351000	−2.653641000	−1.241531000
H	−1.230528000	−3.200899000	−1.513259000
H	0.145015000	−3.008215000	−0.320598000
C	−0.146346000	−2.077109000	−3.654229000
H	−0.144507000	−3.105306000	−4.038455000
H	−1.189896000	−1.743110000	−3.663921000

H	0.439066000	−1.481032000	−4.359916000
C	−0.645928000	−1.876005000	3.787290000
H	−0.642923000	−0.790478000	3.910497000
H	−1.673383000	−2.213092000	3.611123000
H	−0.320149000	−2.336038000	4.729654000

123

theta3_thf_TS2_trans_1,4_isoprene E = −2279.197198 a.u. Imaginary frequency=202i

N	−1.366688000	1.302556000	0.914308000
P	−2.893156000	−1.176032000	0.041348000
P	1.434710000	2.283892000	0.168141000
Si	5.628731000	−4.267379000	−0.063379000
C	4.427197000	−3.603445000	−1.386517000
H	3.846298000	−4.451342000	−1.777690000
H	5.019993000	−3.233220000	−2.235905000
C	4.643174000	−5.028187000	1.358701000
H	4.035995000	−4.281933000	1.886592000
H	5.314726000	−5.477518000	2.100267000
H	3.972026000	−5.820402000	1.004583000
C	6.717461000	−5.579173000	−0.868202000
H	6.120848000	−6.406290000	−1.271350000
H	7.421869000	−6.004874000	−0.143477000
H	7.307157000	−5.162586000	−1.693633000
C	6.698892000	−2.855785000	0.596594000
H	7.247105000	−2.355250000	−0.211079000
H	7.441558000	−3.234285000	1.309345000
H	6.103424000	−2.099003000	1.122475000
C	−3.875320000	−2.025898000	−1.277697000
C	−3.343627000	−2.127916000	−2.569061000
H	−2.352030000	−1.738304000	−2.776598000
C	−4.066764000	−2.737126000	−3.593559000
H	−3.637202000	−2.810617000	−4.589216000
C	−5.331838000	−3.261536000	−3.335724000
H	−5.895744000	−3.741938000	−4.130546000
C	−5.867778000	−3.175843000	−2.050820000
H	−6.851649000	−3.587430000	−1.842158000

C	−5.146415000	−2.564934000	−1.026778000
H	−5.571704000	−2.515963000	−0.028502000
C	−3.510623000	−2.014257000	1.574732000
C	−3.414918000	−3.410736000	1.677875000
H	−3.050670000	−3.997396000	0.837063000
C	−3.815963000	−4.060395000	2.842877000
H	−3.747983000	−5.143199000	2.906727000
C	−4.309522000	−3.324243000	3.921257000
H	−4.622807000	−3.831916000	4.829328000
C	−4.408644000	−1.937542000	3.824445000
H	−4.805133000	−1.360069000	4.655490000
C	−4.012432000	−1.283093000	2.656927000
H	−4.108383000	−0.203800000	2.581206000
C	−3.577595000	0.523156000	0.146601000
C	−2.719319000	1.546361000	0.632338000
C	−3.289600000	2.837580000	0.748358000
H	−2.684267000	3.648503000	1.139718000
C	−4.601554000	3.100554000	0.380911000
H	−4.984622000	4.113290000	0.481456000
C	−5.421738000	2.088430000	−0.121475000
H	−6.446484000	2.292919000	−0.416309000
C	−4.897385000	0.806976000	−0.228652000
H	−5.522124000	0.005594000	−0.613116000
C	−0.774004000	2.122653000	1.897979000
C	−1.456805000	2.428348000	3.096439000
H	−2.464785000	2.045972000	3.229205000
C	−0.876276000	3.190305000	4.099085000
H	−1.439372000	3.400585000	5.004992000
C	0.431345000	3.660149000	3.959342000
H	0.899961000	4.244058000	4.746131000
C	1.128399000	3.371642000	2.791937000
H	2.147153000	3.729680000	2.684560000
C	0.541907000	2.635607000	1.752488000
C	3.230052000	2.602114000	0.483541000
C	3.722390000	3.887723000	0.762926000

H	3.042710000	4.734646000	0.800250000
C	5.084534000	4.092668000	0.968504000
H	5.450902000	5.091885000	1.188094000
C	5.976705000	3.022882000	0.883640000
H	7.039248000	3.186528000	1.041267000
C	5.503475000	1.748351000	0.579733000
H	6.195013000	0.914710000	0.492653000
C	4.138712000	1.542365000	0.376798000
H	3.779728000	0.550080000	0.119963000
C	1.005489000	3.741492000	−0.906465000
C	−0.203392000	4.426649000	−0.750677000
H	−0.881002000	4.148945000	0.048481000
C	−0.536249000	5.481152000	−1.601593000
H	−1.474931000	6.010140000	−1.458719000
C	0.335489000	5.864408000	−2.619679000
H	0.080336000	6.691954000	−3.276004000
C	1.545302000	5.188064000	−2.781901000
H	2.238576000	5.488750000	−3.563158000
C	1.878195000	4.132738000	−1.934581000
H	2.834103000	3.629875000	−2.061739000
C	1.902795000	−0.572451000	1.944444000
C	0.774430000	−0.887957000	2.659411000
C	−0.173963000	−1.850205000	2.161484000
C	0.196996000	−2.895510000	1.315207000
H	−0.530920000	−3.674865000	1.120940000
H	1.233735000	−3.215616000	1.306270000
Y	−0.001005000	−0.377429000	0.012090000
H	−1.183221000	−1.832086000	2.561437000
O	−0.428299000	0.632878000	−2.201191000
C	0.623707000	0.993714000	−3.140387000
C	−1.653183000	1.341850000	−2.583364000
C	−0.108975000	1.346685000	−4.419191000
H	1.158092000	1.860886000	−2.744408000
H	1.305898000	0.148021000	−3.211162000
C	−1.326292000	2.094636000	−3.872088000

H	−2.430017000	0.585705000	−2.721885000
H	−1.936891000	2.000633000	−1.762837000
H	−0.407260000	0.441333000	−4.959984000
H	0.503509000	1.959942000	−5.086356000
H	−2.174312000	2.100918000	−4.562691000
H	−1.060916000	3.132672000	−3.647662000
H	2.619416000	0.152144000	2.315261000
H	2.264994000	−1.223089000	1.143144000
C	3.455974000	−2.496605000	−0.922201000
C	2.526236000	−2.102565000	−2.034008000
H	2.885653000	−2.853052000	−0.056618000
H	4.047225000	−1.633881000	−0.582997000
H	3.030741000	−1.764443000	−2.941675000
C	1.187182000	−2.322577000	−2.111964000
C	0.352335000	−2.873144000	−1.008818000
H	−0.676486000	−3.059567000	−1.333334000
H	0.768268000	−3.817803000	−0.661346000
C	0.498902000	−2.198839000	−3.454237000
H	0.154601000	−3.192869000	−3.768092000
H	−0.383556000	−1.552445000	−3.418074000
H	1.171309000	−1.824293000	−4.233448000
C	0.459249000	−0.198225000	3.954898000
H	0.963481000	0.766018000	4.049403000
H	−0.615232000	−0.051058000	4.086585000
H	0.802772000	−0.846150000	4.772694000

123

theta3_thf_P2_trans_1,4_isoprene E = −2279.257585 a.u.

N	1.499483000	1.463299000	−0.727914000
P	2.983045000	−1.081003000	0.030626000
P	−1.406079000	2.244839000	−0.202649000
Si	−6.241361000	−3.631828000	0.266498000
C	−4.584857000	−3.241131000	1.131572000
H	−4.255075000	−4.154408000	1.648463000
H	−4.770397000	−2.499509000	1.923044000
C	−5.997679000	−5.066622000	−0.936206000

H	−5.289147000	−4.819299000	−1.736154000
H	−6.946354000	−5.335120000	−1.416451000
H	−5.625395000	−5.962621000	−0.424853000
C	−7.493390000	−4.100518000	1.596241000
H	−7.162586000	−4.975452000	2.168568000
H	−8.465521000	−4.347220000	1.152657000
H	−7.654311000	−3.280157000	2.305983000
C	−6.838720000	−2.108610000	−0.680438000
H	−6.931920000	−1.234796000	−0.023858000
H	−7.823816000	−2.291322000	−1.126485000
H	−6.155703000	−1.848813000	−1.498808000
C	3.831898000	−2.014817000	1.389744000
C	3.243701000	−2.044565000	2.662280000
H	2.284877000	−1.560477000	2.827143000
C	3.871584000	−2.698331000	3.720586000
H	3.403196000	−2.713290000	4.701349000
C	5.092825000	−3.340505000	3.519065000
H	5.580480000	−3.855963000	4.341873000
C	5.683020000	−3.321647000	2.256550000
H	6.634939000	−3.819432000	2.092178000
C	5.059801000	−2.662703000	1.197147000
H	5.532649000	−2.655340000	0.219837000
C	3.820365000	−1.715956000	−1.493229000
C	3.688118000	−3.073263000	−1.824918000
H	3.126628000	−3.741880000	−1.175921000
C	4.290331000	−3.580817000	−2.973361000
H	4.190570000	−4.636084000	−3.213252000
C	5.018891000	−2.736339000	−3.812892000
H	5.485870000	−3.131800000	−4.710709000
C	5.150650000	−1.387139000	−3.491697000
H	5.724141000	−0.726572000	−4.136596000
C	4.558435000	−0.877127000	−2.334916000
H	4.681794000	0.171699000	−2.082214000
C	3.617384000	0.635112000	0.215880000
C	2.791821000	1.699531000	−0.230411000

C	3.311154000	3.006276000	−0.090685000
H	2.715296000	3.848440000	−0.426469000
C	4.562748000	3.242602000	0.462249000
H	4.915366000	4.267106000	0.553789000
C	5.359813000	2.186095000	0.906175000
H	6.338524000	2.368530000	1.339607000
C	4.875006000	0.890026000	0.777896000
H	5.482084000	0.054487000	1.115587000
C	0.994643000	2.402900000	−1.654216000
C	1.791757000	2.867276000	−2.721737000
H	2.817868000	2.518635000	−2.792573000
C	1.289774000	3.734193000	−3.681941000
H	1.933762000	4.064190000	−4.493166000
C	−0.038459000	4.161324000	−3.621537000
H	−0.440487000	4.830801000	−4.376326000
C	−0.844884000	3.716763000	−2.580696000
H	−1.880001000	4.040583000	−2.534792000
C	−0.344986000	2.862548000	−1.587994000
C	−3.149953000	2.595244000	−0.712715000
C	−3.661062000	3.903236000	−0.714300000
H	−3.032965000	4.733952000	−0.404340000
C	−4.977275000	4.145373000	−1.100056000
H	−5.358987000	5.162855000	−1.100050000
C	−5.803063000	3.087084000	−1.480423000
H	−6.830304000	3.278143000	−1.778719000
C	−5.309762000	1.784146000	−1.468133000
H	−5.950949000	0.954388000	−1.752169000
C	−3.991619000	1.540042000	−1.083260000
H	−3.616631000	0.521857000	−1.073249000
C	−1.180130000	3.503882000	1.145851000
C	−0.113671000	4.408274000	1.149132000
H	0.587563000	4.417305000	0.321018000
C	0.038781000	5.319317000	2.195718000
H	0.868359000	6.021348000	2.179992000
C	−0.878603000	5.344759000	3.244589000

H	−0.766570000	6.063648000	4.051649000
C	−1.952929000	4.452760000	3.244479000
H	−2.684158000	4.478790000	4.048355000
C	−2.099523000	3.534762000	2.206341000
H	−2.957943000	2.866009000	2.202702000
C	−1.348649000	−0.353878000	−2.329720000
C	−0.193119000	−1.057826000	−2.732918000
C	0.198370000	−2.257507000	−2.117174000
C	−0.819830000	−3.348579000	−1.818639000
H	−0.715779000	−4.100539000	−2.616888000
H	−1.829247000	−2.945917000	−1.934975000
Y	0.108686000	−0.292137000	−0.243022000
H	1.176304000	−2.634269000	−2.414366000
O	0.211578000	0.217153000	2.203951000
C	−0.861388000	0.146622000	3.175012000
C	1.218269000	1.171022000	2.691112000
C	−0.211660000	0.501277000	4.498915000
H	−1.637639000	0.868944000	2.896945000
H	−1.273743000	−0.861916000	3.142534000
C	0.758819000	1.607724000	4.080851000
H	2.173818000	0.643155000	2.705023000
H	1.275898000	1.997231000	1.982687000
H	0.324791000	−0.364924000	4.903427000
H	−0.942733000	0.829371000	5.243214000
H	1.602470000	1.716622000	4.768041000
H	0.238241000	2.568202000	4.024812000
H	−1.652754000	0.530276000	−2.883345000
H	−2.178361000	−0.922334000	−1.899936000
C	−3.454504000	−2.742847000	0.208982000
C	−2.163764000	−2.617291000	0.976849000
H	−3.333314000	−3.434225000	−0.630341000
H	−3.752914000	−1.776272000	−0.221785000
H	−2.275955000	−2.101589000	1.932729000
C	−0.990634000	−3.253783000	0.749183000
C	−0.719949000	−4.093921000	−0.483174000

H	0.278606000	−4.540034000	−0.395419000
H	−1.423225000	−4.939947000	−0.499702000
C	0.750998000	−0.402826000	−3.713068000
H	0.647092000	0.684169000	−3.745432000
H	1.797083000	−0.657688000	−3.516363000
H	0.506958000	−0.787311000	−4.712949000
C	0.073595000	−3.332329000	1.812518000
H	−0.138253000	−2.704346000	2.683041000
H	0.143982000	−4.370328000	2.168506000
H	1.069803000	−3.084230000	1.428132000
